# Citrate reduced oxidative damage in stem cells by regulating cellular redox signaling pathways and represent a potential treatment for oxidative stress-induced diseases

**DOI:** 10.1016/j.redox.2018.11.015

**Published:** 2018-11-22

**Authors:** Xiaopei Wu, Honglian Dai, Langlang Liu, Chao Xu, Yixia Yin, Jiling Yi, Monika Dorota Bielec, Yingchao Han, Shipu Li

**Affiliations:** a State Key Laboratory of Advanced Technology for Materials Synthesis and Processing, Wuhan University of Technology, Wuhan 430070, PR China; b Biomedical Materials and Engineering Research Center of Hubei Province, Wuhan 430070, PR China

**Keywords:** Anti-oxidant, Citrate, Oxidative stress, Stem cells, ITRAQ

## Abstract

Chemical substances containing citrate such as calcium citrate, citrate esters and citric acid exhibit anti-oxidant and anti-inflammatory properties in different cells and tissues. However, data on the anti-oxidant and anti-inflammatory properties and mechanisms of action of citrate are insufficient. In this study, we systematically evaluated the anti-oxidant capacity of citrate using chemical, cellular and animal assays. Citrate showed a stable molecular structure and did not directly react with oxides. Citrate exerted protective and anti-apoptotic effects on BMSCs and also showed significant inhibitory effects on the oxidative stress and inflammatory reactions in the rat air pouch model. By using proteomics, we found that PPARγ contributed to the upregulation of various free radical scavenging proteins and the downregulation of diverse components of the inflammatory responses. Citrate-regulated global PPARγ expression was evidenced by the significant increase expression of PPARγ in PC12 cell line. Our results provide novel insights into the role of citrate in regulating cellular redox signaling and the function of PPARγ signaling in this process and also provide basic molecular cell biology information to improve the applications of biomaterials or stem cells as treatments for oxidative stress-induced degenerative diseases and inflammatory diseases.

## Highlights

• Citrate exerts anti-oxidant and anti-inflammatory properties in BMSCs and tissues.
• Citrate can upregulate and downregulate anti-oxidant and anti-inflammatory proteins in BMSCs.
• Citrate can regulate anti-oxidant and anti-inflammatory proteins via PPARγ dependent and independent pathways.

## Graphical abstract

**Figure:**
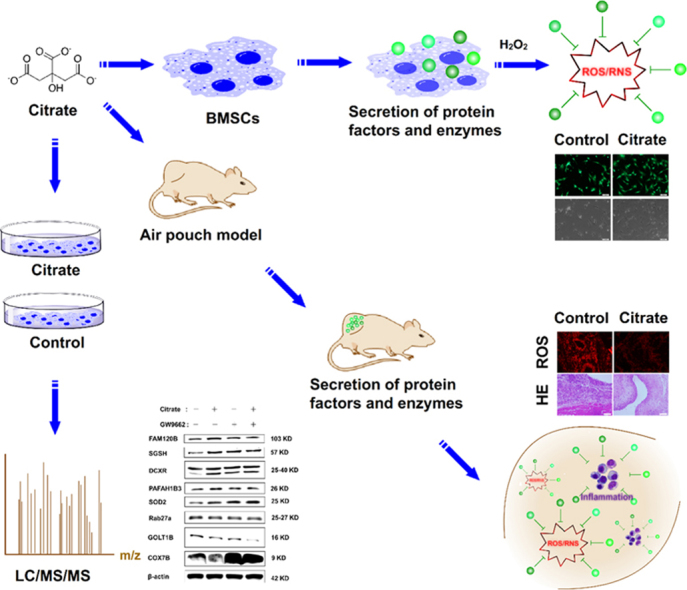

## 1 Introduction

Oxidative stress has been implicated in cardiovascular disease [1], pulmonary diseases [2], diabetes [3], neurodegenerative diseases [4], cancer [5], and inflammatory diseases [6]. Providing cells with anti-oxidant (vitamin C and E, carotenoids, and phenolics) may decrease intracellular oxidative stress levels [7], [8]. However, the direct reduction of the radical oxidant levels derived from oxidative stress requires a high dose of anti-oxidant supplementation, which may be unsafe [9]. Oral anti-oxidants do not improve the effects of cardiovascular disease or cancer treatments [10], [11].

Biomaterials that carry anti-oxidants, drugs or cells to the site of oxidative injury in the tissue and decrease intracellular oxidative stress levels in a sustained manner may be an efficient therapeutic approach for these medical problems. Numerous studies examining conjugation of small molecule anti-oxidants such as superoxide dismutase mimetics (mSOD), vitamin E, gallic acid, catechin, ascorbic acid and glutathione to ultra-high molecular weight poly (ethylene) (UHMPE), poly (acrylic acid), gelatin, poly (methyl methacrylate) and poly (ethylene glycol) have been reported [12], [13], [14], [15], [16]. Although this approach has resulted in the suppression of oxidative stress to some extent, the delivery of a low dose and unstable molecular structure of anti-oxidants in these materials have limited their long-term clinical use [17]. Therefore, biomaterials with native intrinsic anti-oxidant structural components may provide the benefits of relatively high anti-oxidant content and continuous local anti-oxidant release while the materials are degraded. Meanwhile, a chemically stable anti-oxidant small molecule, that is not based on consumable chemical defense (reacting with radicals) but on the perturbation of cellular redox signaling, is an essential anti-oxidant structural components of biomaterials that may provide the benefits of relatively high anti-oxidant content and continuous local anti-oxidant release with the degradation of the materials. In addition, small molecules which have diffusion coefficients an order of magnitude greater than large proteins are better suited for localized drug delivery applications [18], [19].

The small organic molecule citrate is an intermediate in the Krebs cycle in the mitochondria and is widely used in the food and pharmaceutical industries for its buffering capacity and anti-coagulant and anti-oxidant properties. Cellular redox signaling need to be regulated because disruptions in redox signaling may lead to organ-specific alterations in metabolic pathways in patients with oxidative diseases [20]. Calcium citrate and citrate esters have been shown regulate redox signaling by down-regulating levels of ROS and suppression of NF-ĸB activation in RAW264.7 cells [21], [22]. Citrate-based anti-coagulating have also exhibited the same biological functions including the suppression of inflammation and reductions in lipid peroxidation by reducing polymorphonuclear cell degranulation and the release of myeloperoxidase, elastase, interleukin-1β and platelet factor 4 [23], [24], [25], [26]. Trisodium citrate dihydrate reduced endothelial inflammation by inhibiting ICAM-1 expression and cytokine production [27]. We hypothesized that citrate would exert direct or indirect anti-oxidant effects due to its iron chelation activity and interaction with redox signaling pathways (regulation of the cellular anti-oxidant response). However, data on the anti-oxidant effects of citrate are insufficient, and thus the mechanisms of action of citrate must be investigated to determine its therapeutic potential.

**Fig. 1:**
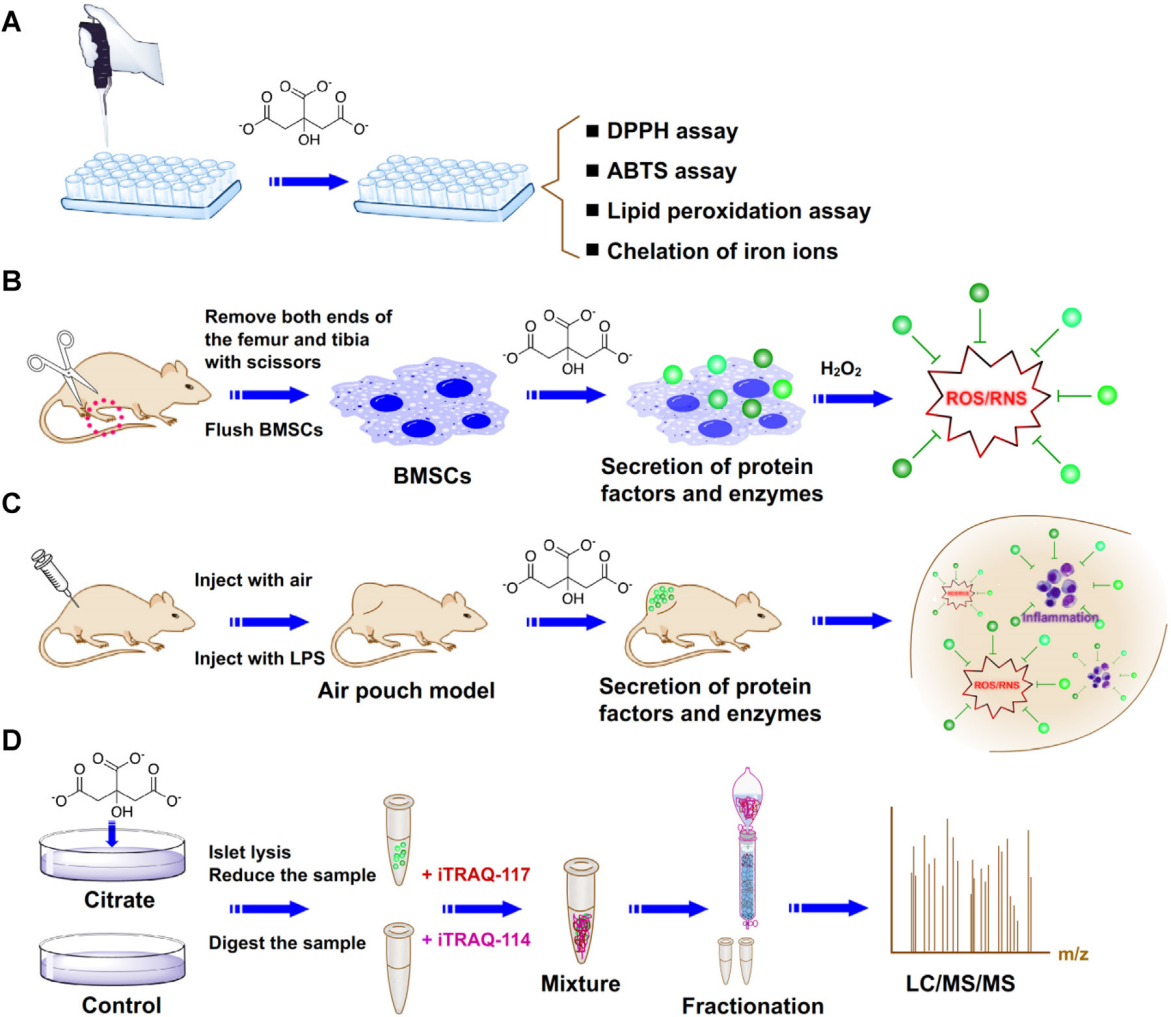
Schematic illustrating the investigation of the anti-oxidant effects and mechanisms of citrate in vitro and in vivo. (A) The anti-oxidant effects of citrate were investigated using chemical assays, including the DPPH assay, ABTS assay, lipid peroxidation assay and chelation of iron ions. (B) The anti-oxidant effects of citrate were investigated using cellular assays, including experiments examining the effects of citrate on metabolic activity, membrane morphology and apoptosis of BMSCs under oxidative stress. (C) The anti-oxidant effects of citrate were investigated using animal assays, including experiments designed to determine the effects of citrate on oxidative stress and inflammatory reactions in the air pouch model. (D) The anti-oxidant mechanisms of citrate were investigated by applying iTRAQ quantitative proteomics technology, bioinformatics analyses and functional studies.

In the present study, we first evaluated the anti-oxidant capacity of citrate using chemical, cellular and animal assays (Fig. 1A–C). Citrate did not show rapid free radical scavenging activity in DPPH (2,2-diphenyl-1-picrylhydrazyl) and ABTS (2,2′-azinobis-(3-ethylbenzothiazol e-6-sulphonate)) assays. Lipid peroxidation was not inhibited by citrate as assessed by the β-carotene bleaching assay. Citrate showed a stable molecular structure and did not directly react with oxides. In cellular assays, citrate exerted protective effects on bone marrow mesenchymal stem cells (BMSCs) under oxidative stress. However, a high concentration of citrate (5 mM) reduced cell viability and abolished the protective effects on cells. Therefore, citrate enhanced the anti-apoptotic ability of stem cells by inhibiting reactive oxygen species. An air pouch model was established for in vivo animal assays. Citrate exerted significant inhibitory effects on oxidative stress and inflammatory reactions induced by lipopolysaccharide (LPS).

Second, we performed proteomics assays to identify proteins regulated by citrate in BMSCs and further reveal the anti-oxidant mechanisms of citrate (Fig. 1D). By applying the iTRAQ quantitative proteomics analysis, we found that, the expression of 171 proteins was regulated by citrate compared to that of the control. A large number of differentially expressed proteins that exert potential anti-oxidant effects following citrate treatment was identified. Functional cluster analysis of these data at the systems level revealed major changes in proteins involved in nucleic acid binding, transcription factors, signaling molecules and enzyme modulators. Further functional studies revealed that the nuclear receptor peroxisome proliferator-activated receptor γ (PPARγ), a key protein involved in the transcription factors, contributes to the upregulation of various free radical scavenging proteins and the downregulation of diverse components of the inflammatory responses. By combining proteomics data with functional data, novel insights into the anti-oxidant mechanisms of citrate in BMSCs emerged. We postulate that the novel findings reported in this article will provide new routes to develop intrinsic anti-oxidant biomaterials with a relatively high anti-oxidant content and continuous local anti-oxidant release for both basic research and clinical therapy of oxidative stress-induced diseases.

## 2 Materials and methods

### 2.1 Materials

Trisodium citrate dihydrate was purchased from Sinopharm Chemical Reagent Co., Ltd (PR China). Lithium chloride anhydrous was purchased from Shanghai Macklin Biochemical Co., Ltd (PR China). 1,1-diphenyl-2-picrylhydrazyl (DPPH), PPARγ antagonist (GW9662), lipopolysaccharide (LPS), β-carotene-linoleic acid and linoleic acid were ordered from Sigma-Aldrich (USA). All chemicals were used as received without further purification. MEM Alpha Modification (α-MEM), Penicillin-Streptomycin and paraformaldehyde were obtained from HyClone (USA). Fetal bovine serum (FBS) was obtained from Every Green (PR China). Trypsin was obtained from Gibco (USA). ABTS, DCFH-DA, RIPA buffer and Dio assay kits were purchased from Beyotime Institute of Biotechnology (PR China). Protein quantitative detection, Malondialdehyde (MAD), Superoxide dismutase (SOD), Glutathione (GSH) and DHE assay kit were purchased from Servicebio Biological Technology Co., Ltd (PR China). Calcein-AM stain kit was purchased from Yeasen (PR China). Anti-FAM120B anti-body produced in rabbit, anti-PAFAH1B3 anti-body produced in rabbit, and anti-COX7B anti-body produced in rabbit were purchased from Abcam. Anti-GOLTIB anti-body derived from human were purchased from SAB. Anti-DCXR anti-body produced in rabbit were purchased from Zenbio. Anti-SOD2 anti-body produced in rabbit, anti-β-actin anti-body produced in rabbit, and anti-Rab27a anti-body produced in rabbit were purchased from Proteintech. Anti-mouse HRP-conjugated secondary anti-bodies were purchased from Proteintech. The water used in all experiments was purified using an Ulupure system (PR China).

### 2.2 Animals

Wistar rats were purchased from Hubei Provincial Center for Disease Control and Prevention (PR China). In each of the animal experiments, male Wistar rats weighing 100 g were used in each group. All animal experiments were conducted using protocol approved by the China National Institutional Animal Care and Use Committee.

### 2.3 Cell culture

Rat BMSCs were harvested from male Wistar rats. Briefly, under sterile conditions, the femur and tibia were dissected from male Wistar rats weighing 100 g, and all soft tissues attached to the bones were carefully removed. Both ends of the femur and tibia were removed with scissors, and then the bone marrow cavity was flushed with α-MEM containing 0.5% heparin. The cells were dispersed, centrifuged (1000 rpm, 10 min), resuspended in α-MEM supplemented with 20% FBS, seeded into 25 cm^2^ plastic flasks, and incubated at 37 ℃ in a humidified atmosphere with 5% CO_2_ for 24 h before the first medium change. Then, the medium was replaced every 3 days and the cells were used after 1 week. BMSCs were used at passage 2–5 in all experiments. The cultured BMSCs were then seeded at a density of 100,000 cells/cm^2^ in α-MEM supplemented containing 10% fetal bovine serum, 1% penicillin and streptomycin, and the medium was refreshed every 3 days. The PPARγ antagonist (GW9662) and lithium chloride were added 1 h before the citrate treatment. An equivalent amount of vehicle was used as a control when required.

### 2.4 Scavenging activity toward stable free radicals

DPPH and ABTS are two stable and colored free radicals that have been widely used to determine the cellular anti-oxidant capacity. For the DPPH assay, DPPH was dissolved in ethanol to form a 400 mM solution. Different concentrations of citrate were placed in DPPH solution (50 mg/mL) and incubated at 37 ℃. The changes in the DPPH content were measured with a microplate reader at 517 nm at each time point. For the ABTS assay, the amount of ABTS was measured using an assay kit. The ABTS assay was performed according to the manufacturer's instructions. At each time point, the absorbance of the ABTS solution was measured at 734 nm by using a microplate reader. All measurements were performed three times. The scavenging activity was measured as the percent inhibition of free radicals by measuring the decrease in absorbance compared to that of the control solutions.

### 2.5 The β-carotene bleaching assay

The anti-oxidant activity of citrate was evaluated using the β-carotene bleaching assay [21], [28]. Briefly, 4 g of tween-40, 4 mg of β-carotene and 0.5 mL of linoleic acid were mixed in 20 mL of chloroform. After removing the chloroform using a vacuum dryer, 30 mL of Britton buffer (100 mM, pH 6.5) were added to 1 mL of the oily residue with vigorous stirring. Citrate was added to 200 μL of the obtained emulsion to form different concentrations of reaction mixtures. The mixtures were incubated at 45 ℃ for 100 min and the absorbance was measured at 470 nm by using a microplate reader.

### 2.6 Iron chelation

The method used to inhibit the iron chelation activity was described previously [21]. Briefly, citrate was incubated with a 0.25 mM FeCl_2_·4H_2_O solution (50 mg/mL) at 37 ℃. Supernatants were collected at each time point and reacted with a 5 mM ferrozine indicator solution in a 5:1 ratio. The absorbance was measured at 534 nm.

### 2.7 Cell viability

BMSCs were plated in 96 well culture plates at a density of 2 × 10^3^ cells/well, and cultured with medium containing different concentrations of citrate for the indicated times. Then, cells were washed three times with PBS and incubated with MTT (0.5 mg/mL, 3-(4,5-dimethyl-2-thiazolyl)-2,5-diphenyl-2-H-tetrazolium bromide) at 37 ℃ for 4 h. The reagent was removed, and 150 μL of DMSO were added to each well. The number of viable cells was determined by measuring the absorbance at 490 nm using a microplate reader. The viability of BMSCs was further assessed by staining with Calcein-AM according to manufacturer's instructions to confirm the results obtained from the MTT assay. Cells were observed using an inverted fluorescence microscope (Olympus, Olympus IX71, Japan).

### 2.8 Cell membrane staining

The membranes of BMSCs were stained with DiO (3,3′-dioctadecyloxacarbocyanin eperchlorate). Cells were fixed with 4% (vol/vol) paraformaldehyde for 30 min at room temperature. Cell membranes were stained with DiO for 30 min at room temperature. After three washes with PBS for 15 min, cell membranes were observed under an inverted fluorescent microscope (Olympus, Olympus IX71, Japan).

### 2.9 ROS measurements

The measure of ROS is an integral part of redox biology. However, oxidant-sensitive fluorescent probes can provide partial information on cellular redox activity detecting ROS in cells and tissues [29]. We used two oxidant-sensitive fluorescent probes 2′7′-di-chlorofluorescein diacetate (DCFH-DA) and dihydroethidium (DHE) detect ROS in cells and tissues to obtain some oxidation status information. Intracellular ROS levels were measured using DCFH-DA fluorescent probe assay kit. The assay was performed according to the manufacturer's instructions. BMSCs were treated with citrate medium for 3 days followed by exposure to 5 mM tert-butyl hydroperoxide or 200 μM hydrogen peroxide for 1 h. The medium was removed from cells and cells were washed three times with PBS. Then cells were exposed to the DCFH-DA (10 μM) diluted by serum-free medium for 30 min at 37 ℃. Fluorescence was measured using an inverted fluorescent microscope (Olympus, Olympus IX71, Japan) at 488 nm (excitation) and 525 nm (emission) wavelengths. ROS levels in vivo were measured using DHE fluorescent probe assay kit. The assay was performed according to the manufacturer's instructions. Frozen slices protected from light was incubated with DHE (50 μM) diluted by PBS for 30 min at room temperature. The DHE was removed and slices were washed three times with PBS. Fluorescence was measured using an inverted fluorescent microscope (Olympus, Olympus IX71, Japan) at 535 nm (excitation) and 610 nm (emission) wavelengths.

### 2.10 Cell apoptosis evaluation

BMSCs were seeded on 25 cm^2^ plastic flasks. When cells were 80% confluent, the medium was replaced with citrate medium for 3 days. Then, hydrogen peroxide (H_2_O_2_) was added to the citrate medium to a final concentration of 200 μM. After 1 day of culture, BMSCs apoptosis was analyzed using flow cytometric (CytoFLEX, Beckman Coulter, USA) by staining the cells with Annexin V-FITC Apoptosis Detection Kit (Becton Dickinson, USA), according to the manufacturer's protocol.

### 2.11 In vivo animal assays

An air pouch model was used to examine inhibitory effects of citrate on LPS-induced oxidative stress and inflammatory reactions [30]. An area of the dorsal skin (2 cm^2^) was cleaned with alcohol and shaved to provide the pouch site. The subcutaneous tissue on the back of each rat was injected with 10 mL of air. Subsequently, 5 mL of air were injected into the same cavity every 3 days to maintain an open cavity. On day 6, the pouches were injected with 2 mL of LPS (0.5 mg/mL). On day 12, the pouches were injected with 2.5 mL of a suspension containing the control, citrate, stem cells and stem cells/citrate. Control pouches received 2.5 mL of sterile PBS alone. Rats were sacrificed 72 h after the introduction of citrate into the pouches. The pouches were then dissected from the surrounding tissue. The pouch was divided with one-third fixed in paraformaldehyde for the histological evaluation and the remainder was snap-frozen at −80 ℃ for RNA extraction (IL-1, IL-1β, IL-6 and TNF-α assays), the collection of tissue supernatants (MAD, SOD and GSH assays) and the production of frozen slices (ROS assay).

### 2.12 Histological evaluation

The pouches were fixed, dehydrated and embedded in paraffin blocks. Sections were cut along the longitudinal axis of the pouches. Then, sections were mounted and stained with hematoxylin and eosin (H&E). After staining, the slides were permanently covered with cover slips. Means of three separate sections were evaluated in a random fashion using the Image J image analysis software package. The total numbers of neutrophils were determined as cells per mm^2^ based upon counts of the nuclei.

### 2.13 Redox state in the pouches

Tissue supernatants were harvested at different treatment groups and the levels of Malondialdehyde (MAD), Superoxide dismutase (SOD) and Glutathione (GSH) were measured using assay kits. The assays were performed according to the manufacturer's instructions.

### 2.14 Real time quantitative PCR

Total RNA was isolated using TRIzol (Invitrogen), phase-separated with chloroform, and precipitated using isopropanol. RNA was collected in RNAase-free water and the total quantity was analyzed by spectrophotometry. First strand cDNAs were synthesized from total RNA using the Revert Aid First Strand cDNA Synthesis Kit (Thermo) according to the manufacturer's instructions. 2.5 μL of undiluted cDNAs was used for quantitative real time PCR performed on a Light Cycler PCR machine (Roche) using Fast Start Universal SYBR Green Master (Roche). The following temperature profile was used: initial denaturation at 95 ℃ for 10 min for one cycle, 40 cycles of 15 s at 95 ℃, 60 s at 60 ℃, and then 95 ℃ for 10 min. The primer sequences are as follows: IL1 (forward: 5´-GCTAAGTTTCAATCAGCCCTTTAC-3´, reverse: 5´- CATGATGAACTCCTGCTTGACG)-3´), IL6 (forward: 5´-AGGATACCACCCACAACAGACC-3´, reverse: 5´-TTGCCATTGCACAACTCTTTTC-3´), IL1β (forward: ´-TGACCTGTTCTTTGAGGCTGAC-3´, reverse: 5´-CATCATCCCACGAGTCACAGAG-3´), TNF-α (forward: 5´-CCAGGTTCTCTTCAAGGGACAA-3´, reverse: 5´-GGTATGAAATGGCAAATCGGCT-3´), and β-actin (forward: 5´-TGCTATGTTGCCCTAGACTTCG-3´, reverse:5´-GTTGGCATAGAGGTCTTTACGG-3´).

### 2.15 Protein extraction

Samples were suspended in lysis buffer (8 M urea, 2 mM EDTA, 10 mM DTT and 0.1% Cocktail Set IV), and then sonicated for 5 min on ice using a high intensity ultrasonic processor (Scientz). The remaining debris were removed by centrifugation at 20,000 g at 4 ℃ for 10 min. Protein concentrations were quantified using a 2D-Quant Kit (GE Healthcare) and SDS-PAGE was performed. Trypsin was added to the protein solution at a trypsin to protein ratio of 1:50 (w/w) and digested at 37 ℃ for 16 h. Dithiothreitol was then added to a final concentration of 5 mM, followed by an incubation at 50 ℃ for 30 min. Then, iodoacetamide was added to alkylate proteins with final concentration 15 mM followed by an incubation at 25 ℃ in dark for 30 min. The alkylation reaction was quenched by 30 mM of cysteine at 25 ℃ for another 30 min. Trypsin was then added again with ratio of trypsin to protein at 1:100 (w/w) for digestion at 37 ℃ for 3 h to complete the digestion cycle. Peptides were fractionated using strong-cation exchange (SCX). In brief, SCX was performed using a Zorbax BioSCX-Series II column (0.8 mm × 50 mm, 3.5 µm). Solvent A consisted of 0.05% formic acid (FA) in 20% acetonitrile (ACN), solvent B consisted of 0.05% formic acid and 0.5 M NaCl in 20% ACN. The following gradient was used: 0–0.01 min (0–2%B); 0.01–8.01 min (2–3%B); 8.01–14.01 min (3–8%B); 14.01–28 min (8–20%B); 28–38 min (20–40% B); 38–48 min (40–90%B); 48–54 min (90%B); and 54–60 min (0%B). Each sample was labeled by iTRAQ according to the manufacturer's instructions (Thermo Scientific). Fourteen fractions were collected and dried in vacuo.

### 2.16 LC-MS/MS analysis

The dried peptides from each gel slice were dissolved in RP-solvent A (0.1% FA in 2% ACN) and directly loaded onto a reversed-phase pre-column (Acclaim PepMap 100, Thermo Scientific). Peptide separation was performed using a reversed-phase analytical column (Acclaim PepMap RSLC, Thermo Scientific) with a linear gradient of 6–22% RP-solvent B (0.1% FA in 98% ACN) for 22 min, 22–34% solvent B for 8 min and 34–90% solvent B for 3 min at a constant flow rate of 300 nl/min on an EASY-nLC 1000 UPLC system. The resulting peptides were analyzed by Q Exactive hybrid quadrupole-Orbitrap mass spectrometer (Thermo Fisher Scientific). Peptides were subjected to an NSI source followed by tandem mass spectrometry (MS/MS) in Q Exactive (Thermo) coupled online to the UPLC. Intact peptides were detected in the Orbitrap at a resolution of 70000. Peptides were selected for MS/MS using 28% NCE; ion fragments were detected in the Orbitrap at a resolution of 17500. The electrospray voltage applied was 2.0 kV. For MS scans, the *m/z* scan range was 350–1800 Da.

### 2.17 Data processing and protein quantification

Raw data files were processed to generate peak list files using Proteome Discoverer software (Thermo Fisher Scientific, v.1.3.0.339). The resulting MS/MS data were processed using MaxQuant software (v.1.4.1.2) with the integrated Andromeda search engine. The M. tuberculosis protein database used for MS/MS searches was downloaded from NCBI ftp site (http://www.ncbi.nlm.nih.gov/) containing 4018 protein sequences. Trypsin/P was specified as the cleavage enzyme allowing up to 2 missed cleavages. The precursor charge states were allowed from 1 to 5. The mass error was set to 10 ppm for precursor ions and 0.02 Da for fragment ions. Carbamidomethylation (C), TMT6plex (K) and TMT6plex (N-term) were set as fixed modifications. Oxidation of methionine (M) was set as a variable modification. Detection of at least two matching peptides per protein was set as a requirement for unambiguous identification. The TMT datasets were quantified using the centroid peak intensity with the “reporter ions quantifier” mode. For all experiments only unique peptides were considered for protein quantification. The peptide false discovery rate (FDR) was set to 1% and minimum peptide score was set to 13.0. The minimum peptide length was set to 7. All the other parameters in MaxQuant were set to default values. For data analyses, only the proteins identified in both of the two independent experiments were used. Protein ratios were log2 transformed and the frequency distribution of the quantified proteins was calculated to determine differentially expressed proteins.

### 2.18 Bioinformatics analysis

Gene Ontology (GO) annotation proteome was derived from the UniProt-GOA database (http://www.ebi.ac.uk/GOA/). Firstly, protein IDs was converted to UniprotKB ID and then mapped to GO ID by protein ID. Then proteins were further classified by Gene Ontology annotation based on three categories: biological process, cellular component and molecular function. The Kyoto Encyclopedia of Genes and Genomes (KEGG) database was used to annotate protein pathway. Firstly, the KEGG online service tools KAAS was used to annotate protein's KEGG database description. Then annotation result was mapped on the KEGG pathway database using KEGG online service tools KEGG mapper. Domain annotation was performed by using InterProScan on the InterPro domain database via Web-based interfaces and services. Cello was used for subcellular localization predication. All quantified proteins were searched against the STRING database version 9.1 for protein-protein interactions. Only interactions between the proteins contained in the searched data set were selected. STRING defines a metric called the “confidence score” to define interaction confidence: interactions are fetched when a confidence score ≥0.7 (high confidence) is reached. Interaction networks obtained form STRING were visualized using Cytoscape software.

### 2.19 Western blotting

Confluent monolayer cells were lysed in RIPA buffer. Samples were then centrifuged at 12,000 g for 5 min at 4 ℃. The protein concentrations of cell lysates were determined with a protein quantitative detection assay kit. Total proteins were separated by SDS-PAGE. After electrophoresis, proteins were transferred to a PVDF membrane using a liquid transblot system (Bio-Rad). Membranes used for the immunodetection of proteins were blocked with 5% skim milk for 60 min at room temperature. Diluted primary antibodies (1:1000) bound to the membrane were detected with HRP-conjugated anti-mouse secondary antibodies diluted 1:3000 in 5% skim milk in TBST (TBS/Tween20). Blots were visualized by enhanced chemiluminescence (ECL) using a Western blotting detection system.

### 2.20 Statistical analysis

Each experiment was performed three times with similar results. All statistical analyses were performed by one-way analysis of variance (ANOVA) using SPSS 18 software (IBM SPSS, Armonk, New York, USA). All data are presented as the means ± standard deviations (SD). The level of significance was set to P < 0.05.

## 3 Results

### 3.1 Anti-oxidant properties of citrate in chemical assays

**Fig. 2:**
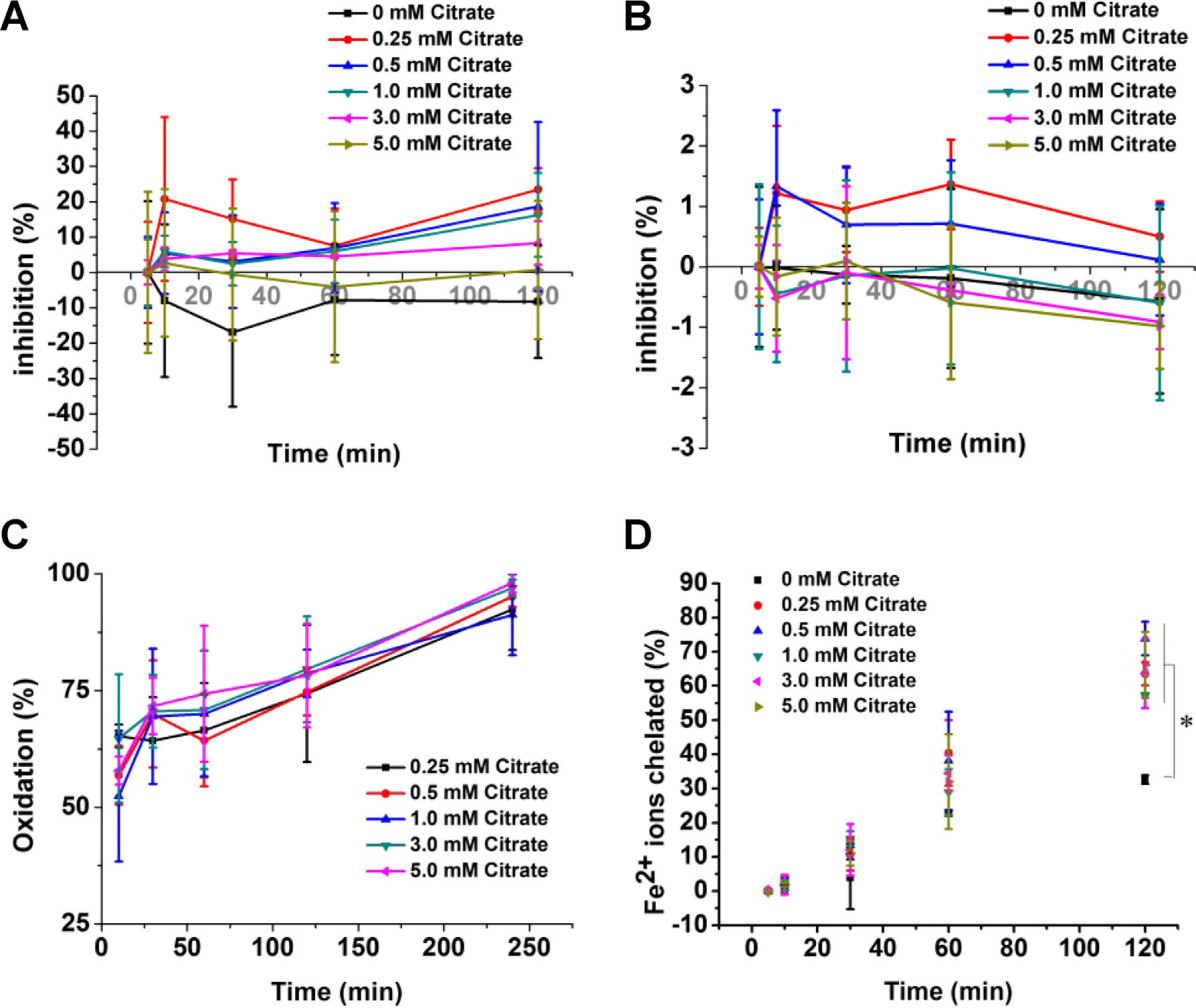
Determination of the anti-oxidant capacity of citrate using chemical assays. DPPH (A) and ABTS (B) assays revealed a lack of radical- scavenging activity for different concentrations of citrate. (C) Different concentrations of citrate did not inhibit lipid peroxidation. (D) Different concentrations of citrate chelate iron ions. *p < 0.05, compared to 0 mM citrate.

We first evaluated the anti-oxidant capacity of citrate using chemical assays. Citrate did not show rapid free radical scavenging activity in DPPH (Fig. 2A) and ABTS (Fig. 2B) assays within 2 h. DPPH and ABTS are two colored free radicals that have been widely used to determine the anti-oxidant capacity. Citrate did not transfer electrons and protons to DPPH or oxidize ABTS. Lipid peroxidation was not inhibited by citrate as assessed by the β-carotene bleaching assay (Fig. 2C). Ferrous ions chelated by citrate will not be able to participate in the ferrozine reaction, resulting in lower absorbance. As expected, citrate showed strong iron chelation properties at different concentrations (Fig. 2D). Citrate exhibited a stable molecular structure and did not participate in direct reactions with oxide; however, the metal iron chelation property of citrate may prevent reactions with free radicals under physiological conditions.

### 3.2 Protective effects of citrate on BMSCs under oxidative stress

**Fig. 3:**
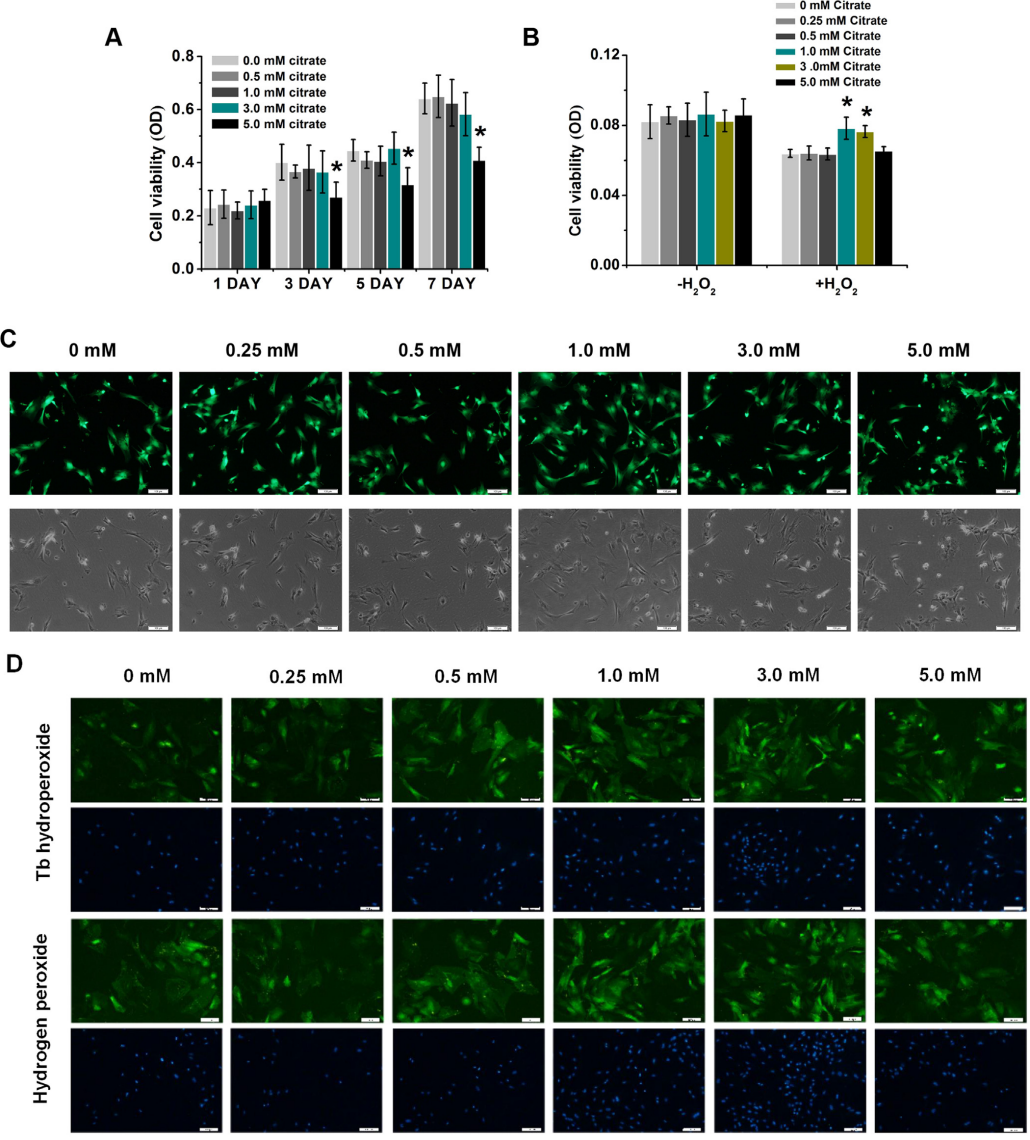
Protective effects of citrate on the viability and membrane of BMSCs under oxidative stress. (A) BMSCs were incubated with 0, 0.5, 1.0, 3.0 and 5.0 mM citrate medium. The viability of BMSCs was significantly decreased after 3 days of culture with 5.0 mM citrate compared with the 0, 0.5, 1.0, 3.0 mM citrate medium. (B) BMSCs were exposed to 0, 0.25, 0.5, 1.0, 3.0 and 5.0 mM citrate medium for 3 days. After hydrogen peroxide (H_2_O_2_) challenge, both 1.0 and 3.0 mM citrate protected against increasing levels of oxidative stress. (C) BMSCs cultured in citrate medium for 3 days showed prolonged survival upon hydrogen peroxide stimulation for 12 h. Green indicates Calcein-AM staining in live cells. Bar = 100 µm. (D) BMSCs were stained with DiO to label the cell membrane (green) after 3 days of incubation with 0, 0.25, 0.5, 1.0, 3.0 and 5.0 mM citrate medium. The stem cells showed damaged cell membrane treated with 0, 0.25, 0.5 and 5.0 mM citrate in the medium under oxidative stress, while treated with 1.0 and 3.0 mM citrate in the medium showed rather integrated morphology. Bar = 100 µm. Means ± SD. *p < 0.05, compared to 0 mM citrate.

**Fig. 4:**
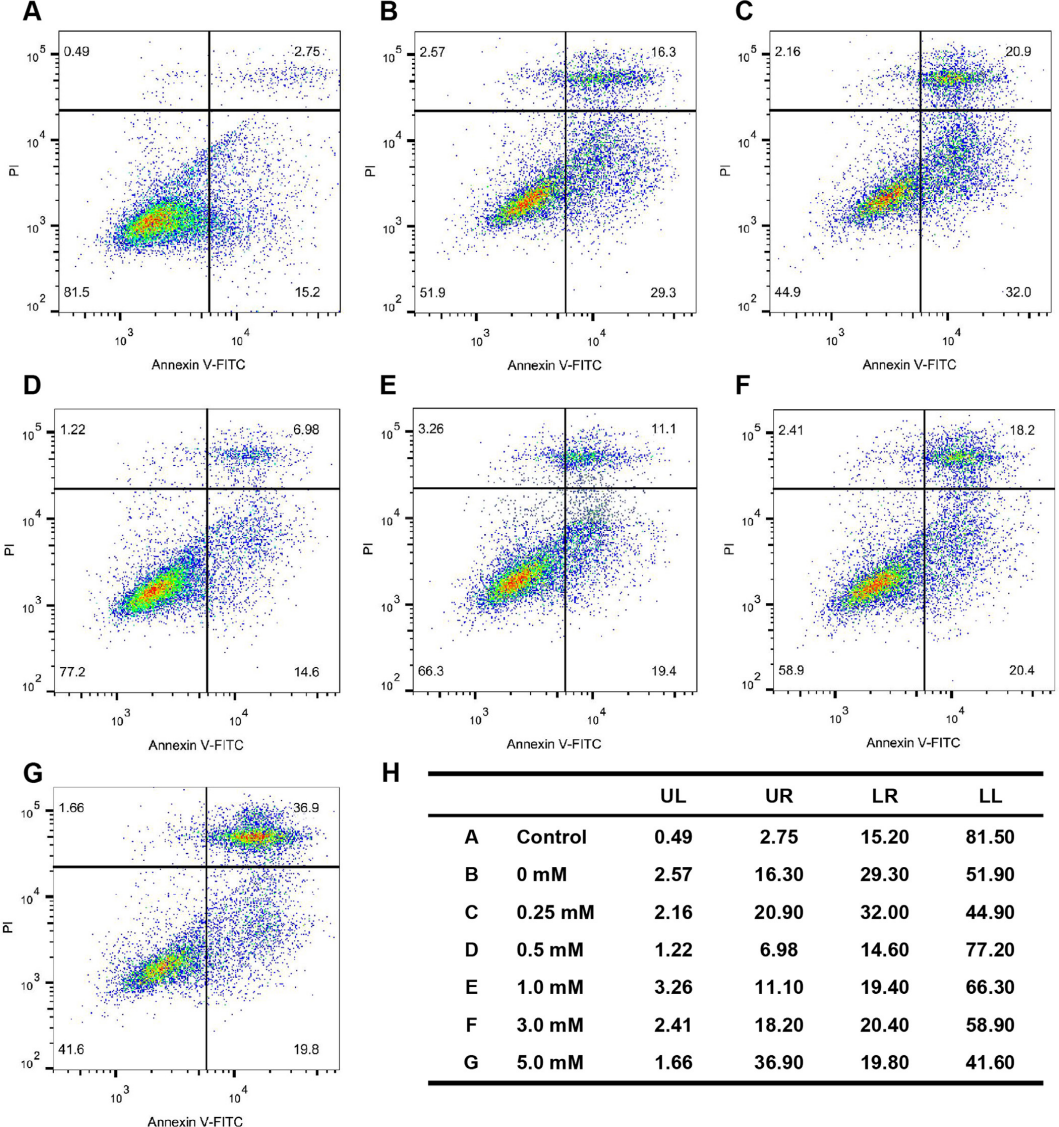
Protective effects of citrate on the apoptosis of BMSCs under oxidative stress. Stem cells were cultured in control (A), 0 (B), 0.25 (C), 0.5 (D), 1.0 (E), 3.0 (F) and 5.0 (G) mM citrate medium for 3 days and then treated with 200 μM hydrogen peroxide for 12 h to measure the effect of citrate on BMSCs apoptosis following an ROS challenge. The 0.5, 1.0 and 3.0 mM citrate medium indeed significantly decreased the rate of BMSCs apoptosis compared with that of the 0, 0.25 and 5.0 mM citrate. (H) Summary of apoptotic BMSCs. Control, stem cells without any treatment.

According to previous studies, chemical substances containing citrate such as calcium citrate, citrate esters, citric acid and citrate anti-coagulants exhibit anti-oxidant and anti-inflammatory properties in different cells and tissues. Although the same conclusions are based on the anti-oxidant performance of different materials, they possess same molecular structure as citrate. Therefore, citrate may play an important role in protecting BMSCs from oxidative stress-induced damage. We evaluated the response of stem cells treated with 0, 0.5, 1.0, 3.0 and 5.0 mM citrate using the MTT assay to obtain an understanding of the effects of the concentrations of citrate ions on the metabolic activity of BMSCs. The viability of BMSCs was significantly decreased after 3 days of treatment with 5.0 mM citrate compared with the 0, 0.5, 1.0 and 3.0 mM citrate (Fig. 3A). To measure the citrate effects on stem cells viability upon an ROS challenge, BMSCs were cultured in 0, 0.25, 0.5, 1.0, 3.0 and 5.0 mM citrate medium for 3 days. Then, BMSCs were treated with 200 μM hydrogen peroxide (H_2_O_2_) for 12 h, this treatment is known to induce rapid cell death due to excess ROS generation. According to the results of the MTT assay, BMSCs cultured in 0, 0.25, 0.5 and 5.0 mM citrate medium, showed low viability after 12 h of treatment with 200 μM H_2_O_2_; however, stem cells cultured in 1.0 and 3.0 mM citrate medium maintained their viability (Fig. 3B). This finding was corroborated by the increased fluorescence intensity of Calcein-AM staining after 3 days of culture in 1.0 and 3.0 mM citrate medium and decreased intensity after culture in 0, 0.25, 0.5 and 5.0 mM citrate medium (Fig. 3C). BMSCs were also stained with DiO fluorescent dyes for cell membrane (green) respectively after 5 mM tert-butyl hydroperoxide or 200 μM hydrogen peroxide (H_2_O_2_) challenge. The results, shown in Fig. 3D, revealed that the BMSCs showed damaged cell membrane after treated with 0, 0.25, 0.5 and 5.0 mM citrate in the medium, while the stem cells treated with 1.0 and 3.0 mM citrate in the medium showed a rather integrated morphology. Because oxidative stress usually induces apoptosis, we used flow cytometry analysis to examine the percentage of apoptotic cells. BMSCs were cultured in 0, 0.25, 0.5, 1.0, 3.0 and 5.0 mM citrate medium for 3 days. Then, BMSCs were treated with 200 μM hydrogen peroxide (H_2_O_2_) for 12 h. The 0.5, 1.0 and 3.0 mM citrate medium indeed significantly decreased the rate of BMSCs apoptosis compared with that of the 0, 0.25 and 5.0 mM citrate (Fig. 4). Thus, appropriate concentrations of citrate ions exert positive effects on the metabolic activity, membrane morphology and apoptosis of BMSCs under oxidative stress.

### 3.3 The Na-coupled citrate transporter contributes to the anti-oxidative effects of citrate on BMSCs

**Fig. 5:**
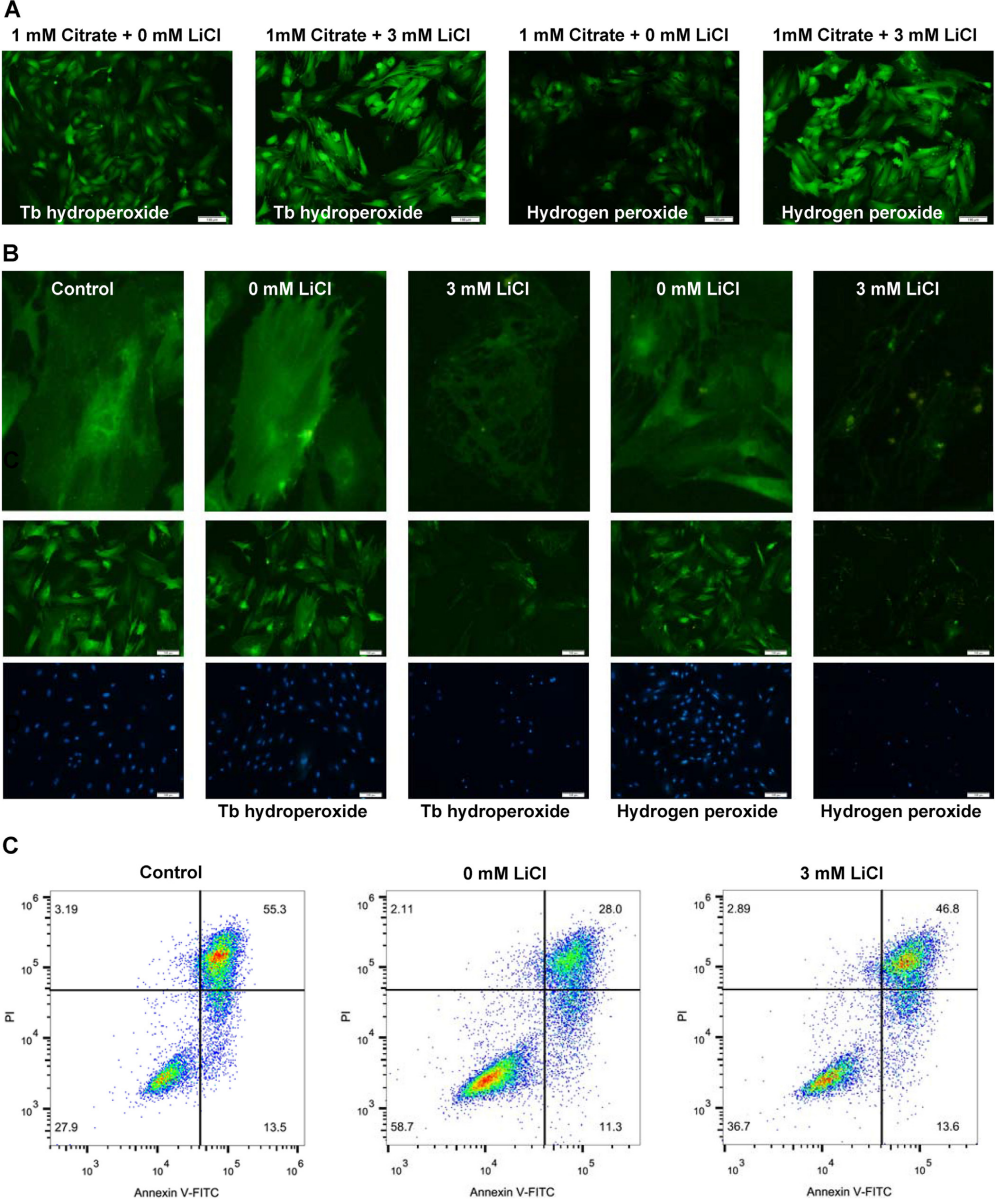
The role of the Na-coupled citrate transporter in citrate-mediated anti-oxidant functions in BMSCs. (A) The effects of the Na-coupled citrate transporter on the ROS levels were evaluated in BMSCs treated with 1.0 mM citrate for 3 days followed by exposure to 5.0 mM tert-butyl hydroperoxide or 200 μM hydrogen peroxide for 1 h. Bar = 100 µm. (B) Staining with the fluorescent dye DiO revealed damaged membranes in BMSCs treated with 3 mM lithium chloride medium containing 1.0 mM citrate, while cells treated with 0 mM lithium chloride showed a rather integrated morphology. Bar = 100 µm. (C) A significant decrease in the rate of apoptosis was observed in BMSCs cultured in 1.0 mM citrate medium and subsequently exposed to 200 μM hydrogen peroxide for 12 h; this decrease was abrogated by 3 mM lithium chloride.

The Na-coupled citrate transporter in the rat liver cell line exhibits higher affinity for citrate than that of the transporter in human liver cell lines. We used lithium chloride, an inhibitor of Na-coupled citrate transporter in the rat liver cell line, to investigate the mechanism by which citrate in the extracellular milieu enhances the cellular anti-oxidant capacity [31]. The effects of the Na-coupled citrate transporter on ROS levels were evaluated in BMSCs treated with 1.0 mM citrate medium for 3 days followed by exposure to 5 mM tert-butyl hydroperoxide or 200 μM hydrogen peroxide for 1 h. Inhibition of the Na-coupled citrate transporter significantly increased ROS levels compared with those of the non-suppressed group (Fig. 5A). We next examined the effect of the Na-coupled citrate transporter on the membranes of BMSCs by staining the cells with the fluorescent dye DiO. As shown in Fig. 5B, the membranes of BMSCs were damaged following treatment with 3 mM lithium chloride medium containing 1.0 mM citrate, while the stem cells treated with 0 mM lithium chloride showed a rather integrated morphology. A significant decrease in the rate of apoptosis was observed in BMSCs cultured in 1.0 mM citrate medium and subsequently exposed to 200 μM hydrogen peroxide for 12 h, and this decrease was abrogated by 3 mM lithium chloride (Fig. 5C). Thus, the Na-coupled citrate transporter is important for maintaining the anti-oxidative effects of citrate on BMSCs.

### 3.4 Inhibitory effects of citrate on oxidative stress and inflammatory reactions in vivo

**Fig. 6:**
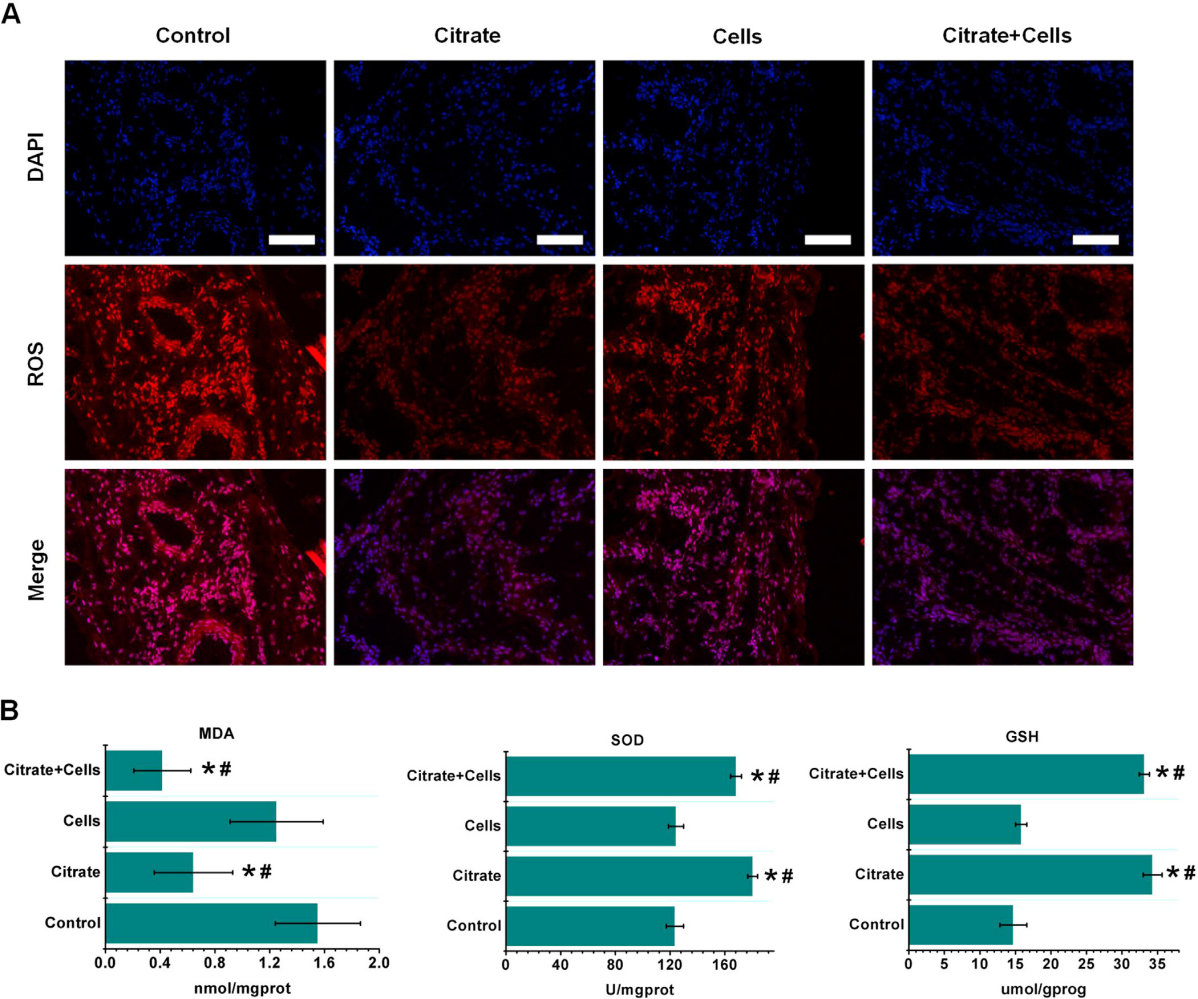
Inhibitory effects of citrate on ROS production in the LPS-stimulated air pouch model. (A) Citrate showed significant inhibitory effects on ROS generation induced by LPS. Bar = 100 µm. (B) SOD and GSH production were significantly increased by citrate, while treatment with citrate significantly reduced the MDA levels in the LPS-stimulated pouch. N = 3, means ± SD. *p < 0.05, compared to control. #p < 0.05, compared to cells.

**Fig. 7:**
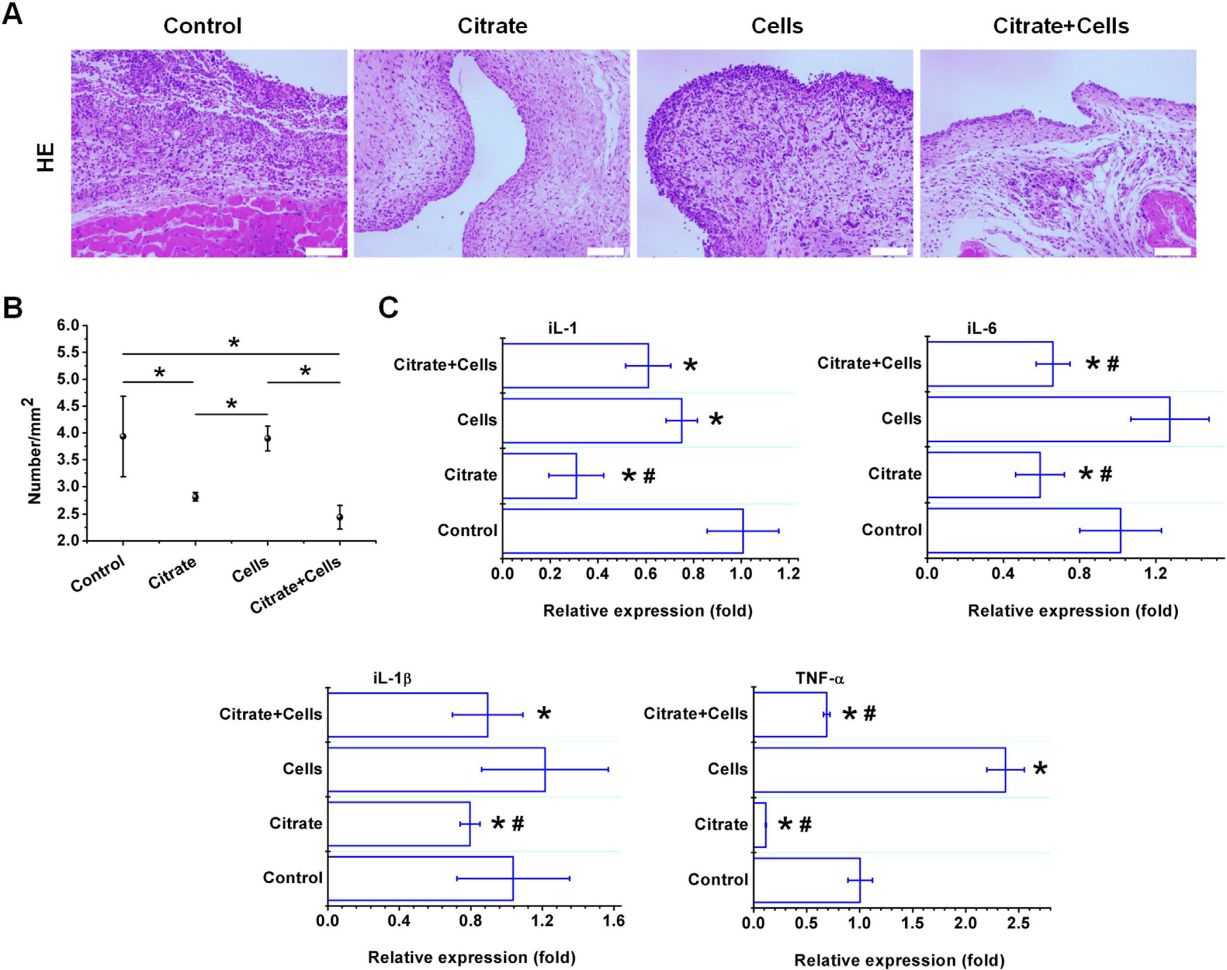
Inhibitory effects of citrate on inflammatory reactions in the LPS-stimulated air pouch. (A) The number of inflammatory cells exuded from the pouch was detected by HE staining. The introduction of citrate into the air pouch dramatically decreased the histological parameters of exuded inflammatory cells, compared with the parameters of the control or stem cells pouch membranes. Bar = 100 µm. (B) The objective measurements of changes in inflammatory cells counts were determined by analyzing images of the histological sections. (C) Exposure to LPS increased the levels of the IL-1, IL-1β, IL-6 and TNF-α mRNAs in the air pouch. However, the citrate treatment reduced levels of the IL-1, IL-1β, IL-6 and TNF-α mRNAs in the LPS-stimulated air pouch. N = 3, means ± SD. *p < 0.05, compared to control. #p < 0.05, compared to cells.

Citrate exhibited both anti-oxidant and anti-inflammatory properties in BMSCs. Accurate models of oxidative stress and the inflammatory response must be established to evaluate the anti-oxidant and anti-inflammatory capabilities of citrate in vivo. We have utilized the rat air pouch model (Fig. S1) to investigate the oxidation level and inflammatory response to a lipopolysaccharide (LPS)-induced air pouch after treatment by 1.0 mM citrate and stem cells. In vivo, LPS first activates macrophages and neutrophils to increase ROS production and ROS mediated-signaling pathways, subsequently generating inflammatory cytokines and chemokines in leukocytes [32], [33], [34], [35], [36]. Citrate exerted significant inhibitory effects on ROS generation (Fig. 6A). SOD and GSH production were significantly increased by citrate, while the citrate treatment significantly reduced the MDA levels (Fig. 6B). The introduction of citrate into the air pouch dramatically decreased the histological parameters of cellular infiltration compared with the parameters of the control or stem cells in pouch membranes (Fig. 7A). The objective measurements of changes in inflammatory cell counts in response to the different treatments, as determined by an analysis of images of the histological sections (Fig. S2), are summarized in Fig. 7B. The expression of inflammatory cytokine genes in membrane extracts was analyzed by RT-PCR and the results are shown in Fig. 7C. Exposure to LPS increased the levels of the IL-1, IL-1β, IL-6 and TNF-α mRNAs in the air pouch. However, the citrate treatment reduced IL-1, IL-1β, IL-6 and TNF-α mRNAs in the air pouch. In summary, citrate exerts significant inhibitory effects on LPS-induced oxidative stress and inflammatory reactions.

### 3.5 Identification of citrate-regulated proteins using a quantitative proteomic approach

**Fig. 8:**
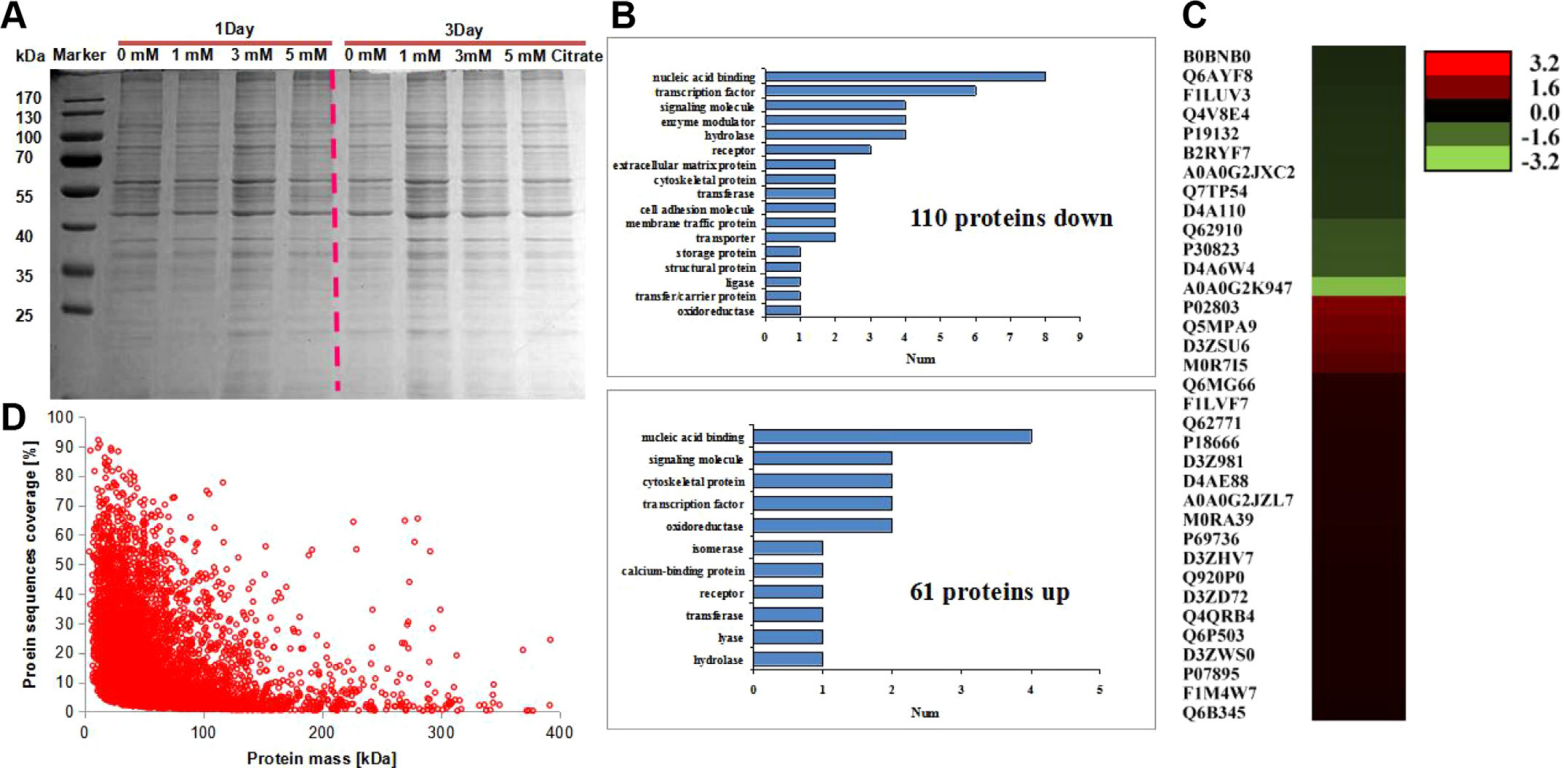
Quantitative proteomic identification of citrate-regulated proteins in BMSCs. (A) Proteins were extracted from BMSCs after an incubation with 1.0 mM citrate for 3 days, and then the proteins were separated by SDS-PAGE. (B) A total of 3998 proteins were identified, 110 of which were down-regulated and 61 were up-regulated. (C) The heatmap shows the expression of differentially expressed proteins in BMSCs incubated with 1.0 mM citrate for 3 days. (D) The distribution of the quantified peptides is shown.

Because inflammatory cytokines were expressed at high levels in BMSCs, we deduced that the citrate treatment would alter the expression of their target genes. Cells treated with citrate medium exhibited reduced expression of inflammatory cytokine mRNAs, and we therefore employed an iTRAQ-based quantitative proteomics approach to identify proteins that were regulated by citrate in BMSCs. We first examined the molecular composition of the expressed proteins in BMSCs treated with 1.0, 3.0 and 5.0 mM citrate medium using SDS-PAGE. The isolated proteins were shown in Fig. 8A. Proteins isolated from different groups showed complex banding patterns, indicating that the expressed proteins consist of many different proteins. Proteins in BMSCs treated with 1.0 mM citrate for 3 days showed increase in net lane intensity that reflects an increase in the total adsorbed protein density. We digested the isolated proteins with trypsin and analyzed the resulting peptide mixtures using a quantitative proteomic approach to obtain a better understanding of this phenomenon. A total of 3998 proteins were identified, 110 of which were down-regulated and 61 were up-regulated (Fig. 8B and C). The distribution of quantified peptides is shown in Fig. 8D and Fig. S3. The identified proteins were initially classified based on the UniProt database. Anti-oxidant and anti-inflammatory proteins were listed in Table S1. All raw data have been deposited in Table S5.

### 3.6 Bioinformatics analysis of citrate-regulated proteins

We first classified the citrate-regulated proteins using PANTHER Protein Class Ontology to evaluate the biological relevance of these proteins [37]. The proteins assigned to PANTHER Protein Class Categories were classified into 20 classes (Fig. 8B), the five largest of which were the nucleic acid binding, transcription factor, signaling molecule, enzyme modulator and hydrolase classes. We next classified the citrate-regulated proteins according to the Gene Ontology (GO) molecular function, biological process and cellular components to obtain an overview of the distribution of GO terms (Fig. S4). Based on the results of the GO molecular function analysis, the citrate-regulated proteins are involved in a variety of biological processes, such as catalytic activity, anti-oxidant activity, signal transducer activity, nucleic acid binding and molecular transducer activity (Fig. S4). Then proteins were further classified by Gene Ontology annotation based on three categories: biological process, cellular component and molecular function. The Kyoto Encyclopedia of Genes and Genomes (KEGG) database was used to annotate protein pathways. Many citrate-regulated proteins were assigned to the transcription factor, enzyme modulator and metabolic pathways (Fig. S5–7). Based on these bioinformatics results, citrate may be a critical regulator of diverse cellular functions distributed among various biological processes.

**Fig. 9:**
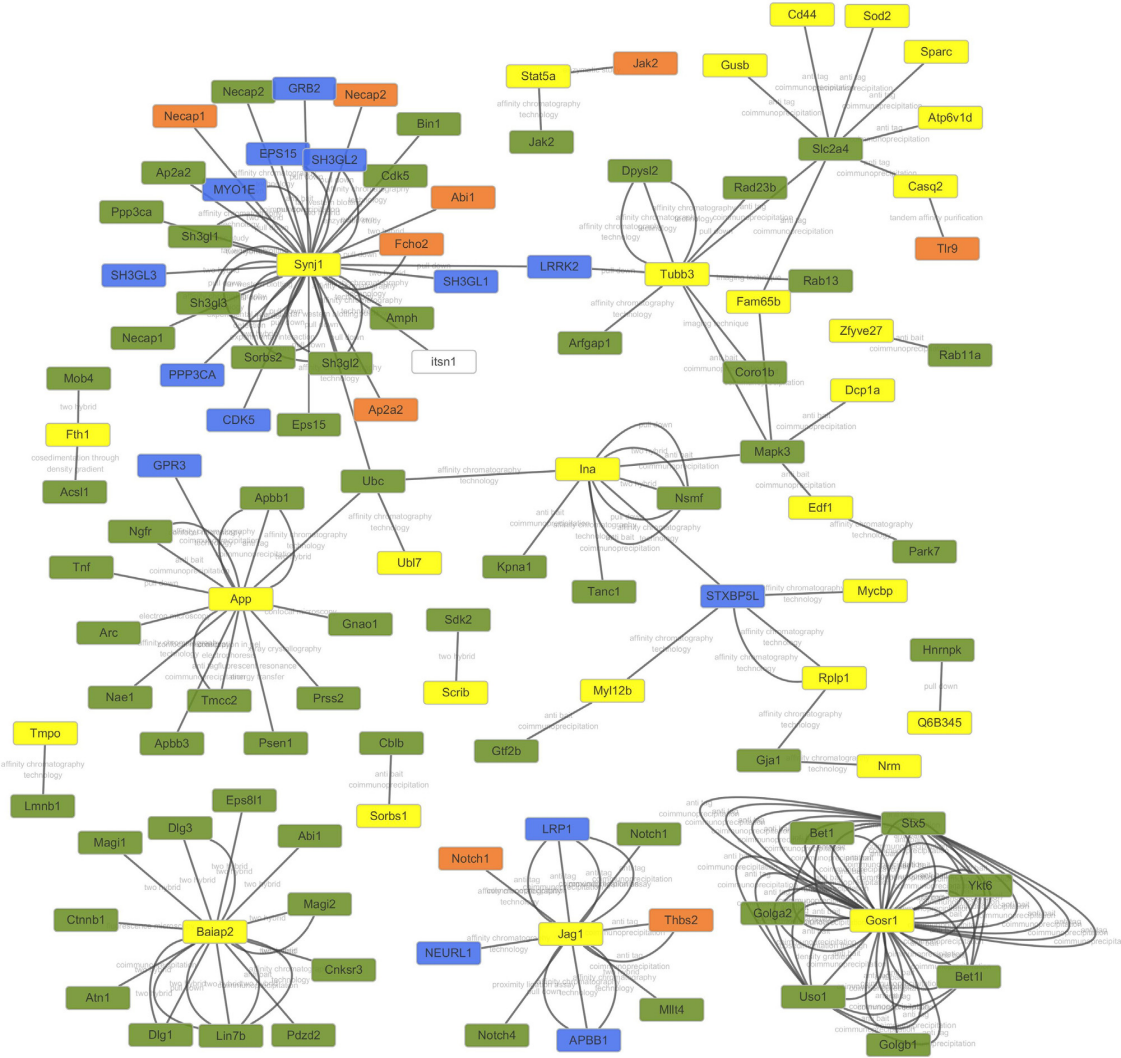
Bioinformatics analysis of citrate-regulated proteins. The citrate-regulated protein interaction network was constructed using the STRING database version 9.1 with default settings except that organism was set to rat. Some anti-oxidant proteins were involved in protein-protein interactions. Superoxide dismutase [Mn] (SOD2), Tubulin beta-3 chain (Tubb3) and Endothelial differentiation-related factor 1 (Edf1) formed highly connected cluster, suggesting that physiological interactions between these proteins might contribute to the anti-oxidant function of citrate. Yellow nodes indicate the differentially expressed proteins identified in the present study. Other nodes indicate interacting proteins.

A citrate-regulated proteins interaction network was generated using the STRING database version 9.1 and revealed that some anti-oxidant proteins were involved in protein-protein interactions (Fig. 9, Table S2). Superoxide dismutase [Mn] (SOD2), Tubulin beta-3 chain (Tubb3) and Endothelial differentiation-related factor 1 (Edf1) formed a highly connected cluster, suggesting that physiological interactions between these proteins might contribute to the anti-oxidant function of citrate. The relative abundance of Tubb3 in cells exposed oxidative stress might exert a protective effect (from UniProt). Notably, other anti-oxidant and anti-inflammatory proteins were not involved in protein-protein interactions, such as Protein Fam120b (PPARγ), Protein Sgsh (SGSH), Platelet-activating factor acetylhydrolase IB subunit gamma (PAFAH1B3), L-xylulose reductase (DCXR), Golt1b protein (GOLT1B), Ras-related protein Rab-27A (Rab27a), and Cytochrome c oxidase subunit 7B (COX7B), suggesting that these two clusters may play an important role in mediating the function of citrate in BMSCs. Interestingly, Edf1 actively regulates PPARγ expression and activity [38], [39], [40]. Therefore, PPARγ might be also involved in protein-protein interactions (Fig. 9).

### 3.7 PPARγ contributes to the anti-oxidative effects of citrate on BMSCs

**Fig. 10:**
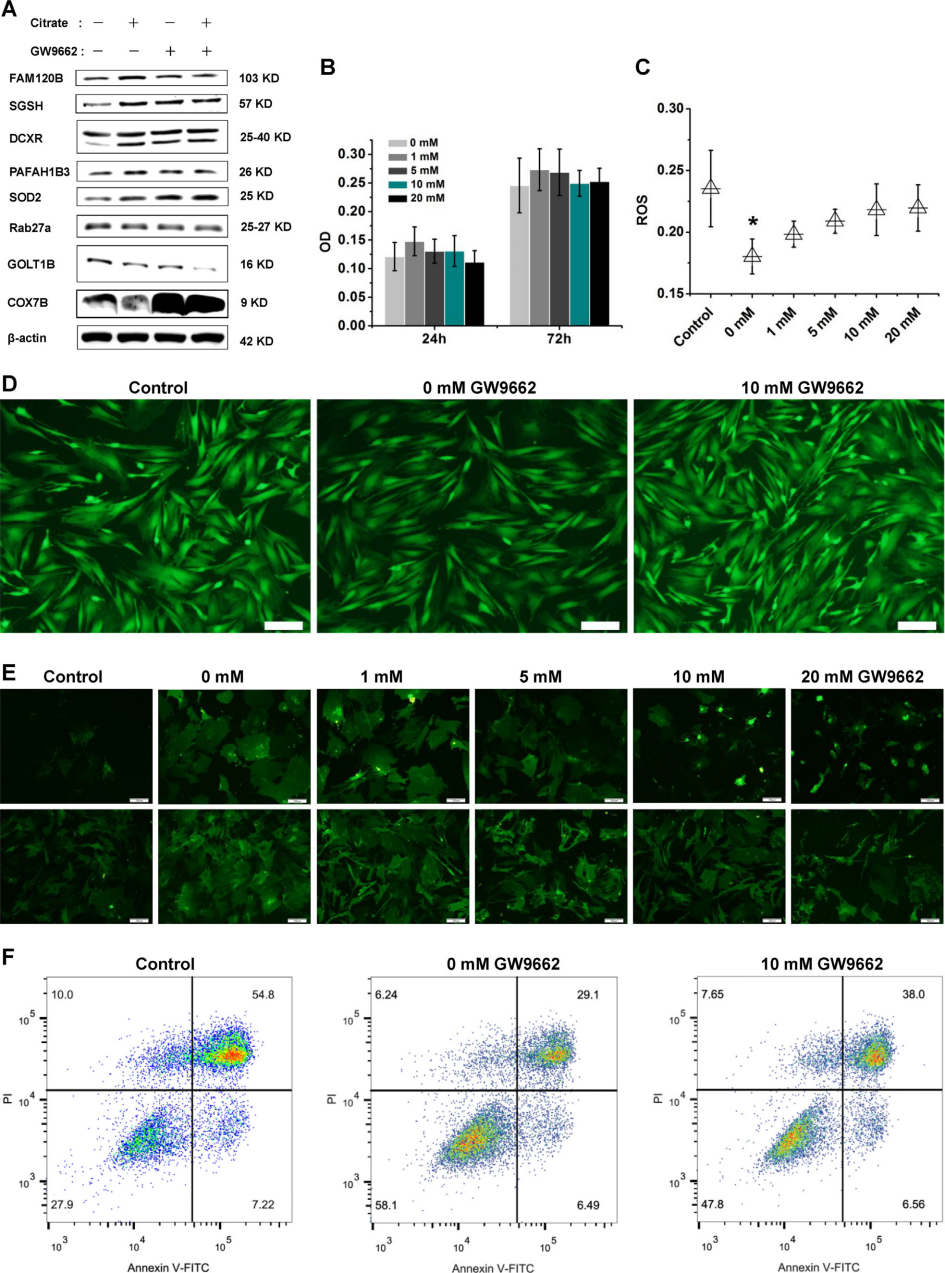
The role of PPARγ in citrate-mediated anti-oxidant functions and proteins expression in BMSCs. (A) Inhibition of PPARγ with 10 mM GW9662 increased the levels of COX7B, inhibited the expression of PAFAH1B3 and DCXR, and showed no effects on the expression of SGSH, SOD2, Rab27a and GOLT1B. (B) The dose of GW9662 that did not alter cell viability. (C and D) PPARγ inhibition resulted in a significant increase in ROS levels. Bar = 100 µm. (E) The cell membrane was damaged following treatment with GW9662 medium containing 1.0 mM citrate. Bar = 100 µm. (F) The percentage of apoptotic cells was examined using flow cytometry, and a significant increase was observed following inhibition of PPARγ. Means ± SD. *p < 0.05, compared with the control.

Bioinformatics analyses suggested that PPARγ might play an important role in mediating the anti-oxidant and anti-inflammatory effects of citrate on BMSCs (Fig. 9 and Table S2). We first examined the levels of the PPARγ, SGSH, PAFAH1B3, DCXR, SOD2, GOLT1B, Rab27a and COX7B proteins in BMSCs treated with 1.0 mM citrate medium for 3 days to determine citrate-regulated protein expression. The results were consistent with those obtained using the quantitative proteomics approach (Fig. 10A). Furthermore, the inhibition of PPARγ with 10 mM GW9662 (the dose that does not alter cell viability (Fig. 10B)) increased the levels of COX7B, inhibited the expression of PAFAH1B3 and DCXR, and showed no effects on the expression of SGSH, SOD2, Rab27a and GOLT1B (Fig. 10A). In addition, we used the PC12 cell line to determine if citrate-regulated PPARγ expression was a global change. A significant increase expression of PPARγ was observed in PC12 cells cultured in 1.0 mM citrate medium and this increase was abrogated by 10 mM GW9662 (Fig. S8). The effects of PPARγ on ROS levels were evaluated in BMSCs treated with 1.0 mM citrate medium for 3 days and subsequently exposed to 200 μM hydrogen peroxide for 1 h. PPARγ inhibition significantly increased ROS levels compared with those of the non-suppressed group (Fig. 10C and D). We next examined the effects of PPARγ on the membranes of BMSCs by staining cells with the fluorescent dye DiO after challenge with 5 mM tert-butyl hydroperoxide or 200 μM hydrogen peroxide (H_2_O_2_). As shown in Fig. 10E, the membranes of stem cells treated with 1, 5, 10 and 20 mM GW9662 medium containing 1.0 mM citrate were damaged, while the stem cells treated with 0 mM GW9662 medium containing 1.0 mM citrate showed rather integrated morphology. We used flow cytometry to examine the percentage of apoptotic cells and determine the effect of PPARγ on BMSC apoptosis. The rate of BMSCs apoptosis was significantly increased following inhibition of PPARγ with 10 mM GW9662 (Fig. 10F). Based on these results, PPARγ plays an important role in mediating the anti-oxidant functions of citrate and citrate-regulated protein expression in BMSCs.

## 4 Discussion

Biomaterials loaded with anti-oxidants with a low relative mass and unstable molecular structure do not achieve long-term sustained release as an oxidative stress-induced disease therapy [21]. A chemically stable anti-oxidant small molecule that can be used as the structure component of the material may provide the benefits of a relatively high anti-oxidant content and continuous local anti-oxidant release. Chemical substances containing small organic molecule citrate, such as calcium citrate, citrate esters, citric acid and citrate anti-coagulants exhibit anti-oxidant and anti-inflammatory properties [21], [22], [23], [24], [25], [26], [27]. In the present study, we systematically evaluated the anti-oxidant capacity of citrate, such as scavenging free radicals and chelating iron ions in chemical assays and modulating redox signaling in cellular assays and animal assays. Using proteomics technology, we further investigated the mechanisms of action by which citrate contributes to redox signaling in BMSCs.

The mechanisms of action of anti-oxidants are the inhibition of oxidant enzymes, direct reactions with ROS/RNS or interactions with redox signaling pathways that translate to a cellular anti-oxidant response [41]. The anti-oxidant mechanism of citrate may include the three pathways mentioned above. Our studies have confirmed that citrate did not show rapid free radical scavenging activity in DPPH and ABTS assays. Lipid peroxidation was not inhibited by citrate, as assessed by the β-carotene bleaching assay. The stable molecular structure ensures that citrate did not directly react with ROS/RNS. Ferrous ions chelated by citrate were not able to participate in the ferrozine reaction, resulting in a lower absorbance. As expected, citrate showed a strong metal iron chelation property. The metal iron plays an important role in the catalytic decomposition of hydrogen peroxide, leading to the formation of reactive hydroxyl radicals, and the effective removal of excess iron is postulated to be an effective method for suppressing free radical-mediated tissue damage [42]. Therefore, the metal iron chelation property of citrate may prevent reaction of free radical under physiological conditions.

Because anti-oxidant actions are not limited to direct reactions with ROS/RNS but includes the regulation of anti-oxidant enzymes, detoxifying enzymes and redox signaling, cellular and animal assays are appropriate models to evaluate the potential anti-oxidant activity of citrate. Citrate exerted positive effects on metabolic activity and membrane morphology of BMSCs under oxidative stress and enhanced the antiapoptotic activity of BMSCs by inhibiting reactive oxygen species production. Citrate also functions as a cellular anti-oxidant by regulating redox signaling, such as decreasing ROS levels and suppressing NF-ĸB activation [21], [22]. This regulation of redox signals in cells provide an explanation for the observed protective effects of citrate on BMSCs under oxidative stress. However, a high concentration of citrate (5.0 mM) reduced BMSCs viability and abolished the protective effects on BMSCs. This finding is consistent with studies showing that high concentrations of citrate (5.0 mM) inhibit on rate-limiting enzymes in glucose metabolism and subsequently inhibit intracellular ATP synthesis and cell proliferation [43]. However, a fundamental question remains regarding the mechanism by citrate from the extracellular milieu enhances cells the cellular anti-oxidant capacity through citrate transporters at the plasma membrane. Some studies have used lithium chloride, an inhibitor of the Na-coupled citrate transporter in the rat liver cell line, to investigate this issue [31]. The protective effects of citrate on BMSCs were negated by the inhibition of the Na-coupled citrate transporter. ROS levels detected by DCFH-DA were also increased compared with those of the non-suppressed group. However, the DCFH-DA assay alone cannot identify what kind of ROS and a change in signal cannot be directly attributed to generation of oxidant. Our ROS detection providing an indication of disturbance of intracellular redox state. Those results suggested that citrate in the extracellular milieu and its transport by Na-coupled citrate transporter, are important factors to regulate intracellular redox state and reduce oxidative damage in BMSCs. Furthermore, an air pouch model was established on the back of the rat for in vivo animal assays. The oxidative stress and inflammatory reactions were induced by LPS. LPS first activates macrophages and neutrophils to up-regulate ROS production and ROS mediated-signaling pathways to generate inflammatory cytokines and chemokines in leukocytes [32], [33], [34], [35], [36]. DHE was used to detect ROS levels in tissues to obtain some oxidation status information. Similar to DCFH-DA assay, DHE is oxidized non-specifically by cellular oxidants. Therefore, a simple fluorescence assay with DHE cannot detect superoxide formation. We further examined the enzyme activity of intracellular MDA, SOD and GSH. Citrate exerted significant inhibitory effects on LPS-induced oxidative stress. The introduction of citrate into the air pouch dramatically decreased the histological parameters of cellular infiltration and reduced IL-1, IL-1β, IL-6 and TNF-α mRNAs in the air pouch. Therefore, citrate exerts significant inhibitory effects on LPS-induced oxidative stress and inflammatory reactions. However, the injection of BMSCs alone did not result in anti-oxidant and anti-inflammatory effects. This redox state inside the air pouch was reversed after citrate was introduced with BMSCs. Therefore, the local administration of stem cells with citrate may be used as a strategy to cure oxidative stress-induced diseases therapy and improve the viability of implantable stem cells under oxidative stress or inflammatory conditions. Despite the availability of different assays, the measurement of ROS in cells and tissues remains very challenging. Oxidant-sensitive fluorescent probes (DCFH-DA and DHE) can provide partial information on cellular redox activity detecting ROS in cells and tissues. Many factors limit their application, such as the interference effect of catalyst, the oxidization of probe by free radicals and antioxidants, the competitive reaction of antioxidant enzymes with the probe, and light-induced oxidation of probe etc [29]. Although citrate did not oxidatively react with probe and the time of light excitation was controlled for a short time, we still cannot quantitatively detect ROS levels by using oxidant-sensitive fluorescent probes for the interference effect of catalyst and oxidization of probe by free radicals.

Based on the regulatory effects of citrate on the oxidative damage and inflammatory factors observed in vitro and in vivo assays, we deduced that citrate may exert its function in BMSCs through multiple protein pathways. An iTRAQ quantitative proteomics assay was performed to globally search for citrate-regulated proteins followed by bioinformatics analyses and functional validation to identify protein pathways regulated by citrate in BMSCs and further reveal the anti-oxidant mechanisms. Bioinformatics analyses suggested that the levels of many proteins related to nucleic acid binding, transcription factors, signaling molecules and enzyme modulators were regulated by citrate. As evidenced by the citrate-regulated protein interaction network, some anti-oxidant proteins, such as Superoxide dismutase [Mn] (SOD2), Tubulin beta-3 chain (Tubb3) and Endothelial differentiation-related factor 1 (Edf1), are involved in protein-protein interactions. SOD2 scavenges mitochondrial ROS and exerts an anti-apoptotic effect on cells exposed to oxidative stress, ionizing radiation and inflammatory cytokines [44], [45]. The relative abundance of Tubb3 in cells exposed to oxidative stress exert a protective effect [46]. Edf1 actively regulates FAM102B (PPARγ) expression and activity, playing an important role in mediating the anti-inflammatory function [38], [39], [40]. PPARγ, an important member of ligand-activated nuclear receptor family, functions as a critical protein involved in metabolism, cellular proliferation, differentiation, and the immune response [47], [48], [49]. Accumulating evidence has revealed an association between the expression of PPARγ and anti-inflammatory activity, as increased PPARγ expression inhibits NF-ĸB and IĸB kinase [50]. According to in vitro studies, the suppression of NF-ĸB activity by citrate in RAW264.7 cells results in reduced anti-inflammatory effects [22]. Inhibition of NF-ĸB expression prevents the induction of pro-oxidant genes [41]. Therefore, PPARγ may also be involved in protein-protein interactions and may play an important role in mediating the anti-oxidant and anti-inflammatory effects of citrate on BMSCs. Notably, other anti-oxidant proteins were not involved in protein-protein interactions, such as Protein Sgsh (SGSH, inhibiting sulfated heparan sulfate-stimulated inflammation [51], [52]), Platelet-activating factor acetyl hydrolase IB subunit gamma (PAFAH1B3, inhibiting Platelet-activating factor-induced inflammation and apoptosis [53]), L-xylulose reductase (DCXR, reducing α-Dicarbonyl compounds to detoxify endogenous and xenobiotic carbonyl compounds [54]), Golt1b protein (GOLT1B, positive regulation of I-ĸB kinase/NF-ĸB signaling [55]), Ras-related protein Rab-27A (Rab27a, positive regulation of reactive oxygen species biosynthetic processes [56]) and Cytochrome c oxidase subunit 7B (COX7B, protein of the mitochondrial apoptotic pathway [57]).

Based on the results from our functional studies, citrate increases the levels of free radical scavenging enzymes (SGSH, DCXR, PAFAH1B3 and SOD2) and decreases the levels of a pro-oxidant protein (Rab27a), pro-inflammatory factors (GOLT1B) and pro-apoptotic protein (COX7B) in BMSCs. The inhibition of PPARγ by GW9662 decreased of DCXR and PAFAH1B3 levels and increased COX7B levels. Therefore, we speculated that PPARγ inhibition may perturb the anti-oxidant function of citrate in BMSCs. The inhibition of PPARγ suppressed levels of ROS production and reduced the protective effects of citrate on BMSCs. Interestingly, the expression of SGSH, Rab27a, GOLT1B and SOD2 was not affected in response to PPARγ inhibition. Thus, the biological function of PPARγ may not be necessary for some anti-oxidant protein pathways, indicating that mechanisms underlying the anti-oxidant effects of citrate include PPARγ dependent and PPARγ independent pathways. We observed PPARγ expression in the PC12 cell line after treatment with 1.0 mM citrate, suggesting that the citrate-mediated increase in PPARγ expression is not cell type specific. However, some phenotypic differences between PPARγ-inhibited and Na-coupled citrate transporter-blocked BMSCs were observed, such as the levels of membrane damage, ROS and apoptosis. Notably, although the focus of the current study is the regulation of some anti-oxidant proteins by citrate, other identified proteins might also be regulated by PPARγ; thus, the network of citrate-regulated proteins requires further study. For instance, Metallothionein-1 (Table S1) protect against metal toxicity, by regulating zinc and copper levels, and protect against oxidative stress. Cysteine residues from Metallothionein-1 capture harmful oxidant radicals, such as superoxide and hydroxyl radicals [58]. Therefore, the potential mechanism of action of citrate may be more complex than presently conceived, and the other proteins listed in Tables S1 and S2 may be as valuable as PPARγ in the network of citrate-regulated proteins.

**Fig. 11:**
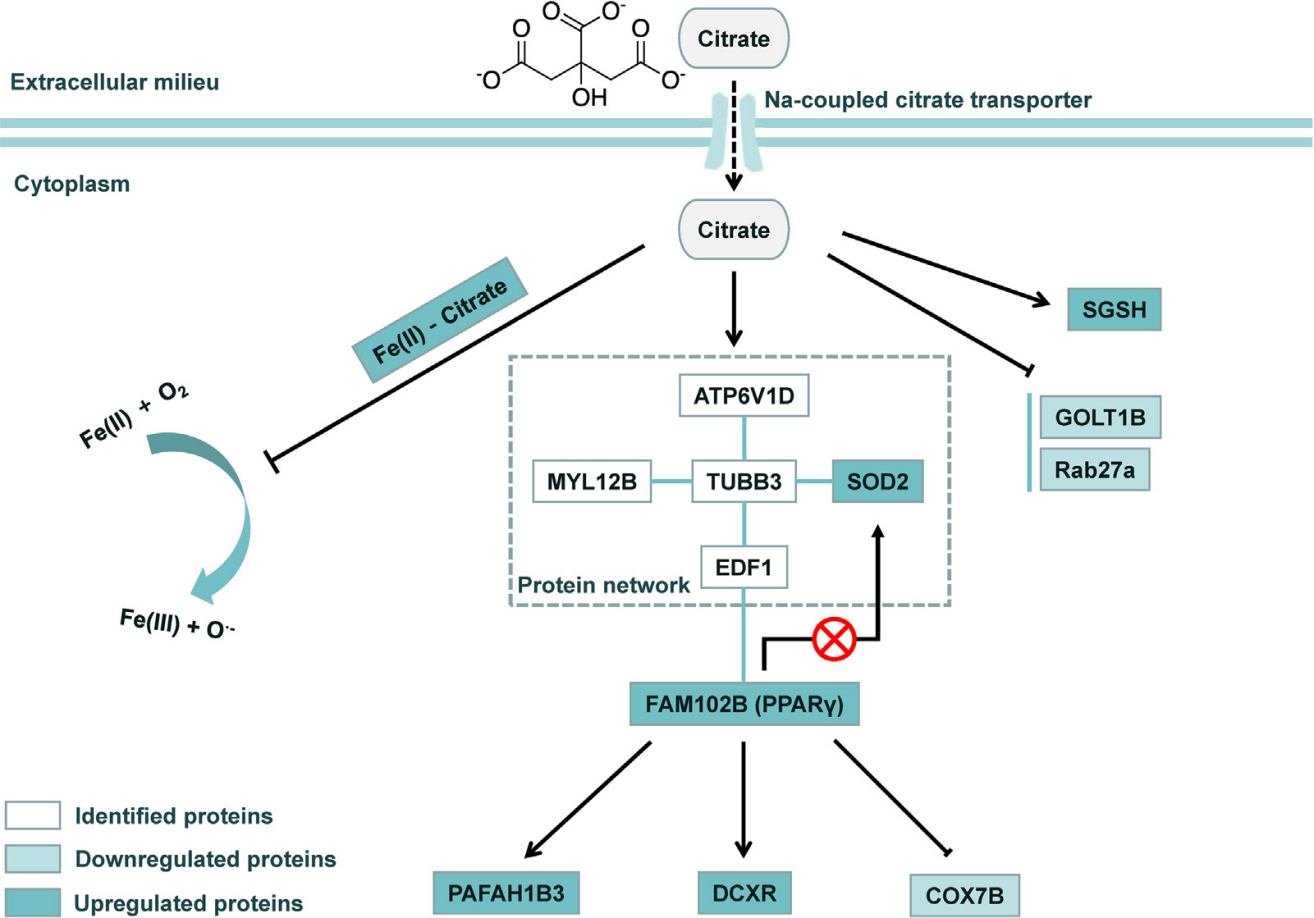
Schematic depicting the proposed molecular mechanism by which citrate regulates cellular redox signaling pathways in BMSCs. Citrate increases the cellular anti-oxidant capacity under oxidative stress conditions via different mechanisms. Citrate may serve as a metal iron chelator to prevent reaction of free radicals under physiological conditions. Citrate may regulate PPARγ expression. As PPARγ is a central organizer of the anti-inflammatory and anti-oxidant responses, inhibition of this protein may disrupt the cellular redox signaling pathways regulated by citrate. Third, citrate directly regulates anti-oxidant proteins via a PPARγ independent pathway. The combination of all these mechanisms regulates the expression of proteins and increases cellular anti-oxidant capacity. PPARγ may be a key molecule in citrate-mediated redox signaling.

Together, we proposed a model depicting the molecular mechanisms by which citrate regulates multiple pro-oxidant and anti-oxidant protein pathways in BMSCs (Fig. 11). Citrate protects BMSCs from oxidative stress-induced damage through at least three pathways. First, as reported in previous studies, citrate may serve as a metal iron chelator to prevent reactions with free radicals under physiological conditions [21], [42]. Second, citrate may regulate PPARγ expression. As PPARγ is a central organizer of the anti-inflammatory and anti-oxidant responses, inhibition of this protein may disrupt the cellular redox signaling pathway regulated by citrate. Third, citrate directly regulates the levels of anti-oxidant proteins via a PPARγ independent pathway.

## 5 Conclusion

In summary, these findings substantially expand the present knowledge concerning the protective effects of citrate on cells or tissues under oxidative stress and provide a new strategy for oxidative stress-induced disease therapy. Furthermore, this study revealed the role of citrate in regulating cellular redox signaling and the function of PPARγ signaling in this process, thereby providing basic molecular cell biology information for improving the applications of biomaterials or stem cells as treatments for oxidative stress-induced degenerative diseases and inflammatory diseases.
