# Supplementary material for: Citrate reduced oxidative damage in stem cells by regulating cellular redox signaling pathways and represent a potential treatment for oxidative stress-induced diseases
Source: Redox Biol. 2018 Nov 22;21:101057. doi: 10.1016/j.redox.2018.11.015 (PMC6302140; doi:10.1016/j.redox.2018.11.015)
Supplement: Supplementary file 1 — Supplementary material [file mmc1.docx]

Supplementary Materials for

**Citrate reduced oxidative damage in stem cells by regulating cellular redox signaling pathways and represent a potential treatment for oxidative stress-induced diseases**

Xiaopei Wu, Honglian Dai, Langlang liu, Chao Xu, Yixia Yin, Jiling Yi, Monika Dorota Bielec, Yingchao Han, Shipu Li

This file includes:

- - Fig. S1. Rat air pouch model.
  - Fig. S2. The objective measurements of changes of inflammatory cells count.
  - Fig. S3. The peptide length distribution of quantified peptides.
  - Table S1. List of anti-oxidant and anti-inflammatory proteins detected in BMSCs.
  - Fig. S4. Functional distribution and subcellular localization of the quantified protein from GO analysis.
  - Fig. S5. PPARγ in KEGG Pathway.
  - Fig. S6. DCXR in KEGG Pathway
  - Fig. S7. SGSH in KEGG Pathway.
  - Table S2. List of proteins in the citrate-regulated protein interaction network.
  - Fig. S8. The role of PPARγ in citrate-mediated proteins expression in PC12 cell line.
  - Table S3. Summary of apoptotic BMSCs for Fig 5C.
  - Table S4. Summary of apoptotic BMSCs for Fig 10F.
  - Fig. S9. The raw data of FAM120B (PPARγ).
  - Fig. S10. The raw data of SGSH.
  - Fig. S11. The raw data of DCXR.
  - Fig. S12. The raw data of PAFAH1B3.
  - Fig. S13. The raw data of SOD2.
  - Fig. S14. The raw data of Rab27a.
  - Fig. S15. The raw data of GOLT1B.
  - Fig. S16. The raw data of COX7B.
  - Fig. S17. The raw data of β-actin.
  - Table S5. List of proteins detected in BMSCs.


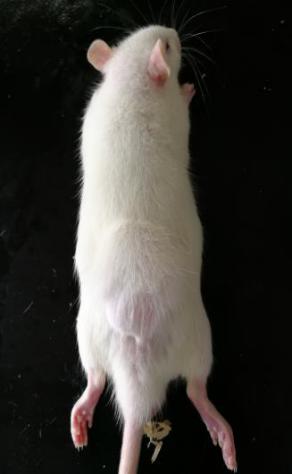

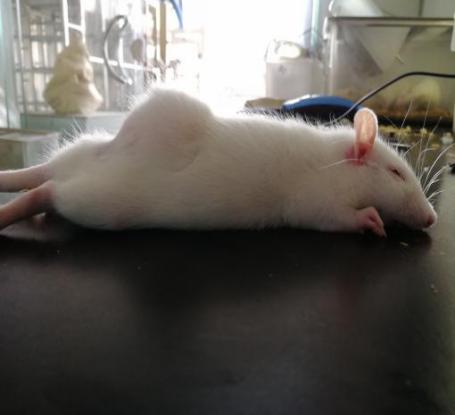

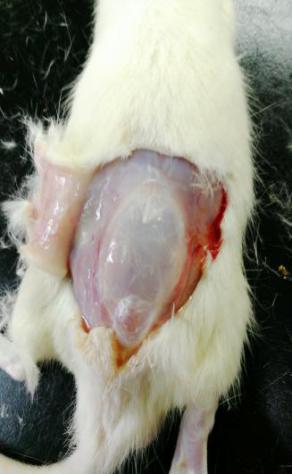

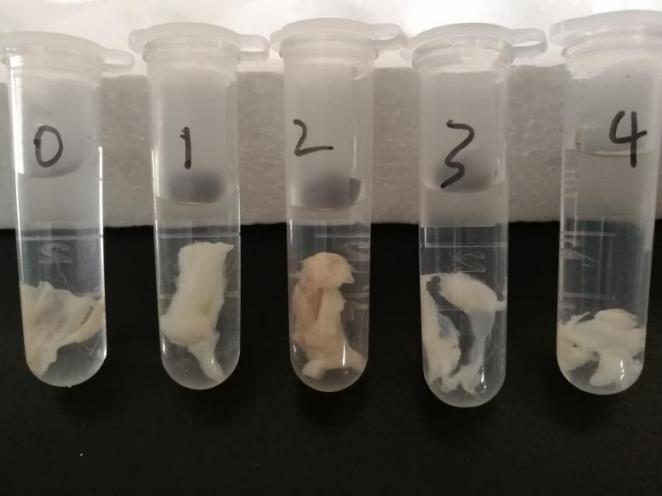


Control

Citrate

Cell

Citrate + Cell

**Fig. S1. Rat air pouch model.** An air pouch model was used to examine inhibitory effects of citrate on the oxidative stress and inflammatory reaction induced by lipopolysaccharide (LPS). The pouches were injected with 2.5 ml of suspension containing control, citrate, stem cells and stem cells/citrate. Control pouches received 2.5 ml of sterile PBS alone.


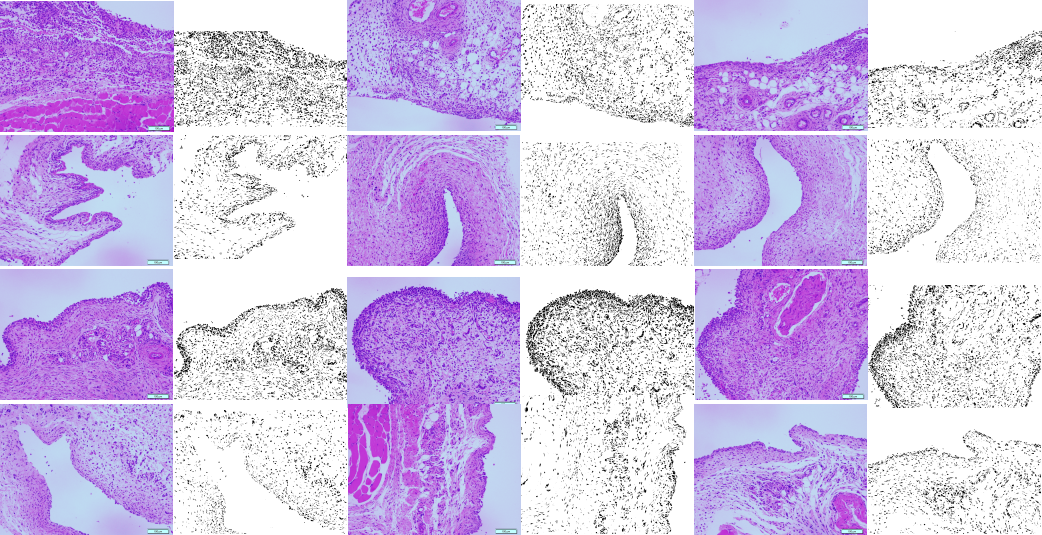


Control

Citrate

Cells

Citrate + Cells

**Fig. S2. The objective measurements of changes of inflammatory cells count.** Inflammatory cells count in response to the different treatment group, determined by image analysis of the histological sections were summarized by Image J. n=3.

**Fig. S3. The peptide length distribution of quantified peptides.**

**Table S1. List of anti-oxidant and anti-inflammatory proteins detected in BMSCs.**

| Protein accession | Protein description | Average |
| --- | --- | --- |
| P02803 | Metallothionein-1 | 2.90 |
| M0R7I5 | Protein Sgsh | 2.07 |
| D4AE88 | Protein Fam120b | 1.30 |
| O35263 | Platelet-activating factor acetylhydrolase IB subunit gamma | 1.29 |
| D4A4U3 | Protein Mdp1 | 1.27 |
| Q5BJY6 | Putative N-acetylglucosamine-6-phosphate deacetylase | 1.27 |
| P69736 | Endothelial differentiation-related factor 1 | 1.27 |
| Q920P0 | L-xylulose reductase | 1.25 |
| Q4QRB4 | Tubulin beta-3 chain | 1.24 |
| P07895 | Superoxide dismutase [Mn], mitochondrial | 1.23 |
| D4AA31 | Protein Prcp | 1.23 |
| Q3MIF4 | Xylulose kinase | 1.21 |
| Q5RJP0 | Aldose reductase-related protein 1 | 1.20 |
| P80431 | Cytochrome c oxidase subunit 7B, mitochondrial | 0.83 |
| P23640 | Ras-related protein Rab-27A | 0.77 |
| P86172 | NmrA-like family domain-containing protein 1 | 0.73 |
| Q76MV3 | Cytochrome C oxidase assembly protein COX17 | 0.71 |
| B0BNB0 | Golt1b protein | 0.67 |


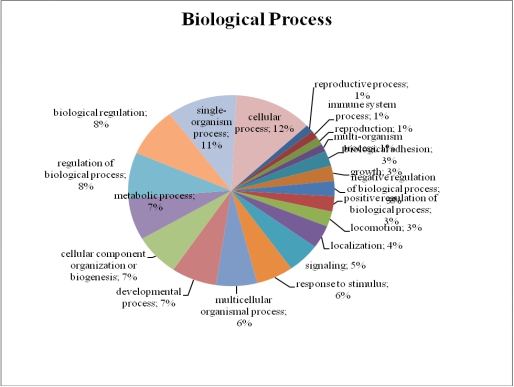

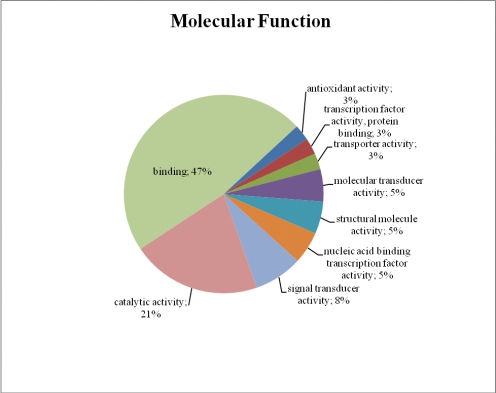

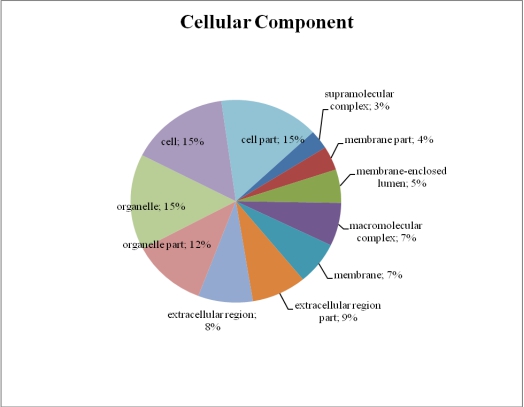

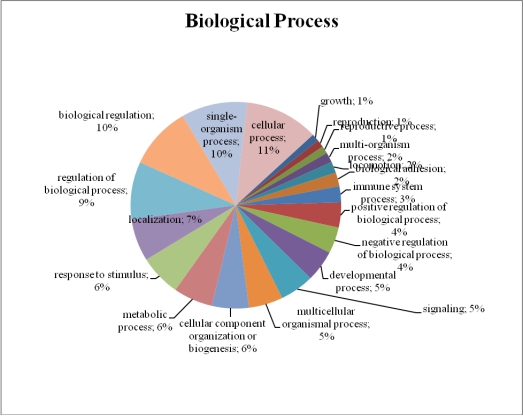


**B**

**D**

**C**

**A**


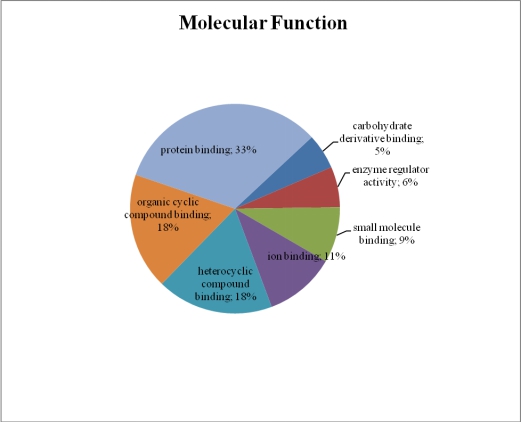

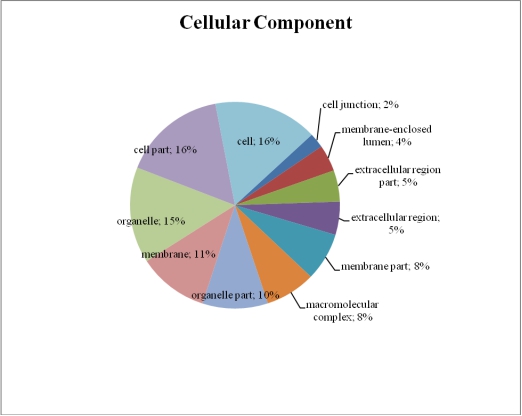


**F**

**E**

**Fig. S4. Functional distribution and subcellular localization of the quantified protein from GO analysis.** (A), (B), (C) represent up-regulated and (D), (E), (F) represent down-regulated.


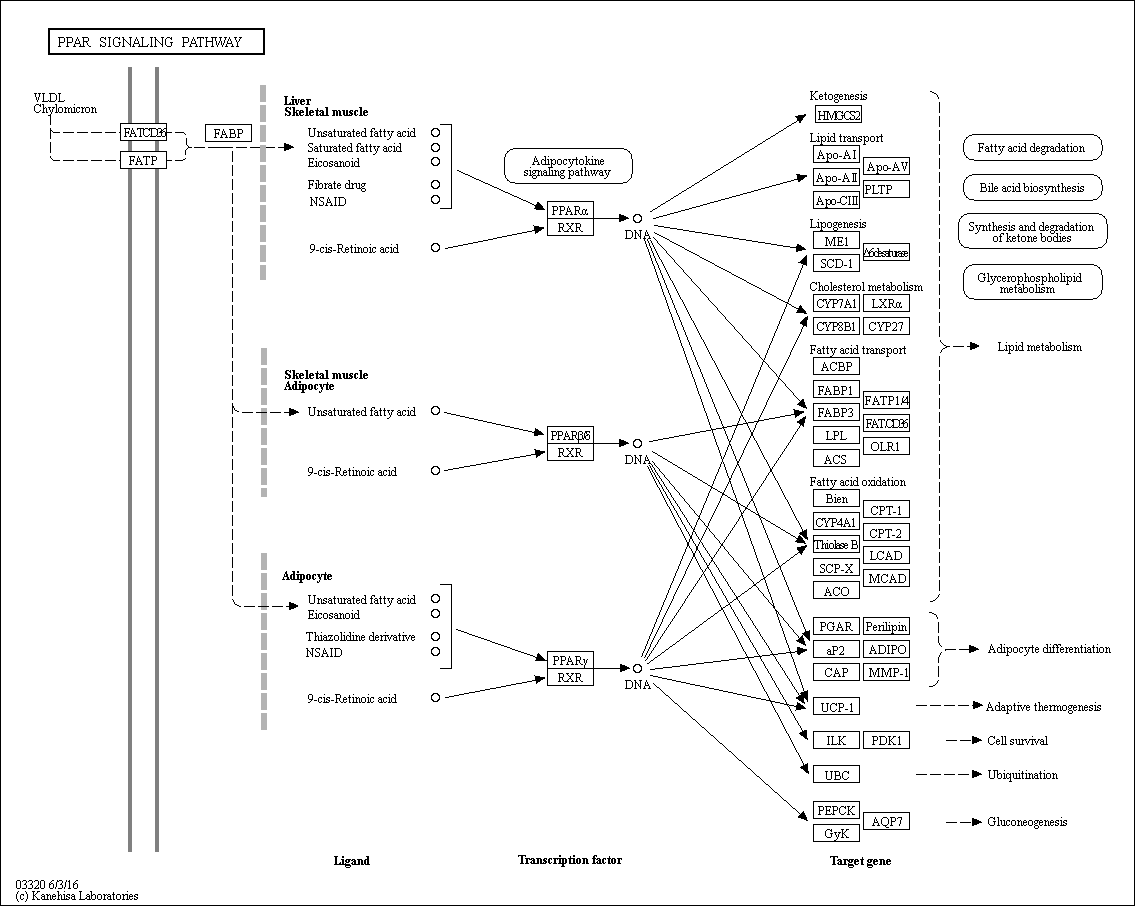


**Fig. S5. PPARγ in KEGG Pathway.** PPARγ have an integrated role in controlling the expression of genes playing key roles in the storage and mobilization of lipids, in glucose metabolism, in morphogenesis and inflammatory response.


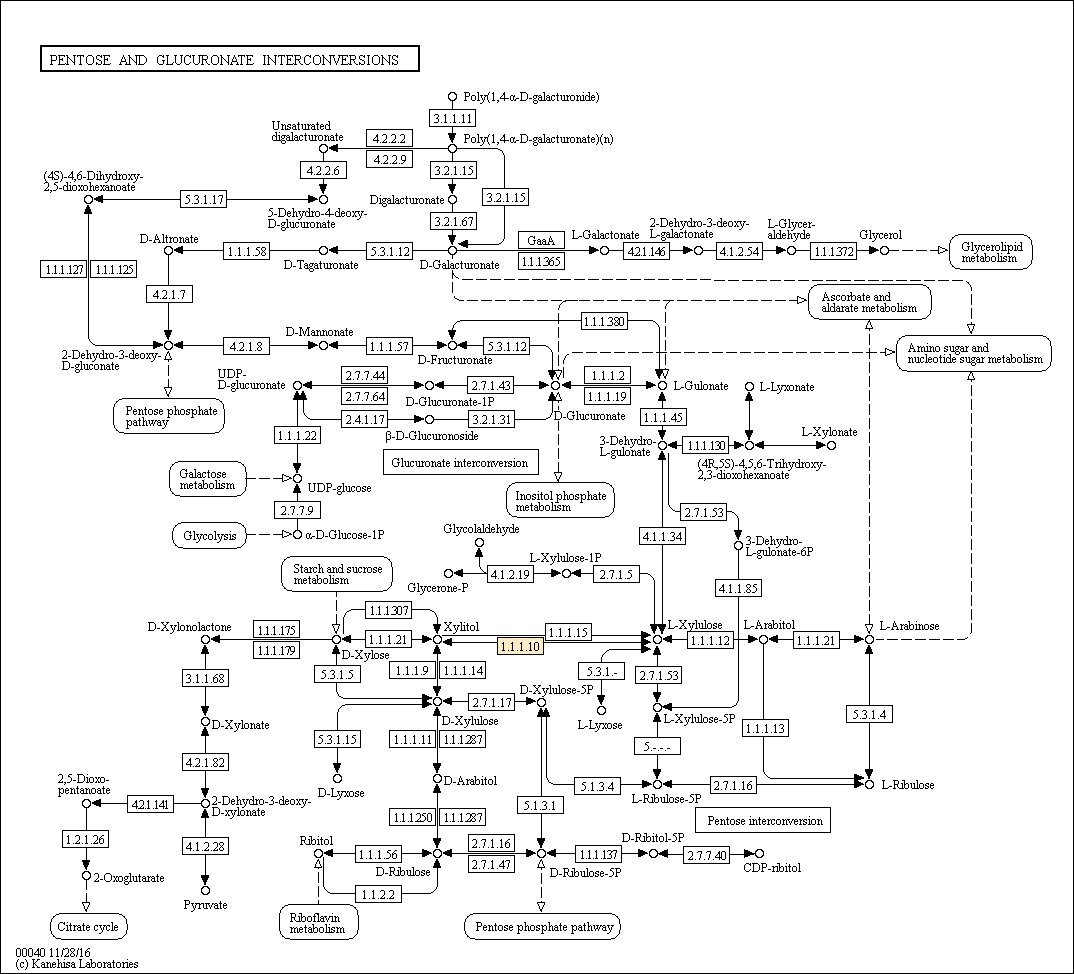


**Fig. S6. DCXR in KEGG Pathway.** DCXR reduces α-Dicarbonyl compounds to detoxify endogenous and xenobiotic carbonyl compounds.


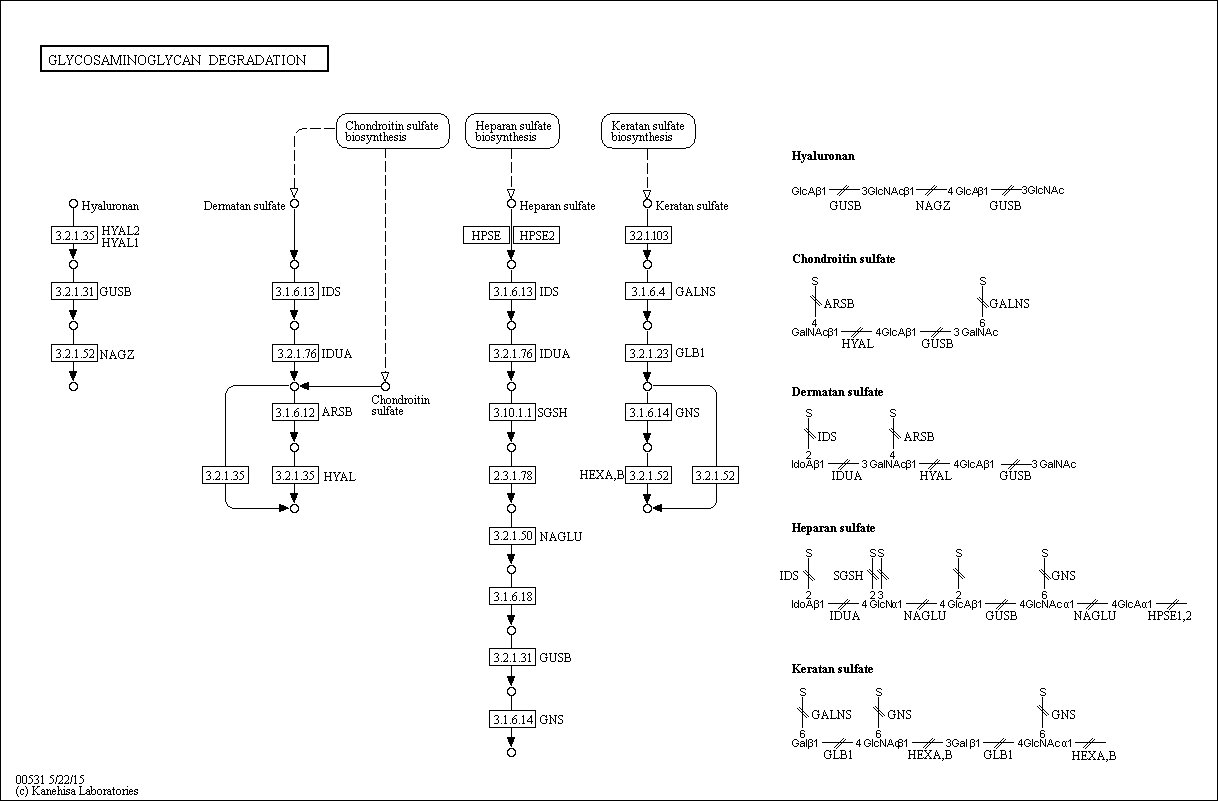


**Fig. S7. SGSH in KEGG Pathway.** SGSH inhibits sulfated heparan sulfate-stimulated inflammation. Deficiency in the enzyme SGSH leads to accumulation of highly sulfated heparan sulfate (HS), resulting in cellular and organ dysfunction, particularly in the brain.

**Table S2. List of proteins in the citrate-regulated protein interaction network.**

| Protein accession | Abbreviation | Protein description | Average |
| --- | --- | --- | --- |
| Q62771 | Stat5a | Signal transducer and activator of transcription 5A | 1.34 |
| P18666 | Myl12b | Myosin regulatory light chain 12B | 1.31 |
| P69736 | Edf1 | Endothelial differentiation-related factor 1 | 1.27 |
| Q4QRB4 | Tubb3 | Tubulin beta-3 chain | 1.24 |
| Q6P503 | Atp6v1d | ATPase, H+ transporting, V1 subunit D, isoform CRA_c | 1.23 |
| D3ZWS0 | Scrib | Protein Scrib | 1.23 |
| P07895 | Sod2 | Superoxide dismutase [Mn], mitochondrial | 1.23 |
| Q6B345 | S100a11 | Protein S100-A11 | 1.20 |
| P19944 | Rplp1 | 60S acidic ribosomal protein P1 | 0.83 |
| P16975 | Sparc | SPARC | 0.83 |
| Q6MG14 | Nrm | Nurim | 0.82 |
| P23565 | Ina | Alpha-internexin | 0.81 |
| Q62733 | Tmpo | Lamina-associated polypeptide 2, isoform beta | 0.80 |
| Q6P7B7 | Zfyve27 | Protrudin | 0.80 |
| P51868 | Casq2 | Calsequestrin-2 | 0.79 |
| P84109 | Sorbs1 | Sorbin and SH3 domain-containing protein 1 | 0.79 |
| D4AE80 | Dcp1a | Protein Dcp1a | 0.78 |
| Q63722 | Jag1 | Protein jagged-1 | 0.76 |
| P06760 | Gusb | Beta-glucuronidase | 0.76 |
| Q62931 | Gosr1 | Golgi SNAP receptor complex member 1 | 0.75 |
| P26051 | Cd44 | CD44 antigen | 0.75 |
| D4A7F2 | Mycbp | Protein Mycbp | 0.73 |
| P08592 | App | Amyloid beta A4 protein | 0.72 |
| Q6GMN2 | Baiap2 | Brain-specific angiogenesis inhibitor 1-associated protein 2 | 0.70 |
| Q6AY24 | Ubl7 | Bone marrow stromal cell-derived ubiquitin-like protein | 0.69 |
| P19132 | Fth1 | Ferritin heavy chain | 0.62 |
| Q7TP54 | Fam65b | Ferritin light chain 1 | 0.59 |
| Q62910 | Synj1 | Synaptojanin-1（Syng1） | 0.45 |


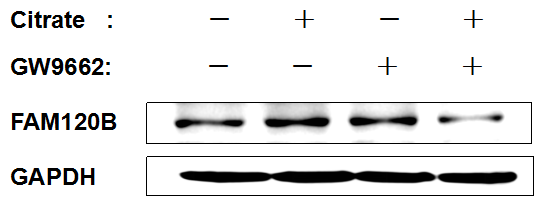


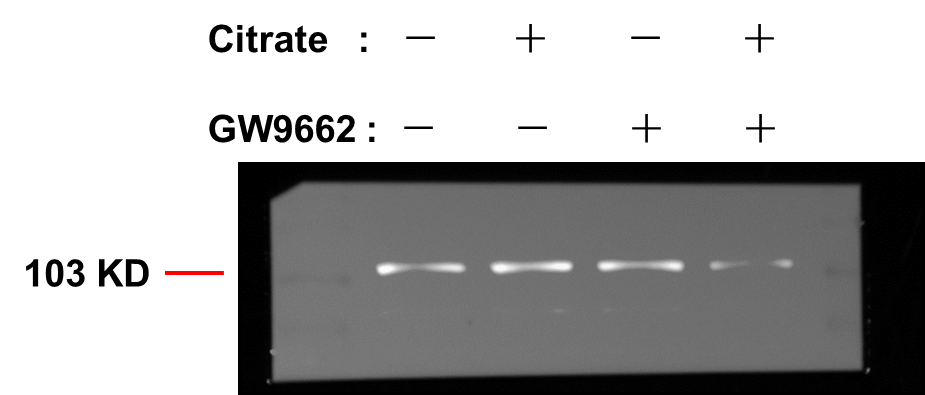


**Fig. S8. The role of PPARγ in citrate-mediated proteins expression in PC12 cell line.** A significant increase expression of PPARγ was observed after PC12 cultured in 1 mM citrate medium and this increase was abrogated by 10 mM GW9662. The results suggested that the citrate-mediated PPARγ increase is not cell-type specific among different cells.

**Table S3. Summary of apoptotic BMSCs for Fig 5C.**

|  | UL | UR | LR | LL |
| --- | --- | --- | --- | --- |
| Control | 3.19 | 55.30 | 13.50 | 27.90 |
| 0 mM LiCl | 2.11 | 28.00 | 11.30 | 58.70 |
| 3 mM LiCl | 2.89 | 46.80 | 13.60 | 36.70 |

**Table S4. Summary of apoptotic BMSCs for Fig 10F.**

|  | UL | UR | LR | LL |
| --- | --- | --- | --- | --- |
| Control | 10.00 | 54.80 | 7.22 | 27.90 |
| 0 mM GW9662 | 6.24 | 29.10 | 6.49 | 58.10 |
| 10 mM GW9662 | 7.65 | 38.00 | 6.56 | 47.80 |


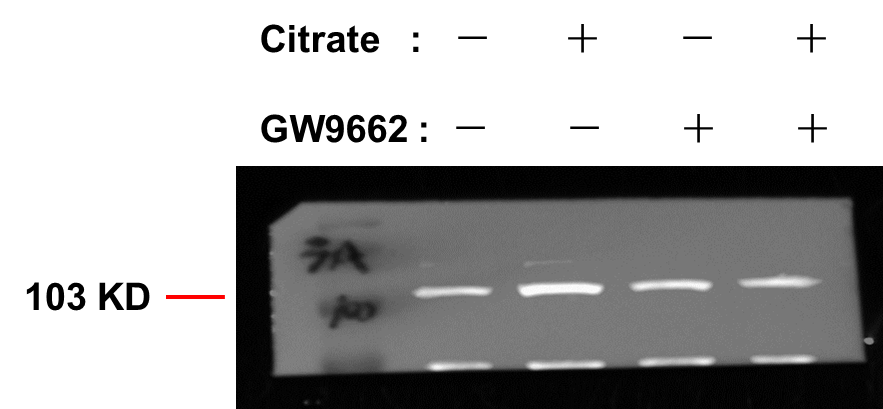


**Fig. S9. The raw data of FAM120B (PPARγ).**


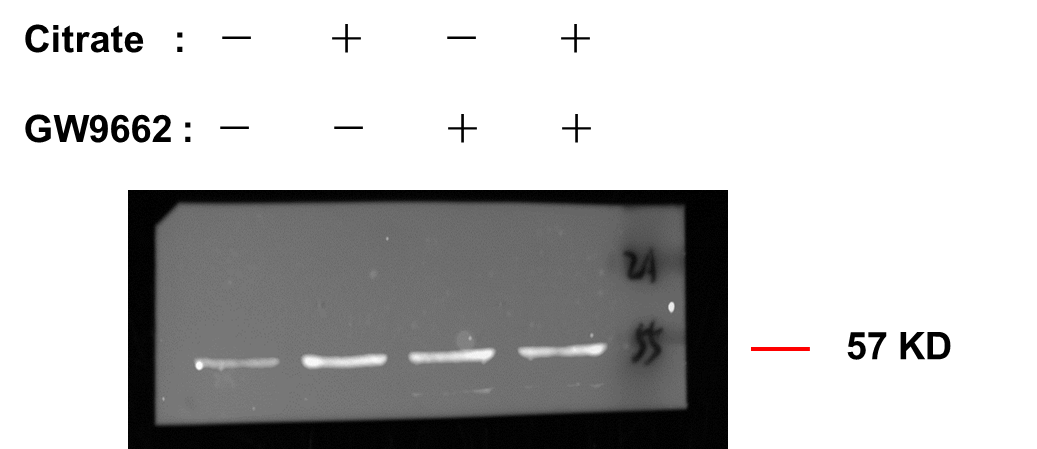


**Fig. S10. The raw data of SGSH.**


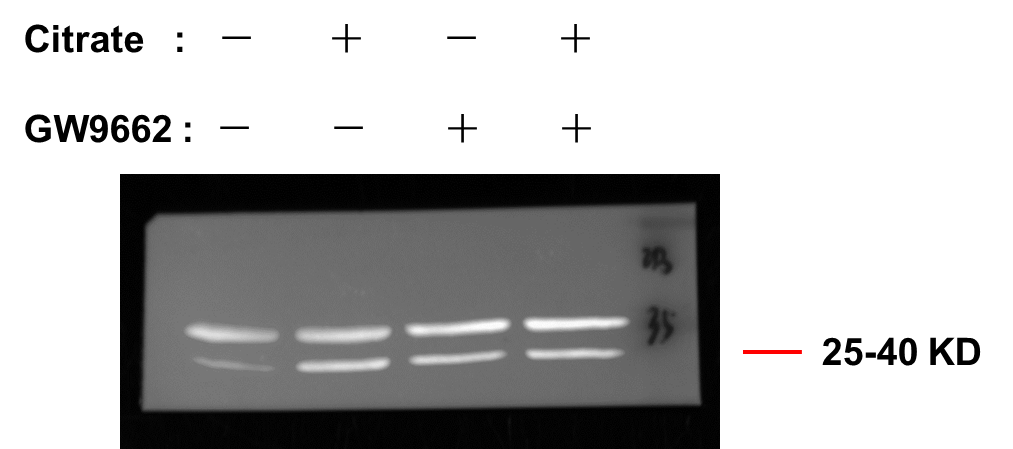


**Fig. S11. The raw data of DCXR.**


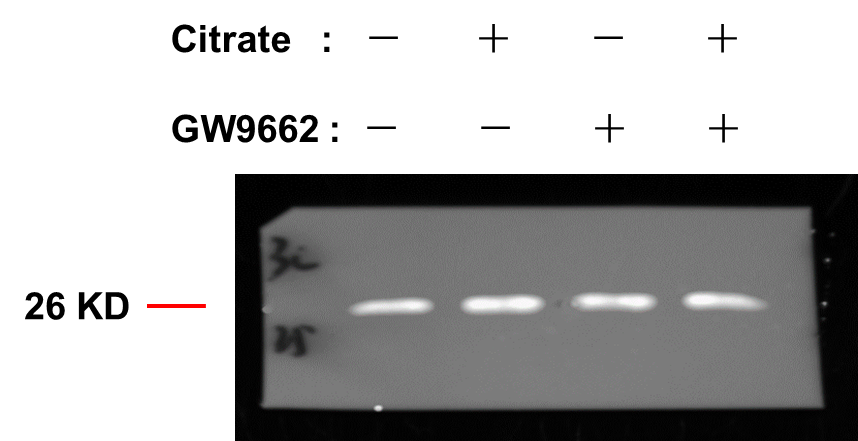


**Fig. S12. The raw data of PAFAH1B3.**


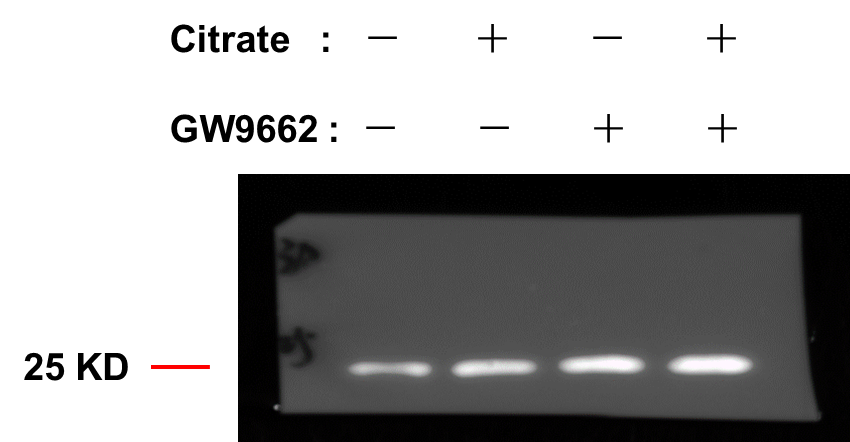


**Fig. S13. The raw data of SOD2.**


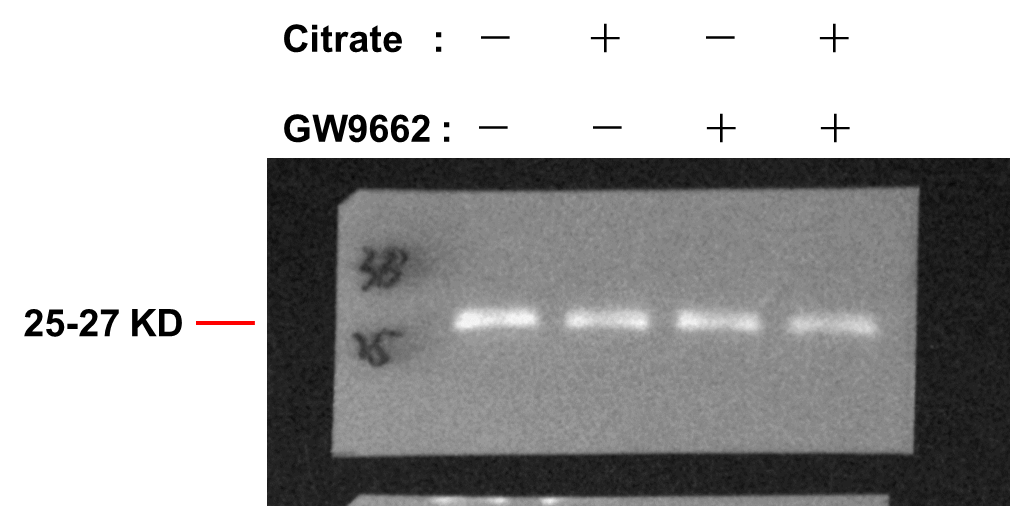


**Fig. S14. The raw data of Rab27a.**


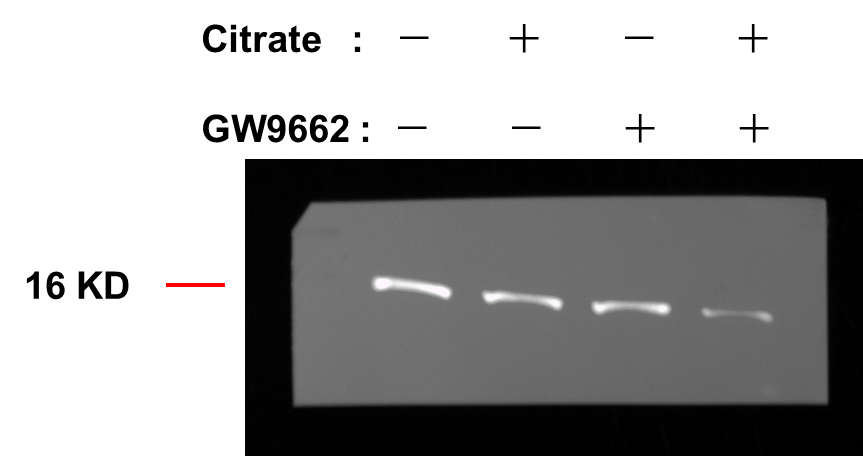


**Fig. S15. The raw data of GOLT1B.**


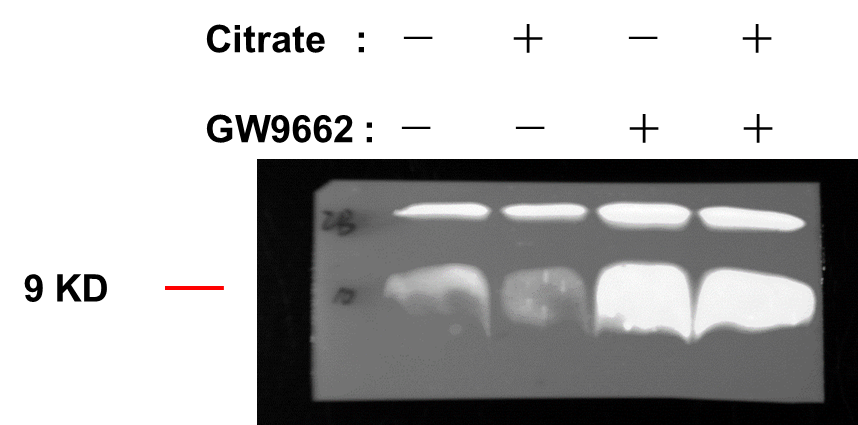


**Fig. S16. The raw data of COX7B.**


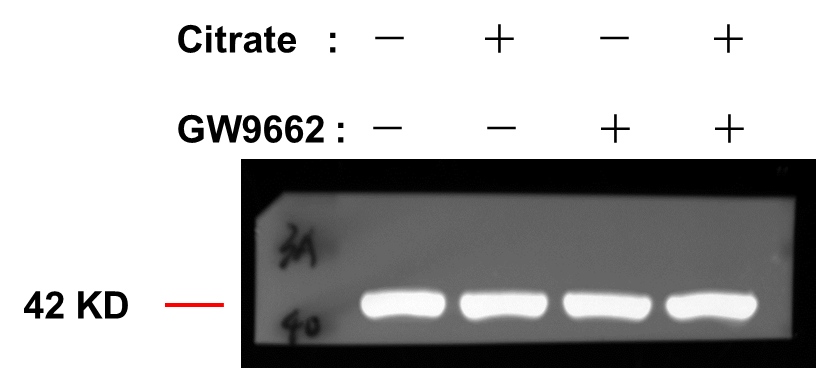


**Fig. S17. The raw data of β-actin.**

**Table S5. List of proteins detected in BMSCs.**

| **Protein accession** | **Protein description** | **MW [kDa]** | **Score** | **Coverage [%]** | **#Peptides** | **#PSMs** | **#Unique peptides** | **Average** | **CV** |
| --- | --- | --- | --- | --- | --- | --- | --- | --- | --- |
| P02803 | Metallothionein-1 | 6.0061 | 2.6485 | 32.8 | 2 | 1 | 1 | 2.90 | 5.8% |
| Q5MPA9 | Serine/threonine-protein kinase DCLK2 | 84.015 | 5.143 | 4 | 3 | 3 | 3 | 2.52 | 2.9% |
| D3ZSU6 | Protein Ankrd11 | 292.34 | 1.9372 | 0.3 | 1 | 1 | 1 | 2.33 | 8.2% |
| M0R7I5 | Protein Sgsh | 35.269 | 9.5646 | 9.5 | 2 | 2 | 2 | 2.07 | 0.9% |
| M0R6T4 | Protein Mms19 | 112.93 | 41.349 | 3.3 | 2 | 2 | 2 | 2.01 | 42.9% |
| D3ZK73 | Cullin 4B (Predicted) | 110.81 | 10.36 | 5.4 | 6 | 7 | 2 | 1.38 | 26.1% |
| Q6MG66 | LSM2 homolog, U6 small nuclear RNA associated [S. cerevisiae] | 14.779 | 11.254 | 14.5 | 1 | 3 | 1 | 1.37 | 5.1% |
| F1LVF7 |  | 43.389 | 2.7625 | 2.3 | 1 | 2 | 1 | 1.36 | 2.1% |
| Q62771 | Signal transducer and activator of transcription 5A | 90.832 | 24.911 | 7.4 | 5 | 5 | 2 | 1.34 | 7.2% |
| A0A0G2JZ01 | Protein Eya3 | 66.569 | 6.4228 | 4.1 | 2 | 2 | 2 | 1.33 | 14.1% |
| A0A096MJZ0 | Protein Dnal1 | 21.503 | 14.756 | 10.5 | 1 | 1 | 1 | 1.31 | 11.1% |
| Q920P6 | Adenosine deaminase | 39.899 | 25.5 | 15.6 | 5 | 6 | 5 | 1.31 | 17.8% |
| F1LRJ1 | Protein Col4a3 | 161.77 | 2.2163 | 1.3 | 2 | 2 | 2 | 1.31 | 10.5% |
| P18666 | Myosin regulatory light chain 12B | 19.838 | 193.58 | 66.3 | 14 | 73 | 1 | 1.31 | 3.3% |
| A0A0G2KAX1 | Protein N6amt2 | 25.532 | 22.036 | 18.3 | 3 | 3 | 3 | 1.31 | 13.7% |
| D3Z981 | Protein Plxna1 | 213.19 | 10.387 | 2.5 | 5 | 5 | 5 | 1.30 | 1.7% |
| D4AE88 | Protein Fam120b | 88.764 | 13.196 | 4.7 | 3 | 4 | 3 | 1.30 | 0.4% |
| D4A6C4 | Protein Rin2 | 101.32 | 3.4822 | 1.9 | 1 | 1 | 1 | 1.30 | 19.0% |
| A0A0G2JZL7 | Protein Nedd9 | 100.29 | 7.3819 | 2.1 | 2 | 2 | 2 | 1.29 | 1.2% |
| M0RA39 | Protein Mxra7 | 19.166 | 4.6647 | 17.3 | 1 | 3 | 1 | 1.29 | 4.6% |
| O35263 | Platelet-activating factor acetylhydrolase IB subunit gamma | 25.863 | 5.476 | 6.9 | 2 | 2 | 2 | 1.29 | 20.1% |
| D4A4U3 | Protein Mdp1 | 18.61 | 31.615 | 31.7 | 4 | 5 | 4 | 1.27 | 16.0% |
| Q5BJY6 | Putative N-acetylglucosamine-6-phosphate deacetylase | 43.538 | 45.251 | 9.8 | 3 | 3 | 3 | 1.27 | 6.3% |
| P69736 | Endothelial differentiation-related factor 1 | 16.369 | 18.289 | 36.5 | 5 | 6 | 5 | 1.27 | 3.2% |
| D4ADV8 | Protein Dvl3 | 78.136 | 4.5209 | 3.9 | 3 | 3 | 3 | 1.26 | 6.3% |
| Q62951 | Dihydropyrimidinase-related protein 4 | 61.085 | 2.0314 | 2.8 | 1 | 1 | 1 | 1.26 | 7.4% |
| D3ZHV7 | Protein Sdsl | 34.953 | 19.76 | 11.2 | 3 | 3 | 3 | 1.26 | 3.5% |
| B2RYW7 | Protein Srp14 | 12.51 | 24.514 | 21.8 | 2 | 4 | 2 | 1.25 | 10.4% |
| A0A0G2JWV4 | Protein Ccdc113 | 44.402 | -2 | 2.1 | 1 | 1 | 1 | 1.25 | 17.9% |
| Q920P0 | L-xylulose reductase | 25.719 | 4.6542 | 7.4 | 2 | 3 | 2 | 1.25 | 2.8% |
| Q5M9F8 | N-terminal kinase-like protein | 89.125 | 18.274 | 4.1 | 3 | 4 | 3 | 1.25 | 5.5% |
| D3ZD72 | Protein Ncapg | 112.55 | 4.6662 | 1.2 | 1 | 1 | 1 | 1.25 | 0.8% |
| A0A0G2JVP9 | RCG63136 | 28.666 | 13.93 | 16.1 | 4 | 7 | 1 | 1.24 | 10.1% |
| Q4QRB4 | Tubulin beta-3 chain | 50.418 | 47.996 | 46.4 | 19 | 98 | 4 | 1.24 | 2.4% |
| Q5BK16 | Protein Trex1 | 33.603 | 7.4042 | 3.5 | 1 | 1 | 1 | 1.24 | 30.1% |
| P67999 | Ribosomal protein S6 kinase beta-1 | 59.131 | 7.2307 | 3.4 | 2 | 2 | 2 | 1.23 | 4.0% |
| Q6P503 | ATPase, H+ transporting, V1 subunit D, isoform CRA_c | 28.309 | 6.6787 | 10.5 | 3 | 3 | 3 | 1.23 | 0.2% |
| D3ZWS0 | Protein Scrib | 179.85 | 85.08 | 5.3 | 6 | 7 | 5 | 1.23 | 0.0% |
| M0RBL8 | Protein Tceal3 | 22.336 | 1.9381 | 4.5 | 1 | 1 | 1 | 1.23 | 11.2% |
| P68182 | cAMP-dependent protein kinase catalytic subunit beta | 40.707 | 34.342 | 24.8 | 9 | 11 | 2 | 1.23 | 6.0% |
| P07895 | Superoxide dismutase [Mn], mitochondrial | 24.674 | 52.503 | 26.6 | 7 | 17 | 7 | 1.23 | 1.8% |
| D4AA31 | Protein Prcp | 55.562 | 15.51 | 8.9 | 3 | 3 | 3 | 1.23 | 15.7% |
| M0RDF1 | Protein Kdm3b | 186.92 | 2.7141 | 0.9 | 2 | 2 | 2 | 1.22 | 6.0% |
| F1M4W7 | Protein Cstf3 | 72.166 | 11.312 | 5.4 | 4 | 4 | 4 | 1.22 | 1.1% |
| P62986 | Ubiquitin-60S ribosomal protein L40 | 14.728 | 5.1559 | 55.5 | 9 | 39 | 1 | 1.22 | 7.7% |
| D3ZQE8 | Protein Xpo5 | 136.88 | 23.864 | 3.4 | 3 | 3 | 3 | 1.22 | 4.8% |
| G3V9M8 | Protein Fam50a | 40.209 | 10.239 | 6.8 | 2 | 2 | 2 | 1.22 | 8.2% |
| Q3MIF4 | Xylulose kinase | 57.969 | 13.109 | 6.5 | 3 | 4 | 3 | 1.21 | 4.1% |
| D4AAM0 | Protein Tnpo3 | 104.18 | 63.967 | 8.5 | 6 | 8 | 6 | 1.21 | 1.7% |
| B0BMY7 | Protein Twf2 | 39.472 | 14.374 | 4.9 | 1 | 2 | 1 | 1.21 | 8.0% |
| Q99MY2 | Diphosphoinositol polyphosphate phosphohydrolase 2 | 20.138 | 9.5489 | 16.8 | 3 | 3 | 2 | 1.20 | 4.4% |
| A0A0G2K2P3 | Protein Tnfaip8 | 22.244 | 3.6039 | 4.7 | 1 | 1 | 1 | 1.20 | 8.6% |
| Q6QI44 | N-alpha-acetyltransferase 25, NatB auxiliary subunit | 111.26 | 7.6249 | 1.3 | 1 | 2 | 1 | 1.20 | 6.2% |
| P63081 | V-type proton ATPase 16 kDa proteolipid subunit | 15.808 | 12.608 | 11.6 | 1 | 3 | 1 | 1.20 | 3.8% |
| F1M2D4 | Protein Arhgap23 | 159.23 | 14.471 | 3.4 | 4 | 5 | 4 | 1.20 | 14.4% |
| D3ZSL2 | Protein Abracl | 9.0303 | 14.967 | 27.2 | 2 | 3 | 2 | 1.20 | 2.6% |
| Q6B345 | Protein S100-A11 | 11.065 | 50.065 | 46.9 | 5 | 29 | 5 | 1.20 | 0.1% |
| Q64361 | Latexin | 25.58 | 119.56 | 40.4 | 7 | 16 | 7 | 1.20 | 1.9% |
| P63255 | Cysteine-rich protein 1 | 8.5497 | 8.9559 | 35.1 | 3 | 5 | 3 | 1.20 | 2.2% |
| Q5RJP0 | Aldose reductase-related protein 1 | 36.121 | 5.1433 | 18.4 | 5 | 11 | 1 | 1.20 | 3.6% |
| D3ZVD8 | Protein Hdac6 | 125.88 | 14.554 | 4.1 | 5 | 5 | 5 | 1.20 | 0.1% |
| Q5RKI1 | Eukaryotic initiation factor 4A-II | 46.402 | 63.825 | 48.2 | 19 | 59 | 8 | 1.19 | 3.3% |
| Q9JHK1 | Caspase | 50.399 | 15.931 | 3.1 | 1 | 1 | 1 | 1.19 | 1.9% |
| A0A0G2JY07 | Protein Mcm5 | 82.467 | 3.5036 | 3 | 2 | 2 | 2 | 1.19 | 3.9% |
| Q3MQ06 | Autophagy protein 5 | 32.398 | 23.481 | 15.6 | 3 | 6 | 3 | 1.19 | 2.3% |
| P62994 | Growth factor receptor-bound protein 2 | 25.206 | 59.63 | 52.5 | 11 | 18 | 11 | 1.19 | 6.1% |
| B2RYP8 | A disintegrin and metalloprotease domain 8 (Predicted), isoform CRA_b | 103.06 | 21.57 | 5.1 | 4 | 6 | 4 | 1.19 | 0.1% |
| P52632 | Signal transducer and activator of transcription 5B | 90.222 | 27.676 | 7.6 | 5 | 6 | 2 | 1.19 | 0.2% |
| Q5XI26 | Signal transducer and activator of transcription | 96.856 | 27.724 | 6.7 | 5 | 6 | 5 | 1.19 | 6.3% |
| Q5PQN1 | Probable E3 ubiquitin-protein ligase HERC4 | 118.54 | 38.836 | 6.1 | 6 | 6 | 6 | 1.19 | 9.7% |
| A0A0G2K6F1 | Protein Tcp11l1 | 56.222 | 19.7 | 5.5 | 3 | 5 | 3 | 1.19 | 0.3% |
| Q9QX69 | LanC-like protein 1 | 45.239 | 17.128 | 16.5 | 6 | 6 | 6 | 1.19 | 2.0% |
| A0A0G2KAI8 | Protein Drg2 | 40.557 | 66.682 | 25 | 9 | 13 | 8 | 1.19 | 2.4% |
| P61149 | Fibroblast growth factor 1 | 17.417 | 14.225 | 25.2 | 4 | 6 | 4 | 1.19 | 2.2% |
| Q5BJS4 | FUN14 domain-containing protein 1 | 17.158 | 8.873 | 11 | 1 | 1 | 1 | 1.19 | 4.6% |
| A0A0G2K435 | Protein Dnajc7 | 65.528 | 17.69 | 9.3 | 6 | 6 | 6 | 1.19 | 4.8% |
| D3ZVU4 | Protein Rbks | 33.974 | 4.5082 | 5.3 | 2 | 3 | 2 | 1.19 | 5.5% |
| D3ZH87 | Protein LOC102547275 | 18.148 | 2.4225 | 14.7 | 3 | 3 | 2 | 1.19 | 0.2% |
| Q5PQN7 | Protein LZIC | 21.394 | 63.754 | 41.1 | 8 | 12 | 8 | 1.19 | 1.1% |
| P17425 | Hydroxymethylglutaryl-CoA synthase, cytoplasmic | 57.433 | 46.578 | 6.9 | 2 | 2 | 2 | 1.19 | 1.4% |
| G3V6C3 | Protein Pbdc1 | 22.423 | 49.672 | 30.3 | 5 | 6 | 5 | 1.19 | 9.6% |
| O88350 | Putative hydrolase RBBP9 | 20.995 | 27.83 | 41.9 | 6 | 6 | 6 | 1.18 | 10.8% |
| Q78P75 | Dynein light chain 2, cytoplasmic | 10.35 | 33.413 | 51.7 | 5 | 12 | 4 | 1.18 | 3.1% |
| M0RDC7 | Protein Mlkl | 54.348 | 26.44 | 16.7 | 7 | 8 | 7 | 1.18 | 1.7% |
| Q6AYG3 | Protein prune homolog | 49.998 | 52.775 | 24.9 | 8 | 10 | 8 | 1.18 | 1.8% |
| P35745 | Acylphosphatase-2 | 10.863 | 15.192 | 29.9 | 3 | 3 | 3 | 1.18 | 11.7% |
| O70593 | Small glutamine-rich tetratricopeptide repeat-containing protein alpha | 34.157 | 49.878 | 23.6 | 6 | 11 | 6 | 1.18 | 1.1% |
| G3V915 | Protein Rnasel | 83.221 | 7.4774 | 3.5 | 3 | 3 | 3 | 1.18 | 1.7% |
| D4AA33 | Lipase | 45.628 | 2.4274 | 3.5 | 1 | 1 | 1 | 1.18 | 17.2% |
| Q6P7Q4 | Lactoylglutathione lyase | 20.819 | 48.8 | 44.6 | 11 | 15 | 11 | 1.18 | 3.5% |
| B0BNE5 | S-formylglutathione hydrolase | 31.363 | 73.012 | 39.7 | 7 | 12 | 7 | 1.18 | 0.5% |
| Q63690 | Apoptosis regulator BAX | 21.35 | 50.292 | 26.6 | 5 | 8 | 5 | 1.18 | 4.1% |
| Q6MG61 | Chloride intracellular channel protein 1 | 26.98 | 222.85 | 74.3 | 14 | 35 | 14 | 1.18 | 2.6% |
| Q80XX4 | Musculoskeletal embryonic nuclear protein 1 | 8.9851 | 22.102 | 31.7 | 2 | 4 | 2 | 1.18 | 0.0% |
| A0A0G2JT65 | Protein Zfp106 | 208.47 | 2.2551 | 1.1 | 2 | 4 | 2 | 1.17 | 10.3% |
| M0RBM9 | Protein Arhgef10l | 139.55 | 30.414 | 6.1 | 6 | 6 | 6 | 1.17 | 9.5% |
| Q5BK81 | Prostaglandin reductase 2 | 38.136 | 45.541 | 21.9 | 6 | 7 | 6 | 1.17 | 8.9% |
| Q00981 | Ubiquitin carboxyl-terminal hydrolase isozyme L1 | 24.838 | 119.02 | 52.9 | 11 | 24 | 11 | 1.17 | 0.1% |
| Q68FS4 | Cytosol aminopeptidase | 56.149 | 256.35 | 54.3 | 22 | 40 | 22 | 1.17 | 0.1% |
| Q566C7 | Diphosphoinositol polyphosphate phosphohydrolase 1 | 19.095 | 16.312 | 17.3 | 3 | 3 | 2 | 1.17 | 4.9% |
| P97697 | Inositol monophosphatase 1 | 30.511 | 80.51 | 38.3 | 10 | 18 | 10 | 1.17 | 4.6% |
| A0A0G2JT64 | Protein Akr1b8 | 35.989 | 161.81 | 43.7 | 11 | 20 | 6 | 1.17 | 0.4% |
| A0A0G2JVP5 | Protein Paip1 | 45.729 | 38.217 | 17.5 | 6 | 9 | 6 | 1.17 | 0.6% |
| D4A0M2 | Nucleoredoxin (Predicted), isoform CRA_a | 41.052 | 80.465 | 37.4 | 9 | 16 | 9 | 1.17 | 0.8% |
| P68511 | 14-3-3 protein eta | 28.211 | 181.59 | 64.6 | 18 | 47 | 14 | 1.17 | 1.8% |
| A4GW50 | Protein Stk38l | 53.758 | 11.658 | 8.4 | 4 | 5 | 2 | 1.17 | 7.2% |
| F1M1L9 | Protein Cpne2 | 61.023 | 23.39 | 6.9 | 3 | 4 | 2 | 1.17 | 6.4% |
| D3ZDZ1 | Amyloid beta (A4) protein-binding, family B, member 1 interacting protein | 73.675 | 6.2259 | 4.7 | 3 | 4 | 2 | 1.17 | 3.0% |
| P70600 | Protein-tyrosine kinase 2-beta | 115.78 | 3.6177 | 1 | 1 | 1 | 1 | 1.17 | 4.3% |
| B5DFN2 | Adenosylhomocysteinase | 53.753 | 66.366 | 21.7 | 10 | 15 | 4 | 1.17 | 3.0% |
| D4A6X4 | Acylphosphatase | 11.312 | 27.003 | 46.5 | 4 | 7 | 4 | 1.17 | 0.1% |
| M0R5N4 | Prefoldin subunit 4 | 15.783 | 38.277 | 20.1 | 3 | 7 | 3 | 1.17 | 0.3% |
| A0A0G2JZH0 | Protein Cab39 | 39.873 | 25.984 | 22.3 | 8 | 8 | 7 | 1.17 | 6.5% |
| M0RD40 | Protein Sik3 | 145.99 | 31.976 | 2.9 | 3 | 3 | 3 | 1.17 | 2.9% |
| Q9QX67 | Death-associated protein 1 | 11.165 | 7.166 | 23.5 | 3 | 5 | 3 | 1.17 | 7.8% |
| Q5U2U2 | Crk-like protein | 33.865 | 48.913 | 31.4 | 8 | 11 | 8 | 1.17 | 3.2% |
| P47942 | Dihydropyrimidinase-related protein 2 | 62.277 | 248.61 | 54.9 | 23 | 74 | 19 | 1.17 | 0.6% |
| P12711 | Alcohol dehydrogenase class-3 | 39.575 | 89.875 | 32.9 | 11 | 18 | 11 | 1.17 | 5.4% |
| P29315 | Ribonuclease inhibitor | 49.974 | 323.31 | 67.3 | 21 | 44 | 21 | 1.17 | 0.5% |
| P0C0A2 | Vacuolar protein-sorting-associated protein 36 | 43.717 | 61.024 | 21.8 | 7 | 10 | 7 | 1.17 | 11.3% |
| P27605 | Hypoxanthine-guanine phosphoribosyltransferase | 24.477 | 82.655 | 51.8 | 10 | 31 | 9 | 1.17 | 0.2% |
| E9PT90 | Protein Spg20 | 85.865 | 47.958 | 12.1 | 7 | 7 | 7 | 1.16 | 0.4% |
| C0KUC6 | LIM and senescent cell antigen-like domains 1 isoform E | 44.382 | 105.55 | 38.5 | 12 | 24 | 8 | 1.16 | 2.5% |
| Q9EQH5 | C-terminal-binding protein 2 | 48.986 | 17.281 | 16.9 | 8 | 11 | 4 | 1.16 | 3.4% |
| F1M4A6 |  | 34.783 | 5.6087 | 5.6 | 3 | 5 | 2 | 1.16 | 2.1% |
| D3ZUQ8 | Protein Szt2 | 377.43 | 2.0213 | 0.3 | 1 | 1 | 1 | 1.16 | 12.9% |
| Q5M819 | Phosphoserine phosphatase | 24.967 | 30.163 | 25.3 | 4 | 10 | 4 | 1.16 | 1.6% |
| O35952 | Hydroxyacylglutathione hydrolase, mitochondrial | 34.109 | 32.68 | 24.9 | 6 | 12 | 6 | 1.16 | 3.2% |
| Q4G009 | Malignant T-cell-amplified sequence 1 | 20.55 | 61.163 | 31.9 | 4 | 5 | 4 | 1.16 | 5.9% |
| Q1RP74 | Protein LOC100911774 | 27.247 | 50.284 | 42.2 | 11 | 16 | 11 | 1.16 | 1.6% |
| Q63768 | Adapter molecule crk | 33.844 | 139.64 | 46.4 | 12 | 20 | 12 | 1.16 | 1.6% |
| A0A0G2JW38 | Protein Rpe | 24.945 | 36.92 | 16.2 | 4 | 5 | 4 | 1.16 | 2.6% |
| A0A0G2QC33 | Protein Atg4b | 44.363 | 44.566 | 13 | 3 | 4 | 3 | 1.16 | 1.9% |
| D3ZSF3 | Protein Gm12728 | 16.475 | 1.8649 | 17.8 | 1 | 6 | 1 | 1.16 | 6.5% |
| P50398 | Rab GDP dissociation inhibitor alpha | 50.536 | 233.42 | 59.1 | 27 | 64 | 20 | 1.16 | 0.8% |
| Q5XXR3 | Rho guanine nucleotide exchange factor 6 | 87.012 | 15.97 | 5.8 | 4 | 5 | 2 | 1.16 | 10.9% |
| Q8CGS4 | Charged multivesicular body protein 3 | 25.062 | 20.262 | 20.2 | 5 | 8 | 5 | 1.16 | 3.1% |
| Q6AZ50 | Ubiquitin-like-conjugating enzyme ATG3 | 35.822 | 45.668 | 24.5 | 8 | 9 | 8 | 1.16 | 2.2% |
| F1LN87 | Protein Mapkapk5 | 54.112 | 2.5024 | 1.7 | 1 | 1 | 1 | 1.16 | 4.7% |
| B0K015 | COMM domain containing 8 | 20.767 | 10.814 | 18 | 3 | 5 | 3 | 1.16 | 0.8% |
| D4A746 | GDP-mannose pyrophosphorylase B (Predicted), isoform CRA_a | 39.902 | 74.167 | 25.6 | 7 | 11 | 7 | 1.16 | 0.4% |
| P53812 | Phosphatidylinositol transfer protein beta isoform | 31.45 | 135.25 | 52 | 12 | 18 | 11 | 1.16 | 3.2% |
| M0RBX6 | Histone H3 | 15.37 | 9.4861 | 25 | 5 | 8 | 5 | 1.16 | 1.9% |
| Q7TNZ6 | STE20-related kinase adapter protein alpha | 43.553 | 19.175 | 5.1 | 1 | 2 | 1 | 1.16 | 0.6% |
| M0R6T1 | Protein Tatdn1 | 33.291 | 20.404 | 18.4 | 5 | 5 | 5 | 1.16 | 1.9% |
| Q66H60 | Coiled-coil domain-containing protein 146 | 114.64 | 3.2141 | 0.9 | 1 | 3 | 1 | 1.16 | 8.1% |
| P07824 | Arginase-1 | 34.973 | 88.843 | 41.2 | 9 | 16 | 9 | 1.16 | 2.3% |
| P61023 | Calcineurin B homologous protein 1 | 22.432 | 70.669 | 52.3 | 9 | 11 | 9 | 1.16 | 14.3% |
| F7FLB2 | Pgm2 protein | 69.057 | 106.58 | 33.2 | 16 | 21 | 16 | 1.15 | 1.8% |
| B2RYS6 | Phosphoribosyl transferase domain containing 1 | 25.557 | 15.911 | 22.2 | 4 | 5 | 3 | 1.15 | 3.0% |
| P61203 | COP9 signalosome complex subunit 2 | 51.596 | 45.659 | 20.5 | 8 | 10 | 8 | 1.15 | 0.2% |
| F1LSP9 | Protein Fggy | 60.253 | 10.097 | 3.6 | 2 | 2 | 2 | 1.15 | 0.7% |
| D3ZKT7 | Protein kish | 8.0598 | 5.0883 | 25 | 2 | 2 | 2 | 1.15 | 3.7% |
| Q5I0D7 | Xaa-Pro dipeptidase | 54.75 | 62.85 | 20.3 | 8 | 14 | 8 | 1.15 | 1.2% |
| P11232 | Thioredoxin | 11.673 | 48.838 | 51.4 | 8 | 24 | 8 | 1.15 | 3.0% |
| P54645 | 5-AMP-activated protein kinase catalytic subunit alpha-1 | 63.973 | 51.76 | 19 | 8 | 10 | 8 | 1.15 | 15.1% |
| O88664 | Serine/threonine-protein kinase TAO1 | 115.95 | 28.307 | 7 | 7 | 7 | 5 | 1.15 | 2.5% |
| P62501 | TSC22 domain family protein 1 | 15.582 | 9.4996 | 18.9 | 3 | 6 | 1 | 1.15 | 3.2% |
| B0BN65 | Family with sequence similarity 49, member A | 37.328 | 36.548 | 19.5 | 5 | 6 | 3 | 1.15 | 1.3% |
| P47858 | ATP-dependent 6-phosphofructokinase, muscle type | 85.559 | 40.953 | 12.9 | 11 | 12 | 8 | 1.15 | 4.3% |
| P14669 | Annexin A3 | 36.363 | 62.449 | 29.9 | 10 | 11 | 10 | 1.15 | 3.6% |
| D3ZCB9 | Protein Fam92b | 33.406 | 2.0694 | 2.4 | 1 | 1 | 1 | 1.15 | 7.0% |
| F1MAF8 | Protein Atg2b | 220.34 | 12.252 | 1.9 | 4 | 4 | 4 | 1.15 | 8.9% |
| P97541 | Heat shock protein beta-6 | 17.505 | 25.936 | 22.8 | 3 | 8 | 3 | 1.15 | 3.8% |
| Q9ES53 | Ubiquitin fusion degradation protein 1 homolog | 34.485 | 52.676 | 25.4 | 6 | 11 | 6 | 1.15 | 3.6% |
| D4A1V7 | MOB kinase activator 1A | 25.091 | 18.446 | 10.6 | 2 | 6 | 2 | 1.15 | 1.1% |
| Q5M965 | Probable tRNA(His) guanylyltransferase | 34.849 | 10.387 | 13.4 | 4 | 4 | 4 | 1.15 | 6.2% |
| O88280 | Slit homolog 3 protein | 167.77 | 14.009 | 2.4 | 3 | 3 | 3 | 1.15 | 1.7% |
| Q5U2X7 | Mitochondrial import inner membrane translocase subunit Tim21 | 27.876 | 11.963 | 8.2 | 2 | 2 | 2 | 1.15 | 8.3% |
| P0DL28 | F-box/WD repeat-containing protein 8 | 67.818 | 3.7308 | 2.9 | 2 | 2 | 2 | 1.15 | 2.7% |
| D4A719 | Protein Snx12 | 19.807 | 75.327 | 49.4 | 10 | 17 | 8 | 1.15 | 1.7% |
| B2RZ27 | Protein Sh3bgrl3 | 10.477 | 41.329 | 47.3 | 5 | 14 | 5 | 1.15 | 0.6% |
| E9PTG8 | Serine/threonine-protein kinase 10 | 111.88 | 12.193 | 5.3 | 5 | 7 | 4 | 1.15 | 7.9% |
| D3ZPE5 | Protein Vps53 | 94.428 | 33.985 | 6 | 4 | 5 | 4 | 1.15 | 2.6% |
| Q52KK3 | Solute carrier family 25 member 51 | 33.774 | 16.731 | 9.1 | 2 | 3 | 2 | 1.15 | 14.5% |
| P05942 | Protein S100-A4 | 11.776 | 26.993 | 57.4 | 8 | 20 | 8 | 1.15 | 0.0% |
| B5DFJ4 | Protein Vps18 | 110.18 | 29.687 | 6.3 | 5 | 6 | 5 | 1.15 | 1.8% |
| Q66HG4 | Aldose 1-epimerase | 37.892 | 21.21 | 16.1 | 5 | 5 | 5 | 1.15 | 1.8% |
| Q1KQ07 | Signal transducer and activator of transcription | 93.868 | 43.849 | 13.6 | 11 | 13 | 11 | 1.15 | 1.6% |
| P06302 | Prothymosin alpha | 12.382 | 39.61 | 35.7 | 4 | 5 | 4 | 1.15 | 6.2% |
| D4A8E7 | Protein Commd6 | 11.991 | 21.33 | 42.6 | 4 | 5 | 4 | 1.15 | 1.6% |
| P30349 | Leukotriene A-4 hydrolase | 69.175 | 152.64 | 36.7 | 19 | 32 | 19 | 1.15 | 1.3% |
| P62138 | Serine/threonine-protein phosphatase PP1-alpha catalytic subunit | 37.512 | 121.58 | 42.4 | 15 | 37 | 4 | 1.15 | 0.1% |
| A0A0G2JYA4 | Serine/threonine-protein phosphatase 2A catalytic subunit alpha isoform | 35.523 | 98.991 | 48.9 | 13 | 25 | 2 | 1.15 | 3.1% |
| P39069 | Adenylate kinase isoenzyme 1 | 21.584 | 102.54 | 59.8 | 12 | 26 | 12 | 1.15 | 2.4% |
| Q9Z1Z3 | Epsin-2 | 62.348 | 26.323 | 12.3 | 4 | 5 | 4 | 1.15 | 10.3% |
| D3ZE72 | Methionine aminopeptidase 1 | 43.205 | 48.422 | 16.8 | 5 | 6 | 5 | 1.14 | 3.2% |
| Q80U96 | Exportin-1 | 123.04 | 179.59 | 20.7 | 19 | 26 | 19 | 1.14 | 4.4% |
| B0BN63 | LOC681996 protein | 38.104 | 82.256 | 36.4 | 9 | 10 | 9 | 1.14 | 4.7% |
| Q5RK25 | Phosphomannomutase | 29.688 | 48.635 | 19.8 | 4 | 5 | 4 | 1.14 | 6.6% |
| P41498 | Low molecular weight phosphotyrosine protein phosphatase | 18.151 | 53.682 | 52.5 | 7 | 9 | 7 | 1.14 | 10.1% |
| Q5BJX0 | N-terminal Xaa-Pro-Lys N-methyltransferase 1 | 25.464 | 17.38 | 9.9 | 2 | 2 | 2 | 1.14 | 2.1% |
| Q5I0F0 | Developmentally regulated GTP binding protein 1 | 40.512 | 71.656 | 39.5 | 14 | 15 | 13 | 1.14 | 1.7% |
| A0A0G2K5T3 | Protein LOC685590 | 123.73 | 3.1166 | 0.8 | 1 | 1 | 1 | 1.14 | 2.1% |
| A0A0G2K7J8 | Bis(5-nucleosyl)-tetraphosphatase [asymmetrical] | 12.22 | 10.818 | 23.4 | 2 | 2 | 2 | 1.14 | 1.1% |
| Q5XI83 | UPF0505 protein C16orf62 homolog | 106.15 | 33.6 | 8.3 | 7 | 10 | 7 | 1.14 | 0.2% |
| Q6IRE4 | Tumor susceptibility gene 101 protein | 44.077 | 28.002 | 14.3 | 5 | 7 | 5 | 1.14 | 2.1% |
| P0C8E4 | Mitogen-activated protein kinase kinase kinase 7 | 67.199 | 12.666 | 5.1 | 2 | 2 | 2 | 1.14 | 4.2% |
| P85968 | 6-phosphogluconate dehydrogenase, decarboxylating | 53.236 | 299.7 | 47 | 24 | 58 | 24 | 1.14 | 0.0% |
| P52759 | Ribonuclease UK114 | 14.303 | 10.838 | 21.2 | 2 | 2 | 2 | 1.14 | 2.1% |
| D4A9N5 | Protein Trim25 | 71.919 | 87.607 | 18 | 10 | 13 | 10 | 1.14 | 3.7% |
| B2RYG8 | Elongator complex protein 6 | 29.459 | 4.6198 | 3.8 | 1 | 1 | 1 | 1.14 | 4.9% |
| P69897 | Tubulin beta-5 chain | 49.67 | 323.31 | 70 | 26 | 170 | 5 | 1.14 | 2.1% |
| Q4V8K5 | BRO1 domain-containing protein BROX | 46.191 | 41.099 | 10.7 | 4 | 10 | 4 | 1.14 | 0.3% |
| P97571 | Calpain-1 catalytic subunit | 82.118 | 104.44 | 22.9 | 12 | 17 | 12 | 1.14 | 1.8% |
| Q5M7T1 | Probable cytosolic iron-sulfur protein assembly protein CIAO1 | 37.617 | 10.847 | 9.1 | 2 | 3 | 2 | 1.14 | 0.8% |
| M0R8C5 | Protein Eri3 | 37.273 | 8.5038 | 3.6 | 1 | 2 | 1 | 1.14 | 3.9% |
| Q68FQ9 | LanC lantibiotic synthetase component C-like 2 (Bacterial) | 50.968 | 17.632 | 10.4 | 3 | 3 | 3 | 1.14 | 8.9% |
| Q04631 | Protein farnesyltransferase/geranylgeranyltransferase type-1 subunit alpha | 44.049 | 12.102 | 9.8 | 4 | 7 | 4 | 1.14 | 5.0% |
| Q6P798 | RCC1 and BTB domain-containing protein 2 | 60.122 | 11.144 | 3.1 | 1 | 1 | 1 | 1.14 | 2.5% |
| Q5XI73 | Rho GDP-dissociation inhibitor 1 | 23.407 | 124.77 | 41.2 | 9 | 39 | 9 | 1.14 | 0.4% |
| Q7TP15 | S-methyl-5-thioadenosine phosphorylase | 43.08 | 88.604 | 27.9 | 8 | 15 | 8 | 1.14 | 0.4% |
| Q6MGD0 | Protein CutA | 18.659 | 28.091 | 15.8 | 3 | 6 | 3 | 1.14 | 5.2% |
| Q3B7U4 | Cytoplasmic tRNA 2-thiolation protein 2 | 57.758 | 6.5873 | 5.1 | 2 | 2 | 2 | 1.14 | 4.7% |
| P48500 | Triosephosphate isomerase | 26.849 | 217.65 | 76.7 | 17 | 49 | 17 | 1.14 | 0.1% |
| P0C5E3 | Palladin | 66.704 | 220.15 | 38.6 | 22 | 54 | 22 | 1.14 | 0.0% |
| P16446 | Phosphatidylinositol transfer protein alpha isoform | 31.907 | 133.06 | 65.3 | 18 | 33 | 17 | 1.14 | 1.1% |
| Q642C0 | DnaJ homolog subfamily C member 8 | 29.812 | 9.3852 | 12.6 | 4 | 4 | 4 | 1.14 | 3.8% |
| Q64620 | Serine/threonine-protein phosphatase 6 catalytic subunit | 35.159 | 10.323 | 16.4 | 5 | 5 | 5 | 1.14 | 1.6% |
| D3ZD23 | ATP-binding cassette, sub-family E (OABP), member 1 | 67.3 | 115.76 | 26.9 | 15 | 30 | 15 | 1.14 | 2.3% |
| Q5FWT7 | Ubiquitin-like domain-containing CTD phosphatase 1 | 36.866 | 7.3055 | 4.4 | 2 | 2 | 2 | 1.14 | 8.3% |
| B0BN18 | Prefoldin subunit 2 | 16.58 | 42.811 | 47.4 | 5 | 10 | 5 | 1.14 | 3.5% |
| Q5BK20 | Hematological and neurological expressed 1-like protein | 20.07 | 18.475 | 26.8 | 4 | 5 | 4 | 1.14 | 4.0% |
| B2GUZ1 | Ubiquitin carboxyl-terminal hydrolase 4 | 108.37 | 41.183 | 10.1 | 8 | 13 | 7 | 1.14 | 5.3% |
| A0A0G2K3Z9 | Peroxiredoxin-1 | 22.164 | 90.735 | 49.7 | 14 | 44 | 13 | 1.14 | 0.1% |
| M0R4P9 | Protein Ube2h | 20.611 | 36.091 | 31.1 | 6 | 11 | 6 | 1.14 | 0.9% |
| Q63767 | Breast cancer anti-estrogen resistance protein 1 | 104.26 | 9.685 | 2.6 | 2 | 2 | 2 | 1.13 | 8.2% |
| D3Z955 | Protein Pgm2l1 | 70.234 | 25.652 | 6.8 | 4 | 5 | 4 | 1.13 | 1.5% |
| P35213 | 14-3-3 protein beta/alpha | 28.054 | 131.83 | 48.4 | 12 | 54 | 6 | 1.13 | 4.0% |
| D4A4E5 | Protein Ccser2 | 93.393 | 6.4088 | 1.3 | 1 | 1 | 1 | 1.13 | 2.2% |
| D3Z8E0 | Ribosomal protein S6 kinase | 83.723 | 165.19 | 35.1 | 21 | 25 | 20 | 1.13 | 0.4% |
| M0RDC8 | Protein Mtrf1 | 52.32 | 2.1364 | 4.3 | 2 | 3 | 2 | 1.13 | 4.4% |
| Q5XIG6 | N-acetylgalactosamine kinase | 50.197 | 87.01 | 26.4 | 10 | 11 | 10 | 1.13 | 2.8% |
| P46844 | Biliverdin reductase A | 33.565 | 91.807 | 48.1 | 13 | 18 | 13 | 1.13 | 3.7% |
| D4ADC3 | Protein Mgst2 | 16.722 | 16.172 | 9.5 | 1 | 2 | 1 | 1.13 | 5.3% |
| B2RZ72 | Actin related protein 2/3 complex, subunit 4 (Predicted), isoform CRA_a | 19.667 | 54.336 | 44.6 | 8 | 14 | 8 | 1.13 | 3.0% |
| Q9JJZ1 | Solute carrier family 2, facilitated glucose transporter member 8 | 51.458 | 4.9013 | 2.5 | 1 | 1 | 1 | 1.13 | 2.9% |
| A0A0G2K1D2 | Protein Rap1gds1 | 57.511 | 205.82 | 49.5 | 21 | 52 | 21 | 1.13 | 1.9% |
| Q07009 | Calpain-2 catalytic subunit | 79.918 | 323.31 | 51 | 28 | 51 | 28 | 1.13 | 0.5% |
| Q04753 | Methylosome subunit pICln | 26.092 | 13.962 | 16.1 | 3 | 4 | 3 | 1.13 | 0.0% |
| Q3MHS7 | GDP-mannose 4, 6-dehydratase | 42.094 | 58.232 | 27.4 | 8 | 10 | 8 | 1.13 | 0.7% |
| A0A0G2JVT8 | Protein LOC500013 | 178.77 | 101.27 | 7.7 | 9 | 11 | 9 | 1.13 | 1.4% |
| F1MAA5 | Protein Rangap1 | 63.142 | 99.951 | 30.9 | 18 | 28 | 18 | 1.13 | 0.4% |
| Q5EBB0 | Protein LOC298795 | 27.916 | 2.998 | 13.3 | 4 | 13 | 1 | 1.13 | 5.2% |
| P97839 | Disks large-associated protein 4 | 108.03 | 40.253 | 9.4 | 7 | 9 | 7 | 1.13 | 3.9% |
| P97578 | Fasciculation and elongation protein zeta-2 | 42.085 | 12.689 | 13.3 | 4 | 4 | 4 | 1.13 | 0.4% |
| Q6AYC4 | Macrophage-capping protein | 38.798 | 186.69 | 55.3 | 13 | 31 | 12 | 1.13 | 0.2% |
| D3ZW57 | 60S ribosomal protein L38 | 7.9604 | 12.902 | 31.9 | 3 | 6 | 3 | 1.13 | 3.2% |
| Q9Z2A6 | Mitogen-activated protein kinase 15 | 60.723 | 1.9971 | 1.5 | 1 | 2 | 1 | 1.13 | 3.6% |
| D3ZXL1 | Protein Arih1 | 64.016 | 23.433 | 12.6 | 6 | 8 | 6 | 1.13 | 4.4% |
| A0A0G2K8N6 | Protein Ankmy2 | 48.341 | 56.131 | 6 | 2 | 2 | 2 | 1.13 | 0.9% |
| P26817 | Beta-adrenergic receptor kinase 1 | 79.784 | 20.884 | 9.4 | 7 | 7 | 7 | 1.13 | 1.5% |
| O08839 | Myc box-dependent-interacting protein 1 | 64.532 | 135.38 | 15.5 | 6 | 9 | 6 | 1.13 | 9.0% |
| Q7TQ20 | DnaJ homolog subfamily C member 2 | 71.768 | 39.213 | 10.5 | 5 | 6 | 5 | 1.13 | 0.4% |
| Q6P756 | Adaptin ear-binding coat-associated protein 2 | 28.404 | 45.516 | 27.4 | 5 | 6 | 5 | 1.13 | 1.2% |
| D3ZSY8 | Protein Tbc1d10b | 86.648 | 31.23 | 12.8 | 6 | 7 | 6 | 1.13 | 10.5% |
| Q923W4 | Hepatoma-derived growth factor-related protein 3 | 22.446 | 21.953 | 19.3 | 3 | 5 | 2 | 1.13 | 5.7% |
| Q6MG60 | N(G),N(G)-dimethylarginine dimethylaminohydrolase 2 | 29.688 | 88.593 | 43.5 | 12 | 16 | 11 | 1.13 | 0.3% |
| D4A3E3 | Protein Arid1a | 217.58 | 38.415 | 1.9 | 2 | 2 | 2 | 1.13 | 5.9% |
| A0A0G2K933 | Protein Eif4e2 | 29.411 | 24.95 | 14.5 | 3 | 4 | 3 | 1.13 | 6.4% |
| O08730 | Glycogenin-1 | 37.378 | 26.857 | 13.2 | 4 | 9 | 4 | 1.13 | 2.4% |
| Q5RKJ1 | Macrophage erythroblast attacher | 45.336 | 41.862 | 12.9 | 4 | 4 | 4 | 1.13 | 2.3% |
| F1MAN8 | Laminin, alpha 5, isoform CRA_a | 403.77 | 6.4233 | 0.5 | 2 | 2 | 2 | 1.13 | 0.6% |
| O88506 | STE20/SPS1-related proline-alanine-rich protein kinase | 60.05 | 21.19 | 12.8 | 7 | 7 | 3 | 1.13 | 11.6% |
| Q7M0E3 | Destrin | 18.533 | 185.4 | 83.6 | 20 | 100 | 19 | 1.13 | 1.5% |
| G3V7W1 | Programmed cell death 6 (Predicted), isoform CRA_a | 21.904 | 91.626 | 46.1 | 8 | 12 | 8 | 1.13 | 0.7% |
| A0A0G2K8D6 | Protein Dcun1d1 | 31.666 | 47.36 | 21.2 | 5 | 7 | 3 | 1.13 | 4.8% |
| P63102 | 14-3-3 protein zeta/delta | 27.771 | 244.97 | 69.4 | 18 | 80 | 13 | 1.13 | 2.5% |
| M0RC59 | Protein LOC100912393 (Fragment) | 41.801 | 6.9595 | 6.6 | 3 | 3 | 3 | 1.13 | 3.8% |
| D3ZRD3 | Phosphodiesterase 6D, cGMP-specific, rod, delta (Predicted), isoform CRA_a | 17.332 | 29.624 | 20.7 | 2 | 3 | 2 | 1.13 | 4.6% |
| D3ZYI0 | Grancalcin (Predicted) | 24.584 | 2.0463 | 3.2 | 1 | 1 | 1 | 1.13 | 4.0% |
| D3Z9G1 | Protein Fbxw17 | 54.326 | 4.1083 | 2.7 | 1 | 1 | 1 | 1.13 | 1.0% |
| G3V8Q1 | Coatomer protein complex, subunit epsilon (Predicted), isoform CRA_c | 34.653 | 143.88 | 55.2 | 12 | 22 | 12 | 1.13 | 3.5% |
| B0BNA5 | Coactosin-like protein | 15.932 | 78.453 | 70.4 | 12 | 22 | 12 | 1.13 | 1.0% |
| Q5M827 | Pirin | 32.178 | 25.386 | 17.5 | 5 | 8 | 5 | 1.13 | 4.8% |
| D3ZJ01 | Protein RGD1307235 | 134.65 | 49.653 | 6 | 6 | 8 | 6 | 1.13 | 0.3% |
| Q6P9U0 | Protein Serpinb6 | 43.018 | 205.05 | 57.5 | 21 | 66 | 19 | 1.13 | 0.7% |
| Q9Z272 | ARF GTPase-activating protein GIT1 | 85.23 | 27.487 | 10.8 | 7 | 7 | 6 | 1.13 | 7.4% |
| D3ZD89 | NMDA receptor-regulated gene 1 (Predicted), isoform CRA_b | 101.01 | 93.982 | 24.6 | 18 | 23 | 18 | 1.13 | 0.6% |
| F7F163 | Protein Nudcd1 | 66.269 | 12.124 | 6 | 3 | 3 | 3 | 1.13 | 1.7% |
| Q8R5M3 | Leucine-rich repeat-containing protein 15 | 64.127 | 21.286 | 8.1 | 4 | 4 | 4 | 1.13 | 4.3% |
| P21139 | Alpha-mannosidase 2C1 | 115.97 | 101.16 | 15.8 | 12 | 13 | 12 | 1.13 | 4.0% |
| Q6AYK6 | Calcyclin-binding protein | 26.541 | 50.667 | 26.6 | 7 | 10 | 7 | 1.12 | 1.3% |
| P63029 | Translationally-controlled tumor protein | 19.462 | 79.564 | 44.8 | 9 | 30 | 9 | 1.12 | 1.1% |
| Q66HA7 | Core-binding factor, beta subunit | 21.517 | 36.052 | 34.1 | 6 | 6 | 6 | 1.12 | 2.9% |
| D3ZLN7 | COMM domain containing 2 (Predicted), isoform CRA_c | 22.781 | 3.0641 | 8 | 2 | 2 | 2 | 1.12 | 2.6% |
| Q62658 | Peptidyl-prolyl cis-trans isomerase FKBP1A | 11.922 | 31.935 | 25 | 2 | 6 | 2 | 1.12 | 4.9% |
| D4A648 | Protein Stk4 | 55.37 | 24.572 | 12.1 | 4 | 5 | 2 | 1.12 | 2.4% |
| P07632 | Superoxide dismutase [Cu-Zn] | 15.911 | 85.828 | 50.6 | 9 | 20 | 9 | 1.12 | 2.2% |
| Q5XIC6 | Proteasome (Prosome, macropain) 26S subunit, non-ATPase, 12 | 52.936 | 153.38 | 48.2 | 21 | 28 | 21 | 1.12 | 0.8% |
| P11030 | Acyl-CoA-binding protein | 10.027 | 58.384 | 62.1 | 5 | 15 | 5 | 1.12 | 0.3% |
| O55096 | Dipeptidyl peptidase 3 | 83.038 | 273.34 | 40.1 | 25 | 50 | 25 | 1.12 | 0.8% |
| O35331 | Pyridoxal kinase | 34.908 | 20.219 | 12.2 | 2 | 2 | 2 | 1.12 | 2.6% |
| D4A255 | Protein Tldc1 | 50.265 | 18.876 | 5.1 | 1 | 1 | 1 | 1.12 | 12.4% |
| Q5U2Z3 | Nucleosome assembly protein 1-like 4 | 43.916 | 61.714 | 28.5 | 10 | 17 | 9 | 1.12 | 0.0% |
| O89046 | Coronin-1B | 53.845 | 69.187 | 24.8 | 11 | 24 | 11 | 1.12 | 5.9% |
| O70196 | Prolyl endopeptidase | 80.741 | 134.55 | 29.2 | 16 | 20 | 16 | 1.12 | 3.4% |
| A1L1K3 | Anaphase-promoting complex subunit 5 | 81.738 | 21.576 | 6.2 | 4 | 5 | 4 | 1.12 | 1.7% |
| M0RC65 | Cofilin 2, muscle (Predicted), isoform CRA_b | 18.709 | 44.96 | 66.3 | 10 | 36 | 7 | 1.12 | 0.5% |
| P81799 | N-acetyl-D-glucosamine kinase | 37.196 | 83.222 | 31.2 | 11 | 16 | 11 | 1.12 | 2.0% |
| A0A0G2JUD2 | Protein Sar1a | 34.527 | 84.856 | 45.4 | 13 | 22 | 13 | 1.12 | 1.6% |
| Q66H79 | Protein Trim32 | 72.153 | 7.281 | 1.7 | 1 | 2 | 1 | 1.12 | 1.0% |
| P85845 | Fascin | 54.49 | 323.31 | 47.5 | 22 | 59 | 22 | 1.12 | 0.0% |
| Q6V9V9 | Protein Stk25 | 48.157 | 9.2974 | 16 | 6 | 8 | 4 | 1.12 | 0.8% |
| Q9JJS5 | SH3 domain-binding protein 4 | 107.51 | 60.861 | 15 | 12 | 12 | 12 | 1.12 | 0.9% |
| Q6AYQ8 | Acylpyruvase FAHD1, mitochondrial | 24.48 | 39.868 | 27.6 | 3 | 4 | 3 | 1.12 | 5.4% |
| G3V7V2 | Angio-associated migratory protein (Predicted) | 46.939 | 54.357 | 16.6 | 5 | 5 | 5 | 1.12 | 3.1% |
| D4ABS5 | Protein Pithd1 | 13.748 | 19.135 | 34.7 | 3 | 3 | 3 | 1.12 | 2.7% |
| P0C644 | Inositol hexakisphosphate and diphosphoinositol-pentakisphosphate kinase 1 | 159.62 | 4.2233 | 1.3 | 2 | 2 | 2 | 1.12 | 8.8% |
| Q5BJP9 | Phytanoyl-CoA dioxygenase domain-containing protein 1 | 32.553 | 45.837 | 27.1 | 5 | 5 | 5 | 1.12 | 3.1% |
| B0BN99 | Hmgb3 protein | 22.985 | 11.177 | 6.5 | 1 | 1 | 1 | 1.12 | 0.9% |
| P14668 | Annexin A5 | 35.744 | 212.09 | 71.5 | 25 | 64 | 24 | 1.12 | 0.3% |
| P53678 | AP-3 complex subunit mu-2 | 46.874 | 2.6445 | 4.3 | 2 | 2 | 1 | 1.12 | 2.2% |
| D3Z916 | Protein Fam188a | 49.502 | 5.9662 | 3.8 | 2 | 2 | 2 | 1.12 | 2.6% |
| P51635 | Alcohol dehydrogenase [NADP(+)] | 36.505 | 138.61 | 70.2 | 20 | 48 | 20 | 1.12 | 1.4% |
| P07943 | Aldose reductase | 35.797 | 148.77 | 58.5 | 19 | 58 | 19 | 1.12 | 1.8% |
| D3ZUQ0 | RILP-like protein 1 | 47.33 | 64.5 | 21.9 | 8 | 9 | 8 | 1.12 | 2.9% |
| Q62952 | Dihydropyrimidinase-related protein 3 | 61.967 | 323.31 | 68.1 | 29 | 137 | 25 | 1.12 | 1.7% |
| D4AEG2 | Protein Rab32 | 25.26 | 46.682 | 27.8 | 5 | 7 | 4 | 1.12 | 0.0% |
| F1M1A6 | Protein LOC681355 | 33.279 | 18.268 | 12.7 | 4 | 5 | 3 | 1.12 | 3.3% |
| F1LUQ0 | Protein Ccdc109b | 39.138 | 42.128 | 14 | 3 | 4 | 3 | 1.12 | 15.4% |
| Q923V4 | F-box only protein 6 | 32.786 | 20.442 | 14.1 | 4 | 7 | 4 | 1.12 | 1.5% |
| Q3ZAU6 | Protein Rnf14 | 54.106 | 13.742 | 9.2 | 4 | 5 | 4 | 1.12 | 0.4% |
| P62828 | GTP-binding nuclear protein Ran | 24.423 | 56.302 | 32.9 | 9 | 17 | 9 | 1.12 | 7.0% |
| P28077 | Proteasome subunit beta type-9 | 23.324 | 7.7941 | 19.2 | 3 | 4 | 3 | 1.12 | 4.9% |
| P62775 | Myotrophin | 12.861 | 63.312 | 44.1 | 7 | 14 | 7 | 1.12 | 2.9% |
| Q6TXG9 | Swi5-dependent recombination DNA repair protein 1 homolog | 28.729 | 17.717 | 9.2 | 2 | 2 | 2 | 1.12 | 0.8% |
| Q9Z0W7 | Chloride intracellular channel protein 4 | 28.633 | 163.81 | 55.7 | 14 | 48 | 14 | 1.12 | 1.1% |
| D3ZWN1 | Protein Zfp259 | 50.854 | 6.1353 | 5.4 | 3 | 4 | 3 | 1.12 | 5.9% |
| P05197 | Elongation factor 2 | 95.283 | 323.31 | 57.3 | 45 | 130 | 44 | 1.12 | 0.6% |
| P48037 | Annexin A6 | 75.753 | 323.31 | 72.7 | 55 | 129 | 53 | 1.12 | 0.2% |
| B2RYJ4 | L-aminoadipate-semialdehyde dehydrogenase-phosphopantetheinyl transferase | 35.816 | 16.071 | 12.9 | 3 | 3 | 3 | 1.12 | 3.4% |
| Q2MCP5 | Protein Wdr45b | 38.02 | 15.531 | 8.7 | 2 | 4 | 2 | 1.12 | 5.8% |
| M0R8P6 | Protein Wwc3 | 128.14 | 7.002 | 1.3 | 1 | 2 | 1 | 1.12 | 6.7% |
| Q765A7 | GPI inositol-deacylase | 104.38 | 10.323 | 2.2 | 2 | 2 | 2 | 1.12 | 4.0% |
| Q9JIL8 | DNA repair protein RAD50 | 153.78 | 24.36 | 5.3 | 6 | 7 | 6 | 1.12 | 3.8% |
| F1LP57 | Protein Map2k4 | 42.397 | 50.604 | 17.2 | 5 | 6 | 5 | 1.12 | 0.9% |
| B0K010 | Protein Txndc17 | 14.092 | 25.771 | 34.1 | 4 | 8 | 4 | 1.12 | 0.3% |
| D4A781 | Protein Ipo5 | 123.7 | 323.31 | 42 | 35 | 73 | 35 | 1.12 | 0.2% |
| D3ZLM5 | NHL repeat containing 2 (Predicted) | 78.561 | 34.529 | 10.3 | 6 | 7 | 6 | 1.12 | 4.0% |
| A0A0G2JSV3 | Ribose-phosphate pyrophosphokinase 1 | 34.806 | 58.271 | 23.6 | 7 | 9 | 3 | 1.12 | 8.3% |
| G3V6L9 | Peptidyl-prolyl cis-trans isomerase | 25.178 | 127.72 | 46 | 10 | 18 | 10 | 1.12 | 1.4% |
| Q9ER24 | Ataxin-10 | 53.726 | 75.078 | 31.2 | 13 | 19 | 13 | 1.12 | 0.0% |
| P21708 | Mitogen-activated protein kinase 3 | 43.08 | 37.269 | 32.9 | 11 | 15 | 8 | 1.12 | 0.4% |
| Q4KM73 | UMP-CMP kinase | 22.169 | 69.127 | 49.5 | 8 | 20 | 8 | 1.12 | 0.8% |
| Q711G3 | Isoamyl acetate-hydrolyzing esterase 1 homolog | 28.004 | 46.304 | 24.5 | 4 | 10 | 4 | 1.12 | 1.3% |
| B0K017 | ADP-ribosylhydrolase like 2 | 39.375 | 6.7533 | 6.2 | 2 | 2 | 2 | 1.12 | 2.5% |
| P84079 | ADP-ribosylation factor 1 | 20.697 | 39.647 | 58.6 | 9 | 38 | 1 | 1.12 | 3.4% |
| Q6AYZ1 | Tubulin alpha-1C chain | 49.937 | 68.706 | 60.8 | 22 | 139 | 2 | 1.12 | 4.3% |
| Q5FVQ9 | Tubulin-specific chaperone E | 59.042 | 14.01 | 10.5 | 6 | 6 | 6 | 1.12 | 2.2% |
| D3ZWA8 | Protein Appl1 | 75.401 | 93.177 | 29.5 | 16 | 18 | 16 | 1.12 | 1.9% |
| O35567 | Bifunctional purine biosynthesis protein PURH | 64.208 | 323.31 | 66.6 | 31 | 52 | 31 | 1.12 | 1.4% |
| A0A0G2K3I9 | MAGUK p55 subfamily member 3 | 23.076 | 75.486 | 36.5 | 6 | 10 | 6 | 1.12 | 1.1% |
| P09117 | Fructose-bisphosphate aldolase C | 39.283 | 5.805 | 16 | 5 | 10 | 1 | 1.12 | 2.1% |
| Q91Y78 | Ubiquitin carboxyl-terminal hydrolase isozyme L3 | 26.123 | 52.907 | 39.6 | 8 | 12 | 8 | 1.12 | 3.3% |
| P07323 | Gamma-enolase | 47.14 | 94.743 | 45.2 | 13 | 42 | 8 | 1.11 | 1.5% |
| M0RD63 | Protein Ranbp3 | 59.147 | 9.4966 | 8.8 | 4 | 6 | 4 | 1.11 | 0.1% |
| M0R781 | Protein Dpp9 | 98.08 | 22.008 | 7.4 | 6 | 8 | 6 | 1.11 | 1.2% |
| Q8R511 | Formin-binding protein 1 | 71.292 | 31.113 | 14.3 | 7 | 8 | 7 | 1.11 | 7.9% |
| D4A769 | Protein Samd4b | 74.911 | 27.831 | 5.8 | 3 | 3 | 3 | 1.11 | 2.6% |
| Q5BK64 | Ribonuclease P protein subunit p40 | 41.639 | 9.3603 | 5.5 | 2 | 3 | 2 | 1.11 | 1.9% |
| F7EZ89 | Protein Tbc1d15 | 78.698 | 30.909 | 12.6 | 8 | 8 | 8 | 1.11 | 1.3% |
| A0A0G2JUZ0 | Protein Mtmr1 | 75.542 | 4.4969 | 2.4 | 2 | 2 | 2 | 1.11 | 3.9% |
| P50399 | Rab GDP dissociation inhibitor beta | 50.537 | 323.31 | 71.9 | 31 | 77 | 24 | 1.11 | 0.3% |
| D3ZAR1 | Protein Ldlrap1 | 33.785 | 39.831 | 13.7 | 3 | 5 | 3 | 1.11 | 5.8% |
| D4AE18 | Protein Zak | 91.765 | 42.353 | 7.1 | 4 | 5 | 4 | 1.11 | 5.3% |
| F1MAA3 | Protein LOC100909464 | 69.183 | 63.809 | 19.3 | 10 | 14 | 9 | 1.11 | 3.1% |
| O88272 | Matrix metalloproteinase-23 | 44.618 | 26.938 | 21.2 | 6 | 6 | 6 | 1.11 | 8.7% |
| P83888 | Tubulin gamma-1 chain | 51.1 | 36.43 | 7.1 | 2 | 2 | 2 | 1.11 | 7.5% |
| P63086 | Mitogen-activated protein kinase 1 | 41.275 | 63.338 | 33.2 | 12 | 19 | 9 | 1.11 | 1.6% |
| O08557 | N(G),N(G)-dimethylarginine dimethylaminohydrolase 1 | 31.426 | 208.84 | 62.1 | 18 | 49 | 17 | 1.11 | 0.3% |
| Q5I0D1 | Glyoxalase domain-containing protein 4 | 33.267 | 103.05 | 51.3 | 14 | 29 | 14 | 1.11 | 1.0% |
| A0JPJ7 | Obg-like ATPase 1 | 44.535 | 58.621 | 26.8 | 9 | 15 | 9 | 1.11 | 1.3% |
| Q9WVR7 | Protein phosphatase 1F | 49.165 | 18.206 | 7.6 | 3 | 5 | 3 | 1.11 | 6.8% |
| D3ZL86 | Protein NEWGENE_1564769 | 241.22 | 17.393 | 3.4 | 7 | 7 | 7 | 1.11 | 7.9% |
| Q6AYR8 | Secernin-2 | 46.501 | 28.802 | 10.2 | 3 | 5 | 3 | 1.11 | 7.6% |
| P36972 | Adenine phosphoribosyltransferase | 19.546 | 188.63 | 83.3 | 12 | 27 | 12 | 1.11 | 0.5% |
| Q5FVC7 | Arf-GAP with coiled-coil, ANK repeat and PH domain-containing protein 2 | 87.229 | 51.967 | 12.9 | 8 | 9 | 8 | 1.11 | 4.1% |
| E9PT65 | Protein Rdx | 68.485 | 198.06 | 44.9 | 29 | 71 | 21 | 1.11 | 2.7% |
| B0K019 | BAG family molecular chaperone regulator 1 | 40.159 | 25.554 | 12.6 | 4 | 4 | 4 | 1.11 | 2.1% |
| B5DF46 | Phosphomannomutase | 27.708 | 41.581 | 36 | 11 | 23 | 11 | 1.11 | 2.5% |
| P81128 | Rho GTPase-activating protein 35 | 172.38 | 29.145 | 6 | 9 | 9 | 9 | 1.11 | 8.0% |
| P85973 | Purine nucleoside phosphorylase | 32.302 | 150.36 | 57.1 | 14 | 43 | 14 | 1.11 | 0.5% |
| P38652 | Phosphoglucomutase-1 | 61.402 | 73.073 | 28.5 | 14 | 26 | 14 | 1.11 | 1.4% |
| P97536 | Cullin-associated NEDD8-dissociated protein 1 | 136.36 | 256.28 | 31.4 | 38 | 64 | 37 | 1.11 | 2.1% |
| E9PTZ3 | Protein RGD1563667 | 41.257 | 1.8703 | 2.8 | 1 | 1 | 1 | 1.11 | 0.7% |
| P49911 | Acidic leucine-rich nuclear phosphoprotein 32 family member A | 28.564 | 52.709 | 27.1 | 8 | 15 | 7 | 1.11 | 4.7% |
| O88658 | Kinesin-like protein KIF1B | 204.17 | 9.2023 | 4 | 6 | 10 | 3 | 1.11 | 10.4% |
| Q9WTT6 | Guanine deaminase | 51.016 | 270.22 | 57.3 | 23 | 47 | 23 | 1.11 | 2.5% |
| P05964 | Protein S100-A6 | 10.035 | 25.946 | 56.2 | 6 | 18 | 6 | 1.11 | 1.4% |
| Q9Z2P5 | Receptor-interacting serine/threonine-protein kinase 3 | 52.217 | 7.1192 | 8.6 | 4 | 4 | 4 | 1.11 | 8.4% |
| P70645 | Bleomycin hydrolase | 52.322 | 121.06 | 35.5 | 14 | 21 | 14 | 1.11 | 0.2% |
| P10688 | 1-phosphatidylinositol 4,5-bisphosphate phosphodiesterase delta-1 | 85.961 | 153.97 | 23.3 | 14 | 20 | 14 | 1.11 | 1.4% |
| P22062 | Protein-L-isoaspartate(D-aspartate) O-methyltransferase | 24.641 | 72.479 | 33.5 | 8 | 11 | 8 | 1.11 | 0.6% |
| D3ZEM8 | Protein Tsr1 | 92.058 | 13.191 | 7 | 5 | 5 | 5 | 1.11 | 4.5% |
| Q02589 | [Protein ADP-ribosylarginine] hydrolase | 39.961 | 12.433 | 11.9 | 4 | 9 | 4 | 1.11 | 2.7% |
| O35346 | Focal adhesion kinase 1 | 119.72 | 27.76 | 7.8 | 7 | 9 | 7 | 1.11 | 0.0% |
| P10111 | Peptidyl-prolyl cis-trans isomerase A | 17.874 | 156.03 | 79.3 | 15 | 76 | 15 | 1.11 | 0.6% |
| P62959 | Histidine triad nucleotide-binding protein 1 | 13.777 | 49.114 | 40.5 | 6 | 9 | 6 | 1.11 | 1.1% |
| P68255 | 14-3-3 protein theta | 27.778 | 162.16 | 66.1 | 17 | 66 | 12 | 1.11 | 0.6% |
| Q3KRD8 | Eukaryotic translation initiation factor 6 | 26.571 | 113.97 | 40.4 | 6 | 10 | 6 | 1.11 | 6.0% |
| P0C1X8 | AP2-associated protein kinase 1 | 103.76 | 47.245 | 7.9 | 7 | 9 | 6 | 1.11 | 1.9% |
| G3V6R9 | Cappuccino homolog (Mouse) | 23.284 | 6.173 | 9.8 | 2 | 2 | 2 | 1.11 | 5.1% |
| Q4KM45 | UPF0687 protein C20orf27 homolog | 19.456 | 8.2057 | 8 | 1 | 1 | 1 | 1.11 | 8.1% |
| Q9EPC6 | Profilin-2 | 15.002 | 62.05 | 44.3 | 7 | 16 | 7 | 1.11 | 3.3% |
| P07335 | Creatine kinase B-type | 42.725 | 222.91 | 60.6 | 17 | 43 | 17 | 1.11 | 2.8% |
| Q5BJP3 | Ubiquitin-fold modifier 1 | 9.1175 | 21.178 | 25.9 | 2 | 4 | 2 | 1.11 | 2.7% |
| Q63798 | Proteasome activator complex subunit 2 | 26.857 | 35.739 | 23.5 | 6 | 14 | 6 | 1.11 | 2.5% |
| Q5U2R0 | Methionine adenosyltransferase 2 subunit beta | 37.374 | 39.259 | 21 | 6 | 6 | 6 | 1.11 | 1.0% |
| Q4FZT2 | Protein phosphatase methylesterase 1 | 42.316 | 86.714 | 38.9 | 12 | 17 | 12 | 1.11 | 1.5% |
| Q7TQ94 | Nitrilase homolog 1 | 32.09 | 94.318 | 46.9 | 11 | 15 | 11 | 1.11 | 2.2% |
| B5DEY8 | Protein Snx6 | 46.636 | 146.03 | 44.1 | 17 | 32 | 17 | 1.11 | 3.0% |
| P83941 | Transcription elongation factor B polypeptide 1 | 12.473 | 61.122 | 54.5 | 5 | 16 | 5 | 1.11 | 5.3% |
| Q8VHT6 | Arsenite methyltransferase | 41.056 | 34.133 | 10.8 | 3 | 4 | 3 | 1.11 | 5.7% |
| Q5EB62 | Solute carrier family 25 member 46 | 46.21 | 7.2273 | 5.7 | 3 | 4 | 3 | 1.11 | 6.5% |
| P62260 | 14-3-3 protein epsilon | 29.174 | 203.56 | 72.9 | 23 | 80 | 20 | 1.11 | 0.8% |
| M0RDU0 | Protein F8a1 | 40.543 | 22.67 | 8.7 | 2 | 2 | 2 | 1.11 | 1.0% |
| P42123 | L-lactate dehydrogenase B chain | 36.612 | 55.223 | 27.8 | 9 | 16 | 8 | 1.11 | 0.9% |
| Q63797 | Proteasome activator complex subunit 1 | 28.577 | 38.87 | 30.9 | 8 | 15 | 5 | 1.11 | 0.0% |
| P18298 | S-adenosylmethionine synthase isoform type-2 | 43.715 | 26.358 | 12.4 | 5 | 6 | 5 | 1.11 | 7.4% |
| D3ZPF0 | Four and a half LIM domains 3 (Predicted) | 31.767 | 46.074 | 30.9 | 8 | 10 | 8 | 1.11 | 1.0% |
| Q5XFX0 | Transgelin-2 | 22.393 | 187.67 | 89.4 | 19 | 91 | 17 | 1.11 | 0.8% |
| Q66H41 | Protein Snx7 | 45.011 | 85.085 | 30 | 12 | 14 | 12 | 1.11 | 0.1% |
| P62716 | Serine/threonine-protein phosphatase 2A catalytic subunit beta isoform | 35.575 | 14.906 | 48.9 | 13 | 24 | 2 | 1.11 | 9.3% |
| D4A3S8 | NOL1/NOP2/Sun domain family, member 2 (Predicted) | 88.09 | 70.615 | 11.6 | 7 | 8 | 7 | 1.11 | 8.4% |
| D3ZFY8 | Protein LOC100362142 | 16.355 | 40.968 | 59.9 | 9 | 13 | 6 | 1.11 | 4.2% |
| O89049 | Thioredoxin reductase 1, cytoplasmic | 54.67 | 206.5 | 42.5 | 17 | 38 | 17 | 1.11 | 0.8% |
| Q68FX1 | Mannose-6-phosphate isomerase | 46.423 | 25.436 | 9.9 | 3 | 6 | 3 | 1.11 | 4.6% |
| D4A6C6 | Protein Tab1 | 54.6 | 12.806 | 2.6 | 1 | 1 | 1 | 1.11 | 6.5% |
| P05370 | Glucose-6-phosphate 1-dehydrogenase | 59.375 | 193.71 | 52.8 | 25 | 56 | 25 | 1.10 | 0.7% |
| Q4FZY0 | EF-hand domain-containing protein D2 | 26.759 | 73.891 | 36.8 | 10 | 20 | 10 | 1.10 | 0.2% |
| Q6P6T4 | Echinoderm microtubule-associated protein-like 2 | 70.71 | 101.65 | 20.2 | 11 | 17 | 11 | 1.10 | 0.0% |
| B1WBV1 | LOC682999 protein | 34.86 | 42.216 | 19.7 | 5 | 5 | 5 | 1.10 | 0.1% |
| Q56R18 | Importin subunit alpha | 57.772 | 59.716 | 21.7 | 10 | 10 | 4 | 1.10 | 0.9% |
| D3ZCS3 | Protein Pcbp4 | 41.452 | 7.3609 | 8.2 | 3 | 4 | 2 | 1.10 | 6.5% |
| D3ZAW4 | Abhydrolase domain containing 4 (Predicted) | 40.368 | 14.702 | 11.5 | 3 | 3 | 3 | 1.10 | 4.9% |
| Q62881 | Nucleolar protein 3 | 24.576 | 63.804 | 13.6 | 3 | 4 | 3 | 1.10 | 2.1% |
| P07150 | Annexin A1 | 38.829 | 323.31 | 77.2 | 32 | 116 | 32 | 1.10 | 0.3% |
| D3ZQG6 | Tripartite motif-containing protein 2 | 81.426 | 44.455 | 5.6 | 3 | 3 | 3 | 1.10 | 0.0% |
| O54975 | Xaa-Pro aminopeptidase 1 | 69.657 | 213.69 | 30.7 | 19 | 36 | 19 | 1.10 | 0.4% |
| Q66H71 | Serine/threonine-protein phosphatase CPPED1 | 35.26 | 7.22 | 6.1 | 2 | 2 | 2 | 1.10 | 0.9% |
| D4AB92 | Protein RGD1560436 | 24.81 | 5.5378 | 7.4 | 1 | 1 | 1 | 1.10 | 2.9% |
| Q9QUK5 | Heat shock protein beta-7 | 9.8038 | 6.5624 | 26.7 | 2 | 3 | 2 | 1.10 | 1.9% |
| D4AAX0 |  | 24.473 | 4.3511 | 4.5 | 1 | 5 | 1 | 1.10 | 1.1% |
| Q5RK27 | Solute carrier family 12 member 7 | 119.38 | 22.544 | 4.2 | 4 | 4 | 4 | 1.10 | 0.4% |
| D3Z8X6 | Protein Dtx3l | 83.102 | 51.997 | 5.7 | 3 | 4 | 3 | 1.10 | 0.3% |
| P47727 | Carbonyl reductase [NADPH] 1 | 30.578 | 121.69 | 53.4 | 11 | 21 | 10 | 1.10 | 0.8% |
| P41562 | Isocitrate dehydrogenase [NADP] cytoplasmic | 46.734 | 117.76 | 43.2 | 17 | 53 | 16 | 1.10 | 1.7% |
| Q64303 | Serine/threonine-protein kinase PAK 2 | 57.96 | 152.24 | 43.5 | 18 | 30 | 11 | 1.10 | 2.1% |
| Q6AXU4 | E3 ubiquitin-protein ligase RNF181 | 19.288 | 13.995 | 22.4 | 3 | 3 | 3 | 1.10 | 0.5% |
| Q9JLJ3 | 4-trimethylaminobutyraldehyde dehydrogenase | 53.652 | 161.02 | 40.5 | 18 | 33 | 18 | 1.10 | 0.0% |
| Q6DGG0 | Peptidyl-prolyl cis-trans isomerase D | 40.765 | 65.673 | 33.8 | 12 | 20 | 11 | 1.10 | 1.8% |
| B2RZB6 | LSM8 homolog, U6 small nuclear RNA associated (S. cerevisiae) | 10.403 | 14.75 | 27.1 | 2 | 4 | 2 | 1.10 | 5.4% |
| D3ZSD8 | Protein Tmem143 | 51.569 | 7.1082 | 5.5 | 2 | 2 | 2 | 1.10 | 15.2% |
| Q64640 | Adenosine kinase | 40.133 | 13.552 | 10 | 4 | 4 | 4 | 1.10 | 3.6% |
| P41499 | Tyrosine-protein phosphatase non-receptor type 11 | 68.458 | 95.829 | 27.1 | 15 | 18 | 15 | 1.10 | 3.4% |
| D4A5S9 | Protein Prpf39 | 77.937 | 3.2629 | 2.9 | 2 | 2 | 2 | 1.10 | 0.8% |
| P70623 | Fatty acid-binding protein, adipocyte | 14.708 | 4.9546 | 9.1 | 1 | 1 | 1 | 1.10 | 1.5% |
| D4ACV3 | Histone H2A | 13.645 | 6.9895 | 21.4 | 5 | 12 | 1 | 1.10 | 2.7% |
| D3ZI16 | COP9 (Constitutive photomorphogenic) homolog, subunit 6 (Arabidopsis thaliana) (Predicted), isoform CRA_a | 37.702 | 95.33 | 32 | 9 | 13 | 9 | 1.10 | 2.5% |
| P20673 | Argininosuccinate lyase | 51.549 | 21.904 | 11.5 | 5 | 6 | 5 | 1.10 | 1.6% |
| Q8VI04 | Isoaspartyl peptidase/L-asparaginase | 34.41 | 20.214 | 17.1 | 5 | 5 | 5 | 1.10 | 1.2% |
| Q4KM49 | Tyrosine--tRNA ligase, cytoplasmic | 59.115 | 128.51 | 52.7 | 28 | 40 | 28 | 1.10 | 2.0% |
| Q62785 | 28 kDa heat- and acid-stable phosphoprotein | 20.605 | 68.61 | 38.7 | 7 | 12 | 7 | 1.10 | 2.0% |
| Q5XI67 | F-box only protein 30 | 82.011 | 12.556 | 4.9 | 3 | 3 | 3 | 1.10 | 4.0% |
| Q9R0C9 | Sigma non-opioid intracellular receptor 1 | 25.27 | 14.936 | 5.8 | 1 | 2 | 1 | 1.10 | 10.4% |
| F1LXA9 | Protein Plekha2 | 47.246 | 47.389 | 18.8 | 7 | 8 | 7 | 1.10 | 5.4% |
| P55053 | Fatty acid-binding protein, epidermal | 15.059 | 137.02 | 54.8 | 11 | 37 | 11 | 1.10 | 3.2% |
| B5DFD8 | SH3 domain-binding glutamic acid-rich-like protein | 12.8 | 101.88 | 72.8 | 8 | 21 | 8 | 1.10 | 0.5% |
| Q99PD4 | Actin-related protein 2/3 complex subunit 1A | 41.599 | 43.843 | 21.4 | 7 | 10 | 7 | 1.10 | 5.4% |
| Q4V7A0 | WD repeat-containing protein 61 | 33.746 | 64.435 | 28.2 | 6 | 9 | 6 | 1.10 | 2.6% |
| Q5M9G7 | Digestive organ expansion factor homolog | 87.824 | 4.1936 | 1.6 | 1 | 1 | 1 | 1.10 | 0.7% |
| G3V6T1 | Coatomer subunit alpha | 138.36 | 322.1 | 41.6 | 46 | 71 | 46 | 1.10 | 1.7% |
| Q3B7D1 | Ubiquitin-conjugating enzyme E2 Z | 38.352 | 26.759 | 18.8 | 6 | 7 | 6 | 1.10 | 5.4% |
| Q6F6B3 | Protein TANC1 | 200.5 | 66.252 | 6.5 | 10 | 11 | 10 | 1.10 | 2.1% |
| P83953 | Importin subunit alpha-5 | 60.136 | 23.46 | 14.3 | 8 | 9 | 6 | 1.10 | 1.7% |
| D3ZF39 | Protein Uap1 | 58.394 | 115.87 | 29.9 | 12 | 17 | 10 | 1.10 | 1.2% |
| A0A0G2K2V5 | Protein Exoc1 | 81.995 | 30.728 | 10.8 | 7 | 9 | 7 | 1.10 | 0.4% |
| Q497B0 | Omega-amidase NIT2 | 30.701 | 114.7 | 58.7 | 14 | 17 | 14 | 1.10 | 0.7% |
| M0R4B8 | Pyruvate kinase | 52.888 | 85.846 | 65.7 | 29 | 88 | 2 | 1.10 | 1.5% |
| Q3T1J1 | Eukaryotic translation initiation factor 5A-1 | 16.832 | 73.897 | 55.2 | 7 | 20 | 7 | 1.10 | 1.9% |
| Q9Z254 | PDZ domain-containing protein GIPC1 | 36.133 | 59.622 | 20.1 | 5 | 8 | 5 | 1.10 | 3.3% |
| Q62920 | PDZ and LIM domain protein 5 | 63.201 | 323.31 | 62.8 | 30 | 97 | 30 | 1.10 | 0.5% |
| A0A0G2K2C7 | Protein Usp9x | 290.22 | 247.79 | 16.9 | 37 | 45 | 37 | 1.10 | 1.5% |
| O35763 | Moesin | 67.738 | 323.31 | 70.2 | 52 | 184 | 39 | 1.10 | 1.0% |
| Q76IC5 | Pyroglutamyl-peptidase 1 | 22.913 | 2.9661 | 3.8 | 1 | 2 | 1 | 1.10 | 0.1% |
| D4A4K4 | Protein Vps13c | 418.62 | 72.178 | 4.5 | 15 | 17 | 15 | 1.10 | 0.8% |
| A0A0G2K1K5 | Protein Trappc11 | 135.04 | 12.154 | 4.4 | 5 | 6 | 5 | 1.10 | 6.6% |
| D3ZLA3 | Copine 3 protein | 59.685 | 52.928 | 16.1 | 8 | 11 | 7 | 1.10 | 6.5% |
| D3ZMX6 | Protein Sntb2 | 56.525 | 29.04 | 10.5 | 5 | 5 | 5 | 1.10 | 0.3% |
| A0A0G2JUX4 | Protein Usp47 | 157.3 | 85.996 | 9.6 | 11 | 13 | 11 | 1.10 | 0.9% |
| O08719 | Ena/VASP-like protein | 42.094 | 10.068 | 3.3 | 1 | 1 | 1 | 1.10 | 10.8% |
| P24155 | Thimet oligopeptidase | 78.385 | 108.28 | 31 | 19 | 25 | 19 | 1.10 | 4.1% |
| P08699 | Galectin-3 | 27.201 | 36.117 | 17.6 | 4 | 5 | 4 | 1.10 | 8.2% |
| P0C0A1 | Vacuolar protein-sorting-associated protein 25 | 20.762 | 9.5727 | 18.2 | 3 | 3 | 3 | 1.10 | 1.5% |
| Q9Z2F5 | C-terminal-binding protein 1 | 46.628 | 55.22 | 17.4 | 7 | 11 | 3 | 1.10 | 4.5% |
| Q02293 | Protein farnesyltransferase subunit beta | 48.673 | 4.3016 | 4.1 | 2 | 2 | 2 | 1.10 | 8.0% |
| B5DF55 | Protein Stam | 59.591 | 84.862 | 23.2 | 11 | 14 | 10 | 1.10 | 2.9% |
| P35465 | Serine/threonine-protein kinase PAK 1 | 60.577 | 66.219 | 32.9 | 14 | 25 | 6 | 1.10 | 0.9% |
| P11762 | Galectin-1 | 14.857 | 97.692 | 74.8 | 11 | 81 | 11 | 1.10 | 0.4% |
| Q4QQV4 | Dead end homolog 1 (Zebrafish) | 57.36 | 196.19 | 50.8 | 24 | 34 | 15 | 1.10 | 0.9% |
| D4ADP2 | Protein Ube2q2l | 42.189 | -2 | 3 | 1 | 1 | 1 | 1.10 | 6.4% |
| Q9Z136 | Hamartin | 129.02 | 14.365 | 2.8 | 3 | 4 | 3 | 1.10 | 6.4% |
| B1WC02 | CTP synthase | 66.64 | 79.676 | 23.5 | 13 | 19 | 13 | 1.10 | 2.4% |
| D3ZQ26 | Protein Rnf25 | 50.828 | 9.5984 | 5.5 | 2 | 2 | 2 | 1.10 | 0.7% |
| O70248 | Amyloid beta A4 precursor protein-binding family A member 3 | 60.881 | 2.9245 | 1.6 | 1 | 1 | 1 | 1.10 | 10.4% |
| Q4QQS8 | Nuclear pore complex protein Nup85 | 74.881 | 63.462 | 8.7 | 4 | 4 | 4 | 1.10 | 4.8% |
| Q08163 | Adenylyl cyclase-associated protein 1 | 51.588 | 209.57 | 63.7 | 27 | 68 | 26 | 1.10 | 0.2% |
| D3ZJR1 | Protein Eps15l1 | 99.486 | 104.28 | 23.4 | 18 | 25 | 18 | 1.10 | 2.2% |
| Q9EQX9 | Ubiquitin-conjugating enzyme E2 N | 17.124 | 60.007 | 53.3 | 7 | 24 | 7 | 1.10 | 2.6% |
| Q69BT7 | Trafficking protein particle complex subunit 4 | 24.359 | 21.582 | 22.8 | 5 | 8 | 5 | 1.10 | 6.4% |
| O35826 | Bifunctional UDP-N-acetylglucosamine 2-epimerase/N-acetylmannosamine kinase | 79.226 | 20.038 | 7.6 | 4 | 5 | 4 | 1.10 | 6.6% |
| Q6RUV5 | Ras-related C3 botulinum toxin substrate 1 | 21.45 | 46.041 | 44.8 | 9 | 22 | 8 | 1.10 | 0.1% |
| B0BNJ1 | LOC683667 protein | 21.624 | 80.42 | 49 | 9 | 17 | 9 | 1.10 | 0.9% |
| O35115 | Four and a half LIM domains protein 2 | 32.086 | 150.36 | 74.2 | 22 | 42 | 22 | 1.10 | 1.0% |
| A0A0G2JTN4 | Protein LOC100912917 | 144.39 | 37.908 | 6.9 | 7 | 9 | 7 | 1.10 | 2.7% |
| G3V985 | Protein Sco1 | 31.768 | 5.7929 | 5.6 | 1 | 1 | 1 | 1.10 | 0.4% |
| D4A4T9 | Cysteine and histidine-rich domain-containing protein 1 | 37.361 | 80.087 | 28.4 | 7 | 11 | 7 | 1.10 | 0.7% |
| Q4V8I9 | Protein Ugp2 | 57.023 | 127.72 | 33.9 | 16 | 26 | 16 | 1.10 | 0.7% |
| Q6AXS4 | Renin receptor | 39.081 | 45.155 | 15.1 | 4 | 5 | 4 | 1.10 | 7.9% |
| P07483 | Fatty acid-binding protein, heart | 14.775 | 34.399 | 53.4 | 7 | 15 | 7 | 1.09 | 2.9% |
| Q7TP48 | Adipocyte plasma membrane-associated protein | 42.062 | 7.626 | 13.3 | 4 | 4 | 4 | 1.09 | 2.8% |
| P10686 | 1-phosphatidylinositol 4,5-bisphosphate phosphodiesterase gamma-1 | 148.55 | 28.673 | 6.4 | 7 | 7 | 7 | 1.09 | 2.2% |
| B2RYM5 | Lys-63-specific deubiquitinase BRCC36 | 33.015 | 8.8152 | 14.1 | 3 | 3 | 3 | 1.09 | 0.1% |
| B5DFK6 | AP-3 complex subunit delta | 135.67 | 173.94 | 19.2 | 18 | 29 | 18 | 1.09 | 4.2% |
| P55260 | Annexin A4 | 35.848 | 169.26 | 59.9 | 21 | 38 | 21 | 1.09 | 1.1% |
| P85971 | 6-phosphogluconolactonase | 27.234 | 178.06 | 67.3 | 10 | 14 | 10 | 1.09 | 4.8% |
| D4A9U6 | Protein Ubr2 | 199.17 | 33.331 | 3 | 3 | 4 | 3 | 1.09 | 2.7% |
| Q66H76 | Paxillin | 64.018 | 26.122 | 12.1 | 6 | 7 | 6 | 1.09 | 2.9% |
| F1LZ05 | Protein Parp14 | 199.53 | 51.725 | 6 | 9 | 11 | 9 | 1.09 | 3.5% |
| D3ZKT8 | HD domain containing 2 (Predicted), isoform CRA_b | 22.988 | 47.627 | 29.1 | 4 | 5 | 4 | 1.09 | 3.9% |
| Q7M767 | Ubiquitin-conjugating enzyme E2 variant 2 | 16.353 | 16.757 | 52.4 | 8 | 13 | 5 | 1.09 | 5.0% |
| Q68FS2 | COP9 signalosome complex subunit 4 | 46.289 | 122.92 | 47 | 14 | 19 | 14 | 1.09 | 0.6% |
| Q9Z339 | Glutathione S-transferase omega-1 | 27.669 | 44.535 | 30.7 | 7 | 12 | 7 | 1.09 | 0.3% |
| Q6AY84 | Secernin-1 | 46.396 | 107.85 | 33.1 | 13 | 23 | 13 | 1.09 | 0.3% |
| Q4QR85 | Methylosome protein 50 | 37.075 | 37.418 | 14 | 4 | 5 | 4 | 1.09 | 5.9% |
| Q9EST6 | Acidic leucine-rich nuclear phosphoprotein 32 family member B | 31.06 | 28.454 | 26.8 | 7 | 12 | 6 | 1.09 | 2.2% |
| P45592 | Cofilin-1 | 18.532 | 148.68 | 81.9 | 19 | 81 | 16 | 1.09 | 0.7% |
| Q5M9G1 | Protein HEXIM1 | 40.317 | 8.1796 | 7.3 | 2 | 2 | 2 | 1.09 | 0.4% |
| Q2YDU3 | OTU domain-containing protein 5 | 60.288 | 2.4213 | 1.8 | 1 | 1 | 1 | 1.09 | 11.4% |
| Q8CF97 | Deubiquitinating protein VCIP135 | 134.56 | 11.088 | 3.3 | 4 | 4 | 4 | 1.09 | 10.1% |
| Q63598 | Plastin-3 | 70.679 | 284.52 | 53.7 | 35 | 112 | 32 | 1.09 | 0.1% |
| D3ZVQ0 | Ubiquitin carboxyl-terminal hydrolase | 95.778 | 145.89 | 26.1 | 19 | 27 | 19 | 1.09 | 0.8% |
| D3ZW47 | Protein Trim16 | 62.985 | 20.936 | 8.1 | 4 | 4 | 4 | 1.09 | 7.2% |
| F1LQM9 | Protein Xpo7 | 124.18 | 25.481 | 3.2 | 3 | 3 | 3 | 1.09 | 3.6% |
| Q9ERE4 | Golgi phosphoprotein 3 | 33.779 | 39.855 | 20.8 | 4 | 5 | 4 | 1.09 | 1.0% |
| P16617 | Phosphoglycerate kinase 1 | 44.538 | 323.31 | 75.8 | 28 | 82 | 28 | 1.09 | 0.5% |
| B5DEN5 | Eukaryotic translation elongation factor 1 beta 2 | 24.675 | 77.654 | 48.9 | 10 | 30 | 10 | 1.09 | 1.1% |
| Q80WK7 | Equilibrative nucleoside transporter 3 | 51.676 | 23.339 | 5.1 | 2 | 3 | 2 | 1.09 | 1.1% |
| D3ZYU1 | Protein Wdr93 | 78.67 | 2.5487 | 1.2 | 1 | 1 | 1 | 1.09 | 1.4% |
| D4A4I9 | Interleukin 16 (Mapped) | 141.58 | 2.1417 | 0.6 | 1 | 2 | 1 | 1.09 | 2.6% |
| A0A0G2K2D6 | Protein Tppp | 25.583 | 33.618 | 13.6 | 3 | 5 | 3 | 1.09 | 4.8% |
| D3ZFX4 | Phosphoglucomutase 3 (Predicted), isoform CRA_a | 60.845 | 178.59 | 48.6 | 23 | 37 | 23 | 1.09 | 2.0% |
| Q5XHY5 | Threonine--tRNA ligase, cytoplasmic | 80.575 | 194.98 | 40 | 24 | 49 | 23 | 1.09 | 1.4% |
| P25113 | Phosphoglycerate mutase 1 | 28.832 | 154.4 | 47.6 | 11 | 38 | 11 | 1.09 | 0.9% |
| D3ZUC9 | Oxidative-stress responsive 1 (Predicted) | 58.204 | 123.48 | 28.1 | 15 | 22 | 11 | 1.09 | 1.2% |
| M0RAS3 | Protein Ccdc137 | 32.803 | 2.5184 | 8.7 | 2 | 2 | 2 | 1.09 | 1.6% |
| P13697 | NADP-dependent malic enzyme | 64.002 | 212.93 | 38.1 | 17 | 39 | 17 | 1.09 | 0.6% |
| Q5RK19 | Vacuolar-sorting protein SNF8 | 28.884 | 62.661 | 34.5 | 8 | 11 | 8 | 1.09 | 0.1% |
| A0A0G2KAX0 | Protein Psme4 | 212.98 | 17.21 | 3.4 | 5 | 6 | 5 | 1.09 | 1.4% |
| Q6IRJ7 | Annexin | 50.019 | 80.054 | 28.5 | 12 | 18 | 12 | 1.09 | 1.3% |
| P05982 | NAD(P)H dehydrogenase [quinone] 1 | 30.946 | 126.04 | 39.4 | 15 | 55 | 15 | 1.09 | 0.5% |
| P04961 | Proliferating cell nuclear antigen | 28.748 | 58.047 | 34.9 | 8 | 11 | 8 | 1.09 | 1.8% |
| P40307 | Proteasome subunit beta type-2 | 22.912 | 49.54 | 24.4 | 5 | 9 | 5 | 1.09 | 2.7% |
| Q6NX65 | Programmed cell death protein 10 | 24.355 | 33.765 | 32.9 | 6 | 6 | 6 | 1.09 | 5.7% |
| Q63663 | Interferon-induced guanylate-binding protein 2 | 67.108 | 62.643 | 13.8 | 8 | 9 | 8 | 1.09 | 2.6% |
| E9PTB2 | Transcription elongation factor SPT5 | 121.04 | 10.533 | 3 | 3 | 3 | 3 | 1.09 | 2.6% |
| A0A0G2JWM7 | Protein LOC100911249 | 21.459 | 2.573 | 7.3 | 1 | 1 | 1 | 1.09 | 8.9% |
| P54319 | Phospholipase A-2-activating protein | 87.083 | 167.18 | 28.3 | 21 | 31 | 21 | 1.09 | 1.3% |
| O88767 | Protein DJ-1 | 19.974 | 131.77 | 76.2 | 13 | 32 | 13 | 1.09 | 0.7% |
| Q9QZA2 | Programmed cell death 6-interacting protein | 96.63 | 262.3 | 44.4 | 37 | 69 | 37 | 1.09 | 1.1% |
| O70467 | Protein arginine N-methyltransferase 3 | 59.419 | 8.3783 | 2.7 | 1 | 2 | 1 | 1.09 | 9.5% |
| D4A3T4 | Protein Zfyve1 | 86.903 | 17.162 | 1.8 | 1 | 1 | 1 | 1.09 | 6.1% |
| D3ZBE5 | Serine/threonine-protein kinase Nek7 | 34.529 | 17.403 | 16.9 | 5 | 12 | 5 | 1.09 | 1.2% |
| M0R5J4 | Alpha-enolase | 47.074 | 323.31 | 71 | 30 | 150 | 25 | 1.09 | 1.2% |
| F1LRL4 | Protein Tbc1d9b | 141.87 | 48.005 | 6.7 | 8 | 9 | 8 | 1.09 | 4.4% |
| Q5QJC9 | BAG family molecular chaperone regulator 5 | 51.03 | 25.385 | 12.5 | 5 | 6 | 5 | 1.09 | 1.1% |
| Q8CGV7 | Thiamine-triphosphatase | 24.543 | 3.4448 | 8 | 2 | 2 | 2 | 1.09 | 1.5% |
| A0A0G2K9C0 | Protein Vasp | 39.578 | 61.308 | 26.1 | 9 | 15 | 9 | 1.09 | 2.5% |
| F1M6L8 | Uncharacterized protein | 7.0242 | 2.1662 | 15.6 | 1 | 2 | 1 | 1.09 | 10.1% |
| D4AE58 | Protein Kank1 | 148.01 | 2.2163 | 0.8 | 1 | 1 | 1 | 1.09 | 10.7% |
| Q6J2U6 | E3 ubiquitin-protein ligase RNF114 | 25.664 | 8.5827 | 12.2 | 2 | 2 | 2 | 1.09 | 7.1% |
| D3ZDK7 | Protein Pgp | 34.6 | 38.474 | 18.7 | 4 | 6 | 4 | 1.09 | 5.3% |
| Q4V7E8 | Leucine-rich repeat flightless-interacting protein 2 | 49.772 | 18.415 | 16.9 | 6 | 10 | 5 | 1.09 | 4.3% |
| D3ZEY4 | Diacylglycerol kinase | 102.54 | 12.994 | 2.9 | 2 | 2 | 2 | 1.09 | 3.9% |
| P83868 | Prostaglandin E synthase 3 | 18.721 | 54.673 | 35.6 | 7 | 12 | 7 | 1.09 | 2.8% |
| Q9R063 | Peroxiredoxin-5, mitochondrial | 22.178 | 225.26 | 59.6 | 14 | 55 | 14 | 1.09 | 1.1% |
| F1M1D5 | Protein Tbcd | 133.54 | 110.27 | 16.3 | 17 | 24 | 17 | 1.09 | 1.0% |
| Q4V8C3 | Echinoderm microtubule-associated protein-like 1 | 89.801 | 113.14 | 21.1 | 18 | 32 | 18 | 1.09 | 0.1% |
| O35824 | DnaJ homolog subfamily A member 2 | 45.765 | 99.239 | 30.3 | 12 | 23 | 12 | 1.09 | 0.6% |
| Q6PEC1 | Tubulin-specific chaperone A | 12.744 | 75.564 | 71.3 | 11 | 16 | 11 | 1.09 | 0.6% |
| Q63544 | Gamma-synuclein | 12.976 | 21.24 | 27.6 | 3 | 4 | 3 | 1.09 | 4.8% |
| A0A0G2K9U6 | Protein Atg16l1 | 69.621 | 21.414 | 10.6 | 7 | 8 | 7 | 1.09 | 2.6% |
| A0A0G2K865 | Protein Tpd52 | 26.883 | 76.333 | 38.5 | 8 | 12 | 8 | 1.09 | 2.3% |
| Q6MG06 | Guanine nucleotide-binding protein-like 1 | 68.706 | 29.031 | 10 | 6 | 9 | 6 | 1.09 | 0.1% |
| O35828 | Coronin-7 | 100.78 | 23.419 | 9.1 | 7 | 8 | 7 | 1.09 | 3.0% |
| P35815 | Protein phosphatase 1B | 42.889 | 64.756 | 25.6 | 7 | 7 | 6 | 1.09 | 3.2% |
| D3Z994 | Protein Tbc1d22a | 41.848 | 8.667 | 9.4 | 3 | 3 | 3 | 1.09 | 3.3% |
| Q5I0G4 | Glycine--tRNA ligase | 72.029 | 240.67 | 43.5 | 26 | 45 | 26 | 1.09 | 3.4% |
| D3ZPF5 | Protein Smarcc2 | 131.99 | 62.362 | 9.5 | 10 | 12 | 7 | 1.09 | 7.1% |
| Q5BJZ6 | UPF0586 protein C9orf41 homolog | 46.384 | 4.0022 | 2.8 | 1 | 1 | 1 | 1.09 | 4.0% |
| Q5I0K7 | UDP-N-acetylglucosamine transferase subunit ALG13 homolog | 18.329 | 10.356 | 9.7 | 1 | 1 | 1 | 1.09 | 14.3% |
| D4A511 | Signal recognition particle 9 kDa protein | 10.137 | 12.773 | 53.5 | 5 | 8 | 5 | 1.09 | 14.1% |
| B2RYQ2 | Serine/threonine-protein phosphatase 2A activator | 36.617 | 26.129 | 21.4 | 6 | 14 | 6 | 1.09 | 4.9% |
| Q9R1T1 | Barrier-to-autointegration factor | 10.044 | 54.341 | 64 | 7 | 13 | 7 | 1.09 | 2.8% |
| Q80W92 | Protein VAC14 homolog | 88.067 | 120.4 | 17.5 | 12 | 12 | 12 | 1.09 | 2.0% |
| Q64537 | Calpain small subunit 1 | 28.57 | 111.57 | 37.8 | 8 | 28 | 8 | 1.09 | 0.7% |
| Q6Q0N1 | Cytosolic non-specific dipeptidase | 52.693 | 147.65 | 36.6 | 15 | 27 | 15 | 1.09 | 0.7% |
| D3ZPN3 | Myeloid leukemia factor 2 (Predicted), isoform CRA_a | 28.055 | 2.0834 | 8.1 | 2 | 2 | 2 | 1.09 | 1.6% |
| Q5U3Y8 | Transcription factor BTF3 | 17.699 | 110.62 | 64.8 | 6 | 9 | 6 | 1.09 | 2.3% |
| D2XV59 | GTP-binding protein 1 | 72.488 | 37.171 | 4.2 | 2 | 3 | 2 | 1.09 | 2.7% |
| D4AC23 | Protein Cct7 | 59.658 | 262.18 | 55 | 24 | 42 | 24 | 1.09 | 3.4% |
| B5DFH7 | Protein Traf2 | 55.562 | 10.779 | 2.6 | 1 | 1 | 1 | 1.09 | 5.5% |
| Q5XFW8 | Protein SEC13 homolog | 35.547 | 99.079 | 33.5 | 8 | 16 | 8 | 1.09 | 2.9% |
| B5DEH4 | Protein Uap1l1 | 56.435 | 91.088 | 26 | 13 | 17 | 11 | 1.09 | 0.3% |
| P50503 | Hsc70-interacting protein | 41.279 | 78.988 | 28.8 | 12 | 30 | 12 | 1.09 | 0.1% |
| Q6AYD5 | G1 to S phase transition 1 | 68.751 | 190.51 | 38.7 | 24 | 35 | 24 | 1.09 | 1.1% |
| Q9QYU4 | Ketimine reductase mu-crystallin | 33.554 | 26.978 | 17.3 | 4 | 4 | 4 | 1.09 | 2.0% |
| P97924 | Kalirin | 336.58 | 30.247 | 2.3 | 6 | 6 | 5 | 1.09 | 2.4% |
| P30835 | ATP-dependent 6-phosphofructokinase, liver type | 85.338 | 79.226 | 20.1 | 13 | 17 | 9 | 1.09 | 4.8% |
| Q9ESW0 | DNA damage-binding protein 1 | 126.86 | 112 | 21.1 | 23 | 30 | 23 | 1.09 | 0.1% |
| P50137 | Transketolase | 67.643 | 323.31 | 48.2 | 27 | 60 | 27 | 1.09 | 1.6% |
| F1LSM0 | Ubiquitin carboxyl-terminal hydrolase | 294.03 | 16.388 | 1.9 | 5 | 5 | 5 | 1.09 | 7.5% |
| Q5GFD9 | Protein IMPACT | 35.995 | 55.372 | 31.5 | 7 | 7 | 7 | 1.09 | 3.5% |
| Q9R085 | Ubiquitin carboxyl-terminal hydrolase 15 | 109.25 | 56.844 | 14 | 12 | 15 | 11 | 1.09 | 2.4% |
| O88656 | Actin-related protein 2/3 complex subunit 1B | 41.056 | 156.97 | 38.4 | 12 | 31 | 12 | 1.09 | 1.3% |
| M0R919 | Protein Vbp1 | 20.929 | 83.046 | 46.8 | 7 | 10 | 7 | 1.09 | 0.7% |
| Q2LAP6 | Testin | 47.632 | 163.68 | 48 | 16 | 25 | 16 | 1.09 | 2.2% |
| A0A096MKE9 | DNA ligase | 112.02 | 14.681 | 3.5 | 3 | 3 | 3 | 1.09 | 2.9% |
| Q566Q8 | UPF0696 protein C11orf68 homolog | 27.493 | 23.748 | 16.7 | 4 | 5 | 4 | 1.09 | 2.9% |
| D3ZK09 | Protein Scyl3 | 81.529 | 14.282 | 2.3 | 1 | 1 | 1 | 1.09 | 10.0% |
| D4A9A3 | Protein Cenpv | 27.503 | 17.954 | 6.8 | 1 | 1 | 1 | 1.09 | 7.2% |
| D3ZAP9 | Glycerol-3-phosphate dehydrogenase [NAD(+)] | 38.066 | 44.999 | 20.8 | 6 | 7 | 6 | 1.09 | 1.5% |
| A0A096MKC0 | Protein Gigyf2 (Fragment) | 83.432 | 16.628 | 4.5 | 2 | 2 | 2 | 1.09 | 0.4% |
| Q68FR9 | Elongation factor 1-delta | 31.33 | 116.84 | 55.2 | 14 | 29 | 14 | 1.09 | 1.5% |
| G3V9S9 | Protein Sec24d | 112.86 | 81.145 | 16.4 | 16 | 21 | 16 | 1.09 | 1.7% |
| Q64559 | Cytosolic acyl coenzyme A thioester hydrolase | 42.735 | 31.909 | 19.4 | 6 | 10 | 6 | 1.09 | 3.1% |
| Q91XS8 | Serine/threonine-protein kinase 17B | 42.132 | 9.8333 | 7.3 | 2 | 3 | 2 | 1.09 | 5.2% |
| D3ZFH7 | Protein Stxbp5l | 122.8 | 23.797 | 3.1 | 2 | 2 | 2 | 1.09 | 6.2% |
| Q09429 | ATP-binding cassette sub-family C member 8 | 177.18 | 2.4075 | 0.6 | 1 | 3 | 1 | 1.09 | 8.6% |
| A0A0G2JVI4 | Protein Fuk | 119.55 | 19.986 | 3.1 | 3 | 3 | 3 | 1.09 | 9.1% |
| F1LSC4 | Protein Dhx57 | 155.76 | 44.325 | 3.5 | 3 | 4 | 3 | 1.09 | 11.5% |
| Q63692 | Hsp90 co-chaperone Cdc37 | 44.51 | 155.96 | 42.2 | 16 | 32 | 16 | 1.09 | 1.5% |
| F7FFR1 | Protein Rars2 | 65.228 | 26.723 | 6.6 | 3 | 3 | 3 | 1.09 | 4.1% |
| Q91XR8 | Phospholipid hydroperoxide glutathione peroxidase, nuclear | 29.334 | 27.502 | 22.5 | 5 | 7 | 5 | 1.09 | 2.1% |
| F1LNF0 | Protein Myh14 | 228.91 | 28.689 | 6.8 | 18 | 54 | 3 | 1.09 | 1.2% |
| D4AE56 | Prostaglandin E synthase 2 (Predicted), isoform CRA_b | 43.451 | 7.729 | 6 | 2 | 2 | 2 | 1.09 | 5.8% |
| P55213 | Caspase-3 | 31.491 | 7.6575 | 10.1 | 3 | 3 | 3 | 1.09 | 6.9% |
| O35397 | Caspase-6 | 31.556 | 1.9774 | 3.6 | 1 | 1 | 1 | 1.09 | 1.9% |
| D3ZUV3 | Eukaryotic translation initiation factor 2 subunit 1 | 65.348 | 135.59 | 26.3 | 11 | 13 | 11 | 1.09 | 1.2% |
| B5DF89 | Cullin-3 | 88.931 | 92.032 | 22.4 | 15 | 22 | 15 | 1.09 | 0.8% |
| A0A0G2JZZ3 | Protein Ctif | 67.389 | 36.971 | 8.5 | 5 | 7 | 5 | 1.09 | 12.4% |
| Q8R491 | EH domain-containing protein 3 | 60.79 | 119.08 | 39.8 | 19 | 36 | 8 | 1.09 | 2.7% |
| P43424 | Galactose-1-phosphate uridylyltransferase | 43.314 | 4.7702 | 6.1 | 2 | 2 | 2 | 1.09 | 1.3% |
| P82458 | E3 ubiquitin-protein ligase Midline-1 | 75.21 | 153.52 | 34 | 21 | 32 | 21 | 1.09 | 0.5% |
| D4A777 | Protein Fam114a1 | 60.757 | 162.73 | 30.9 | 11 | 20 | 11 | 1.09 | 0.4% |
| B4F7A3 | Galectin | 18.955 | 12.68 | 11.6 | 2 | 3 | 2 | 1.09 | 2.4% |
| D3ZUI1 | APAF1 interacting protein (Predicted), isoform CRA_a | 27.053 | 20.532 | 12.9 | 3 | 5 | 3 | 1.08 | 3.3% |
| F1M3K6 | Protein Bag4 | 49.025 | 8.3027 | 3.9 | 2 | 2 | 2 | 1.08 | 2.6% |
| Q8K3Y6 | Zinc finger CCCH-type antiviral protein 1 | 86.77 | 24.623 | 7.3 | 5 | 6 | 5 | 1.08 | 1.5% |
| A0A0G2K6E0 | Protein Rhoc | 23.908 | 12.593 | 40.5 | 8 | 19 | 3 | 1.08 | 1.8% |
| B1WC42 | LOC686323 protein | 50.915 | 60.549 | 19.2 | 6 | 7 | 6 | 1.08 | 2.7% |
| A0A0G2JWG0 | Protein Ntan1 | 34.887 | 2.9886 | 2.6 | 1 | 1 | 1 | 1.08 | 3.0% |
| F1LZJ4 | Hydroxypyruvate isomerase | 30.589 | 47.078 | 28.9 | 5 | 8 | 5 | 1.08 | 6.2% |
| Q5HZE4 | Methylthioribose-1-phosphate isomerase | 39.587 | 2.7385 | 2.7 | 1 | 4 | 1 | 1.08 | 7.3% |
| P85972 | Vinculin | 116.61 | 323.31 | 77.7 | 85 | 291 | 85 | 1.08 | 0.6% |
| P47875 | Cysteine and glycine-rich protein 1 | 20.613 | 238.34 | 68.9 | 13 | 70 | 13 | 1.08 | 0.2% |
| Q5XIP0 | DnaJ (Hsp40) homolog, subfamily B, member 4 | 37.822 | 118.2 | 38.3 | 14 | 28 | 13 | 1.08 | 1.3% |
| F1MAC6 | Protein Ston1 | 81.931 | 22.505 | 5.7 | 4 | 5 | 4 | 1.08 | 6.1% |
| Q6AXT1 | Four and a half LIM domains protein 5 | 32.97 | 36.062 | 34.9 | 10 | 13 | 10 | 1.08 | 7.7% |
| F1MAQ7 | Protein Son | 266.12 | 23.079 | 2.4 | 5 | 7 | 5 | 1.08 | 8.4% |
| O35244 | Peroxiredoxin-6 | 24.818 | 157.2 | 77.7 | 16 | 36 | 16 | 1.08 | 1.5% |
| D3ZLS5 | Protein Hectd1 | 289.03 | 123.54 | 9.3 | 21 | 23 | 21 | 1.08 | 2.4% |
| F1LRC6 | Protein LOC100911302 | 171.48 | 34.032 | 2.5 | 3 | 3 | 3 | 1.08 | 13.5% |
| P11980 | Pyruvate kinase PKM | 57.817 | 323.31 | 71.2 | 37 | 128 | 9 | 1.08 | 0.8% |
| D4ADF5 | Protein LOC100912106 | 14.204 | 42.145 | 59.2 | 7 | 14 | 7 | 1.08 | 1.9% |
| D4AEH9 | Amylo-1, 6-glucosidase, 4-alpha-glucanotransferase (Glycogen debranching enzyme, glycogen storage disease type III) (Predicted), isoform CRA_a | 174.33 | 95.712 | 12.4 | 17 | 20 | 17 | 1.08 | 2.8% |
| P04906 | Glutathione S-transferase P | 23.439 | 69.791 | 31.9 | 5 | 20 | 5 | 1.08 | 0.9% |
| Q5XFW6 | WD repeat-containing protein 6 | 121.94 | 23.366 | 4.2 | 3 | 4 | 3 | 1.08 | 5.2% |
| Q6AY80 | Ribosyldihydronicotinamide dehydrogenase [quinone] | 26.275 | 41.86 | 32 | 7 | 8 | 7 | 1.08 | 3.5% |
| D4A4W6 | Protein Slirp | 12.576 | 5.1495 | 9 | 1 | 3 | 1 | 1.08 | 2.2% |
| A0A096MJ11 | Gasdermin domain containing 1 (Predicted), isoform CRA_a | 53.212 | 83.103 | 21.9 | 10 | 11 | 10 | 1.08 | 1.2% |
| Q5M7U6 | Actin-related protein 2 | 44.733 | 172.02 | 39.6 | 16 | 52 | 16 | 1.08 | 0.6% |
| B5DFA1 | TBC1 domain family member 2A | 104.97 | 11.475 | 3.7 | 3 | 5 | 3 | 1.08 | 2.4% |
| Q9Z1Y3 | Cadherin-2 | 99.685 | 21.097 | 5.3 | 3 | 4 | 3 | 1.08 | 8.6% |
| P0DMW1 | Heat shock 70 kDa protein 1B | 70.184 | 273.8 | 56.8 | 34 | 74 | 17 | 1.08 | 0.4% |
| Q5U2T9 | FK506 binding protein 5 | 50.964 | 44.435 | 18.2 | 7 | 9 | 7 | 1.08 | 7.1% |
| D4A857 | Importin 9 (Predicted) | 116.01 | 97.3 | 12.2 | 11 | 15 | 11 | 1.08 | 6.9% |
| Q5M7T9 | Threonine synthase-like 2 | 54.137 | 26.265 | 11.3 | 4 | 5 | 4 | 1.08 | 5.9% |
| D4A2D7 | Importin 4 (Predicted), isoform CRA_b | 118.93 | 108.96 | 20.9 | 17 | 22 | 17 | 1.08 | 5.1% |
| P09811 | Glycogen phosphorylase, liver form | 97.482 | 16.416 | 7.6 | 6 | 6 | 4 | 1.08 | 5.5% |
| Q6AXZ0 | Polyadenylate-binding protein-interacting protein 2 | 14.7 | 45.933 | 15.3 | 1 | 1 | 1 | 1.08 | 12.6% |
| P31044 | Phosphatidylethanolamine-binding protein 1 | 20.801 | 232.57 | 85 | 10 | 34 | 10 | 1.08 | 1.8% |
| D3ZPY1 | Protein Prkg1 | 55.271 | 70.021 | 30.7 | 14 | 16 | 14 | 1.08 | 0.7% |
| Q68FS8 | Protein Rtcd1 | 39.313 | 152.29 | 35.5 | 10 | 20 | 10 | 1.08 | 2.8% |
| G3V9Y9 | Adaptor-related protein complex 3, sigma 1 subunit (Predicted), isoform CRA_a | 18.135 | 29.872 | 14.6 | 2 | 3 | 2 | 1.08 | 9.1% |
| F1LSC9 | Protein Bcas3 | 58.086 | 13.882 | 2.6 | 1 | 3 | 1 | 1.08 | 6.8% |
| D4A567 | Protein Bub3 | 67.846 | 17.102 | 8 | 4 | 5 | 4 | 1.08 | 2.1% |
| D4AE96 | Importin 7 (Predicted), isoform CRA_c | 119.49 | 127.3 | 13.6 | 12 | 20 | 11 | 1.08 | 5.6% |
| D3ZGS3 | Inositol polyphosphate 5-phosphatase OCRL-1 | 104.41 | 23.816 | 4.8 | 3 | 4 | 3 | 1.08 | 7.8% |
| D3ZEP6 | Protein S100-A10 | 11.17 | 47.684 | 47.9 | 6 | 14 | 6 | 1.08 | 4.9% |
| D4AD33 | Protein Fam103a1 | 14.414 | 15.448 | 23.5 | 2 | 2 | 2 | 1.08 | 3.5% |
| Q3MHS9 | Chaperonin containing Tcp1, subunit 6A (Zeta 1) | 58.017 | 178.49 | 45 | 19 | 36 | 19 | 1.08 | 3.0% |
| D3ZYQ8 | Uridine-cytidine kinase | 60.826 | 25.458 | 16.2 | 9 | 13 | 9 | 1.08 | 1.2% |
| Q71UE8 | NEDD8 | 8.9723 | 24.33 | 33.3 | 4 | 10 | 4 | 1.08 | 1.2% |
| P39052 | Dynamin-2 | 98.229 | 148.88 | 30.5 | 21 | 32 | 17 | 1.08 | 0.6% |
| P61954 | Guanine nucleotide-binding protein G(I)/G(S)/G(O) subunit gamma-11 | 8.4807 | 7.1026 | 28.8 | 2 | 2 | 2 | 1.08 | 0.6% |
| M0R5N3 | Microtubule-associated protein RP/EB family member 2 | 28.134 | 25.681 | 20.8 | 4 | 8 | 3 | 1.08 | 1.0% |
| O35964 | Endophilin-A2 | 41.492 | 116.31 | 44.3 | 17 | 26 | 17 | 1.08 | 1.5% |
| D4ADP9 | Coatomer protein complex, subunit zeta 2 (Predicted), isoform CRA_a | 23.267 | 45.808 | 24.2 | 4 | 9 | 4 | 1.08 | 1.3% |
| Q8CG45 | Aflatoxin B1 aldehyde reductase member 2 | 40.675 | 32.001 | 24 | 8 | 11 | 8 | 1.08 | 0.6% |
| B2GV73 | Actin-related protein 2/3 complex subunit 3 | 20.535 | 44.991 | 43.3 | 9 | 17 | 9 | 1.08 | 0.0% |
| A0A0G2K9N3 | Protein Ddx3 | 73.033 | 22.861 | 40.6 | 26 | 59 | 4 | 1.08 | 1.2% |
| Q5PQS4 | PTB domain-containing engulfment adapter protein 1 | 34.285 | 43.768 | 31.9 | 10 | 12 | 10 | 1.08 | 3.6% |
| F1M7Y5 | Protein kinase C iota type | 68.276 | 7.7152 | 5.2 | 3 | 5 | 3 | 1.08 | 4.2% |
| D4A2H2 | Protein Sptlc1 | 52.548 | 15.968 | 10.1 | 4 | 5 | 4 | 1.08 | 5.1% |
| Q68FU1 | Pleckstrin homology domain-containing family F member 1 | 31.441 | 5.0538 | 3.6 | 1 | 3 | 1 | 1.08 | 5.1% |
| Q6AYT0 | Quinone oxidoreductase | 34.975 | 64.924 | 28 | 9 | 12 | 9 | 1.08 | 1.7% |
| F1M0R1 | Protein Rnf213 | 583.37 | 3.5974 | 0.6 | 3 | 3 | 2 | 1.08 | 6.6% |
| Q5XIM0 | BCS1-like (Yeast) | 47.393 | 21.428 | 9.6 | 4 | 4 | 4 | 1.08 | 2.5% |
| P62845 | 40S ribosomal protein S15 | 17.04 | 37.063 | 26.2 | 3 | 13 | 3 | 1.08 | 1.4% |
| D3ZBL6 | Protein Nup160 | 137.86 | 62.347 | 7.4 | 6 | 6 | 6 | 1.08 | 9.9% |
| Q5M823 | NudC domain-containing protein 2 | 17.674 | 32.643 | 29.9 | 4 | 7 | 4 | 1.08 | 2.4% |
| Q4KMA2 | UV excision repair protein RAD23 homolog B | 43.496 | 65.842 | 28.4 | 13 | 22 | 11 | 1.08 | 1.5% |
| P82995 | Heat shock protein HSP 90-alpha | 84.814 | 323.31 | 58.4 | 45 | 138 | 33 | 1.08 | 0.7% |
| F1LSK5 | Protein Heatr5a | 220.21 | 89.372 | 6 | 10 | 14 | 10 | 1.08 | 5.4% |
| B3DMA5 | CCR4-NOT transcription complex, subunit 7 | 32.718 | 9.8091 | 8.4 | 2 | 3 | 2 | 1.08 | 7.3% |
| Q920G2 | Na(+)/H(+) exchange regulatory cofactor NHE-RF2 | 37.368 | 9.3047 | 7.4 | 2 | 2 | 2 | 1.08 | 6.3% |
| G3V7I7 | Protein Lrrc40 | 67.94 | 25.661 | 13 | 7 | 8 | 7 | 1.08 | 1.8% |
| Q5XI43 | Matrix-remodeling-associated protein 8 | 42.735 | 15.304 | 10.2 | 3 | 3 | 3 | 1.08 | 10.3% |
| Q6P7B0 | Tryptophan--tRNA ligase, cytoplasmic | 54.143 | 146.71 | 47.8 | 18 | 26 | 18 | 1.08 | 1.3% |
| P42667 | Signal peptidase complex catalytic subunit SEC11A | 20.599 | 27.765 | 41.3 | 8 | 13 | 8 | 1.08 | 0.9% |
| Q9R1R4 | Tudor domain-containing protein 7 | 125.31 | 15.35 | 2.2 | 2 | 2 | 2 | 1.08 | 3.6% |
| Q62639 | GTP-binding protein Rheb | 20.479 | 31.377 | 22.3 | 4 | 4 | 4 | 1.08 | 2.1% |
| M0R9U5 | Nuclear migration protein nudC | 37.779 | 101.86 | 53.5 | 17 | 24 | 17 | 1.08 | 1.4% |
| D3ZCD7 | Protein Tp53rk | 27.427 | 16.354 | 12.7 | 3 | 3 | 3 | 1.08 | 2.5% |
| D3ZMN5 | Protein Snx29 | 90.572 | 8.8757 | 3.1 | 2 | 2 | 2 | 1.08 | 4.6% |
| B2RZ78 | Vacuolar protein sorting-associated protein 29 | 20.468 | 42.512 | 31.9 | 6 | 10 | 6 | 1.08 | 1.8% |
| F1M9Q3 | Protein Ltn1 | 201.94 | 46.67 | 4.1 | 6 | 7 | 6 | 1.08 | 0.5% |
| F1M9V7 | Protein Npepps | 103.34 | 205.77 | 32.6 | 26 | 41 | 26 | 1.08 | 0.0% |
| Q6YH22 | Protein Pkig | 7.9434 | 5.4474 | 21.1 | 1 | 1 | 1 | 1.08 | 0.0% |
| Q6AYF2 | LIM and cysteine-rich domains 1 | 41.085 | 195.12 | 62.2 | 21 | 54 | 21 | 1.08 | 0.1% |
| Q9QXL7 | Nucleoside diphosphate kinase 7 | 44.539 | 2.1166 | 3 | 1 | 1 | 1 | 1.08 | 3.6% |
| F1M3U7 | Protein Ccdc178 | 88.871 | 2.2506 | 1.3 | 1 | 2 | 1 | 1.08 | 6.0% |
| Q9R172 | Neurogenic locus notch homolog protein 3 | 244.3 | 4.5276 | 0.4 | 1 | 1 | 1 | 1.08 | 1.9% |
| Q99JE6 | 1-phosphatidylinositol 4,5-bisphosphate phosphodiesterase beta-3 | 139.45 | 108.65 | 11.9 | 13 | 15 | 13 | 1.08 | 2.0% |
| Q562C6 | Leucine zipper transcription factor-like protein 1 | 34.638 | 43.335 | 24.7 | 6 | 9 | 6 | 1.08 | 5.1% |
| Q561Q8 | Mediator of RNA polymerase II transcription subunit 4 | 29.825 | 20.881 | 4.8 | 1 | 2 | 1 | 1.08 | 5.6% |
| D3ZNQ6 | Protein Ube2m | 20.9 | 47.979 | 37.7 | 7 | 14 | 7 | 1.08 | 3.3% |
| Q5RKI0 | WD repeat-containing protein 1 | 66.181 | 323.31 | 54.1 | 29 | 83 | 29 | 1.08 | 1.4% |
| P34058 | Heat shock protein HSP 90-beta | 83.28 | 323.31 | 58.8 | 45 | 143 | 31 | 1.08 | 0.2% |
| Q5PPJ4 | Deoxyhypusine hydroxylase | 33.076 | 46.069 | 25.5 | 6 | 8 | 6 | 1.08 | 0.8% |
| D3ZZE3 | Protein RGD1564887 | 78.668 | 10.382 | 3.2 | 2 | 3 | 2 | 1.08 | 1.4% |
| D3ZA22 | Protein Trim47 | 70.027 | 25.195 | 10.7 | 5 | 5 | 5 | 1.08 | 3.5% |
| P62275 | 40S ribosomal protein S29 | 6.6767 | 10.336 | 46.4 | 3 | 4 | 3 | 1.08 | 7.4% |
| Q99ND9 | RWD domain-containing protein 1 | 27.782 | 6.8063 | 7.8 | 3 | 3 | 3 | 1.08 | 2.9% |
| B0BN85 | Suppressor of G2 allele of SKP1 homolog | 38.09 | 79.541 | 39.9 | 12 | 14 | 12 | 1.08 | 2.6% |
| D3ZT03 | Protein Upf2 | 148.59 | 13.231 | 1.3 | 1 | 1 | 1 | 1.08 | 2.6% |
| O88989 | Malate dehydrogenase, cytoplasmic | 36.483 | 110.72 | 39.8 | 15 | 30 | 15 | 1.08 | 2.3% |
| P23514 | Coatomer subunit beta | 107.01 | 323.31 | 46 | 35 | 69 | 35 | 1.08 | 2.2% |
| Q4FZT9 | 26S proteasome non-ATPase regulatory subunit 2 | 100.19 | 279.1 | 40.6 | 31 | 51 | 31 | 1.08 | 0.9% |
| A0A0G2K8N9 | Protein Nek9 | 104.85 | 55.183 | 13.8 | 11 | 19 | 11 | 1.08 | 0.5% |
| Q9Z2Q1 | Protein transport protein Sec31A | 135.27 | 266.16 | 27.2 | 34 | 55 | 34 | 1.08 | 0.4% |
| P63155 | Crooked neck-like protein 1 | 83.415 | 16.385 | 3.2 | 2 | 3 | 2 | 1.08 | 0.1% |
| Q6QBQ4 | Phospholipid scramblase 3 | 31.646 | 13.982 | 4.1 | 1 | 1 | 1 | 1.08 | 0.7% |
| Q9EQS0 | Transaldolase | 37.46 | 54.312 | 26.7 | 12 | 20 | 12 | 1.08 | 0.7% |
| D4A415 | Protein Fam175b | 47.936 | 62.996 | 19.1 | 7 | 9 | 7 | 1.08 | 2.0% |
| G3V7Q4 | Protein Ptpn12 | 85.983 | 45.633 | 10.4 | 6 | 7 | 6 | 1.08 | 4.4% |
| P61972 | Nuclear transport factor 2 | 14.478 | 69.608 | 53.5 | 5 | 16 | 5 | 1.08 | 2.6% |
| A0A0G2JVS9 | Protein Ankrd27 | 117.49 | 4.259 | 2.7 | 3 | 3 | 3 | 1.08 | 1.3% |
| Q6F596 | Geranylgeranyl pyrophosphate synthase | 34.777 | 3.4704 | 8 | 2 | 2 | 2 | 1.08 | 0.9% |
| D4AE03 | Protein Engase | 83.066 | 22.951 | 4.1 | 2 | 2 | 2 | 1.08 | 3.5% |
| Q62940 | E3 ubiquitin-protein ligase NEDD4 | 102.39 | 222.78 | 32.9 | 26 | 47 | 23 | 1.08 | 3.9% |
| Q5U2N2 | Ubiquitin carboxyl-terminal hydrolase | 55.976 | 210.23 | 52.3 | 21 | 39 | 21 | 1.08 | 0.1% |
| Q5XIG8 | Serine-threonine kinase receptor-associated protein | 38.456 | 105.17 | 38.6 | 11 | 23 | 11 | 1.08 | 1.6% |
| Q9QVC8 | Peptidyl-prolyl cis-trans isomerase FKBP4 | 51.45 | 41.525 | 24.7 | 10 | 12 | 10 | 1.08 | 1.8% |
| P47245 | Nardilysin | 132.97 | 62.995 | 11.9 | 13 | 15 | 13 | 1.08 | 2.6% |
| D4A8T3 | Coatomer protein complex, subunit zeta 1 (Predicted) | 20.198 | 67.15 | 33.9 | 5 | 7 | 5 | 1.07 | 4.1% |
| Q63228 | Glia maturation factor beta | 16.736 | 85.411 | 45.1 | 7 | 14 | 7 | 1.07 | 3.6% |
| A0A0G2K0C9 | Protein Papola | 83.74 | 4.0862 | 1.3 | 1 | 1 | 1 | 1.07 | 2.8% |
| D4A213 | Protein Scara5 | 53.863 | 20.829 | 6.5 | 2 | 3 | 2 | 1.07 | 0.7% |
| F1M5M9 | Protein Srgap3 | 124.45 | 1.9745 | 2.6 | 3 | 3 | 2 | 1.07 | 0.4% |
| Q4V7C7 | Actin-related protein 3 | 47.357 | 272.28 | 58.1 | 23 | 67 | 23 | 1.07 | 0.3% |
| Q5U2U7 | mRNA cap guanine-N7 methyltransferase | 52.816 | 65.39 | 19.3 | 6 | 10 | 6 | 1.07 | 0.1% |
| P11730 | Calcium/calmodulin-dependent protein kinase type II subunit gamma | 59.037 | 104.2 | 32.3 | 15 | 26 | 9 | 1.07 | 2.7% |
| D3ZRN5 | Protein Trove2 | 60.042 | 26.134 | 9.3 | 5 | 7 | 5 | 1.07 | 2.6% |
| D3ZZM3 | Component of oligomeric golgi complex 4 (Predicted) | 88.602 | 58.191 | 12.6 | 9 | 10 | 9 | 1.07 | 1.3% |
| O88600 | Heat shock 70 kDa protein 4 | 94.055 | 323.31 | 56.7 | 46 | 99 | 43 | 1.07 | 0.6% |
| F1MAH5 | Protein Ppp6r3 | 98.387 | 24.792 | 7.2 | 6 | 7 | 6 | 1.07 | 0.2% |
| Q5HZV9 | Protein phosphatase 1 regulatory subunit 7 | 41.296 | 169.75 | 50.6 | 15 | 22 | 15 | 1.07 | 4.9% |
| D4AE79 | Protein Chmp1a | 21.607 | 7.3147 | 12.8 | 3 | 4 | 3 | 1.07 | 2.0% |
| O70199 | UDP-glucose 6-dehydrogenase | 54.891 | 166.27 | 47.1 | 19 | 37 | 19 | 1.07 | 0.1% |
| D3ZCG3 | Protein Mon2 | 188.24 | 52.628 | 5 | 8 | 9 | 8 | 1.07 | 2.1% |
| D4ABB8 | Probable phospholipid-transporting ATPase IIB | 129.18 | 40.21 | 3.5 | 3 | 3 | 3 | 1.07 | 5.6% |
| P15943 | Amyloid-like protein 2 | 86.882 | 5.2025 | 2.5 | 1 | 1 | 1 | 1.07 | 6.4% |
| Q6AYB4 | Heat shock 70 kDa protein 14 | 54.462 | 68.223 | 12.4 | 6 | 9 | 6 | 1.07 | 2.1% |
| F1LVV4 | Protein Rcc2 | 46.652 | 83.104 | 24.8 | 9 | 10 | 9 | 1.07 | 0.2% |
| Q9QYU2 | Elongation factor Ts, mitochondrial | 35.178 | 26.623 | 17.9 | 3 | 3 | 3 | 1.07 | 7.9% |
| Q641Z8 | Peflin | 30.012 | 30.963 | 15.9 | 4 | 11 | 4 | 1.07 | 4.8% |
| O35142 | Coatomer subunit beta | 102.55 | 245.79 | 40.4 | 31 | 50 | 31 | 1.07 | 0.6% |
| Q32Q06 | AP-1 complex subunit mu-1 | 48.556 | 45.218 | 27.4 | 10 | 15 | 10 | 1.07 | 0.5% |
| Q5XI34 | Protein Ppp2r1a | 65.322 | 206.41 | 43.3 | 22 | 58 | 18 | 1.07 | 0.7% |
| D3ZP47 | Phosphohistidine phosphatase 1 (Predicted), isoform CRA_a | 14.01 | 17.091 | 31.5 | 4 | 5 | 4 | 1.07 | 7.9% |
| D3ZRM9 | 60S ribosomal protein L13 | 24.202 | 45.252 | 40.3 | 11 | 28 | 11 | 1.07 | 2.0% |
| P35704 | Peroxiredoxin-2 | 21.783 | 27.129 | 23.2 | 4 | 11 | 4 | 1.07 | 0.3% |
| D4A8H8 | Cytoplasmic FMR1 interacting protein 1 (Predicted) | 145.26 | 176.77 | 23.5 | 26 | 45 | 17 | 1.07 | 0.2% |
| Q5U300 | Ubiquitin-like modifier-activating enzyme 1 | 117.79 | 323.31 | 38.1 | 33 | 68 | 33 | 1.07 | 1.3% |
| P51111 | Huntingtin | 343.76 | 72.333 | 5.7 | 14 | 17 | 14 | 1.07 | 2.5% |
| D3ZTY0 | Protein Cnih4 | 16.091 | 14.854 | 14.4 | 1 | 2 | 1 | 1.07 | 3.8% |
| D3ZVI9 | Parkinson disease 7 domain containing 1 (Predicted), isoform CRA_a | 23.263 | 28.701 | 13.6 | 2 | 2 | 2 | 1.07 | 5.4% |
| P36876 | Serine/threonine-protein phosphatase 2A 55 kDa regulatory subunit B alpha isoform | 51.677 | 70.27 | 26.6 | 9 | 14 | 6 | 1.07 | 3.9% |
| Q5FVF3 | Golgi associated, gamma adaptin ear containing, ARF binding protein 1 | 61.762 | 23.37 | 11 | 7 | 8 | 7 | 1.07 | 2.6% |
| Q6P7Q1 | BRCA1-A complex subunit BRE | 43.558 | 21.605 | 13.6 | 4 | 6 | 4 | 1.07 | 0.4% |
| P51607 | N-acylglucosamine 2-epimerase | 49.604 | 44.528 | 17.7 | 6 | 8 | 6 | 1.07 | 0.1% |
| D3ZUE3 | Protein Ubxn8 | 31.205 | 2.5517 | 2.9 | 1 | 1 | 1 | 1.07 | 3.7% |
| Q811S9 | Guanine nucleotide-binding protein-like 3 | 60.66 | 51.652 | 18.6 | 9 | 11 | 9 | 1.07 | 3.5% |
| Q0VGK4 | Glycerophosphodiester phosphodiesterase domain-containing protein 1 | 35.81 | 11.433 | 8.9 | 2 | 3 | 2 | 1.07 | 1.0% |
| Q920J4 | Thioredoxin-like protein 1 | 32.249 | 269.23 | 68.5 | 16 | 28 | 16 | 1.07 | 3.3% |
| Q9EPY0 | Caspase recruitment domain-containing protein 9 | 62.631 | 21.418 | 6.2 | 3 | 4 | 3 | 1.07 | 7.2% |
| D4ABY2 | Coatomer subunit gamma-2 | 80.488 | 92.529 | 17.2 | 9 | 15 | 6 | 1.07 | 3.1% |
| G3V6A6 | Protein RGD1566265 | 22.161 | 6.0156 | 8.5 | 2 | 3 | 2 | 1.07 | 2.3% |
| Q6TXG7 | Serine hydroxymethyltransferase | 75.373 | 44.744 | 11.3 | 7 | 15 | 6 | 1.07 | 0.3% |
| P62329 | Thymosin beta-4 | 5.0526 | 55.929 | 88.6 | 8 | 23 | 6 | 1.07 | 0.1% |
| Q4AEF8 | Coatomer subunit gamma-1 | 97.613 | 323.31 | 46.9 | 33 | 73 | 30 | 1.07 | 1.2% |
| D3ZTG2 | Protein Ttc27 | 96.547 | 13.005 | 1.5 | 1 | 2 | 1 | 1.07 | 9.2% |
| D4A3S6 | Protein Mtss1l | 76.809 | 45.012 | 8.5 | 4 | 4 | 4 | 1.07 | 6.4% |
| Q9EPH8 | Polyadenylate-binding protein 1 | 70.7 | 182.59 | 31.3 | 21 | 49 | 12 | 1.07 | 3.7% |
| Q9WTV5 | 26S proteasome non-ATPase regulatory subunit 9 | 24.829 | 44.765 | 36.9 | 7 | 12 | 7 | 1.07 | 1.7% |
| P05065 | Fructose-bisphosphate aldolase A | 39.351 | 323.31 | 72.3 | 29 | 84 | 25 | 1.07 | 0.9% |
| Q9HB97 | Alpha-parvin | 42.291 | 127.36 | 32.8 | 12 | 23 | 11 | 1.07 | 0.0% |
| P52944 | PDZ and LIM domain protein 1 | 35.584 | 189.2 | 67.3 | 20 | 62 | 20 | 1.07 | 2.3% |
| D4AE59 | Serine/threonine-protein kinase STK11 | 49.468 | 19.349 | 12.6 | 4 | 5 | 4 | 1.07 | 2.1% |
| P27321 | Calpastatin | 77.312 | 213.57 | 30.4 | 19 | 35 | 19 | 1.07 | 0.8% |
| P13221 | Aspartate aminotransferase, cytoplasmic | 46.428 | 129.04 | 45 | 16 | 23 | 16 | 1.07 | 0.6% |
| Q5I034 | Uncharacterized protein C12orf43 homolog | 30.056 | 15.762 | 7.6 | 1 | 1 | 1 | 1.07 | 3.2% |
| Q5BJ92 | Serine/threonine-protein phosphatase 4 catalytic subunit | 35.067 | 9.0103 | 11.4 | 3 | 4 | 2 | 1.07 | 1.3% |
| D3ZW08 | Adenylosuccinate lyase | 54.852 | 111.78 | 23.3 | 8 | 10 | 8 | 1.07 | 0.6% |
| Q07205 | Eukaryotic translation initiation factor 5 | 48.954 | 98.911 | 35.9 | 15 | 25 | 15 | 1.07 | 1.7% |
| Q68FT7 | Phenylalanyl-tRNA synthetase, beta subunit | 65.651 | 89.016 | 34.3 | 19 | 27 | 19 | 1.07 | 3.9% |
| Q2M2R8 | Peroxisomal targeting signal 1 receptor | 71.021 | 8.7288 | 5.3 | 3 | 5 | 3 | 1.07 | 23.1% |
| Q8VHK7 | Hepatoma-derived growth factor | 26.488 | 73.644 | 35.9 | 9 | 12 | 8 | 1.07 | 2.3% |
| B0BNM1 | NAD(P)H-hydrate epimerase | 30.89 | 14.623 | 16.3 | 3 | 5 | 3 | 1.07 | 3.8% |
| D4A8X8 | CTTNBP2 N-terminal like (Predicted), isoform CRA_a | 70.076 | 9.2881 | 4.7 | 3 | 4 | 3 | 1.07 | 4.2% |
| Q568Z6 | IST1 homolog | 39.941 | 40.09 | 18.3 | 6 | 11 | 6 | 1.07 | 4.1% |
| Q5XID7 | Armadillo repeat-containing X-linked protein 3 | 42.552 | 49.89 | 17.4 | 5 | 6 | 5 | 1.07 | 2.8% |
| F1M656 | Tubby-like protein | 50.314 | 36.802 | 20.4 | 7 | 9 | 7 | 1.07 | 1.5% |
| P32577 | Tyrosine-protein kinase CSK | 50.746 | 63.859 | 24.9 | 9 | 12 | 9 | 1.07 | 0.9% |
| Q9Z2G8 | Nucleosome assembly protein 1-like 1 | 45.203 | 64.696 | 19.2 | 6 | 14 | 4 | 1.07 | 3.8% |
| Q0D2L2 | Protein Mrps22 | 41.235 | 17.06 | 12.3 | 3 | 3 | 3 | 1.07 | 9.4% |
| D4AAE7 | Protein Slc31a2 | 16.147 | 24.218 | 17.5 | 1 | 1 | 1 | 1.07 | 10.7% |
| A0A0G2KA50 | Protein Vps39 | 101.65 | 45.471 | 8.7 | 7 | 8 | 7 | 1.07 | 1.6% |
| P37285 | Kinesin light chain 1 | 63.744 | 173.37 | 41.4 | 25 | 44 | 19 | 1.07 | 0.7% |
| B2GUX5 | 5-nucleotidase | 33.81 | 11.9 | 12.8 | 3 | 3 | 3 | 1.07 | 0.0% |
| D3ZZC3 | Kelch-like protein 22 | 71.773 | 14.657 | 8.8 | 6 | 7 | 6 | 1.07 | 0.0% |
| P63036 | DnaJ homolog subfamily A member 1 | 44.868 | 69.526 | 33.5 | 12 | 18 | 12 | 1.07 | 1.0% |
| P84083 | ADP-ribosylation factor 5 | 20.529 | 117.89 | 64.4 | 10 | 35 | 5 | 1.07 | 1.0% |
| A0A0G2JW94 | Protein Clint1 | 53.808 | 34.037 | 15.6 | 6 | 6 | 6 | 1.07 | 2.0% |
| F1LRQ6 | CDC23 (Cell division cycle 23, yeast, homolog), isoform CRA_b | 65.935 | 21.965 | 7.4 | 4 | 5 | 4 | 1.07 | 5.9% |
| P29147 | D-beta-hydroxybutyrate dehydrogenase, mitochondrial | 38.201 | 19.017 | 5.8 | 1 | 1 | 1 | 1.07 | 4.8% |
| Q712U5 | cAMP-regulated phosphoprotein 19 | 12.293 | 35.986 | 56.2 | 5 | 10 | 4 | 1.07 | 2.2% |
| G3V624 | Coronin | 53.177 | 150.83 | 41.4 | 25 | 67 | 24 | 1.07 | 0.9% |
| F1M1H0 | Protein Dera | 35.124 | 46.086 | 24.5 | 6 | 7 | 6 | 1.07 | 3.3% |
| P61515 | Putative 60S ribosomal protein L37a | 10.275 | 27.826 | 51.1 | 4 | 7 | 4 | 1.07 | 7.8% |
| Q6PEC4 | S-phase kinase-associated protein 1 | 18.672 | 55.548 | 38.7 | 9 | 17 | 9 | 1.07 | 4.8% |
| Q3B8N8 | Pescadillo homolog | 67.603 | 6.8243 | 4.6 | 3 | 3 | 2 | 1.07 | 4.0% |
| F1M6I7 | Proteasome subunit alpha type | 27.893 | 16.711 | 10 | 2 | 6 | 1 | 1.07 | 3.3% |
| Q9JHL4 | Drebrin-like protein | 48.612 | 143.91 | 39.2 | 14 | 28 | 14 | 1.07 | 0.2% |
| A0A0G2JXG7 | Protein Lrrc16a | 157.49 | 24.709 | 3.8 | 5 | 5 | 5 | 1.07 | 1.9% |
| Q641Y5 | Ubiquitin-like modifier-activating enzyme ATG7 | 77.435 | 101.48 | 24.1 | 12 | 18 | 12 | 1.07 | 2.5% |
| O35303 | Dynamin-1-like protein | 83.907 | 229.34 | 40.7 | 26 | 43 | 26 | 1.07 | 0.0% |
| Q5PQZ9 | NADH dehydrogenase [ubiquinone] 1 subunit C2 | 14.359 | 13.818 | 20 | 3 | 6 | 3 | 1.07 | 0.6% |
| Q62622 | Eukaryotic translation initiation factor 4E-binding protein 1 | 12.404 | 6.4931 | 32.5 | 2 | 2 | 2 | 1.07 | 0.6% |
| Q5RKG9 | Eukaryotic translation initiation factor 4B | 69.063 | 75.482 | 21.9 | 13 | 15 | 13 | 1.07 | 2.4% |
| P62142 | Serine/threonine-protein phosphatase PP1-beta catalytic subunit | 37.186 | 37.337 | 51.1 | 15 | 38 | 4 | 1.07 | 2.0% |
| Q7TM96 | Zinc finger protein 622 | 43.889 | 9.7081 | 6.5 | 2 | 3 | 2 | 1.07 | 3.5% |
| P70470 | Acyl-protein thioesterase 1 | 24.708 | 47.522 | 26.5 | 4 | 7 | 4 | 1.07 | 9.0% |
| Q6AY63 | ADP-sugar pyrophosphatase | 24.117 | 22.287 | 23.3 | 5 | 6 | 5 | 1.07 | 3.6% |
| D3ZCA0 | Proline synthetase co-transcribed (Predicted) | 30.104 | 68.177 | 20.7 | 4 | 10 | 4 | 1.07 | 0.4% |
| D3ZRE7 | Protein Swap70 | 68.773 | 82.126 | 29.7 | 18 | 22 | 18 | 1.07 | 0.6% |
| Q3KR59 | Ubiquitin carboxyl-terminal hydrolase 10 | 87.31 | 19.857 | 5.3 | 4 | 4 | 4 | 1.07 | 4.7% |
| B1WBX6 | Protein Smap2 | 46.493 | 23.693 | 11 | 3 | 3 | 3 | 1.07 | 6.5% |
| F1MA87 | Cyclin-dependent kinase 6 | 36.952 | 31.222 | 13.8 | 4 | 7 | 3 | 1.07 | 4.3% |
| P31232 | Transgelin | 22.603 | 251.85 | 88.6 | 25 | 259 | 24 | 1.07 | 1.0% |
| Q9JIH7 | Serine/threonine-protein kinase WNK1 | 225.11 | 65.527 | 7.1 | 14 | 15 | 14 | 1.07 | 0.2% |
| B0BN93 | 26S proteasome non-ATPase regulatory subunit 13 | 42.817 | 79.195 | 37.2 | 15 | 26 | 15 | 1.07 | 1.8% |
| B1WBZ1 | Embryonal Fyn-associated substrate | 58.988 | 2.93 | 1.8 | 1 | 1 | 1 | 1.07 | 6.7% |
| D3ZNI4 | Protein FAM120C | 78.062 | 5.1853 | 2 | 1 | 1 | 1 | 1.07 | 4.3% |
| P20650 | Protein phosphatase 1A | 42.416 | 53.372 | 30.4 | 9 | 9 | 8 | 1.07 | 2.4% |
| Q5RK30 | Ribosome maturation protein SBDS | 28.752 | 89.953 | 44.8 | 10 | 17 | 10 | 1.07 | 2.7% |
| D4ADE5 | Histone-lysine N-methyltransferase SETD7 | 40.507 | 68.571 | 20.8 | 6 | 9 | 6 | 1.07 | 0.1% |
| Q99PV2 | Protein Stxbp3 | 68.02 | 28.475 | 8.3 | 4 | 5 | 4 | 1.07 | 0.2% |
| F1M403 | Protein Ube2o | 125.27 | 41.616 | 5.5 | 5 | 7 | 5 | 1.07 | 2.4% |
| F1M7Z2 | Protein Iqca1 | 99.313 | -2 | 0.9 | 1 | 1 | 1 | 1.07 | 3.4% |
| Q8K3F3 | Protein phosphatase 1 regulatory subunit 14B | 15.957 | 12.111 | 17 | 1 | 1 | 1 | 1.07 | 5.5% |
| Q6IMX7 | Hsp70-binding protein 1 | 39.19 | 37.649 | 13.4 | 3 | 3 | 3 | 1.07 | 7.5% |
| D4A259 | Polymerase (RNA) II (DNA directed) polypeptide D (Predicted) | 16.311 | 12.828 | 12.7 | 1 | 1 | 1 | 1.07 | 8.2% |
| O35923 | Breast cancer type 2 susceptibility protein homolog | 372.21 | 2.0213 | 0.3 | 1 | 1 | 1 | 1.07 | 10.5% |
| E9PTN4 | Protein Srpk1 | 73.763 | 8.1716 | 2.1 | 1 | 2 | 1 | 1.07 | 6.5% |
| Q4KLL0 | Transcription elongation factor A protein 1 | 33.893 | 37.175 | 21.9 | 7 | 8 | 7 | 1.07 | 2.9% |
| Q6P686 | Osteoclast-stimulating factor 1 | 23.668 | 136.08 | 43.5 | 7 | 12 | 7 | 1.07 | 1.8% |
| M0RDM4 | Histone H2A | 14.068 | 5.3568 | 26.6 | 6 | 12 | 2 | 1.07 | 2.0% |
| O88377 | Phosphatidylinositol 5-phosphate 4-kinase type-2 beta | 47.263 | 17.612 | 19.5 | 8 | 13 | 3 | 1.07 | 4.0% |
| F7EUU4 | Protein Cops5 | 43.647 | 62.286 | 30.6 | 9 | 12 | 9 | 1.07 | 4.6% |
| M0R4D7 | Protein LOC100910820 | 51.696 | 2.6865 | 46 | 20 | 65 | 1 | 1.07 | 5.7% |
| P46892 | Cyclin-dependent kinase 11B | 49.547 | 12.279 | 9.6 | 4 | 4 | 4 | 1.06 | 6.8% |
| G3V7G9 | Eukaryotic translation initiation factor 3 subunit L | 45.082 | 79.249 | 33 | 12 | 15 | 12 | 1.06 | 4.9% |
| Q4V8H5 | Aspartyl aminopeptidase | 52.555 | 151.36 | 38.3 | 14 | 33 | 14 | 1.06 | 2.3% |
| Q9JJM9 | Septin-5 | 42.852 | 106.89 | 32.5 | 12 | 21 | 12 | 1.06 | 1.3% |
| D3ZLL8 | 40S ribosomal protein S15a | 14.854 | 45.896 | 60 | 8 | 24 | 8 | 1.06 | 0.6% |
| Q03114 | Cyclin-dependent-like kinase 5 | 33.254 | 23.349 | 26.7 | 7 | 10 | 6 | 1.06 | 0.4% |
| Q62739 | Rab-3A-interacting protein | 50.95 | 3.4574 | 2.6 | 1 | 1 | 1 | 1.06 | 10.3% |
| Q496Z0 | Elongator complex protein 2 | 91.745 | 43.743 | 7.4 | 5 | 6 | 5 | 1.06 | 11.6% |
| A0A0G2JTX9 | Protein LOC100910823 | 27.639 | 18.65 | 12.6 | 1 | 2 | 1 | 1.06 | 11.0% |
| F1LPW0 | Protein Ighm | 54.871 | 4.0774 | 2 | 1 | 2 | 1 | 1.06 | 7.4% |
| D3ZE49 | Protein Trappc12 | 87.546 | 43.902 | 8.7 | 5 | 5 | 5 | 1.06 | 4.0% |
| Q9Z1N4 | 3(2),5-bisphosphate nucleotidase 1 | 33.174 | 66.377 | 37.3 | 10 | 17 | 10 | 1.06 | 3.4% |
| P30904 | Macrophage migration inhibitory factor | 12.477 | 73.476 | 41.7 | 4 | 13 | 4 | 1.06 | 3.2% |
| P14659 | Heat shock-related 70 kDa protein 2 | 69.641 | 29.479 | 28.4 | 19 | 53 | 4 | 1.06 | 0.4% |
| G3V8A5 | Vacuolar protein sorting-associated protein 35 | 91.726 | 297.37 | 44.5 | 36 | 49 | 36 | 1.06 | 0.1% |
| B5DFC8 | Eukaryotic translation initiation factor 3 subunit C | 105.43 | 120.27 | 22 | 20 | 35 | 20 | 1.06 | 0.9% |
| P19804 | Nucleoside diphosphate kinase B | 17.283 | 131.55 | 84.2 | 12 | 65 | 6 | 1.06 | 3.1% |
| D4ADZ9 | Protein Pus7 | 74.64 | 12.721 | 6.4 | 4 | 5 | 4 | 1.06 | 10.2% |
| A0A0G2K161 | Protein Epb4.1 | 96.854 | 43.72 | 4.4 | 3 | 3 | 2 | 1.06 | 11.9% |
| D3ZWP8 | Protein Lrrc58 | 40.127 | 19.975 | 16.9 | 4 | 5 | 4 | 1.06 | 6.9% |
| Q6PDU1 | Serine/arginine-rich splicing factor 2 | 25.476 | 34.562 | 14.9 | 3 | 7 | 3 | 1.06 | 6.7% |
| Q6QLM7 | Kinesin heavy chain isoform 5A | 116.91 | 24.009 | 8.9 | 9 | 15 | 1 | 1.06 | 6.0% |
| Q05030 | Platelet-derived growth factor receptor beta | 122.83 | 102.55 | 14.2 | 14 | 17 | 14 | 1.06 | 2.6% |
| Q9JM04 | Importin-13 | 108.19 | 12.435 | 4.4 | 3 | 3 | 3 | 1.06 | 1.9% |
| M0RCH6 | Uncharacterized protein | 24.812 | 34.251 | 30.6 | 7 | 12 | 7 | 1.06 | 0.0% |
| Q32PZ3 | Protein unc-45 homolog A | 103.24 | 89.533 | 18.4 | 15 | 20 | 15 | 1.06 | 2.9% |
| Q4KLJ8 | Phosducin-like protein 3 | 27.699 | 27.269 | 25 | 8 | 9 | 8 | 1.06 | 3.0% |
| Q64270 | Translation initiation factor eIF-2B subunit alpha | 33.678 | 15.478 | 11.1 | 3 | 4 | 3 | 1.06 | 5.2% |
| Q4V7B0 | Uncharacterized protein C9orf117 homolog | 62.79 | 2.0491 | 1.3 | 1 | 1 | 1 | 1.06 | 14.9% |
| Q561R9 | Beta-lactamase-like protein 2 | 32.484 | 37.613 | 23.6 | 6 | 8 | 6 | 1.06 | 2.3% |
| O08816 | Neural Wiskott-Aldrich syndrome protein | 54.325 | 32.996 | 12.6 | 5 | 7 | 5 | 1.06 | 1.9% |
| F1MA29 | High mobility group protein B1 | 24.908 | 57.766 | 40.9 | 8 | 18 | 6 | 1.06 | 1.9% |
| Q6P4Z9 | COP9 signalosome complex subunit 8 | 23.235 | 51.602 | 38.3 | 6 | 11 | 6 | 1.06 | 3.0% |
| P0C2N6 | Histone-lysine N-methyltransferase SUV420H2 | 53.606 | 1.8856 | 2.6 | 1 | 1 | 1 | 1.06 | 4.6% |
| D3ZV63 | Protein RGD1307830 | 50.063 | 41.491 | 18.1 | 6 | 7 | 6 | 1.06 | 4.2% |
| M0R757 | Elongation factor 1-alpha | 50.107 | 323.31 | 63.6 | 34 | 138 | 34 | 1.06 | 0.7% |
| Q5XI07 | Lipoma-preferred partner homolog | 68.26 | 320.28 | 46 | 24 | 57 | 22 | 1.06 | 0.5% |
| Q5RJQ4 | NAD-dependent protein deacetylase sirtuin-2 | 39.319 | 62.171 | 21.7 | 6 | 8 | 6 | 1.06 | 1.8% |
| D3ZKG5 | Protein Parvb | 41.701 | 2.5109 | 10.1 | 3 | 4 | 2 | 1.06 | 13.2% |
| P63170 | Dynein light chain 1, cytoplasmic | 10.366 | 79.142 | 51.7 | 5 | 19 | 4 | 1.06 | 4.2% |
| Q5XI77 | Annexin | 54.16 | 94.097 | 24.5 | 11 | 19 | 11 | 1.06 | 1.9% |
| P27952 | 40S ribosomal protein S2 | 31.231 | 78.692 | 37.9 | 12 | 29 | 8 | 1.06 | 1.6% |
| Q9Z1P2 | Alpha-actinin-1 | 102.96 | 323.31 | 74.9 | 68 | 309 | 38 | 1.06 | 1.5% |
| Q07936 | Annexin A2 | 38.678 | 323.31 | 81.1 | 35 | 121 | 35 | 1.06 | 0.1% |
| Q5RJK6 | Inositol polyphosphate-1-phosphatase | 43.359 | 28.135 | 17.2 | 5 | 6 | 5 | 1.06 | 0.0% |
| B5DEN9 | Vacuolar protein sorting-associated protein 28 homolog | 26.11 | 71.004 | 38.2 | 8 | 10 | 8 | 1.06 | 0.2% |
| F1M775 | Protein Diaph1 | 140.3 | 143.93 | 17.1 | 18 | 33 | 18 | 1.06 | 0.3% |
| O70521 | Regulator of G-protein signaling 19 | 24.738 | 10.57 | 6 | 1 | 1 | 1 | 1.06 | 3.3% |
| O88588 | Phosphofurin acidic cluster sorting protein 1 | 104.7 | 12.095 | 3.4 | 3 | 4 | 3 | 1.06 | 6.0% |
| D3ZCT7 | Protein Sec23b | 86.354 | 43.685 | 17.9 | 10 | 18 | 7 | 1.06 | 12.1% |
| Q9Z336 | Dynein light chain Tctex-type 1 | 12.452 | 40.92 | 30.1 | 2 | 3 | 2 | 1.06 | 8.5% |
| Q5XI97 | Alanyl-tRNA editing protein Aarsd1 | 45.093 | 30.522 | 10.4 | 4 | 5 | 4 | 1.06 | 4.3% |
| P25093 | Fumarylacetoacetase | 45.975 | 13.862 | 11.7 | 4 | 4 | 4 | 1.06 | 3.9% |
| D3ZHB7 | Protein Ube3c | 106.61 | 56.929 | 12.4 | 8 | 9 | 8 | 1.06 | 2.5% |
| D4ACW1 | Protein Nop2 | 84.969 | 80.371 | 11.4 | 7 | 10 | 7 | 1.06 | 0.3% |
| A0A0G2K6I4 | Protein Enah | 62.675 | 155.6 | 35.1 | 21 | 30 | 21 | 1.06 | 0.4% |
| F1LPV0 | Protein Nars | 64.127 | 264.28 | 48.9 | 26 | 60 | 25 | 1.06 | 0.6% |
| D3ZSW9 | Protein LOC100910755 | 69.983 | 9.0016 | 4.6 | 3 | 4 | 3 | 1.06 | 3.3% |
| A1A5P0 | Cdc42 effector protein 1 | 41.028 | 49.677 | 24 | 7 | 9 | 7 | 1.06 | 2.9% |
| Q9Z0T0 | Thiopurine S-methyltransferase | 27.691 | 11.19 | 15.4 | 3 | 4 | 3 | 1.06 | 1.5% |
| Q6P3V8 | Eukaryotic translation initiation factor 4A1 | 46.153 | 180.85 | 50 | 21 | 64 | 10 | 1.06 | 0.7% |
| Q4QR73 | DnaJ (Hsp40) homolog, subfamily A, member 4 | 61.875 | 36.663 | 16.8 | 7 | 7 | 7 | 1.06 | 0.5% |
| D3ZZK1 | 40S ribosomal protein S20 | 13.354 | 41.818 | 28.6 | 4 | 16 | 4 | 1.06 | 1.6% |
| Q9JLZ1 | Glutaredoxin-3 | 37.849 | 84.748 | 38.6 | 13 | 22 | 13 | 1.06 | 2.7% |
| P21670 | Proteasome subunit alpha type-4 | 29.497 | 81.728 | 45.2 | 9 | 20 | 9 | 1.06 | 2.9% |
| G3V6U9 | Protein LOC100910833 | 67.42 | 56.179 | 12.2 | 7 | 9 | 7 | 1.06 | 3.4% |
| Q9Z1B2 | Glutathione S-transferase Mu 5 | 26.629 | 26.356 | 28.4 | 6 | 10 | 5 | 1.06 | 2.1% |
| D3ZZA8 | Protein Sec24a | 118.87 | 118.76 | 12.8 | 10 | 17 | 10 | 1.06 | 0.7% |
| Q68FR6 | Elongation factor 1-gamma | 50.06 | 208.21 | 55.8 | 26 | 72 | 26 | 1.06 | 0.6% |
| Q9QXQ0 | Alpha-actinin-4 | 104.91 | 323.31 | 73.9 | 72 | 327 | 46 | 1.06 | 0.3% |
| P46413 | Glutathione synthetase | 52.344 | 84.822 | 33.5 | 13 | 16 | 13 | 1.06 | 1.0% |
| Q3B7V0 | Dehydrogenase/reductase (SDR family) member 3 | 33.614 | 14.716 | 4.3 | 1 | 1 | 1 | 1.06 | 2.1% |
| A0A0G2QC02 | Protein Skiv2l | 137.3 | 133.88 | 16.7 | 16 | 22 | 16 | 1.06 | 5.9% |
| B5DF65 | Biliverdin reductase B (Flavin reductase (NADPH)) | 22.094 | 79.855 | 51 | 9 | 15 | 9 | 1.06 | 5.5% |
| D3ZBP4 | Protein-methionine sulfoxide oxidase MICAL1 | 116.67 | 67.212 | 10.6 | 10 | 13 | 10 | 1.06 | 4.5% |
| P15791 | Calcium/calmodulin-dependent protein kinase type II subunit delta | 60.08 | 142.04 | 33.6 | 17 | 41 | 12 | 1.06 | 1.8% |
| B2RYG6 | Ubiquitin thioesterase OTUB1 | 31.27 | 116.5 | 51.7 | 11 | 23 | 11 | 1.06 | 0.7% |
| Q9JJ31 | Cullin-5 | 90.889 | 110.16 | 24.6 | 18 | 20 | 18 | 1.06 | 1.2% |
| D3ZU54 | Density-regulated protein | 21.825 | 16.196 | 18.6 | 4 | 6 | 4 | 1.06 | 5.0% |
| Q499N6 | UBX domain-containing protein 1 | 33.581 | 46.11 | 26.3 | 6 | 6 | 6 | 1.06 | 7.0% |
| B5DFA5 | KDEL (Lys-Asp-Glu-Leu) containing 1, isoform CRA_a | 58.11 | 4.173 | 5.2 | 2 | 2 | 2 | 1.06 | 3.0% |
| P38062 | Methionine aminopeptidase 2 | 53.051 | 73.376 | 19 | 7 | 12 | 7 | 1.06 | 1.1% |
| Q7TPB1 | T-complex protein 1 subunit delta | 58.099 | 265.09 | 55.3 | 25 | 52 | 25 | 1.06 | 0.8% |
| A0A0G2JWC7 | Protein Fermt2 | 79.623 | 250.49 | 40.1 | 24 | 69 | 24 | 1.06 | 1.3% |
| P60522 | Gamma-aminobutyric acid receptor-associated protein-like 2 | 13.667 | 2.4891 | 6.8 | 1 | 1 | 1 | 1.06 | 9.2% |
| Q4TU93 | C-type mannose receptor 2 | 167.02 | 110.14 | 10.6 | 15 | 21 | 15 | 1.06 | 2.5% |
| P38983 | 40S ribosomal protein SA | 32.824 | 143.73 | 49.2 | 12 | 29 | 12 | 1.06 | 0.4% |
| D3ZZ62 | Exportin, tRNA (Nuclear export receptor for tRNAs) (Predicted), isoform CRA_a | 83.483 | 39.946 | 9.9 | 6 | 8 | 6 | 1.06 | 2.1% |
| B5DEG8 | LOC685144 protein | 118.52 | 85.706 | 10.9 | 10 | 15 | 9 | 1.06 | 3.9% |
| Q05982 | Nucleoside diphosphate kinase A | 17.193 | 48.065 | 86.2 | 12 | 44 | 6 | 1.06 | 4.1% |
| Q5RJS8 | Protein-tyrosine sulfotransferase 2 | 42.068 | 15.698 | 10.6 | 4 | 5 | 4 | 1.06 | 7.3% |
| P61983 | 14-3-3 protein gamma | 28.302 | 117.15 | 55.5 | 14 | 52 | 9 | 1.06 | 0.6% |
| P63018 | Heat shock cognate 71 kDa protein | 70.87 | 323.31 | 66.1 | 49 | 185 | 35 | 1.06 | 0.2% |
| Q6P730 | Disabled homolog 2-interacting protein | 110 | 2.6187 | 1.7 | 2 | 2 | 1 | 1.06 | 1.9% |
| Q5PPG7 | Eukaryotic translation initiation factor 2D | 62.669 | 41.202 | 14 | 5 | 5 | 5 | 1.06 | 8.0% |
| Q5U216 | ATP-dependent RNA helicase DDX39A | 49.109 | 22.202 | 23 | 11 | 13 | 3 | 1.06 | 1.2% |
| G3V9N0 | Polyadenylate-binding protein | 70.833 | 86.142 | 27.3 | 18 | 36 | 9 | 1.06 | 1.8% |
| F1LXF5 | Protein Get4 | 36.752 | 33.664 | 10.6 | 3 | 4 | 3 | 1.06 | 7.0% |
| D3ZFP4 | DNA helicase | 91.658 | 3.9836 | 1.7 | 1 | 1 | 1 | 1.06 | 6.2% |
| Q4KLH5 | Arf-GAP domain and FG repeat-containing protein 1 | 58.169 | 69.717 | 18.4 | 12 | 21 | 10 | 1.06 | 4.0% |
| Q64649 | Phosphorylase b kinase regulatory subunit alpha, skeletal muscle isoform | 139.15 | 5.501 | 1.2 | 1 | 1 | 1 | 1.06 | 1.1% |
| P63301 | Selenoprotein W | 9.6871 | 3.0268 | 11.4 | 1 | 1 | 1 | 1.06 | 3.7% |
| P97834 | COP9 signalosome complex subunit 1 | 53.428 | 27.841 | 12.7 | 6 | 6 | 6 | 1.06 | 3.2% |
| Q6AYT5 | UPF0364 protein C6orf211 homolog | 50.183 | 5.9021 | 3.9 | 2 | 2 | 2 | 1.06 | 2.0% |
| B0BN73 | N-acetyltransferase 9 (Predicted) | 27.72 | 7.9565 | 13.3 | 3 | 3 | 3 | 1.06 | 1.7% |
| D3ZC55 | Heat shock 70kDa protein 12A (Predicted), isoform CRA_a | 74.829 | 3.7584 | 5.6 | 3 | 3 | 3 | 1.06 | 1.0% |
| F1M8V2 | Protein Ube4b | 133.3 | 68.38 | 8.3 | 8 | 8 | 8 | 1.06 | 0.9% |
| Q6BBI8 | Ubiquitin-fold modifier-conjugating enzyme 1 | 19.492 | 5.0496 | 10.2 | 2 | 3 | 2 | 1.06 | 3.4% |
| Q4VSI4 | Ubiquitin carboxyl-terminal hydrolase 7 | 128.43 | 93.864 | 14.5 | 13 | 18 | 13 | 1.06 | 4.1% |
| Q99N27 | Sorting nexin-1 | 59.044 | 100.94 | 22.8 | 11 | 16 | 10 | 1.06 | 4.0% |
| Q9ES54 | Nuclear protein localization protein 4 homolog | 68.055 | 157.52 | 24.2 | 14 | 21 | 14 | 1.06 | 1.1% |
| Q99MZ8 | LIM and SH3 domain protein 1 | 29.97 | 125.41 | 59.3 | 19 | 66 | 19 | 1.06 | 1.0% |
| P05369 | Farnesyl pyrophosphate synthase | 40.829 | 101.24 | 33.4 | 10 | 17 | 10 | 1.06 | 0.2% |
| Q4QQR9 | Protein MEMO1 | 33.679 | 17.653 | 12.5 | 4 | 6 | 4 | 1.06 | 0.2% |
| Q4G017 | Nischarin | 166.5 | 42.088 | 5.1 | 6 | 7 | 6 | 1.06 | 0.7% |
| D3ZEH5 | SID1 transmembrane family member 2 | 94.526 | 7.3478 | 2.8 | 2 | 3 | 2 | 1.06 | 5.6% |
| D3ZGD0 | Protein Gemin5 | 166.06 | 20.151 | 2.1 | 3 | 3 | 3 | 1.06 | 3.2% |
| Q9JJ50 | Hepatocyte growth factor-regulated tyrosine kinase substrate | 86.245 | 53.364 | 16.9 | 15 | 21 | 15 | 1.06 | 3.2% |
| Q62868 | Rho-associated protein kinase 2 | 160.39 | 323.31 | 37.7 | 55 | 86 | 49 | 1.06 | 1.6% |
| Q5U318 | Astrocytic phosphoprotein PEA-15 | 15.04 | 89.269 | 60 | 7 | 20 | 7 | 1.06 | 1.3% |
| Q6AYK8 | Eukaryotic translation initiation factor 3 subunit D | 63.988 | 187.64 | 46.7 | 19 | 33 | 19 | 1.06 | 1.4% |
| A0A0G2JU77 | Protein Eif3k | 24.792 | 66.166 | 34.4 | 6 | 12 | 6 | 1.06 | 6.1% |
| Q01986 | Dual specificity mitogen-activated protein kinase kinase 1 | 43.465 | 92.786 | 28.2 | 10 | 16 | 7 | 1.06 | 0.0% |
| A2RRU1 | Glycogen [starch] synthase, muscle | 84.071 | 59.243 | 20.7 | 10 | 12 | 10 | 1.06 | 1.7% |
| G3V9Q4 | Protein Stk38 | 54.159 | 36.928 | 16.8 | 8 | 9 | 6 | 1.06 | 4.4% |
| A0A0G2QC35 | Ubiquitin carboxyl-terminal hydrolase | 41.076 | 2.2396 | 2.3 | 1 | 1 | 1 | 1.06 | 14.6% |
| Q6PCT9 | Proteasome (Prosome, macropain) 26S subunit, non-ATPase, 6 | 45.598 | 89.295 | 46.8 | 19 | 27 | 19 | 1.06 | 0.1% |
| Q2A121 | Alpha-ketoglutarate-dependent dioxygenase FTO | 57.971 | 7.7832 | 5 | 2 | 2 | 2 | 1.06 | 9.9% |
| D4AEG7 | Protein Tbc1d13 | 46.476 | 18.552 | 11.5 | 4 | 4 | 4 | 1.06 | 9.3% |
| Q5XI37 | 28S ribosomal protein S15, mitochondrial | 29.712 | 5.5809 | 5.8 | 1 | 1 | 1 | 1.06 | 3.2% |
| D3ZKQ4 | Protein Rabl6 | 80.253 | 14.165 | 4.9 | 3 | 4 | 3 | 1.06 | 1.1% |
| P0C2B9 | 28S ribosomal protein L42, mitochondrial | 16.469 | 7.1721 | 16.3 | 2 | 2 | 2 | 1.06 | 3.8% |
| G3V8Z6 | Protein Krit1 | 84.005 | 4.035 | 1.6 | 1 | 2 | 1 | 1.05 | 8.1% |
| F1M4P5 | Protein RGD1563354 | 130.76 | -2 | 0.7 | 1 | 1 | 1 | 1.05 | 7.4% |
| Q66HA5 | Coiled-coil and C2 domain-containing protein 1A | 103.59 | 19.985 | 5.8 | 5 | 5 | 5 | 1.05 | 2.2% |
| P97531 | Cdc42-interacting protein 4 | 62.798 | 90.411 | 20.7 | 9 | 9 | 9 | 1.05 | 0.6% |
| Q63413 | Spliceosome RNA helicase Ddx39b | 49.035 | 108.58 | 34.3 | 16 | 22 | 8 | 1.05 | 0.6% |
| Q3T1I4 | Protein PRRC1 | 46.294 | 67.774 | 22.6 | 7 | 12 | 7 | 1.05 | 1.1% |
| G3V918 | Phosphoribosylglycinamide formyltransferase, isoform CRA_a | 107.58 | 183.99 | 22.1 | 15 | 19 | 15 | 1.05 | 1.3% |
| Q4QQT3 | CUGBP Elav-like family member 1 | 52.205 | 30.119 | 15.8 | 7 | 12 | 7 | 1.05 | 13.7% |
| P54690 | Branched-chain-amino-acid aminotransferase, cytosolic | 46.045 | 70.798 | 25.1 | 9 | 19 | 9 | 1.05 | 0.0% |
| G3V7M0 | Protein Cnot1 | 266.86 | 61.119 | 6 | 12 | 12 | 12 | 1.05 | 1.0% |
| O54921 | Exocyst complex component 2 | 104.03 | 47.39 | 9.6 | 8 | 11 | 8 | 1.05 | 5.7% |
| D3ZSZ6 | Diacylglycerol kinase | 134.31 | 1.9573 | 1 | 1 | 1 | 1 | 1.05 | 4.1% |
| Q66HR2 | Microtubule-associated protein RP/EB family member 1 | 30.004 | 80.226 | 44 | 14 | 27 | 12 | 1.05 | 3.7% |
| P63100 | Calcineurin subunit B type 1 | 19.3 | 60.542 | 42.4 | 6 | 8 | 6 | 1.05 | 0.8% |
| D3Z9H7 | Nuclear factor of activated T-cells, cytoplasmic, calcineurin-dependent 4 | 95.763 | 25.054 | 3.6 | 2 | 2 | 2 | 1.05 | 1.0% |
| A0A0G2K8U5 | Protein Lrrc49 | 85.742 | 5.9739 | 3.7 | 2 | 2 | 2 | 1.05 | 2.5% |
| Q5PQL2 | Cell differentiation protein RCD1 homolog | 33.601 | 8.0066 | 4.7 | 1 | 2 | 1 | 1.05 | 5.6% |
| B5DEL5 | Kelch-like 9 (Drosophila) | 69.37 | 10.35 | 5.5 | 3 | 3 | 3 | 1.05 | 6.2% |
| D3ZYB8 | Protein Tex15 | 341.66 | 2.4557 | 0.6 | 2 | 2 | 2 | 1.05 | 13.0% |
| B0K014 | D-tyrosyl-tRNA(Tyr) deacylase | 23.394 | 11.292 | 7.2 | 1 | 1 | 1 | 1.05 | 6.6% |
| B1WC49 | Api5 protein | 56.784 | 106.34 | 22.8 | 10 | 17 | 10 | 1.05 | 2.6% |
| O35814 | Stress-induced-phosphoprotein 1 | 62.569 | 222.37 | 55.6 | 32 | 54 | 32 | 1.05 | 1.6% |
| B1WBV4 | Pleckstrin homology domain containing, family F (With FYVE domain) member 2 | 27.74 | 37.806 | 16.5 | 2 | 2 | 2 | 1.05 | 0.4% |
| Q6P502 | T-complex protein 1 subunit gamma | 60.646 | 234.57 | 56.5 | 28 | 67 | 28 | 1.05 | 0.5% |
| D3Z9L5 | Protein Wdr11 | 135.78 | 39.155 | 4.5 | 6 | 8 | 6 | 1.05 | 3.4% |
| F1LV18 | Protein Mboat1 | 55.977 | 7.6431 | 2.2 | 1 | 1 | 1 | 1.05 | 16.0% |
| P60901 | Proteasome subunit alpha type-6 | 27.399 | 103.1 | 56.9 | 13 | 35 | 13 | 1.05 | 1.7% |
| D3ZHC4 | Heme binding protein 2 (Predicted), isoform CRA_b | 22.95 | 26.82 | 18.7 | 3 | 4 | 3 | 1.05 | 1.6% |
| Q4KM62 | Palmdelphin | 62.418 | 17.216 | 9.4 | 5 | 5 | 5 | 1.05 | 0.4% |
| Q68FQ0 | T-complex protein 1 subunit epsilon | 59.536 | 246.89 | 66 | 31 | 61 | 31 | 1.05 | 0.4% |
| D4A4P4 | Protein Flad1 | 54.604 | 37.916 | 13.5 | 5 | 5 | 5 | 1.05 | 0.0% |
| B1WC26 | N-acetylneuraminic acid synthase | 40.051 | 79.625 | 28.4 | 8 | 12 | 8 | 1.05 | 0.8% |
| A0A0G2JX01 | Protein Ttc7a | 96.112 | 24.144 | 4.2 | 3 | 3 | 3 | 1.05 | 1.0% |
| Q63009 | Protein arginine N-methyltransferase 1 | 40.522 | 117.61 | 36.8 | 13 | 24 | 13 | 1.05 | 2.9% |
| P17220 | Proteasome subunit alpha type-2 | 25.926 | 136.94 | 47.4 | 10 | 18 | 10 | 1.05 | 5.5% |
| Q5XIJ7 | Calcium binding protein 39-like | 39.091 | 5.1368 | 5 | 2 | 2 | 1 | 1.05 | 6.8% |
| Q08603 | Geranylgeranyl transferase type-2 subunit beta | 36.856 | 7.8356 | 6.6 | 2 | 3 | 2 | 1.05 | 3.1% |
| P18422 | Proteasome subunit alpha type-3 | 28.419 | 52.158 | 38.4 | 9 | 22 | 9 | 1.05 | 1.7% |
| P46462 | Transitional endoplasmic reticulum ATPase | 89.348 | 323.31 | 65.9 | 45 | 150 | 45 | 1.05 | 1.7% |
| D3ZA31 | Myotubularin related protein 2 (Predicted), isoform CRA_b | 73.271 | 12.741 | 7.6 | 4 | 4 | 4 | 1.05 | 0.6% |
| B0BMX3 | Protein S100a16 | 14.224 | 32.279 | 22 | 2 | 3 | 2 | 1.05 | 0.4% |
| D4A8A0 | DNA fragmentation factor subunit beta | 243.37 | 79.897 | 8.5 | 14 | 16 | 14 | 1.05 | 0.4% |
| P80254 | D-dopachrome decarboxylase | 13.133 | 69.507 | 69.5 | 8 | 18 | 8 | 1.05 | 0.9% |
| P50475 | Alanine--tRNA ligase, cytoplasmic | 106.79 | 268.88 | 38.3 | 31 | 67 | 31 | 1.05 | 2.3% |
| Q6MGB8 | Protein RT1-A | 41.393 | 42.161 | 18.3 | 7 | 9 | 2 | 1.05 | 2.9% |
| Q99P39 | Cysteine desulfurase, mitochondrial | 50.012 | 76.865 | 25.7 | 9 | 12 | 9 | 1.05 | 9.2% |
| P0C0R5 | Phosphoinositide 3-kinase regulatory subunit 4 | 152.44 | 49.699 | 7.7 | 11 | 13 | 11 | 1.05 | 9.6% |
| P23457 | 3-alpha-hydroxysteroid dehydrogenase | 37.027 | 21.484 | 18.3 | 4 | 6 | 4 | 1.05 | 3.9% |
| Q3MIE7 | COMM domain containing 9 | 21.974 | 81.028 | 51 | 7 | 8 | 7 | 1.05 | 1.6% |
| Q9WVC0 | Septin-7 | 50.507 | 323.31 | 53.9 | 29 | 81 | 27 | 1.05 | 1.0% |
| P19468 | Glutamate--cysteine ligase catalytic subunit | 72.618 | 81.897 | 17.3 | 11 | 16 | 11 | 1.05 | 0.3% |
| Q66H80 | Coatomer subunit delta | 57.199 | 178.72 | 47 | 26 | 56 | 26 | 1.05 | 0.0% |
| P60123 | RuvB-like 1 | 50.213 | 134.95 | 30.7 | 10 | 15 | 10 | 1.05 | 0.7% |
| Q5PPI8 | Protein RGD1311805 | 74.877 | 49.244 | 13.1 | 7 | 10 | 7 | 1.05 | 1.7% |
| Q63433 | Serine/threonine-protein kinase N1 | 104.47 | 26.036 | 7.1 | 6 | 7 | 5 | 1.05 | 3.3% |
| Q53AQ4 | Transmembrane and ubiquitin-like domain-containing protein 1 | 26.349 | 1.9989 | 4.1 | 1 | 1 | 1 | 1.05 | 2.8% |
| P63329 | Serine/threonine-protein phosphatase 2B catalytic subunit alpha isoform | 58.643 | 39.902 | 20.2 | 9 | 13 | 5 | 1.05 | 1.8% |
| G3V6W6 | Protein Psmc6 | 45.796 | 174.65 | 50.4 | 20 | 40 | 20 | 1.05 | 0.9% |
| Q6U6G5 | Zinc finger CCCH domain-containing protein 15 | 48.299 | 50.558 | 17.1 | 6 | 6 | 4 | 1.05 | 1.0% |
| D3ZII8 | Protein Smyd5 | 47.068 | 13.945 | 8.2 | 3 | 3 | 3 | 1.05 | 1.6% |
| D3ZUY0 | Protein Rdh14 | 36.195 | 26.473 | 13.2 | 4 | 4 | 4 | 1.05 | 0.2% |
| B0BNF1 | Septin-8 | 51.251 | 85.82 | 30.1 | 12 | 23 | 8 | 1.05 | 2.9% |
| Q5XIC4 | Maspardin | 29.576 | 37.255 | 18.8 | 5 | 6 | 5 | 1.05 | 1.9% |
| P61751 | ADP-ribosylation factor 4 | 20.396 | 31.087 | 54.4 | 7 | 26 | 3 | 1.05 | 1.6% |
| Q9Z1I6 | Rho guanine nucleotide exchange factor 1 | 102.6 | 136.47 | 26.1 | 24 | 34 | 24 | 1.05 | 1.2% |
| Q6TEK3 | Vitamin K epoxide reductase complex subunit 1-like protein 1 | 19.778 | 3.8426 | 5.7 | 1 | 2 | 1 | 1.05 | 1.5% |
| D3ZIV8 | Protein Ythdc2 | 142.33 | 21.116 | 5.6 | 6 | 6 | 6 | 1.05 | 5.4% |
| F1M208 | Piezo-type mechanosensitive ion channel component | 313.93 | -2 | 0.3 | 1 | 2 | 1 | 1.05 | 1.7% |
| P61928 | 60S ribosomal protein L37 | 11.078 | 8.3527 | 33 | 4 | 9 | 4 | 1.05 | 1.3% |
| P82808 | Glutamine--fructose-6-phosphate aminotransferase [isomerizing] 1 | 76.826 | 142.1 | 28 | 15 | 22 | 15 | 1.05 | 0.6% |
| D4A8H3 | Protein Uba6 | 117.93 | 123.17 | 23.6 | 21 | 28 | 21 | 1.05 | 1.1% |
| P70618 | Mitogen-activated protein kinase 14 | 41.321 | 77.293 | 35.3 | 9 | 10 | 9 | 1.05 | 1.9% |
| Q5XIP6 | Flap endonuclease 1 | 42.608 | 4.7077 | 4.5 | 1 | 1 | 1 | 1.05 | 2.4% |
| P34067 | Proteasome subunit beta type-4 | 29.197 | 118.88 | 42.2 | 8 | 18 | 8 | 1.05 | 2.0% |
| D3ZU51 | Protein Rpp30 | 29.485 | 6.0502 | 7.1 | 2 | 3 | 2 | 1.05 | 1.6% |
| Q9JID2 | Guanine nucleotide-binding protein subunit alpha-11 | 42.026 | 34.782 | 24 | 8 | 10 | 6 | 1.05 | 1.0% |
| F1LTR1 | Protein Wdr26 | 58.49 | 78.483 | 18.3 | 7 | 7 | 7 | 1.05 | 3.5% |
| A0A0G2JUU7 | Protein Tubgcp3 | 103.09 | 43.686 | 9.4 | 6 | 6 | 6 | 1.05 | 5.1% |
| M0RA08 | Perilipin | 47.343 | 72.014 | 31.1 | 11 | 19 | 11 | 1.05 | 6.3% |
| Q4KM35 | Proteasome subunit beta type-10 | 29.038 | 23.972 | 12.5 | 2 | 2 | 2 | 1.05 | 9.6% |
| P62914 | 60S ribosomal protein L11 | 20.252 | 60.056 | 55.1 | 10 | 23 | 10 | 1.05 | 4.3% |
| Q5FVK6 | Coiled-coil and C2 domain-containing protein 1B | 93.533 | 84.864 | 15.1 | 9 | 12 | 9 | 1.05 | 3.2% |
| P18395 | Cold shock domain-containing protein E1 | 88.894 | 114.77 | 30.1 | 25 | 29 | 25 | 1.05 | 2.9% |
| F1M471 | Protein Epm2aip1 | 70.129 | 20.359 | 6.1 | 3 | 4 | 3 | 1.05 | 1.8% |
| Q99PD6 | Transforming growth factor beta-1-induced transcript 1 protein | 50.122 | 204.22 | 56.6 | 20 | 41 | 20 | 1.05 | 1.2% |
| P47860 | ATP-dependent 6-phosphofructokinase, platelet type | 85.719 | 182.15 | 29.7 | 18 | 27 | 15 | 1.05 | 1.0% |
| Q62818 | Translation initiation factor eIF-2B subunit beta | 38.875 | 7.2355 | 5.1 | 1 | 2 | 1 | 1.05 | 0.8% |
| D4A8F2 | Protein Rsu1 | 31.337 | 91.384 | 44.4 | 12 | 29 | 12 | 1.05 | 0.4% |
| A0A0G2JZQ1 | Protein Ddx19a | 55.322 | 95.032 | 26.2 | 11 | 16 | 11 | 1.05 | 0.1% |
| D4A631 | Brefeldin A-inhibited guanine nucleotide-exchange protein 1 | 207.89 | 76.192 | 8.8 | 14 | 17 | 7 | 1.05 | 0.1% |
| F1M8A5 | Protein Hypk | 14.661 | 22.582 | 31.8 | 3 | 5 | 3 | 1.05 | 0.2% |
| P55161 | Nck-associated protein 1 | 128.86 | 128.32 | 16.1 | 16 | 30 | 16 | 1.05 | 0.9% |
| F1LVZ9 | Protein Hectd3 | 97.272 | 45.384 | 11.3 | 7 | 8 | 7 | 1.05 | 1.3% |
| D3ZVR7 | Prostamide/prostaglandin F synthase | 21.607 | 15.123 | 5.5 | 1 | 2 | 1 | 1.05 | 3.4% |
| A0A0G2JUA7 | Protein Nck1 | 42.368 | 21.672 | 15.8 | 6 | 9 | 6 | 1.05 | 5.4% |
| P68907 | E3 ubiquitin-protein ligase PDZRN3 | 119.29 | 11.228 | 3.5 | 4 | 4 | 4 | 1.05 | 3.7% |
| Q63186 | Translation initiation factor eIF-2B subunit delta | 57.809 | 72.559 | 18.7 | 6 | 7 | 6 | 1.05 | 3.1% |
| Q62728 | Tyrosine-protein phosphatase non-receptor type 21 | 133.41 | 35.034 | 4.4 | 5 | 6 | 5 | 1.05 | 3.0% |
| Q5XIM9 | T-complex protein 1 subunit beta | 57.458 | 323.31 | 61.3 | 32 | 72 | 32 | 1.05 | 2.6% |
| Q66HA8 | Heat shock protein 105 kDa | 96.417 | 203.73 | 38 | 28 | 50 | 25 | 1.05 | 0.1% |
| B5DFI3 | Adaptor protein complex AP-1, sigma 1 (Predicted), isoform CRA_b | 18.733 | 24.935 | 14.6 | 2 | 3 | 2 | 1.05 | 0.5% |
| B2GV22 | Phosphatidylserine synthase 2 | 54.722 | 3.1529 | 4.9 | 2 | 3 | 2 | 1.05 | 27.4% |
| F1LNL3 | Protein Abca1 | 253.99 | 2.0081 | 0.7 | 2 | 2 | 2 | 1.05 | 5.0% |
| Q9R0I8 | Phosphatidylinositol 5-phosphate 4-kinase type-2 alpha | 46.209 | 51.616 | 25.6 | 10 | 16 | 5 | 1.05 | 2.9% |
| F1M6V0 | Protein Nbeal1 | 306.62 | 30.093 | 2.8 | 6 | 6 | 6 | 1.05 | 3.7% |
| P12369 | cAMP-dependent protein kinase type II-beta regulatory subunit | 46.122 | 117.57 | 37.5 | 13 | 27 | 11 | 1.05 | 3.7% |
| Q6AY97 | Coiled-coil domain-containing protein 91 | 50.165 | 46.341 | 18.6 | 8 | 9 | 8 | 1.05 | 3.7% |
| P52164 | Protein max | 18.272 | 7.4307 | 19.4 | 3 | 3 | 3 | 1.05 | 5.6% |
| Q91V26 | Sphingosine kinase 1 | 42.418 | 4.0048 | 5.7 | 2 | 2 | 2 | 1.05 | 4.6% |
| Q6X936 | Kin of IRRE-like protein 1 | 87.24 | 20.796 | 4.6 | 2 | 3 | 2 | 1.05 | 3.9% |
| Q6AYS7 | Aminoacylase-1A | 45.804 | 43.661 | 22.1 | 7 | 8 | 7 | 1.05 | 0.0% |
| B0LT89 | Serine/threonine-protein kinase 24 | 47.989 | 38.283 | 23.2 | 8 | 9 | 5 | 1.05 | 0.5% |
| Q5RKH2 | Galactokinase 1 | 42.376 | 65.072 | 33.9 | 12 | 17 | 12 | 1.05 | 1.0% |
| M0R9L3 | Protein Snx9 | 66.023 | 60.903 | 17.5 | 9 | 13 | 9 | 1.05 | 2.6% |
| Q5U2Z7 | Rho GTPase-activating protein 24 | 84.143 | 11.787 | 4.5 | 3 | 4 | 3 | 1.05 | 3.4% |
| Q5XI55 | Peptide-N(4)-(N-acetyl-beta-glucosaminyl)asparagine amidase | 74.676 | 6.5325 | 2.9 | 3 | 3 | 3 | 1.05 | 2.2% |
| B4F7E8 | Niban-like protein 1 | 84.73 | 235.35 | 38 | 21 | 38 | 21 | 1.05 | 1.6% |
| P08010 | Glutathione S-transferase Mu 2 | 25.702 | 142.63 | 77.1 | 19 | 46 | 12 | 1.05 | 1.1% |
| Q5BJW9 | DnaJ homolog subfamily C member 25 | 42.107 | 10.855 | 9 | 3 | 4 | 3 | 1.05 | 6.0% |
| Q9ET64 | Sphingomyelin phosphodiesterase 2 | 47.644 | 4.302 | 2.6 | 1 | 1 | 1 | 1.05 | 0.8% |
| M0R484 | Protein Afap1l2 | 93.001 | 145.17 | 32.1 | 23 | 31 | 23 | 1.05 | 4.1% |
| B4F7C7 | Hebp1 protein | 21.124 | 51.164 | 39.5 | 6 | 8 | 6 | 1.05 | 2.0% |
| Q6P9X4 | Protein tyrosine phosphatase type IVA 2 | 19.127 | 17.541 | 17.4 | 3 | 4 | 3 | 1.05 | 1.9% |
| Q66X93 | Staphylococcal nuclease domain-containing protein 1 | 101.95 | 323.31 | 46.8 | 40 | 77 | 40 | 1.05 | 1.3% |
| P43138 | DNA-(apurinic or apyrimidinic site) lyase | 35.538 | 93.635 | 39.4 | 8 | 15 | 8 | 1.05 | 1.5% |
| Q91Y81 | Septin-2 | 41.592 | 313.76 | 54.8 | 15 | 57 | 15 | 1.05 | 1.5% |
| D4A6W6 | 60S ribosomal protein L8 | 28.156 | 112.67 | 47.5 | 11 | 24 | 11 | 1.05 | 2.2% |
| P35559 | Insulin-degrading enzyme | 117.71 | 79.906 | 15.4 | 14 | 21 | 14 | 1.05 | 3.3% |
| F1LQP9 | Protein Tnpo1 | 101.27 | 91.975 | 16.6 | 13 | 19 | 9 | 1.05 | 7.8% |
| P84850 | D-2-hydroxyglutarate dehydrogenase, mitochondrial | 58.804 | 4.8535 | 6.2 | 2 | 2 | 2 | 1.05 | 12.9% |
| Q99JC6 | Protein Tapbp | 50.044 | 17.495 | 4.3 | 2 | 4 | 2 | 1.05 | 7.2% |
| A0A0G2K9M5 | Protein Tbc1d8b | 128.1 | 27.831 | 4.3 | 5 | 5 | 5 | 1.05 | 3.6% |
| P15178 | Aspartate--tRNA ligase, cytoplasmic | 57.126 | 149.49 | 49.9 | 23 | 37 | 23 | 1.05 | 3.3% |
| A0A0G2JV19 | Protein Tgfbrap1 | 97.345 | 7.7703 | 2.4 | 2 | 2 | 2 | 1.05 | 2.1% |
| D4A3I4 | Transcription factor BTF3 | 17.27 | 11.973 | 15.2 | 2 | 2 | 2 | 1.05 | 1.9% |
| F7F5J1 | Microphthalmia-associated transcription factor | 52.748 | 1.8932 | 1.7 | 1 | 1 | 1 | 1.05 | 1.1% |
| B5DF44 | Kctd15 protein | 31.9 | 8.7725 | 7.4 | 2 | 2 | 2 | 1.05 | 14.7% |
| A0A0G2JSL0 | Proteasome subunit beta type-6 | 25.303 | 63.891 | 32.8 | 8 | 13 | 8 | 1.05 | 1.6% |
| A0A0G2JUG6 | Protein Lrrc45 | 83.152 | 3.9636 | 4.4 | 3 | 3 | 3 | 1.05 | 9.6% |
| O88763 | Phosphatidylinositol 3-kinase catalytic subunit type 3 | 101.53 | 25.724 | 6.7 | 5 | 6 | 5 | 1.05 | 0.7% |
| Q496Z9 | TRMT1-like protein | 80.183 | 14.441 | 4.1 | 2 | 3 | 2 | 1.05 | 3.9% |
| A0A096MIV5 | Protein Abcf2 | 71.655 | 46.302 | 13.9 | 8 | 15 | 8 | 1.05 | 3.2% |
| F1LN91 | Protein Tns3 | 164.79 | 17.275 | 5.2 | 8 | 11 | 5 | 1.05 | 2.1% |
| D3ZVR9 | Protein Pgm5 | 62.204 | 14.514 | 9.5 | 5 | 6 | 5 | 1.05 | 1.2% |
| P30919 | N(4)-(Beta-N-acetylglucosaminyl)-L-asparaginase | 37.167 | 52.808 | 21.2 | 5 | 11 | 5 | 1.05 | 1.1% |
| Q5FWY5 | AH receptor-interacting protein | 37.598 | 48.388 | 29.1 | 8 | 12 | 8 | 1.05 | 0.5% |
| D3ZG24 | Protein Zswim8 | 189.3 | 7.579 | 1.5 | 2 | 2 | 2 | 1.05 | 0.1% |
| F1LMZ8 | 26S proteasome non-ATPase regulatory subunit 11 | 47.463 | 112.2 | 35.8 | 15 | 26 | 15 | 1.05 | 0.6% |
| O09175 | Aminopeptidase B | 72.619 | 112.08 | 24.2 | 12 | 17 | 12 | 1.05 | 1.1% |
| P53042 | Serine/threonine-protein phosphatase 5 | 56.916 | 68.559 | 28.7 | 13 | 18 | 13 | 1.05 | 2.0% |
| Q7TMZ5 | ADP-ribosylation factor-like 6 interacting protein 1 | 23.451 | 7.9239 | 8.9 | 2 | 3 | 2 | 1.05 | 7.8% |
| B4F7E7 | Membrane protein, palmitoylated 5 (MAGUK p55 subfamily member 5) | 77.152 | 10.443 | 1.8 | 1 | 3 | 1 | 1.04 | 6.7% |
| O88637 | Ethanolamine-phosphate cytidylyltransferase | 45.219 | 7.2292 | 9.2 | 3 | 3 | 3 | 1.04 | 6.3% |
| Q4KLF8 | Actin-related protein 2/3 complex subunit 5 | 16.32 | 63.664 | 66.9 | 7 | 15 | 7 | 1.04 | 3.2% |
| F1M1B3 | Protein RGD1564420 | 134.21 | 97.481 | 17.1 | 16 | 18 | 16 | 1.04 | 0.3% |
| Q4G061 | Eukaryotic translation initiation factor 3 subunit B | 90.91 | 205.33 | 32.1 | 22 | 34 | 22 | 1.04 | 1.1% |
| E9PT79 | Protein Tsn | 31.473 | 97.475 | 36 | 8 | 15 | 8 | 1.04 | 2.7% |
| A0A0G2K0J7 | Protein Ctdp1 | 113.33 | 9.9138 | 2.1 | 2 | 3 | 2 | 1.04 | 3.9% |
| P09760 | Tyrosine-protein kinase Fer | 94.313 | 43.723 | 6.7 | 4 | 4 | 4 | 1.04 | 6.9% |
| D3ZR52 | Protein Lpcat4 | 57.136 | 7.3522 | 2.5 | 1 | 1 | 1 | 1.04 | 7.5% |
| Q6AYR6 | Haloacid dehalogenase-like hydrolase domain-containing protein 2 | 28.779 | 19.656 | 13.9 | 3 | 3 | 3 | 1.04 | 10.8% |
| F1LMQ3 | Protein Psmd8 | 39.877 | 56.575 | 19.8 | 6 | 10 | 6 | 1.04 | 11.1% |
| Q9R037 | WD repeat-containing protein 44 | 100.95 | 52.438 | 6.8 | 5 | 6 | 5 | 1.04 | 4.5% |
| B3GNI6 | Septin-11 | 49.694 | 186.28 | 41.3 | 17 | 49 | 13 | 1.04 | 3.0% |
| Q32KK0 | Arylsulfatase E | 65.675 | 37.351 | 8.7 | 4 | 8 | 4 | 1.04 | 2.2% |
| D4AC16 | Protein Tbc1d1 | 142.33 | 27.946 | 4.5 | 5 | 5 | 5 | 1.04 | 1.1% |
| Q6MGC4 | H2-K region expressed gene 2, rat orthologue | 14.497 | 26.664 | 35.4 | 8 | 12 | 8 | 1.04 | 1.3% |
| P52296 | Importin subunit beta-1 | 97.123 | 309.29 | 34.2 | 24 | 48 | 24 | 1.04 | 1.5% |
| F1LT49 | Protein Lrrc47 | 63.53 | 93.263 | 25.3 | 13 | 17 | 13 | 1.04 | 2.2% |
| P19945 | 60S acidic ribosomal protein P0 | 34.215 | 156.88 | 48.3 | 15 | 37 | 15 | 1.04 | 3.0% |
| P01041 | Cystatin-B | 11.196 | 15.385 | 35.7 | 5 | 9 | 5 | 1.04 | 4.3% |
| F1LS35 | Hermansky-Pudlak syndrome 5 protein | 126.28 | 5.6172 | 1 | 1 | 3 | 1 | 1.04 | 4.9% |
| F1LZB7 | Protein Frmd4b | 117.98 | 10.803 | 1.3 | 1 | 1 | 1 | 1.04 | 9.2% |
| Q9Z1A5 | NEDD8-activating enzyme E1 regulatory subunit | 60.382 | 98.593 | 15.4 | 5 | 6 | 5 | 1.04 | 6.2% |
| Q641Z6 | EH domain-containing protein 1 | 60.602 | 56.021 | 29.6 | 15 | 23 | 6 | 1.04 | 9.0% |
| D3ZZN9 | Protein Syde1 | 80.377 | 13.095 | 7.3 | 5 | 5 | 5 | 1.04 | 5.7% |
| Q80X08 | WASH complex subunit FAM21 | 145.15 | 264.11 | 25.8 | 22 | 28 | 22 | 1.04 | 3.4% |
| Q2PQA9 | Kinesin-1 heavy chain | 109.53 | 323.31 | 62.4 | 52 | 96 | 41 | 1.04 | 0.9% |
| Q5XHY0 | DEAD (Asp-Glu-Ala-Asp) box polypeptide 18 | 75.547 | 21.339 | 9.2 | 5 | 5 | 5 | 1.04 | 0.2% |
| D3ZKH6 | Protein Rabgap1l | 92.262 | 21.941 | 3.6 | 2 | 2 | 2 | 1.04 | 1.6% |
| Q9Z244 | GMP reductase 1 | 37.488 | 5.2843 | 11.9 | 4 | 6 | 2 | 1.04 | 3.5% |
| Q8K1P7 | Transcription activator BRG1 | 181.43 | 28.192 | 5.4 | 8 | 10 | 8 | 1.04 | 5.5% |
| E9PU42 | Down syndrome critical region gene 3 (Predicted), isoform CRA_c | 32.936 | 55.274 | 24.2 | 5 | 7 | 5 | 1.04 | 10.2% |
| Q9QYU1 | Peroxisomal biogenesis factor 19 | 32.497 | 64.237 | 33.8 | 8 | 10 | 8 | 1.04 | 1.1% |
| P15205 | Microtubule-associated protein 1B | 269.5 | 323.31 | 23.2 | 46 | 77 | 44 | 1.04 | 0.3% |
| Q63945 | Protein SET | 33.405 | 59.083 | 26.3 | 8 | 17 | 8 | 1.04 | 0.7% |
| Q5PPK9 | Protein TSSC1 | 43.156 | 2.9009 | 2.3 | 1 | 1 | 1 | 1.04 | 6.6% |
| Q9EPF2 | Cell surface glycoprotein MUC18 | 71.326 | 72.086 | 21.5 | 13 | 21 | 13 | 1.04 | 6.4% |
| O70277 | Tripartite motif-containing protein 3 | 80.795 | 38.456 | 7 | 4 | 4 | 4 | 1.04 | 2.3% |
| D4A7R0 | Signal recognition particle subunit SRP72 | 74.651 | 112.14 | 29.4 | 18 | 27 | 18 | 1.04 | 1.6% |
| Q5M9F7 | ARP10 actin-related protein 10 homolog (S. cerevisiae) | 46.213 | 127.78 | 30.2 | 10 | 14 | 10 | 1.04 | 4.0% |
| F1M648 |  | 38.701 | 23.432 | 8.5 | 2 | 3 | 1 | 1.04 | 12.4% |
| Q9R0Z7 | Alpha- and gamma-adaptin-binding protein p34 | 34.363 | 15.44 | 6.3 | 1 | 1 | 1 | 1.04 | 11.0% |
| P85108 | Tubulin beta-2A chain | 49.906 | 123.42 | 64.5 | 25 | 149 | 6 | 1.04 | 1.3% |
| F1LRI5 | Protein Gcn1l1 | 293.07 | 323.31 | 28.3 | 62 | 86 | 62 | 1.04 | 2.5% |
| Q5XHY7 | Signal transducing adapter molecule 2 | 57.159 | 36.284 | 12.8 | 6 | 7 | 5 | 1.04 | 4.7% |
| F1LX81 | Protein Slfn5 | 101.13 | 12.127 | 5 | 4 | 5 | 4 | 1.04 | 5.9% |
| Q66HG9 | Mitochondrial antiviral-signaling protein | 53.804 | 67.911 | 19.7 | 6 | 8 | 6 | 1.04 | 9.4% |
| P52631 | Signal transducer and activator of transcription 3 | 88.039 | 111.47 | 24.2 | 14 | 18 | 14 | 1.04 | 0.5% |
| Q8VD52 | Pyridoxal phosphate phosphatase | 33.114 | 2.6049 | 2.6 | 1 | 1 | 1 | 1.04 | 1.4% |
| D3ZT01 | Protein Cog2 | 81.804 | 64.488 | 14.2 | 7 | 10 | 7 | 1.04 | 2.2% |
| Q68FS1 | Cytosolic Fe-S cluster assembly factor NUBP2 | 28.926 | 8.8305 | 6.6 | 1 | 2 | 1 | 1.04 | 2.6% |
| P84100 | 60S ribosomal protein L19 | 23.466 | 33.466 | 34.7 | 9 | 25 | 8 | 1.04 | 0.3% |
| O35112 | CD166 antigen | 65.021 | 48.012 | 13.4 | 5 | 7 | 5 | 1.04 | 0.5% |
| B5DFN4 | Prefoldin 5 (Predicted), isoform CRA_a | 17.326 | 100.12 | 71.4 | 9 | 20 | 9 | 1.04 | 0.7% |
| D3ZHQ1 | Dipeptidylpeptidase 8 (Predicted), isoform CRA_a | 102.17 | 28.303 | 5.8 | 5 | 6 | 5 | 1.04 | 1.0% |
| D3ZY02 | Protein Athl1 | 76.953 | 18.984 | 3.6 | 2 | 2 | 2 | 1.04 | 17.4% |
| B2RYI5 | Ankrd40 protein | 40.489 | 9.3986 | 9.4 | 2 | 2 | 2 | 1.04 | 4.9% |
| P40112 | Proteasome subunit beta type-3 | 22.965 | 80.176 | 43.4 | 9 | 19 | 9 | 1.04 | 3.5% |
| D4A1B8 | Protein Dctn3 | 21.112 | 32.215 | 36.6 | 8 | 9 | 8 | 1.04 | 2.2% |
| D3ZY58 | Protein Unc119b | 28.492 | 8.3857 | 15.9 | 3 | 3 | 3 | 1.04 | 0.5% |
| Q99JD4 | CLIP-associating protein 2 | 140.64 | 14.856 | 3.7 | 5 | 5 | 4 | 1.04 | 0.1% |
| Q99MC0 | Protein phosphatase 1 regulatory subunit 14A | 16.697 | 27.786 | 43.5 | 4 | 5 | 4 | 1.04 | 2.4% |
| P59215 | Guanine nucleotide-binding protein G(o) subunit alpha | 40.068 | 11.341 | 10.7 | 3 | 7 | 1 | 1.04 | 8.9% |
| P50411 | Protein phosphatase inhibitor 2 | 23.071 | 30.557 | 22.4 | 4 | 4 | 4 | 1.04 | 6.5% |
| D4A7G9 | Protein RGD1564804 | 21.903 | 49.592 | 19.6 | 3 | 6 | 3 | 1.04 | 5.9% |
| D3ZKZ4 | Bromodomain adjacent to zinc finger domain, 2B (Predicted) | 241.31 | 2.6747 | 0.7 | 2 | 3 | 2 | 1.04 | 3.9% |
| D4A3V3 | Poly (ADP-ribose) polymerase family, member 12 (Predicted), isoform CRA_b | 79.485 | 19.298 | 7.1 | 4 | 4 | 4 | 1.04 | 3.1% |
| P49242 | 40S ribosomal protein S3a | 29.945 | 158.78 | 68.2 | 23 | 48 | 23 | 1.04 | 0.4% |
| Q6IMA8 | ADP-ribosylation factor-like 10 | 21.632 | 1.9364 | 3.6 | 1 | 1 | 1 | 1.04 | 1.2% |
| Q63644 | Rho-associated protein kinase 1 | 159.62 | 119.94 | 21.8 | 28 | 39 | 22 | 1.04 | 1.6% |
| F1LR42 | Protein Rufy1 | 80.376 | 26.965 | 8.4 | 5 | 5 | 5 | 1.04 | 3.8% |
| P49791 | Nuclear pore complex protein Nup153 | 152.82 | 75.912 | 7.7 | 8 | 10 | 8 | 1.04 | 4.1% |
| P62832 | 60S ribosomal protein L23 | 14.865 | 80.055 | 70.7 | 9 | 24 | 9 | 1.04 | 2.0% |
| P62755 | 40S ribosomal protein S6 | 28.68 | 77.503 | 36.9 | 13 | 33 | 13 | 1.04 | 0.1% |
| Q6QI16 | LRRGT00192 | 33.824 | 113.56 | 42.9 | 13 | 20 | 13 | 1.04 | 0.0% |
| P09215 | Protein kinase C delta type | 77.519 | 36.526 | 10.5 | 8 | 9 | 8 | 1.04 | 0.1% |
| P49088 | Asparagine synthetase [glutamine-hydrolyzing] | 64.246 | 136.68 | 40.1 | 19 | 50 | 19 | 1.04 | 0.5% |
| P53676 | AP-3 complex subunit mu-1 | 46.981 | 55.84 | 20.1 | 7 | 9 | 6 | 1.04 | 0.6% |
| P62982 | Ubiquitin-40S ribosomal protein S27a | 17.951 | 98.251 | 64.7 | 12 | 48 | 4 | 1.04 | 0.6% |
| P16970 | ATP-binding cassette sub-family D member 3 | 75.315 | 22.641 | 9.6 | 7 | 8 | 7 | 1.04 | 3.6% |
| Q6AXY8 | Dehydrogenase/reductase (SDR family) member 1 | 34.015 | 45.801 | 24 | 6 | 6 | 6 | 1.04 | 4.3% |
| Q5PQN6 | Spermatogenesis-defective protein 39 homolog | 53.304 | 21.855 | 12 | 4 | 6 | 4 | 1.04 | 5.5% |
| D3ZAW2 | Protein Pisd | 45.823 | 4.4497 | 4.4 | 2 | 2 | 2 | 1.04 | 7.5% |
| A0A0G2K189 | Protein Scrn3 | 47.769 | 26.749 | 10.8 | 3 | 4 | 3 | 1.04 | 5.8% |
| F1LQI6 | Protein Zer1 | 89.094 | 3.874 | 2.1 | 1 | 1 | 1 | 1.04 | 3.0% |
| P97521 | Mitochondrial carnitine/acylcarnitine carrier protein | 33.154 | 41.169 | 34.9 | 9 | 12 | 9 | 1.04 | 0.2% |
| P10760 | Adenosylhomocysteinase | 47.538 | 86.739 | 38 | 14 | 19 | 14 | 1.04 | 2.8% |
| D3ZFU9 | Protein Mylk | 214.91 | 217.52 | 15.6 | 29 | 45 | 29 | 1.04 | 1.3% |
| A0JPM9 | Eukaryotic translation initiation factor 3 subunit J | 29.187 | 66.156 | 37.5 | 11 | 19 | 11 | 1.04 | 0.5% |
| Q5RKI5 | Flightless I homolog (Drosophila) | 144.86 | 264.61 | 26.1 | 26 | 34 | 26 | 1.04 | 0.1% |
| A0A0G2K950 | Protein Papss2 | 69.95 | 102.87 | 24.2 | 13 | 17 | 13 | 1.04 | 1.8% |
| Q9JMI1 | Acetoacetyl-CoA synthetase | 75.039 | 8.5158 | 4.2 | 3 | 3 | 3 | 1.04 | 3.1% |
| Q7TT49 | Serine/threonine-protein kinase MRCK beta | 194.89 | 103.22 | 15.8 | 27 | 31 | 23 | 1.04 | 2.1% |
| Q6PDV7 | 60S ribosomal protein L10 | 24.604 | 106.98 | 50.5 | 12 | 37 | 12 | 1.04 | 0.6% |
| Q9QZH7 | F-box/LRR-repeat protein 20 | 30.46 | 5.6986 | 5.1 | 1 | 1 | 1 | 1.04 | 8.6% |
| P34064 | Proteasome subunit alpha type-5 | 26.391 | 106.26 | 51.5 | 8 | 21 | 8 | 1.04 | 4.9% |
| M0R5V1 | Protein Ppm1l | 25.87 | 2.8636 | 3.9 | 1 | 2 | 1 | 1.04 | 3.0% |
| E9PU28 | Inosine-5-monophosphate dehydrogenase 2 | 55.814 | 98.236 | 20.6 | 10 | 14 | 8 | 1.04 | 2.7% |
| O88900 | Growth factor receptor-bound protein 14 | 60.592 | 78.365 | 23.6 | 9 | 11 | 9 | 1.04 | 1.3% |
| P21913 | Succinate dehydrogenase [ubiquinone] iron-sulfur subunit, mitochondrial | 31.83 | 64.543 | 41.1 | 10 | 12 | 10 | 1.04 | 0.1% |
| M0R618 | Protein Etl4 | 216.7 | 32.863 | 6.8 | 13 | 16 | 13 | 1.04 | 0.7% |
| Q9WUH4 | Four and a half LIM domains protein 1 | 31.904 | 161.66 | 59.6 | 17 | 36 | 17 | 1.04 | 1.4% |
| Q5XIN3 | TRAF3-interacting protein 1 | 74.113 | 5.6468 | 2.9 | 2 | 2 | 2 | 1.04 | 2.2% |
| Q4V888 | Type 2 phosphatidylinositol 4,5-bisphosphate 4-phosphatase | 28.024 | 34.562 | 24.5 | 5 | 8 | 4 | 1.04 | 2.6% |
| A0A0G2JY22 | Protein Dip2b | 170.92 | 11.289 | 2.7 | 3 | 4 | 3 | 1.04 | 3.7% |
| P22985 | Xanthine dehydrogenase/oxidase | 146.24 | 9.7273 | 2.6 | 3 | 3 | 2 | 1.04 | 7.8% |
| O08658 | Nuclear pore complex protein Nup88 | 83.584 | 17.474 | 5.8 | 4 | 5 | 4 | 1.04 | 4.5% |
| P04642 | L-lactate dehydrogenase A chain | 36.45 | 120.76 | 41 | 13 | 32 | 12 | 1.04 | 1.0% |
| D4ACK1 | Protein Nup214 | 181.12 | 69.738 | 6.3 | 9 | 10 | 9 | 1.04 | 4.3% |
| Q5XIC8 | Alpha-ketoglutarate-dependent dioxygenase alkB homolog 3 | 34.011 | 5.0308 | 3.7 | 1 | 2 | 1 | 1.04 | 3.6% |
| P70473 | Alpha-methylacyl-CoA racemase | 41.828 | 25.8 | 13.4 | 4 | 4 | 4 | 1.04 | 3.0% |
| Q5XIU5 | Proteasome inhibitor PI31 subunit | 29.854 | 33.106 | 18.5 | 4 | 6 | 4 | 1.04 | 2.8% |
| Q99MI7 | NEDD8-activating enzyme E1 catalytic subunit | 51.723 | 78.581 | 20.6 | 7 | 12 | 7 | 1.04 | 1.8% |
| P18266 | Glycogen synthase kinase-3 beta | 46.742 | 116.32 | 33.6 | 8 | 13 | 8 | 1.04 | 0.5% |
| P47196 | RAC-alpha serine/threonine-protein kinase | 55.735 | 52.187 | 26.9 | 11 | 12 | 8 | 1.04 | 2.2% |
| Q505J8 | Phenylalanine--tRNA ligase alpha subunit | 57.719 | 83.854 | 26.4 | 12 | 22 | 12 | 1.04 | 3.0% |
| D3ZFK5 | Protein Spcs1 | 18.1 | 4.7757 | 8 | 1 | 1 | 1 | 1.04 | 6.2% |
| D4A471 | Protein Cmc2 | 9.4086 | 4.9717 | 12.7 | 1 | 2 | 1 | 1.04 | 4.9% |
| D4A1D2 | Protein Arhgef26 | 97.307 | 8.5232 | 3.7 | 3 | 3 | 3 | 1.04 | 4.7% |
| Q6AY86 | Vacuolar protein sorting-associated protein 26A | 38.114 | 32.933 | 23.9 | 6 | 9 | 6 | 1.04 | 3.5% |
| D3ZW55 | Inosine triphosphate pyrophosphatase | 21.927 | 35.602 | 31.3 | 4 | 8 | 4 | 1.04 | 1.6% |
| P61212 | ADP-ribosylation factor-like protein 1 | 20.411 | 68.496 | 33.7 | 5 | 14 | 5 | 1.04 | 1.5% |
| Q6AYT3 | tRNA-splicing ligase RtcB homolog | 55.249 | 181.37 | 45.7 | 21 | 36 | 21 | 1.04 | 1.1% |
| P51583 | Multifunctional protein ADE2 | 47.096 | 86.795 | 38.6 | 14 | 17 | 14 | 1.04 | 0.3% |
| Q9QYP0 | Multiple epidermal growth factor-like domains protein 8 | 297.55 | 2.5918 | 0.9 | 2 | 2 | 2 | 1.04 | 2.9% |
| B2RYJ1 | Anapc2 protein | 95.372 | 7.0114 | 3.7 | 3 | 3 | 3 | 1.04 | 10.7% |
| Q91XJ1 | Beclin-1 | 51.556 | 7.3319 | 6.5 | 3 | 3 | 3 | 1.04 | 8.8% |
| Q704E8 | ATP-binding cassette sub-family B member 7, mitochondrial | 82.557 | 25.965 | 6.8 | 4 | 5 | 4 | 1.04 | 6.5% |
| F1M446 | Protein AI314180 | 203.92 | 203.46 | 18.3 | 24 | 29 | 24 | 1.04 | 3.9% |
| A0A096MJP9 | Protein Ddi2 | 106.03 | 11.582 | 5.7 | 5 | 5 | 5 | 1.04 | 3.7% |
| P24049 | 60S ribosomal protein L17 | 21.397 | 62.766 | 48.4 | 9 | 18 | 9 | 1.04 | 1.6% |
| P09895 | 60S ribosomal protein L5 | 34.458 | 99.093 | 46.8 | 15 | 51 | 15 | 1.04 | 1.8% |
| P36506 | Dual specificity mitogen-activated protein kinase kinase 2 | 44.281 | 28.648 | 18.5 | 7 | 11 | 4 | 1.04 | 5.0% |
| D3ZSB7 | Ribosomal protein S6 kinase | 85.508 | 7.4641 | 3 | 2 | 2 | 2 | 1.04 | 13.8% |
| B2GV74 | Kinesin light chain 2 (Predicted), isoform CRA_b | 68.61 | 51.003 | 24.9 | 12 | 18 | 7 | 1.04 | 5.5% |
| Q794E4 | Heterogeneous nuclear ribonucleoprotein F | 45.729 | 157.92 | 31.8 | 10 | 20 | 8 | 1.04 | 4.3% |
| Q8R424 | STAM-binding protein | 48.511 | 67.674 | 20.3 | 8 | 10 | 8 | 1.04 | 2.9% |
| Q3SWT5 | Solute carrier family 41 member 3 | 50.526 | 14.492 | 2.8 | 1 | 1 | 1 | 1.04 | 2.0% |
| Q63507 | 60S ribosomal protein L14 | 23.339 | 37.853 | 28.5 | 6 | 15 | 6 | 1.04 | 1.8% |
| Q5U2M6 | DDB1- and CUL4-associated factor 8 | 66.155 | 10.852 | 4.7 | 3 | 3 | 3 | 1.04 | 1.6% |
| O88761 | 26S proteasome non-ATPase regulatory subunit 1 | 105.75 | 250.04 | 34.1 | 26 | 41 | 26 | 1.04 | 1.0% |
| Q63570 | 26S protease regulatory subunit 6B | 47.408 | 201.13 | 48.1 | 18 | 38 | 18 | 1.04 | 0.3% |
| Q5XI39 | Crystallin, zeta (Quinone reductase)-like 1 | 38.783 | 21.121 | 14.7 | 4 | 4 | 4 | 1.04 | 1.4% |
| F7FG68 | Protein LOC100910831 | 67.257 | 3.1501 | 1.3 | 1 | 1 | 1 | 1.04 | 1.7% |
| A1L108 | Actin-related protein 2/3 complex subunit 5-like protein | 17.01 | 72.922 | 37.9 | 5 | 7 | 5 | 1.04 | 2.1% |
| D3ZFJ3 | Protein Sh3bp1 | 74.851 | 83.943 | 13.5 | 7 | 10 | 7 | 1.04 | 3.2% |
| P62859 | 40S ribosomal protein S28 | 7.8409 | 35.093 | 52.2 | 4 | 7 | 4 | 1.04 | 3.1% |
| Q5RJR2 | Twinfilin-1 | 40.09 | 70.186 | 24.6 | 8 | 15 | 8 | 1.04 | 3.0% |
| D4ABC7 | Protein Uprt | 34.242 | 14.552 | 13.9 | 4 | 4 | 4 | 1.04 | 0.1% |
| Q8VIJ5 | Bifunctional protein NCOAT | 102.92 | 105.11 | 14.8 | 11 | 19 | 11 | 1.04 | 0.3% |
| Q6AYE1 | Protein Ccz1b | 32.823 | 19.873 | 9.9 | 2 | 4 | 2 | 1.04 | 3.0% |
| D4AEH3 | Proteasome (Prosome, macropain) 26S subunit, non-ATPase, 7 (Predicted) | 36.491 | 71.631 | 37.8 | 9 | 14 | 9 | 1.04 | 1.3% |
| D4ACB8 | Chaperonin subunit 8 (Theta) (Predicted), isoform CRA_a | 59.588 | 275.45 | 59.7 | 33 | 83 | 33 | 1.04 | 0.2% |
| Q5EGY4 | Synaptobrevin homolog YKT6 | 22.369 | 91.22 | 49 | 10 | 16 | 10 | 1.04 | 0.1% |
| O35787 | Kinesin-like protein KIF1C | 122.33 | 132.13 | 19.4 | 19 | 26 | 15 | 1.04 | 0.2% |
| Q8R3Z7 | EH-domain containing 4 | 61.467 | 138.2 | 45.8 | 21 | 41 | 16 | 1.04 | 0.6% |
| Q3SWS8 | mRNA export factor | 40.919 | 48.143 | 23.1 | 8 | 8 | 8 | 1.04 | 1.3% |
| A0A0G2JZ38 | Protein Fhod1 | 126.47 | 153.49 | 19.9 | 17 | 20 | 17 | 1.04 | 1.4% |
| Q0ZFS8 | Protein LOC100910660 | 19.329 | 21.637 | 28 | 5 | 7 | 4 | 1.04 | 2.1% |
| D3ZL85 | Protein Hccs | 31.129 | 13.022 | 8.8 | 2 | 2 | 2 | 1.04 | 3.4% |
| P49186 | Mitogen-activated protein kinase 9 | 48.016 | 28.056 | 5.7 | 2 | 2 | 2 | 1.04 | 0.1% |
| Q99MI5 | Protein LOC100364487 | 33.997 | 90.514 | 42.1 | 9 | 15 | 9 | 1.04 | 1.1% |
| A0A0G2JU49 | Protein Mtx1 | 51.925 | 57.129 | 13.2 | 5 | 6 | 5 | 1.04 | 2.1% |
| A0A0G2JYJ7 | Protein Rbms2 | 43.609 | 42.223 | 14.8 | 5 | 11 | 3 | 1.04 | 1.5% |
| Q5U2Q7 | Eukaryotic peptide chain release factor subunit 1 | 49.03 | 158.9 | 34.8 | 16 | 27 | 16 | 1.04 | 1.5% |
| Q62871 | Cytoplasmic dynein 1 intermediate chain 2 | 71.177 | 206.34 | 24.5 | 12 | 16 | 12 | 1.04 | 2.5% |
| A0A0G2K9D6 | Protein Smarcc1 | 120.95 | 3.8988 | 5.4 | 6 | 7 | 3 | 1.04 | 2.7% |
| Q6P7P5 | Basic leucine zipper and W2 domain-containing protein 1 | 48.043 | 24.941 | 21.7 | 11 | 19 | 10 | 1.03 | 6.3% |
| Q6P762 | Alpha-mannosidase | 114.33 | 49.193 | 11 | 9 | 11 | 9 | 1.03 | 4.7% |
| P09456 | cAMP-dependent protein kinase type I-alpha regulatory subunit | 43.094 | 128.79 | 37.3 | 14 | 22 | 11 | 1.03 | 0.6% |
| D4A7R3 | Nucleoporin 205kDa (Predicted) | 227.23 | 72.106 | 7.6 | 13 | 14 | 13 | 1.03 | 0.3% |
| Q9Z1Z9 | PDZ and LIM domain protein 7 | 49.912 | 246.98 | 64.8 | 24 | 57 | 24 | 1.03 | 0.4% |
| F1LVA9 | Protein Dock5 | 214.67 | 21.846 | 3.5 | 7 | 8 | 6 | 1.03 | 1.2% |
| A0A0G2JUF2 | Protein Snx30 | 53.239 | 15.091 | 13.8 | 7 | 8 | 7 | 1.03 | 1.4% |
| Q8VHU4 | Elongator complex protein 1 | 149.17 | 163.13 | 18.7 | 20 | 29 | 20 | 1.03 | 1.7% |
| D4AC36 | Protein Eif3f | 37.997 | 57.699 | 22.7 | 8 | 19 | 8 | 1.03 | 2.2% |
| Q8R5M4 | Optineurin | 67.013 | 47.705 | 20.7 | 13 | 17 | 13 | 1.03 | 6.2% |
| D3ZMK9 | Protein Pragmin | 147.71 | 9.9717 | 2.6 | 3 | 3 | 3 | 1.03 | 1.7% |
| P56558 | UDP-N-acetylglucosamine--peptide N-acetylglucosaminyltransferase 110 kDa subunit | 115.6 | 44.768 | 6.8 | 6 | 7 | 6 | 1.03 | 0.8% |
| Q5U211 | Sorting nexin-3 | 18.762 | 35.907 | 42 | 8 | 12 | 6 | 1.03 | 0.4% |
| B5DFC3 | Protein Sec23a | 86.161 | 221.53 | 37.5 | 24 | 58 | 21 | 1.03 | 1.2% |
| D3ZKX1 | Protein Ccdc53 | 21.063 | 44.771 | 24.2 | 4 | 5 | 4 | 1.03 | 3.4% |
| D3Z9C0 | Protein Mios | 98.409 | 16.974 | 5 | 3 | 3 | 3 | 1.03 | 6.8% |
| B2RZ66 | Protein Srp19 | 16.138 | 87.352 | 62.5 | 7 | 10 | 7 | 1.03 | 2.0% |
| Q5FVI3 | Leucine-rich repeat-containing protein 57 | 26.724 | 25.54 | 18.8 | 4 | 10 | 4 | 1.03 | 0.9% |
| D3ZFA8 | 40S ribosomal protein S17 | 15.468 | 124.51 | 59.3 | 8 | 19 | 8 | 1.03 | 0.7% |
| Q63495 | Advanced glycosylation end product-specific receptor | 42.663 | 3.1788 | 3.5 | 1 | 1 | 1 | 1.03 | 12.1% |
| D4AD36 | FCH and double SH3 domains 1 (Predicted), isoform CRA_b | 76.766 | 11.769 | 3.3 | 2 | 3 | 2 | 1.03 | 4.0% |
| Q8CFN2 | Cell division control protein 42 homolog | 21.258 | 84.217 | 43.5 | 8 | 28 | 7 | 1.03 | 0.1% |
| Q02874 | Core histone macro-H2A.1 | 39.503 | 34.469 | 17 | 5 | 6 | 4 | 1.03 | 0.2% |
| Q5XIG4 | OCIA domain-containing protein 1 | 27.659 | 20.087 | 22.7 | 5 | 8 | 5 | 1.03 | 0.4% |
| P35426 | Cyclin-dependent kinase 4 | 33.799 | 29.127 | 15.8 | 4 | 6 | 3 | 1.03 | 3.7% |
| D4ACM1 | Protein Elp3 | 62.36 | 42.048 | 17.2 | 8 | 12 | 8 | 1.03 | 5.3% |
| D4AEJ0 | Protein RGD1560617 | 44.273 | 36.179 | 6.5 | 2 | 8 | 1 | 1.03 | 4.0% |
| Q4QQS7 | Protein Umps | 52.378 | 82.357 | 26.6 | 9 | 11 | 9 | 1.03 | 3.5% |
| Q9R064 | Golgi reassembly-stacking protein 2 | 47.22 | 56.394 | 17 | 7 | 15 | 7 | 1.03 | 0.2% |
| O08651 | D-3-phosphoglycerate dehydrogenase | 56.493 | 150.25 | 28.7 | 13 | 26 | 13 | 1.03 | 0.6% |
| Q5M7A4 | Ubiquitin-like modifier-activating enzyme 5 | 44.895 | 97.245 | 34.5 | 10 | 14 | 10 | 1.03 | 2.6% |
| D3ZUQ7 | Pantothenate kinase 3 (Predicted) | 41.09 | 2.5123 | 1.9 | 1 | 2 | 1 | 1.03 | 2.0% |
| F1LPQ2 | Activating signal cointegrator 1 complex subunit 3 | 250.22 | 57.575 | 6.5 | 13 | 13 | 13 | 1.03 | 2.0% |
| P86182 | Coiled-coil domain-containing protein 22 | 70.854 | 129.91 | 30.8 | 14 | 19 | 14 | 1.03 | 0.7% |
| B2RYP4 | Protein Snx2 | 58.53 | 59.684 | 23.3 | 11 | 17 | 10 | 1.03 | 0.4% |
| B1H267 | Sorting nexin-5 | 46.793 | 113.68 | 33.2 | 14 | 21 | 14 | 1.03 | 1.2% |
| B1WBW2 | Protein Rwdd2b | 33.355 | 2.9563 | 2.8 | 1 | 1 | 1 | 1.03 | 6.4% |
| D4ACG2 | IlvB (Bacterial acetolactate synthase)-like (Predicted), isoform CRA_c | 72.269 | 50.239 | 15.6 | 7 | 7 | 7 | 1.03 | 3.6% |
| Q9JMB5 | Proteasomal ubiquitin receptor ADRM1 | 42.102 | 46.92 | 16.2 | 7 | 12 | 7 | 1.03 | 2.4% |
| P62870 | Transcription elongation factor B polypeptide 2 | 13.17 | 59.377 | 90.7 | 10 | 15 | 10 | 1.03 | 4.0% |
| B2RYA8 | DnaJ homolog subfamily B member 6 | 30.694 | 4.9306 | 7.9 | 2 | 2 | 2 | 1.03 | 2.5% |
| P28480 | T-complex protein 1 subunit alpha | 60.359 | 248.12 | 49.6 | 23 | 60 | 23 | 1.03 | 1.9% |
| P62909 | 40S ribosomal protein S3 | 26.674 | 93.695 | 61.3 | 15 | 28 | 15 | 1.03 | 0.3% |
| Q63862 | Myosin-11 | 152.49 | 323.31 | 56.1 | 93 | 284 | 68 | 1.03 | 0.0% |
| D4A7K6 | Protein Med10 | 15.661 | 11.978 | 9.6 | 1 | 2 | 1 | 1.03 | 0.6% |
| P61314 | 60S ribosomal protein L15 | 24.146 | 50.777 | 52.9 | 12 | 21 | 12 | 1.03 | 1.1% |
| Q6AYU5 | Poly(RC) binding protein 2 | 38.58 | 94.879 | 49.9 | 12 | 22 | 7 | 1.03 | 1.2% |
| P05708 | Hexokinase-1 | 102.41 | 323.31 | 45.1 | 41 | 76 | 38 | 1.03 | 2.1% |
| D3ZVP7 | Zinc finger FYVE domain-containing protein 21 | 26.009 | 16.092 | 9 | 1 | 2 | 1 | 1.03 | 2.6% |
| D4A6D9 | Protein Hs1bp3 | 43.431 | 65.261 | 22.3 | 6 | 9 | 6 | 1.03 | 0.9% |
| G3V852 | Protein Tln1 | 269.67 | 323.31 | 64.8 | 135 | 336 | 116 | 1.03 | 0.7% |
| A0A0G2K350 | Protein Napg | 34.659 | 36.43 | 19.6 | 6 | 8 | 6 | 1.03 | 0.9% |
| B4F775 | Golgi associated PDZ and coiled-coil motif containing | 34.167 | 21.565 | 15.9 | 4 | 5 | 4 | 1.03 | 5.4% |
| A0A0G2JZD1 | Protein Ppp2r5c | 63.746 | 33.197 | 14.5 | 6 | 7 | 5 | 1.03 | 3.5% |
| Q6JP77 | A-kinase anchor protein 7 isoforms delta and gamma | 39.417 | 12.156 | 5.7 | 2 | 2 | 2 | 1.03 | 10.2% |
| D3ZJS3 | Protein Tomm6 | 7.8938 | 5.5052 | 18.9 | 1 | 3 | 1 | 1.03 | 4.4% |
| F1LNH3 | Procollagen, type VI, alpha 2, isoform CRA_a | 109.66 | 23.907 | 7.6 | 7 | 7 | 7 | 1.03 | 3.6% |
| B2GUZ7 | Protein Tbcc | 38.076 | 37.012 | 13.2 | 4 | 5 | 4 | 1.03 | 1.5% |
| F1M0X6 | Protein mago nashi homolog | 17.29 | 53.868 | 48.6 | 6 | 7 | 6 | 1.03 | 1.3% |
| P12785 | Fatty acid synthase | 272.65 | 295.75 | 21.5 | 45 | 69 | 45 | 1.03 | 0.4% |
| Q4V794 | Protein Vps37a | 44.487 | 27.062 | 10.6 | 3 | 3 | 3 | 1.03 | 0.4% |
| Q64119 | Myosin light polypeptide 6 | 16.975 | 103.09 | 60.3 | 9 | 67 | 7 | 1.03 | 0.1% |
| B2RYN1 | Fructosamine-3-kinase-related protein | 34.169 | 12.659 | 8.7 | 2 | 3 | 2 | 1.03 | 1.6% |
| Q5BK85 | Transmembrane emp24 domain-containing protein 1 | 25.209 | 10.418 | 5.7 | 1 | 1 | 1 | 1.03 | 3.2% |
| Q2EJA0 | Yorkie homolog | 50.501 | 90.757 | 23 | 7 | 11 | 7 | 1.03 | 5.9% |
| G3V992 | General transcription factor II E, polypeptide 1 (Alpha subunit) | 49.228 | 6.3606 | 4.6 | 2 | 3 | 2 | 1.03 | 7.8% |
| Q5FVM1 | Transmembrane protein 231 | 36.169 | 2.6708 | 2.2 | 1 | 1 | 1 | 1.03 | 6.2% |
| D3Z8B2 | Protein Nup133 | 128.36 | 88.899 | 9.4 | 7 | 8 | 7 | 1.03 | 3.1% |
| Q5XIB4 | Ufm1-specific protease 2 | 52.306 | 67.465 | 22.8 | 9 | 10 | 9 | 1.03 | 2.9% |
| P52303 | AP-1 complex subunit beta-1 | 104.59 | 225.73 | 38.8 | 33 | 50 | 15 | 1.03 | 0.6% |
| Q5XIG0 | ADP-ribose pyrophosphatase, mitochondrial | 38.562 | 8.3046 | 7.1 | 3 | 3 | 3 | 1.03 | 0.6% |
| G3V926 | Conserved helix-loop-helix ubiquitous kinase (Predicted) | 84.785 | 31.142 | 2.1 | 1 | 2 | 1 | 1.03 | 1.9% |
| Q5M824 | SHC-transforming protein 1 | 51.503 | 18.692 | 9.8 | 4 | 4 | 3 | 1.03 | 6.4% |
| M0R515 | Protein Nudt13 | 39.351 | 6.7527 | 4.3 | 1 | 1 | 1 | 1.03 | 11.8% |
| D4A2D3 | Protein Mycbp2 | 498.91 | 7.1197 | 0.3 | 1 | 1 | 1 | 1.03 | 2.4% |
| P63245 | Guanine nucleotide-binding protein subunit beta-2-like 1 | 35.076 | 212.16 | 78.9 | 19 | 41 | 19 | 1.03 | 0.5% |
| P19836 | Choline-phosphate cytidylyltransferase A | 41.68 | 48.03 | 31.3 | 10 | 14 | 9 | 1.03 | 0.3% |
| Q5BJT7 | Coiled-coil domain-containing protein 93 | 72.635 | 41.386 | 15.3 | 9 | 9 | 9 | 1.03 | 2.2% |
| D3ZVA6 | Protein Bola2 | 10.214 | 65.358 | 62.8 | 5 | 8 | 5 | 1.03 | 2.0% |
| A0A096MIZ1 | Protein Palm2 | 42.055 | 33.539 | 20.5 | 8 | 11 | 6 | 1.03 | 1.0% |
| A0A0G2K719 | Protein Ddx3x | 73.145 | 246.19 | 48.6 | 29 | 62 | 7 | 1.03 | 0.0% |
| A0A0G2JY08 | Protein Myo18a | 233.38 | 135.12 | 11.1 | 19 | 23 | 19 | 1.03 | 0.9% |
| G3V887 | V-type proton ATPase subunit a | 93.209 | 84.112 | 13.8 | 9 | 12 | 9 | 1.03 | 1.5% |
| D4A0E8 | Protein arginine N-methyltransferase 5 | 72.694 | 25.513 | 11.1 | 6 | 7 | 6 | 1.03 | 4.1% |
| Q5RJK9 | Polymerase (RNA) I polypeptide C | 39.017 | 35.6 | 11 | 3 | 4 | 3 | 1.03 | 6.7% |
| P18421 | Proteasome subunit beta type-1 | 26.479 | 99.949 | 40.8 | 8 | 18 | 8 | 1.03 | 1.2% |
| Q9QY17 | Protein kinase C and casein kinase substrate in neurons 2 protein | 55.977 | 34.734 | 19.3 | 10 | 12 | 10 | 1.03 | 0.1% |
| D3ZPR0 | Chromosome segregation 1-like (S. cerevisiae) (Predicted) | 110.21 | 122.45 | 15.9 | 13 | 14 | 13 | 1.03 | 1.0% |
| Q63569 | 26S protease regulatory subunit 6A | 49.16 | 308.86 | 64 | 24 | 62 | 24 | 1.03 | 1.0% |
| D4ADS9 | Protein Efr3a | 92.523 | 26.893 | 9.4 | 5 | 5 | 5 | 1.03 | 1.8% |
| Q4KM87 | Actin-like 6A | 47.42 | 22.184 | 13.3 | 5 | 6 | 5 | 1.03 | 5.1% |
| Q9QYL8 | Acyl-protein thioesterase 2 | 24.807 | 39.046 | 21.2 | 4 | 5 | 4 | 1.03 | 0.7% |
| P50878 | 60S ribosomal protein L4 | 47.256 | 170.78 | 42.8 | 20 | 53 | 20 | 1.03 | 0.5% |
| D4AE06 | Peptidyl-prolyl cis-trans isomerase | 131.17 | 27.714 | 7.9 | 8 | 9 | 8 | 1.03 | 0.0% |
| D3ZER6 | Protein Tnpo2 | 102.88 | 25.806 | 14.2 | 11 | 13 | 7 | 1.03 | 0.1% |
| P62168 | Neuronal calcium sensor 1 | 21.878 | 71.802 | 46.3 | 6 | 7 | 6 | 1.03 | 0.1% |
| G3V817 | X-ray repair cross-complementing protein 5 | 83.091 | 28.597 | 8.1 | 5 | 7 | 5 | 1.03 | 0.8% |
| Q9JI51 | Vesicle transport through interaction with t-SNAREs homolog 1A | 26.042 | 4.437 | 7.6 | 2 | 3 | 2 | 1.03 | 1.6% |
| F1LS86 | Isoleucine-tRNA synthetase (Predicted) | 144.28 | 254.52 | 31 | 33 | 53 | 33 | 1.03 | 2.3% |
| F1M7M4 | Protein Bmp2k | 118.71 | 22.83 | 6.5 | 5 | 5 | 4 | 1.03 | 3.2% |
| B2GV38 | Ubiquitin-like protein 4A | 17.79 | 35.083 | 26.8 | 3 | 5 | 3 | 1.03 | 7.3% |
| D4A5K6 | Protein Zmpste24 | 54.847 | 64.344 | 21.3 | 8 | 10 | 8 | 1.03 | 3.1% |
| Q4KLK9 | RNA polymerase II subunit A C-terminal domain phosphatase SSU72 | 22.544 | 6.5241 | 13.4 | 2 | 2 | 2 | 1.03 | 2.1% |
| Q04462 | Valine--tRNA ligase | 140.37 | 259.77 | 33.5 | 34 | 57 | 34 | 1.03 | 1.6% |
| P62198 | 26S protease regulatory subunit 8 | 45.626 | 250.85 | 52.5 | 18 | 41 | 18 | 1.03 | 0.6% |
| P84082 | ADP-ribosylation factor 2 | 20.746 | 11.056 | 58.6 | 9 | 32 | 2 | 1.03 | 0.2% |
| Q641Y8 | ATP-dependent RNA helicase DDX1 | 82.496 | 235.46 | 39.7 | 24 | 53 | 24 | 1.03 | 0.1% |
| P37805 | Transgelin-3 | 22.5 | 14.329 | 21.6 | 4 | 20 | 1 | 1.03 | 0.9% |
| F1M8B7 | Protein Chmp2b | 23.934 | 16.746 | 18.8 | 5 | 7 | 5 | 1.03 | 7.9% |
| P51870 | Cytochrome P450 4F5 | 60.68 | 12.988 | 5.9 | 3 | 5 | 2 | 1.03 | 2.2% |
| D3ZHA0 | Filamin-C | 290.98 | 323.31 | 54.4 | 132 | 323 | 116 | 1.03 | 1.2% |
| A0A0G2K4R1 | Protein Ppp1r12c | 84.765 | 56.321 | 21.2 | 13 | 15 | 13 | 1.03 | 1.1% |
| F1M4S5 | 60S ribosomal protein L36a | 12.278 | 17.304 | 28.3 | 6 | 13 | 6 | 1.03 | 0.8% |
| Q5U2U8 | Bcl2-associated athanogene 3 | 61.49 | 150.58 | 27.4 | 16 | 21 | 16 | 1.03 | 0.2% |
| A0A0G2K3L8 | Protein Wdfy1 | 46.175 | 65.797 | 35.6 | 12 | 21 | 12 | 1.03 | 0.3% |
| P42930 | Heat shock protein beta-1 | 22.892 | 129.12 | 58.7 | 16 | 60 | 15 | 1.03 | 0.5% |
| Q71UF4 | Histone-binding protein RBBP7 | 47.82 | 85.951 | 23.5 | 8 | 14 | 3 | 1.03 | 2.0% |
| Q32KJ6 | N-acetylgalactosamine-6-sulfatase | 58.302 | 73.054 | 20.4 | 10 | 16 | 10 | 1.03 | 5.9% |
| D3ZEI6 | Nuclear receptor coactivator 5 (Predicted) | 65.316 | 35.118 | 5.9 | 2 | 2 | 2 | 1.03 | 0.1% |
| O88902 | Tyrosine-protein phosphatase non-receptor type 23 | 163.45 | 56.068 | 8.1 | 11 | 11 | 11 | 1.03 | 0.1% |
| Q6AZ42 | Gap junction protein | 30.366 | 2.0676 | 2.7 | 1 | 1 | 1 | 1.03 | 0.5% |
| Q6YDN8 | BWK-1 | 26.778 | 56.479 | 26.8 | 4 | 5 | 4 | 1.03 | 0.9% |
| Q6PCU2 | V-type proton ATPase subunit E 1 | 26.128 | 54.62 | 28.3 | 7 | 15 | 7 | 1.03 | 5.8% |
| Q63448 | Peroxisomal acyl-coenzyme A oxidase 3 | 78.445 | 94.483 | 8.3 | 4 | 7 | 4 | 1.03 | 5.1% |
| Q925G1 | Hepatoma-derived growth factor-related protein 2 | 74.017 | 67.575 | 7.6 | 5 | 6 | 4 | 1.03 | 3.3% |
| P11348 | Dihydropteridine reductase | 25.552 | 18.72 | 32.4 | 6 | 6 | 6 | 1.03 | 0.8% |
| D4A6K9 | Active BCR-related gene (Predicted) | 97.692 | 11.528 | 3 | 2 | 2 | 2 | 1.03 | 1.9% |
| F1MAA2 | Protein Cops7a | 30.385 | 38.763 | 28.2 | 7 | 8 | 7 | 1.03 | 4.1% |
| B0BND5 | Protein Rrp9 | 52.502 | 4.0921 | 5 | 2 | 2 | 2 | 1.03 | 5.7% |
| O54874 | Serine/threonine-protein kinase MRCK alpha | 197.06 | 75.668 | 12.8 | 20 | 23 | 17 | 1.03 | 3.8% |
| A0A0G2JWD6 | Adaptor-related protein complex 3, beta 1 subunit (Predicted), isoform CRA_a | 121.5 | 135.39 | 23.1 | 22 | 28 | 22 | 1.03 | 0.3% |
| D3ZXS8 | Huntingtin interacting protein 2 (Predicted), isoform CRA_a | 22.406 | 51.819 | 36.5 | 8 | 14 | 8 | 1.03 | 0.2% |
| D4A2G9 | Protein Ranbp1 | 23.596 | 72.885 | 35.5 | 7 | 16 | 7 | 1.03 | 1.2% |
| Q66HS7 | PDZ and LIM domain protein 3 | 39.106 | 63.467 | 19.9 | 5 | 11 | 5 | 1.03 | 1.7% |
| P35286 | Ras-related protein Rab-13 | 22.901 | 12.314 | 36.9 | 7 | 12 | 4 | 1.03 | 5.0% |
| Q5XID1 | Anamorsin | 33.041 | 19.041 | 15.5 | 4 | 5 | 4 | 1.03 | 6.8% |
| F1M801 | Anaphase promoting complex subunit 1 (Predicted) | 216.08 | 33.205 | 4 | 7 | 7 | 7 | 1.03 | 4.3% |
| B5DF60 | Eukaryotic translation initiation factor 1A, Y-linked | 16.46 | 47.402 | 48.6 | 8 | 15 | 3 | 1.03 | 2.3% |
| P97874 | Cyclin-G-associated kinase | 143.7 | 48.809 | 9.4 | 11 | 11 | 11 | 1.03 | 1.6% |
| C0JPT7 | Filamin alpha | 280.49 | 323.31 | 65.5 | 150 | 610 | 138 | 1.03 | 0.2% |
| G3V8T5 | Protein Ruvbl2 | 51.112 | 119.01 | 39.3 | 15 | 22 | 15 | 1.03 | 1.9% |
| M0RB26 | Protein Gareml | 93.488 | 2.0467 | 2.2 | 2 | 2 | 2 | 1.03 | 1.9% |
| P63004 | Platelet-activating factor acetylhydrolase IB subunit alpha | 46.67 | 121.11 | 43.9 | 16 | 36 | 16 | 1.03 | 2.5% |
| A0A0G2K761 | Protein Cul2 | 86.968 | 35.084 | 10.7 | 9 | 10 | 9 | 1.03 | 3.0% |
| P62747 | Rho-related GTP-binding protein RhoB | 22.123 | 5.5121 | 23 | 4 | 7 | 2 | 1.03 | 4.2% |
| P70619 | Glutathione reductase | 46.301 | 39.074 | 18.2 | 5 | 5 | 5 | 1.03 | 5.4% |
| Q63560 | Microtubule-associated protein 6 | 100.48 | 147.8 | 31.7 | 17 | 21 | 17 | 1.03 | 2.0% |
| Q9JLT6 | BH3-interacting domain death agonist | 22.249 | 54.488 | 18.4 | 2 | 3 | 2 | 1.03 | 4.2% |
| Q62835 | Rab GTPase-binding effector protein 2 | 61.972 | 52.512 | 19.9 | 9 | 9 | 9 | 1.03 | 4.0% |
| Q5XI68 | Protein Dr1 | 19.429 | 9.0511 | 15.9 | 3 | 5 | 3 | 1.03 | 2.6% |
| Q3SWT7 | Nuclear receptor binding protein | 59.792 | 43.861 | 12 | 4 | 7 | 4 | 1.03 | 0.0% |
| P30839 | Fatty aldehyde dehydrogenase | 54.081 | 41.901 | 17.6 | 8 | 9 | 8 | 1.03 | 0.1% |
| P85970 | Actin-related protein 2/3 complex subunit 2 | 34.391 | 95.184 | 41 | 13 | 37 | 13 | 1.03 | 0.2% |
| Q5XIR9 | Ubiquitin-associated domain-containing protein 1 | 45.546 | 25.248 | 8.1 | 3 | 4 | 3 | 1.03 | 0.2% |
| D3ZQI6 | Exportin 4 (Predicted) | 129.66 | 8.7155 | 3.5 | 3 | 3 | 3 | 1.03 | 1.1% |
| O88321 | Antisecretory factor | 41.073 | 174.08 | 39.2 | 14 | 24 | 14 | 1.03 | 3.3% |
| P13599 | IgG receptor FcRn large subunit p51 | 40.168 | 18.852 | 8.5 | 5 | 7 | 5 | 1.03 | 4.9% |
| A0A0G2K051 | Protein Eea1 | 165.41 | 323.31 | 38.7 | 48 | 62 | 48 | 1.03 | 2.3% |
| B2RYL4 | Armc6 protein | 50.647 | 53.113 | 19.2 | 6 | 9 | 6 | 1.03 | 0.3% |
| A0A0G2K5T9 | Protein Wasf2 | 53.873 | 26.707 | 10.7 | 4 | 5 | 4 | 1.03 | 0.4% |
| B1WBY1 | Cul1 protein | 89.69 | 69.235 | 23.1 | 16 | 19 | 16 | 1.03 | 0.6% |
| Q641Z2 | Tyrosine-protein phosphatase non-receptor type 9 | 67.961 | 54.437 | 17.5 | 9 | 12 | 9 | 1.03 | 0.9% |
| D3ZPF2 | Protein Mcat | 41.832 | 3.5692 | 3.9 | 1 | 1 | 1 | 1.02 | 13.4% |
| Q8K4M9 | Oxysterol-binding protein-related protein 1 | 107.76 | 7.7938 | 4.5 | 4 | 4 | 4 | 1.02 | 10.9% |
| P07314 | Gamma-glutamyltranspeptidase 1 | 61.609 | 2.9757 | 2.1 | 1 | 2 | 1 | 1.02 | 7.3% |
| Q66H91 | G protein-coupled receptor kinase interacting ArfGAP 2 | 84.524 | 13.355 | 6.9 | 5 | 5 | 4 | 1.02 | 4.3% |
| D3Z8Q7 | Protein Fam96b | 17.751 | 2.0686 | 4.2 | 1 | 1 | 1 | 1.02 | 3.3% |
| O54748 | Serine/threonine-protein kinase 3 | 56.121 | 35.712 | 16.3 | 7 | 8 | 5 | 1.02 | 3.2% |
| F1LNI5 | Protein phosphatase 1G | 58.742 | 29.665 | 13.3 | 6 | 7 | 6 | 1.02 | 1.6% |
| E9PTA5 | Protein Pign | 105 | 24.742 | 4.3 | 3 | 4 | 3 | 1.02 | 0.7% |
| A0A0G2KAE2 | Protein Tusc3 | 39.623 | 7.3021 | 5.5 | 2 | 2 | 2 | 1.02 | 0.9% |
| A0A0G2K160 | Protein Ahctf1 | 255.18 | 9.3494 | 1.9 | 3 | 3 | 3 | 1.02 | 1.1% |
| P62907 | 60S ribosomal protein L10a | 24.831 | 102.09 | 42.9 | 11 | 24 | 11 | 1.02 | 1.1% |
| D4A9Z6 | Mitochondrial ribosomal protein S35 (Predicted) | 36.206 | 20.176 | 9.4 | 2 | 3 | 2 | 1.02 | 1.8% |
| D3ZUD8 | Protein Tm9sf3 | 69.968 | 27.156 | 12.2 | 7 | 9 | 7 | 1.02 | 5.6% |
| F1LM93 | Tyrosine-protein kinase Yes | 60.613 | 79.533 | 19.8 | 9 | 12 | 6 | 1.02 | 6.6% |
| D3ZSN4 | Protein Zfp330 | 35.725 | 41.435 | 20.8 | 6 | 10 | 6 | 1.02 | 2.6% |
| F1LVX2 | Protein Ehbp1 | 136.19 | 3.7585 | 1.8 | 2 | 2 | 2 | 1.02 | 2.1% |
| Q5BJN3 | Protein Tial1 | 43.388 | 34.784 | 23 | 7 | 9 | 7 | 1.02 | 0.3% |
| P16638 | ATP-citrate synthase | 120.63 | 136.55 | 21.9 | 20 | 29 | 20 | 1.02 | 2.1% |
| A0A0G2K1P8 | Protein Triobp | 223.92 | 59.053 | 7.8 | 15 | 17 | 14 | 1.02 | 3.4% |
| P18297 | Sepiapterin reductase | 28.128 | 64.37 | 29.8 | 6 | 9 | 6 | 1.02 | 9.0% |
| D4AC65 | Protein Coa7 | 25.711 | 4.9789 | 4.3 | 1 | 1 | 1 | 1.02 | 4.3% |
| D3ZEN0 | MICAL-like protein 2 | 108.38 | 40.68 | 14.2 | 11 | 13 | 11 | 1.02 | 6.0% |
| D3ZP87 | Protein Fam92a1 | 39.719 | 9.9518 | 5.9 | 2 | 2 | 2 | 1.02 | 1.7% |
| Q5FVI6 | V-type proton ATPase subunit C 1 | 43.9 | 101.39 | 46.9 | 18 | 22 | 18 | 1.02 | 1.0% |
| A0A096MJN2 | Protein Nudcd3 | 40.769 | 16.228 | 13.8 | 4 | 4 | 4 | 1.02 | 0.8% |
| D4AAU6 | Coiled-coil domain containing 25 (Predicted) | 24.466 | 11.991 | 15.4 | 3 | 3 | 3 | 1.02 | 0.0% |
| Q9JJ19 | Na(+)/H(+) exchange regulatory cofactor NHE-RF1 | 38.83 | 9.3878 | 12.9 | 4 | 4 | 4 | 1.02 | 0.1% |
| G3V7Q7 | IQ motif containing GTPase activating protein 1 (Predicted), isoform CRA_b | 188.83 | 323.31 | 53.1 | 72 | 177 | 68 | 1.02 | 0.8% |
| Q4QQV1 | Tax1-binding protein 3 | 11.049 | 2.5828 | 7.1 | 1 | 2 | 1 | 1.02 | 2.7% |
| B5DFL0 | Protein Snta1 | 53.362 | 58.777 | 21.6 | 10 | 11 | 10 | 1.02 | 1.7% |
| Q6AY09 | Heterogeneous nuclear ribonucleoprotein H2 | 49.293 | 122.06 | 31.2 | 11 | 27 | 4 | 1.02 | 0.1% |
| Q63615 | Vacuolar protein sorting-associated protein 33A | 67.513 | 13.199 | 6.5 | 4 | 4 | 4 | 1.02 | 4.6% |
| M0RCW9 | Protein LOC100909868 | 25.465 | 6.5989 | 4.8 | 1 | 1 | 1 | 1.02 | 11.7% |
| D3ZG88 | Mammary tumor virus receptor 2, isoform CRA_a | 21.318 | 42.854 | 24.1 | 2 | 3 | 2 | 1.02 | 15.9% |
| G3V6M8 | Nucleoporin 37 (Predicted), isoform CRA_a | 36.768 | 46.017 | 21.2 | 7 | 7 | 6 | 1.02 | 2.5% |
| E9PU64 | Protein Scin | 80.142 | 99.519 | 21.4 | 13 | 14 | 12 | 1.02 | 2.3% |
| Q5FVQ4 | Malectin | 32.418 | 99.314 | 42.6 | 12 | 24 | 12 | 1.02 | 1.2% |
| D3Z7Z5 | Protein Ranbp10 | 77.815 | 13.102 | 6.5 | 4 | 4 | 4 | 1.02 | 0.8% |
| E9PST5 | Protein Acin1 | 151.04 | 34.133 | 6.3 | 9 | 9 | 9 | 1.02 | 0.2% |
| D4A0W7 | Protein Fndc3b | 132.78 | 149.28 | 17.1 | 14 | 17 | 14 | 1.02 | 0.8% |
| Q8K5A9 | Death domain-containing membrane protein NRADD | 24.399 | 24.395 | 21.5 | 4 | 5 | 4 | 1.02 | 12.5% |
| P00173 | Cytochrome b5 | 15.355 | 19.56 | 37.3 | 5 | 8 | 5 | 1.02 | 7.4% |
| Q3MIB4 | Lon protease homolog 2, peroxisomal | 94.392 | 7.5361 | 4.7 | 4 | 4 | 4 | 1.02 | 7.2% |
| Q5BJM8 | Zinc transporter 7 | 41.773 | 6.1605 | 6.6 | 2 | 2 | 2 | 1.02 | 5.6% |
| P37996 | ADP-ribosylation factor-like protein 3 | 20.456 | 97.197 | 62.6 | 9 | 14 | 9 | 1.02 | 2.7% |
| P18484 | AP-2 complex subunit alpha-2 | 104.04 | 283.87 | 36.4 | 31 | 65 | 24 | 1.02 | 0.6% |
| D4A3Z4 | Protein Gcfc2 | 82.836 | 2.7903 | 1.4 | 1 | 1 | 1 | 1.02 | 0.0% |
| D3ZX42 | G protein-coupled receptor 21 (Predicted), isoform CRA_a | 120.96 | 147.14 | 17.7 | 14 | 16 | 14 | 1.02 | 0.4% |
| D3ZDR2 | Chromatin modifying protein 6 (Predicted) | 23.288 | 28.782 | 13.5 | 2 | 3 | 2 | 1.02 | 1.0% |
| O55166 | Vacuolar protein sorting-associated protein 52 homolog | 82.102 | 15.221 | 6.1 | 4 | 4 | 4 | 1.02 | 1.5% |
| D4ABQ7 | Protein Arap1 | 162.3 | 31.761 | 3.9 | 5 | 9 | 5 | 1.02 | 5.2% |
| D3ZNU1 | Protein Cebpz | 119.78 | 9.6096 | 1.1 | 1 | 1 | 1 | 1.02 | 10.4% |
| Q5XI22 | Acetyl-CoA acetyltransferase, cytosolic | 41.108 | 56.944 | 15.6 | 5 | 8 | 5 | 1.02 | 8.0% |
| A0A0G2JWK8 | Protein Ewsr1 | 68.905 | 30.695 | 9.5 | 5 | 8 | 5 | 1.02 | 5.0% |
| M0R9I8 | 40S ribosomal protein S12 | 14.501 | 80.591 | 64.4 | 9 | 24 | 9 | 1.02 | 1.9% |
| B1H222 | RAB6-interacting golgin | 41.631 | 23.018 | 8.2 | 2 | 2 | 2 | 1.02 | 7.2% |
| A0A0G2KAE1 | Protein Lims2 | 38.962 | 30.831 | 25.2 | 8 | 14 | 4 | 1.02 | 5.1% |
| D3ZX38 | Prefoldin 1 (Predicted) | 14.255 | 13.795 | 29.5 | 4 | 9 | 4 | 1.02 | 4.8% |
| D3ZV15 | Protein Cbl | 100.69 | 15.731 | 2.3 | 2 | 3 | 2 | 1.02 | 2.1% |
| M0R5K9 | 40S ribosomal protein S18 | 17.654 | 36.317 | 47 | 10 | 17 | 10 | 1.02 | 2.1% |
| F1LW07 | Protein Ppp1r13l | 89 | 27.333 | 8.2 | 4 | 5 | 4 | 1.02 | 6.8% |
| P27881 | Hexokinase-2 | 102.54 | 25.06 | 8.7 | 7 | 12 | 4 | 1.02 | 7.0% |
| Q7TP47 | Heterogeneous nuclear ribonucleoprotein Q | 59.71 | 102.78 | 29.3 | 15 | 35 | 7 | 1.02 | 3.4% |
| B4F784 | Protein Shq1 | 67.737 | 13.34 | 3.6 | 2 | 2 | 2 | 1.02 | 2.4% |
| D3ZYT2 | Mitochondrial ribosomal protein S5 (Predicted) | 48.136 | 2.6389 | 2.5 | 1 | 1 | 1 | 1.02 | 2.4% |
| Q8R4C0 | Calpain-5 | 73.064 | 3.3699 | 2.3 | 2 | 2 | 2 | 1.02 | 0.6% |
| D4A0W1 | Protein Emc4 | 20.158 | 9.6869 | 14.2 | 2 | 5 | 2 | 1.02 | 11.0% |
| M0RCH5 | Glucosamine-6-phosphate isomerase | 27.987 | 32.354 | 29.6 | 7 | 10 | 5 | 1.02 | 5.4% |
| Q8K4V4 | Sorting nexin-27 | 61.014 | 6.1683 | 3.5 | 2 | 2 | 2 | 1.02 | 3.2% |
| G3V8Q8 | Protein Sec23ip | 110.94 | 102.75 | 23 | 21 | 28 | 21 | 1.02 | 1.7% |
| A0A0G2JTJ9 | Protein Tfcp2 | 56.982 | 7.6725 | 3.8 | 2 | 3 | 1 | 1.02 | 3.5% |
| Q9JK72 | Copper chaperone for superoxide dismutase | 28.889 | 23.711 | 12.8 | 3 | 3 | 3 | 1.02 | 9.5% |
| F7EZF5 | Protein Pid1 | 27.743 | 27.533 | 14.8 | 2 | 3 | 2 | 1.02 | 6.4% |
| P70581 | Nucleoporin p58/p45 | 59.264 | 6.449 | 5.5 | 3 | 3 | 3 | 1.02 | 4.1% |
| D3ZUJ8 | Protein Tmtc3 | 104.06 | 49.236 | 10 | 7 | 8 | 7 | 1.02 | 3.8% |
| E9PU13 | Protein Snx4 | 51.927 | 87.198 | 34.9 | 15 | 21 | 15 | 1.02 | 3.8% |
| Q6TQE1 | Zinc finger CCCH domain-containing protein 18 | 105.54 | 4.2915 | 2.4 | 2 | 2 | 2 | 1.02 | 3.5% |
| Q9WV63 | Kinesin-like protein KIF2A | 79.792 | 28.584 | 11.9 | 8 | 8 | 8 | 1.02 | 1.0% |
| P63074 | Eukaryotic translation initiation factor 4E | 25.053 | 24.902 | 20.3 | 4 | 7 | 4 | 1.02 | 0.9% |
| B2GV14 | Protein Txlna | 62.566 | 71.079 | 22.6 | 10 | 13 | 10 | 1.02 | 0.6% |
| Q6P9U8 | Eukaryotic translation initiation factor 3 subunit H | 39.905 | 125.04 | 42 | 12 | 26 | 12 | 1.02 | 0.5% |
| Q63396 | Activated RNA polymerase II transcriptional coactivator p15 | 14.441 | 19.033 | 25.2 | 3 | 3 | 3 | 1.02 | 0.6% |
| D3ZTW8 | Mitochondrial ribosomal protein L27 (Predicted), isoform CRA_a | 15.823 | 14.093 | 14.2 | 2 | 2 | 2 | 1.02 | 0.8% |
| O88339 | Epsin-1 | 60.157 | 78.911 | 11.5 | 5 | 7 | 4 | 1.02 | 1.1% |
| D3ZT51 | Protein Exosc3 | 22.722 | 9.795 | 18.3 | 2 | 2 | 2 | 1.02 | 2.1% |
| A0A0G2K9M4 | Protein Wdfy3 | 391.57 | 26.748 | 2.2 | 6 | 7 | 6 | 1.02 | 2.4% |
| Q6AXN3 | Transmembrane emp24 domain-containing protein 5 | 26.136 | 15.816 | 12.7 | 3 | 5 | 3 | 1.02 | 2.0% |
| Q810F4 | Protein FAM3C | 24.713 | 58.31 | 30.4 | 5 | 5 | 5 | 1.02 | 2.1% |
| E9PTV0 | Guanylate kinase 1, isoform CRA_a | 24.052 | 44.023 | 28.3 | 5 | 8 | 5 | 1.02 | 3.1% |
| Q5XIB7 | tRNA-splicing endonuclease subunit Sen34 | 33.847 | 10.688 | 4.2 | 1 | 2 | 1 | 1.02 | 3.3% |
| D3ZXD0 | Gremlin | 19.348 | 11.343 | 19.6 | 2 | 3 | 2 | 1.02 | 3.7% |
| D3ZYL0 | Protein Ripk1 | 74.756 | 18.284 | 5.3 | 3 | 4 | 3 | 1.02 | 4.9% |
| F7F350 | Protein Syap1 | 40.797 | 35.919 | 20.8 | 7 | 9 | 7 | 1.02 | 5.4% |
| D3ZPP2 | Protein Arl8a | 21.39 | 79.394 | 39.2 | 7 | 16 | 4 | 1.02 | 0.3% |
| B2RYP3 | Fam91a1 protein | 93.573 | 39.475 | 8.6 | 6 | 6 | 6 | 1.02 | 8.1% |
| Q4KM77 | Etoposide-induced protein 2.4 homolog | 38.892 | 5.7476 | 6.8 | 3 | 3 | 3 | 1.02 | 10.1% |
| D3ZN76 | Protein Sec16a | 253.29 | 53.264 | 5.8 | 12 | 15 | 12 | 1.02 | 0.8% |
| Q9ESI7 | Neuronal migration protein doublecortin | 40.559 | 8.2885 | 3 | 1 | 2 | 1 | 1.02 | 0.2% |
| P05426 | 60S ribosomal protein L7 | 30.329 | 150.77 | 51.9 | 18 | 39 | 17 | 1.02 | 1.4% |
| Q62760 | Mitochondrial import receptor subunit TOM20 homolog | 16.284 | 5.8055 | 13.8 | 2 | 3 | 2 | 1.02 | 2.7% |
| D4A4T0 | Protein Stub1 | 34.886 | 14.83 | 16.4 | 5 | 5 | 5 | 1.02 | 3.7% |
| Q6AYR1 | Protein Tfg | 43.095 | 159.97 | 33.2 | 11 | 20 | 11 | 1.02 | 3.9% |
| M0RC57 | Protein unc-45 homolog A | 47.661 | 43.418 | 15.9 | 5 | 6 | 5 | 1.02 | 4.6% |
| Q5U1V9 | Tetraspanin-31 | 22.66 | 6.5411 | 9.5 | 2 | 5 | 2 | 1.02 | 4.5% |
| Q63484 | RAC-gamma serine/threonine-protein kinase | 55.796 | 3.1439 | 9.8 | 3 | 4 | 1 | 1.02 | 3.1% |
| Q5XIM5 | Protein CDV3 homolog | 24.31 | 176.17 | 57.2 | 11 | 18 | 11 | 1.02 | 1.7% |
| A0A0G2JYI0 | Protein Lrba | 309.45 | 42.099 | 3.1 | 8 | 8 | 7 | 1.02 | 1.6% |
| P48004 | Proteasome subunit alpha type-7 | 28.326 | 61.767 | 33.1 | 7 | 11 | 6 | 1.02 | 0.5% |
| Q6SA80 | Rho-related GTP-binding protein RhoE | 27.368 | 53.056 | 30.3 | 6 | 9 | 6 | 1.02 | 0.5% |
| P32362 | Uroporphyrinogen decarboxylase | 40.452 | 44.584 | 18.7 | 5 | 7 | 5 | 1.02 | 2.2% |
| Q5XIA5 | Coenzyme A synthase | 62.187 | 18.607 | 6.2 | 3 | 5 | 3 | 1.02 | 2.2% |
| Q8K3X8 | Heat shock factor-binding protein 1 | 8.5847 | 65.106 | 81.6 | 4 | 7 | 4 | 1.02 | 3.0% |
| Q5XIA4 | CCR4-NOT transcription complex subunit 10 | 81.818 | 16.018 | 3 | 2 | 3 | 2 | 1.02 | 8.5% |
| Q5BJS0 | Putative ATP-dependent RNA helicase DHX30 | 134 | 22.489 | 5.3 | 6 | 6 | 6 | 1.02 | 9.5% |
| B1WC28 | Histone H2A | 40.092 | 28.233 | 20.7 | 7 | 7 | 6 | 1.02 | 6.4% |
| D3ZUP5 | Protein Brk1 | 8.7608 | 7.4718 | 32 | 3 | 7 | 3 | 1.02 | 5.7% |
| Q793F9 | Vacuolar protein sorting-associated protein 4A | 48.906 | 6.2837 | 10.3 | 5 | 5 | 3 | 1.02 | 2.4% |
| A0A0G2QC22 | Protein RGD1306215 | 22.087 | 4.1331 | 5.4 | 1 | 2 | 1 | 1.02 | 2.0% |
| D4A7I6 | Protein RGD1309995 | 136.13 | 29.129 | 6 | 7 | 9 | 7 | 1.02 | 2.0% |
| F1M943 | Protein Armc8 | 75.432 | 27.5 | 9.8 | 5 | 6 | 5 | 1.02 | 1.4% |
| Q4FZU8 | Protein FAM65A | 131.73 | 30.386 | 3.7 | 5 | 6 | 5 | 1.02 | 1.4% |
| P62243 | 40S ribosomal protein S8 | 24.205 | 104.99 | 55.3 | 11 | 32 | 11 | 1.02 | 0.6% |
| P68101 | Eukaryotic translation initiation factor 2 subunit 1 | 36.108 | 175.87 | 55.6 | 17 | 34 | 17 | 1.02 | 1.8% |
| Q5U2U4 | Protein Scamp2 | 36.549 | 30.377 | 12.2 | 3 | 4 | 3 | 1.02 | 8.1% |
| D3ZUJ5 | Deoxythymidylate kinase (Predicted), isoform CRA_b | 23.973 | 10.153 | 14.2 | 3 | 3 | 3 | 1.02 | 1.5% |
| P97878 | Exocyst complex component 5 | 81.736 | 32.551 | 14.3 | 8 | 10 | 8 | 1.02 | 0.9% |
| A0A0G2K098 | Protein LOC100909795 | 91.129 | 12.135 | 3.8 | 3 | 3 | 3 | 1.02 | 3.3% |
| P09495 | Tropomyosin alpha-4 chain | 28.509 | 323.31 | 88.3 | 41 | 185 | 23 | 1.02 | 1.8% |
| O35987 | NSFL1 cofactor p47 | 40.679 | 153.48 | 55.1 | 16 | 23 | 16 | 1.02 | 0.1% |
| P11466 | Peroxisomal carnitine O-octanoyltransferase | 70.302 | 38.074 | 9.8 | 4 | 4 | 4 | 1.02 | 1.2% |
| D4A5I9 | Protein Myo6 | 148.13 | 121.99 | 16 | 17 | 21 | 17 | 1.02 | 1.7% |
| D3ZFS2 | Protein Clgn | 69.471 | 72.481 | 11.3 | 6 | 10 | 6 | 1.02 | 2.3% |
| O35509 | Ras-related protein Rab-11B | 24.488 | 86.57 | 47.2 | 9 | 21 | 9 | 1.02 | 3.0% |
| B0BN83 | Armadillo repeat-containing protein 1 | 31.204 | 6.9188 | 5.3 | 1 | 1 | 1 | 1.02 | 5.2% |
| G3V727 | DEAD (Asp-Glu-Ala-Asp) box polypeptide 47, isoform CRA_a | 50.696 | 8.9737 | 9 | 4 | 5 | 4 | 1.02 | 9.1% |
| Q4FZU0 | Acid phosphatase 6, lysophosphatidic | 47.28 | 62.22 | 26.4 | 9 | 10 | 9 | 1.02 | 10.3% |
| F1M805 | Protein Utp6 | 70.23 | 4.1912 | 2.7 | 1 | 1 | 1 | 1.02 | 5.7% |
| Q8VI02 | Serine/threonine-protein phosphatase 4 regulatory subunit 1 | 105.61 | 63.77 | 10.1 | 6 | 7 | 6 | 1.02 | 5.2% |
| D3ZXK7 | E3 ubiquitin-protein ligase RNF123 | 149.08 | 5.9217 | 1.8 | 3 | 3 | 3 | 1.02 | 4.5% |
| D3ZZ95 | 60S ribosomal protein L36 | 12.41 | 19.241 | 30.2 | 4 | 10 | 4 | 1.02 | 2.9% |
| P21531 | 60S ribosomal protein L3 | 46.135 | 113.83 | 48.1 | 20 | 49 | 20 | 1.02 | 1.4% |
| A0A0G2K8E5 | Protein Ints10 | 90.721 | 17.112 | 3.9 | 2 | 2 | 2 | 1.02 | 1.7% |
| P62703 | 40S ribosomal protein S4, X isoform | 29.597 | 108.96 | 61.2 | 18 | 45 | 18 | 1.02 | 2.4% |
| Q71LX6 | Xin actin-binding repeat-containing protein 2 | 373.98 | 2.8263 | 0.4 | 1 | 1 | 1 | 1.02 | 11.6% |
| P14056 | Serine/threonine-protein kinase A-Raf | 67.551 | 29.289 | 18.2 | 9 | 9 | 7 | 1.02 | 4.3% |
| A0A0G2QC15 | HIV-1 tat interactive protein 2, homolog (Human) (Predicted), isoform CRA_a | 30.01 | 27.084 | 14.5 | 3 | 3 | 3 | 1.02 | 1.7% |
| P83732 | 60S ribosomal protein L24 | 17.779 | 49.039 | 52.2 | 12 | 28 | 12 | 1.02 | 1.0% |
| Q5U2S7 | Proteasome (Prosome, macropain) 26S subunit, non-ATPase, 3 | 60.687 | 117.24 | 36.4 | 20 | 31 | 20 | 1.02 | 0.8% |
| Q00566 | Methyl-CpG-binding protein 2 | 53.047 | 18.859 | 7.9 | 3 | 3 | 3 | 1.02 | 0.2% |
| A0A0G2JU42 | Protein Eif2s3y | 51.153 | 20.402 | 34.3 | 15 | 23 | 4 | 1.02 | 0.6% |
| G3V8E2 | Protein Strip1 | 101.58 | 58.32 | 11.9 | 8 | 10 | 8 | 1.02 | 0.9% |
| Q5M920 | EBNA1 binding protein 2 | 34.744 | 15.856 | 16.9 | 5 | 5 | 5 | 1.02 | 2.3% |
| D4ADS6 | Protein Ints7 | 106.91 | 3.699 | 1.7 | 2 | 5 | 2 | 1.02 | 2.4% |
| Q3MID3 | ADP-ribosylation factor GTPase-activating protein 2 | 56.556 | 34.893 | 13.1 | 6 | 8 | 6 | 1.02 | 3.0% |
| P62836 | Ras-related protein Rap-1A | 20.987 | 18.873 | 51.1 | 10 | 23 | 3 | 1.02 | 3.6% |
| F1LWE6 | Protein Msi2 | 29.787 | 63.567 | 27.3 | 6 | 8 | 6 | 1.02 | 2.7% |
| P35427 | 60S ribosomal protein L13a | 23.476 | 26.289 | 35 | 8 | 24 | 8 | 1.02 | 2.6% |
| Q5XI64 | Monoacylglycerol lipase ABHD6 | 38.311 | 28.326 | 14.5 | 4 | 5 | 4 | 1.02 | 0.7% |
| Q7TSU1 | Brefeldin A-inhibited guanine nucleotide-exchange protein 2 | 201.97 | 37.094 | 8.6 | 13 | 17 | 6 | 1.02 | 2.1% |
| A0A0G2K1S9 | Protein Ccm2 | 49.83 | 5.1283 | 3.8 | 2 | 2 | 2 | 1.02 | 2.9% |
| P27653 | C-1-tetrahydrofolate synthase, cytoplasmic | 100.99 | 141.2 | 27.9 | 22 | 31 | 22 | 1.02 | 3.0% |
| P62916 | Transcription initiation factor IIB | 34.819 | 12.461 | 13.3 | 3 | 3 | 3 | 1.02 | 5.6% |
| Q641Y0 | Dolichyl-diphosphooligosaccharide--protein glycosyltransferase 48 kDa subunit | 48.895 | 81.347 | 28.6 | 12 | 25 | 12 | 1.02 | 2.2% |
| Q9R1Z0 | Voltage-dependent anion-selective channel protein 3 | 30.797 | 95.713 | 39.2 | 10 | 19 | 9 | 1.02 | 3.4% |
| D3ZXY2 | PDZ domain containing 8 (Predicted) | 128.25 | 5.2505 | 1.9 | 2 | 2 | 2 | 1.02 | 6.9% |
| F1M8L9 | Protein Rapgef1 | 139.61 | 8.0785 | 2.2 | 2 | 2 | 2 | 1.02 | 15.3% |
| D3Z8S0 | Protein Sh3d19 | 86.082 | 11.168 | 4.6 | 3 | 3 | 3 | 1.02 | 1.4% |
| B2RYD7 | Protein Stt3b | 93.435 | 41.556 | 12 | 13 | 20 | 13 | 1.02 | 1.2% |
| Q64560 | Tripeptidyl-peptidase 2 | 138.29 | 150.4 | 23.2 | 27 | 35 | 27 | 1.02 | 0.1% |
| Q9R1T3 | Cathepsin Z | 34.194 | 22.235 | 14.1 | 4 | 5 | 4 | 1.02 | 4.2% |
| D3ZW15 | Protein Sec24b | 134.71 | 75.456 | 10.4 | 10 | 12 | 10 | 1.02 | 2.1% |
| Q8K4F7 | m7GpppX diphosphatase | 38.713 | 50.235 | 17 | 4 | 7 | 4 | 1.02 | 1.5% |
| Q9QZ81 | Protein argonaute-2 | 97.317 | 64.394 | 15.2 | 10 | 12 | 10 | 1.02 | 0.5% |
| Q56A18 | SWI/SNF-related matrix-associated actin-dependent regulator of chromatin subfamily E member 1 | 42.831 | 23.35 | 5.9 | 2 | 3 | 2 | 1.02 | 14.4% |
| Q9WU61 | Chloride channel CLIC-like protein 1 | 61.173 | 14.317 | 10 | 5 | 5 | 5 | 1.02 | 7.3% |
| Q5XIB2 | Peptidyl-prolyl cis-trans isomerase CWC27 homolog | 53.309 | 26.733 | 5.8 | 2 | 3 | 2 | 1.02 | 4.1% |
| Q1JU68 | Eukaryotic translation initiation factor 3 subunit A | 163.19 | 275.09 | 40.4 | 61 | 98 | 61 | 1.02 | 2.2% |
| P17078 | 60S ribosomal protein L35 | 14.552 | 25.601 | 29.3 | 4 | 6 | 4 | 1.02 | 0.2% |
| Q6P777 | Multivesicular body subunit 12A | 28.752 | 21.045 | 10 | 2 | 3 | 2 | 1.02 | 0.6% |
| Q6AYQ9 | Peptidyl-prolyl cis-trans isomerase | 23.009 | 190.55 | 52.8 | 10 | 34 | 10 | 1.02 | 1.4% |
| Q641W2 | UPF0160 protein MYG1, mitochondrial | 42.888 | 10.728 | 7.3 | 2 | 2 | 2 | 1.02 | 6.9% |
| Q6AXM7 | HBS1-like protein | 74.773 | 124.99 | 23.4 | 11 | 14 | 11 | 1.01 | 1.8% |
| Q63356 | Unconventional myosin-Ie | 126.83 | 210.26 | 25.2 | 23 | 38 | 23 | 1.01 | 1.4% |
| Q63737 | Phosducin-like protein | 34.273 | 13.369 | 10.3 | 3 | 3 | 3 | 1.01 | 1.5% |
| Q5U2T3 | SPATS2-like protein | 61.774 | 30.338 | 16.1 | 8 | 8 | 8 | 1.01 | 4.0% |
| Q9Z2P6 | Synaptosomal-associated protein 29 | 29.07 | 14.083 | 12.1 | 3 | 4 | 3 | 1.01 | 2.0% |
| A0A0G2K999 | Protein Sdk2 | 239.88 | 13.941 | 1.6 | 3 | 4 | 3 | 1.01 | 1.7% |
| P62250 | 40S ribosomal protein S16 | 16.445 | 42.847 | 67.1 | 11 | 19 | 11 | 1.01 | 1.7% |
| Q6AYD3 | Proliferation-associated protein 2G4 | 43.656 | 247.21 | 53.6 | 23 | 39 | 23 | 1.01 | 1.6% |
| D4AD01 | Protein Zmat2 | 23.626 | 2.6357 | 5 | 1 | 2 | 1 | 1.01 | 2.0% |
| Q66HR0 | Solute carrier family 12 member 9 | 96.003 | 35.906 | 9.7 | 6 | 6 | 6 | 1.01 | 2.1% |
| P62425 | 60S ribosomal protein L7a | 29.995 | 164.71 | 51.5 | 17 | 35 | 14 | 1.01 | 2.7% |
| A1L1L2 | Transmembrane protein 214 | 76.565 | 49.372 | 16.1 | 9 | 9 | 9 | 1.01 | 3.3% |
| D3ZXJ5 | Protein Eftud1 | 125.69 | 77.914 | 9.6 | 9 | 11 | 9 | 1.01 | 6.4% |
| Q6PEB9 | Coiled-coil domain-containing protein 127 | 30.469 | 6.4596 | 8.5 | 2 | 2 | 2 | 1.01 | 7.3% |
| O88797 | Disabled homolog 2 | 82.376 | 50.779 | 10.3 | 6 | 8 | 6 | 1.01 | 3.2% |
| Q64057 | Alpha-aminoadipic semialdehyde dehydrogenase | 58.748 | 192.04 | 46.4 | 21 | 41 | 21 | 1.01 | 2.6% |
| Q6AYB5 | Signal recognition particle 54 kDa protein | 55.704 | 164.33 | 41.1 | 18 | 29 | 18 | 1.01 | 0.3% |
| P04041 | Glutathione peroxidase 1 | 22.305 | 61.736 | 60.7 | 10 | 17 | 10 | 1.01 | 1.5% |
| Q5PQX0 | UDP-glucuronic acid decarboxylase 1 | 47.538 | 37.438 | 11.7 | 3 | 5 | 3 | 1.01 | 2.9% |
| P15865 | Histone H1.4 | 21.987 | 59.45 | 26.9 | 10 | 21 | 2 | 1.01 | 3.0% |
| Q1AAU6 | Arf-GAP with SH3 domain, ANK repeat and PH domain-containing protein 1 | 127.09 | 59.821 | 7.9 | 6 | 6 | 6 | 1.01 | 7.3% |
| O70513 | Galectin-3-binding protein | 63.742 | 100.17 | 22.1 | 10 | 15 | 10 | 1.01 | 2.5% |
| Q32PX2 | Aminoacyl tRNA synthase complex-interacting multifunctional protein 2 | 35.442 | 42.126 | 21.6 | 6 | 9 | 6 | 1.01 | 2.0% |
| P23358 | 60S ribosomal protein L12 | 17.845 | 65.994 | 50.3 | 6 | 20 | 6 | 1.01 | 1.7% |
| Q0PMD2 | Anthrax toxin receptor 1 | 62.319 | 12.501 | 7.8 | 4 | 6 | 4 | 1.01 | 1.6% |
| A1L1I3 | Numb-like protein | 65.512 | 25.295 | 16.8 | 8 | 11 | 7 | 1.01 | 0.5% |
| Q3T1K5 | F-actin-capping protein subunit alpha-2 | 32.967 | 262.83 | 50.3 | 10 | 22 | 9 | 1.01 | 0.8% |
| Q5XI32 | F-actin-capping protein subunit beta | 30.628 | 78.957 | 54 | 15 | 38 | 14 | 1.01 | 0.8% |
| D3ZUV2 | Protein Mib1 | 110.1 | 28.479 | 4.9 | 4 | 4 | 4 | 1.01 | 7.9% |
| P63281 | SUMO-conjugating enzyme UBC9 | 18.007 | 29.821 | 26.6 | 5 | 5 | 5 | 1.01 | 13.1% |
| P69735 | Rab3 GTPase-activating protein catalytic subunit | 86.586 | 93.312 | 15.1 | 9 | 13 | 9 | 1.01 | 4.1% |
| B2GUV7 | Eukaryotic translation initiation factor 5B | 137.68 | 110.24 | 20.4 | 23 | 32 | 23 | 1.01 | 4.0% |
| G3V7S5 | Protein Senp3 | 64.398 | 13.24 | 3.7 | 2 | 2 | 2 | 1.01 | 2.3% |
| Q62812 | Myosin-9 | 226.34 | 323.31 | 64.4 | 174 | 899 | 149 | 1.01 | 0.7% |
| Q63648 | Merlin | 68.711 | 4.451 | 3.6 | 2 | 2 | 2 | 1.01 | 0.0% |
| Q62651 | Delta(3,5)-Delta(2,4)-dienoyl-CoA isomerase, mitochondrial | 36.171 | 55.571 | 33 | 9 | 12 | 9 | 1.01 | 0.7% |
| F1LQS0 | Protein Nmi | 24.611 | 10.383 | 7.4 | 1 | 1 | 1 | 1.01 | 1.2% |
| P13676 | Acylamino-acid-releasing enzyme | 81.383 | 64.216 | 14.9 | 9 | 13 | 9 | 1.01 | 3.0% |
| A0A0G2K548 | Protein Akap9 | 441.62 | 71.992 | 4.7 | 15 | 16 | 15 | 1.01 | 3.1% |
| Q5U2U3 | Poly (ADP-ribose) polymerase family, member 3 | 58.868 | 83.921 | 29.5 | 14 | 21 | 14 | 1.01 | 3.5% |
| G3V631 | Protein Rabgef1 | 58.139 | 20.726 | 10.6 | 5 | 6 | 5 | 1.01 | 6.9% |
| Q2QDE7 | Deoxyribonuclease-1-like 1 | 35.181 | 3.7154 | 4.2 | 1 | 1 | 1 | 1.01 | 4.2% |
| P29314 | 40S ribosomal protein S9 | 22.591 | 38.345 | 53.1 | 15 | 24 | 15 | 1.01 | 2.4% |
| P58200 | Vesicle transport through interaction with t-SNAREs homolog 1B | 26.703 | 53.051 | 32.3 | 6 | 7 | 6 | 1.01 | 1.6% |
| A0A0G2K931 | Protein Psat1 | 39.835 | 96.971 | 35.9 | 11 | 21 | 11 | 1.01 | 1.5% |
| Q6AXQ0 | SUMO-activating enzyme subunit 1 | 38.512 | 156.69 | 63 | 19 | 29 | 19 | 1.01 | 0.5% |
| P61980 | Heterogeneous nuclear ribonucleoprotein K | 50.976 | 92.615 | 31.3 | 15 | 34 | 15 | 1.01 | 0.1% |
| Q62824 | Exocyst complex component 4 | 110.55 | 123.9 | 21.1 | 16 | 19 | 16 | 1.01 | 0.0% |
| A0A0G2JZY3 | Protein Reps1 | 80.528 | 6.0296 | 2.8 | 2 | 2 | 1 | 1.01 | 0.2% |
| A0A0G2JTA1 | Protein Ppp2r5e | 54.713 | 52.548 | 15.6 | 6 | 9 | 6 | 1.01 | 1.6% |
| Q66HA6 | ADP-ribosylation factor-like protein 8B | 21.539 | 23.659 | 32.3 | 7 | 15 | 4 | 1.01 | 4.8% |
| Q32PZ0 | PAK1 interacting protein 1 | 42.234 | 6.4215 | 6.5 | 2 | 2 | 2 | 1.01 | 5.5% |
| A0A0G2K2P5 | Protein Tjp1 | 197.15 | 191.25 | 13.6 | 20 | 24 | 20 | 1.01 | 1.8% |
| F1LTD0 | Protein Gpr89b | 52.782 | 19.044 | 5.3 | 2 | 4 | 2 | 1.01 | 0.2% |
| D3ZK56 | Protein Rap2c | 20.745 | 36.283 | 29.5 | 4 | 7 | 2 | 1.01 | 20.1% |
| D3ZKG9 | Clustered mitochondria protein homolog | 151.16 | 4.8175 | 1.4 | 2 | 2 | 2 | 1.01 | 3.3% |
| A0A0G2K5A4 | Protein Golga3 | 162.36 | 323.31 | 27.8 | 31 | 38 | 31 | 1.01 | 2.7% |
| P54921 | Alpha-soluble NSF attachment protein | 33.192 | 112.71 | 48.5 | 12 | 18 | 12 | 1.01 | 1.9% |
| P34926 | Microtubule-associated protein 1A | 299.53 | 323.31 | 34.6 | 74 | 111 | 72 | 1.01 | 1.1% |
| D4AEP0 | Adenylosuccinate synthetase isozyme 2 | 50.085 | 152.86 | 36.8 | 14 | 28 | 14 | 1.01 | 1.3% |
| A0A0G2K0K9 | Protein Smoc2 | 51.124 | 8.9488 | 4.8 | 2 | 3 | 2 | 1.01 | 3.7% |
| P35281 | Ras-related protein Rab-10 | 22.858 | 15.795 | 27.5 | 7 | 11 | 5 | 1.01 | 2.8% |
| O55156 | CAP-Gly domain-containing linker protein 2 | 115.48 | 139.11 | 25.6 | 23 | 32 | 19 | 1.01 | 0.2% |
| B0BNG3 | Lman2 protein | 40.392 | 106.49 | 34.6 | 12 | 29 | 12 | 1.01 | 2.4% |
| D3Z9K4 | Protein Plekho2 | 54.007 | 65.975 | 16.8 | 8 | 12 | 8 | 1.01 | 7.6% |
| Q9QYJ2 | Ras GTPase-activating protein 3 | 96.019 | 17.992 | 5 | 4 | 4 | 4 | 1.01 | 3.2% |
| P62282 | 40S ribosomal protein S11 | 18.431 | 69.177 | 48.1 | 11 | 23 | 11 | 1.01 | 1.3% |
| G3V920 | Protein Wdr43 | 74.805 | 6.0258 | 4.5 | 3 | 3 | 3 | 1.01 | 0.8% |
| Q5XIJ6 | BRISC and BRCA1-A complex member 1 | 36.878 | 48.583 | 14.4 | 3 | 3 | 3 | 1.01 | 0.6% |
| G3V8Y8 | Huntingtin interacting protein 1, isoform CRA_a | 115 | 313.42 | 40.2 | 38 | 53 | 38 | 1.01 | 0.3% |
| P40329 | Arginine--tRNA ligase, cytoplasmic | 75.81 | 209.55 | 52.4 | 34 | 58 | 34 | 1.01 | 0.7% |
| Q9EPB1 | Dipeptidyl peptidase 2 | 55.114 | 109.06 | 34.6 | 13 | 26 | 13 | 1.01 | 0.8% |
| D3Z941 | Protein Mars | 101.58 | 238.06 | 38.9 | 30 | 43 | 30 | 1.01 | 0.9% |
| D4A4W9 | 60S ribosomal protein L34 | 13.392 | 11.144 | 28.2 | 4 | 8 | 4 | 1.01 | 1.6% |
| Q5U2X5 | Activated CDC42 kinase 1 | 115.07 | 2.6869 | 1.4 | 1 | 1 | 1 | 1.01 | 8.5% |
| D3Z9E1 | Elastin microfibril interfacer 1 (Predicted), isoform CRA_b | 94.504 | 19.374 | 5.6 | 4 | 4 | 4 | 1.01 | 5.0% |
| Q4V7C6 | GMP synthase [glutamine-hydrolyzing] | 76.757 | 49.412 | 14.6 | 9 | 10 | 9 | 1.01 | 2.9% |
| D3Z9D2 | FYVE and coiled-coil domain containing 1 (Predicted) | 163.55 | 13.204 | 2.5 | 3 | 4 | 3 | 1.01 | 2.8% |
| Q62667 | Major vault protein | 95.797 | 323.31 | 46.3 | 32 | 46 | 32 | 1.01 | 2.5% |
| Q9QZR6 | Septin-9 | 63.791 | 175.24 | 52.1 | 30 | 60 | 30 | 1.01 | 1.2% |
| D3Z9P1 | Follicular lymphoma variant translocation 1 (Predicted), isoform CRA_a | 36.036 | 34.123 | 18.4 | 7 | 10 | 7 | 1.01 | 0.8% |
| D3ZK76 | Protein RGD1559909 | 11.322 | 6.7764 | 10.9 | 1 | 1 | 1 | 1.01 | 2.2% |
| D4A9L2 | Protein Srsf1 | 27.744 | 59.301 | 40.3 | 11 | 20 | 11 | 1.01 | 2.4% |
| Q9ER30 | Kelch-like protein 41 | 68.212 | 2.829 | 1.7 | 1 | 1 | 1 | 1.01 | 16.6% |
| P62912 | 60S ribosomal protein L32 | 15.86 | 47.699 | 51.9 | 10 | 17 | 2 | 1.01 | 4.5% |
| Q63184 | Interferon-induced, double-stranded RNA-activated protein kinase | 58.258 | 27.357 | 11.7 | 5 | 5 | 5 | 1.01 | 2.1% |
| D4A3E1 | Protein Hnrnpll | 64.362 | 64.902 | 20.1 | 9 | 12 | 9 | 1.01 | 0.0% |
| D3ZNV6 | Protein Elmod2 | 34.783 | 15.576 | 12.6 | 4 | 4 | 4 | 1.01 | 0.1% |
| D4A8H5 | Protein Ppp4r2 | 45.943 | 44.209 | 16.2 | 5 | 5 | 5 | 1.01 | 1.5% |
| Q4KM64 | Protein jagunal homolog 1 | 21.102 | 31.172 | 14.2 | 4 | 5 | 4 | 1.01 | 4.5% |
| B5DEJ5 | Eefsec protein | 63.659 | 6.7005 | 1.9 | 1 | 1 | 1 | 1.01 | 7.4% |
| B0BN94 | Protein FAM136A | 15.625 | 25.147 | 55.1 | 6 | 10 | 6 | 1.01 | 9.3% |
| Q6AYC2 | Immunity-related GTPase family M protein | 46.337 | 19.332 | 9.2 | 3 | 3 | 3 | 1.01 | 7.1% |
| B5DFL9 | Protein Sestd1 | 79.421 | 11.786 | 6.2 | 4 | 4 | 4 | 1.01 | 4.9% |
| A0A0G2K2J9 | Protein Ctage5 | 91.074 | 59.716 | 13 | 8 | 11 | 8 | 1.01 | 3.2% |
| A0A096MJX5 | Protein RGD1305178 | 33.543 | 13.718 | 11.4 | 3 | 3 | 3 | 1.01 | 2.3% |
| Q5M853 | KxDL motif-containing protein 1 | 19.913 | 35.909 | 22.6 | 3 | 4 | 3 | 1.01 | 1.4% |
| A0A0G2K8D1 | Protein Mospd2 | 59.742 | 7.8247 | 4.1 | 2 | 2 | 2 | 1.01 | 1.1% |
| F7EXQ7 | Protein Ndufa8 | 22.122 | 16.418 | 17.4 | 3 | 6 | 3 | 1.01 | 0.1% |
| Q641X3 | Beta-hexosaminidase subunit alpha | 60.537 | 98.46 | 24.1 | 12 | 27 | 12 | 1.01 | 0.2% |
| P17074 | 40S ribosomal protein S19 | 16.085 | 49.682 | 54.5 | 11 | 20 | 11 | 1.01 | 0.8% |
| Q9Z1A6 | Vigilin | 141.58 | 323.31 | 43.9 | 51 | 91 | 51 | 1.01 | 1.1% |
| A0A0G2JTI7 | Protein Prpf3 | 77.44 | 44.23 | 13.2 | 7 | 9 | 7 | 1.01 | 1.2% |
| O08662 | Phosphatidylinositol 4-kinase alpha | 231.32 | 71.383 | 7.4 | 14 | 15 | 14 | 1.01 | 1.4% |
| D3ZHA7 | Protein Myl6b | 22.807 | 3.988 | 12.6 | 2 | 9 | 1 | 1.01 | 3.8% |
| M0RCY2 | 40S ribosomal protein S13 | 17.773 | 54.169 | 58.7 | 10 | 23 | 10 | 1.01 | 4.3% |
| D4A3P1 | Protein Ubqln4 | 63.55 | 25.791 | 8.6 | 3 | 3 | 3 | 1.01 | 6.7% |
| D4A2F6 | Protein Nhlrc3 | 42.636 | 65.597 | 26.2 | 8 | 12 | 8 | 1.01 | 5.3% |
| Q62627 | PRKC apoptosis WT1 regulator protein | 35.865 | 197.78 | 54.5 | 15 | 34 | 15 | 1.01 | 2.1% |
| D3ZGS5 | Protein Uaca | 160.86 | 89.994 | 10.1 | 12 | 13 | 12 | 1.01 | 1.7% |
| F1M9C0 | Protein Mapkapk2 | 42.504 | 10.345 | 10 | 5 | 5 | 5 | 1.01 | 0.9% |
| F1M0Z1 | Protein Trio | 343.87 | 27.198 | 3 | 8 | 8 | 8 | 1.01 | 0.7% |
| P42346 | Serine/threonine-protein kinase mTOR | 288.79 | 82.206 | 4.5 | 9 | 12 | 9 | 1.01 | 0.2% |
| P04797 | Glyceraldehyde-3-phosphate dehydrogenase | 35.828 | 285.18 | 63.4 | 20 | 118 | 10 | 1.01 | 0.3% |
| D3ZWC6 | Protein Sntb1 | 58.292 | 20.992 | 13.2 | 8 | 9 | 8 | 1.01 | 4.2% |
| Q91XU1 | Protein quaking | 37.642 | 36.961 | 15 | 3 | 4 | 3 | 1.01 | 9.0% |
| D3ZXA6 | Protein Pdpr | 98.818 | 50.117 | 9.2 | 7 | 8 | 7 | 1.01 | 5.3% |
| A0A0G2K994 | Protein Vsig10 | 60.703 | 5.3555 | 2.1 | 1 | 1 | 1 | 1.01 | 3.8% |
| Q63347 | 26S protease regulatory subunit 7 | 48.574 | 191.16 | 54.5 | 23 | 48 | 23 | 1.01 | 2.0% |
| O55012 | Phosphatidylinositol-binding clathrin assembly protein | 69.285 | 196.27 | 27.7 | 15 | 26 | 15 | 1.01 | 1.3% |
| D3ZQC6 | Protein Ubr1 | 150.9 | 67.804 | 5.5 | 5 | 5 | 5 | 1.01 | 0.1% |
| G3V982 | Engulfment and cell motility 2, ced-12 homolog (C. elegans), isoform CRA_b | 83.742 | 51.472 | 17.3 | 10 | 12 | 10 | 1.01 | 0.7% |
| G3V762 | Protein Tsta3 | 35.796 | 88.264 | 29 | 7 | 12 | 7 | 1.01 | 0.8% |
| D3ZMY7 | Protein Nt5c2 | 67.76 | 43.432 | 11.3 | 6 | 7 | 6 | 1.01 | 1.0% |
| D4ABM5 | Mitochondrial ribosomal protein S34 (Predicted), isoform CRA_a | 25.799 | 5.481 | 10.1 | 3 | 3 | 3 | 1.01 | 1.1% |
| Q66H59 | N-acetylneuraminate lyase | 35.115 | 83.404 | 23.8 | 6 | 11 | 6 | 1.01 | 3.5% |
| M0RCF3 | Integrin beta | 87.083 | 6.0298 | 3.2 | 2 | 2 | 2 | 1.01 | 4.0% |
| P62749 | Hippocalcin-like protein 1 | 22.338 | 83.501 | 54.9 | 12 | 28 | 5 | 1.01 | 6.3% |
| F1LYS7 | Protein Sgcd | 32.087 | 18.401 | 26 | 7 | 8 | 7 | 1.01 | 13.4% |
| Q6AYE0 | Protein Tmbim1 | 34.294 | 5.6714 | 4.9 | 2 | 2 | 2 | 1.01 | 2.4% |
| Q5XI79 | NADH dehydrogenase [ubiquinone] complex I, assembly factor 7 | 48.715 | 15.47 | 9.2 | 3 | 4 | 3 | 1.01 | 1.8% |
| Q4KLL7 | Protein Vps4b | 49.451 | 25.597 | 12.8 | 5 | 5 | 3 | 1.01 | 1.6% |
| Q01460 | Di-N-acetylchitobiase | 41.531 | 77.14 | 29.4 | 9 | 13 | 9 | 1.01 | 1.2% |
| Q06486 | Casein kinase I isoform delta | 47.316 | 32.045 | 12.5 | 4 | 4 | 4 | 1.01 | 1.6% |
| D3ZQ02 | Protein Wdr37 | 49.872 | 12.654 | 6.2 | 3 | 4 | 3 | 1.01 | 5.0% |
| D4ACY1 | Protein RGD1565784 | 15.658 | 11.957 | 18.4 | 1 | 1 | 1 | 1.01 | 12.3% |
| A0A0G2K9L9 | Protein Mdn1 | 473.75 | 6.847 | 0.6 | 2 | 2 | 2 | 1.01 | 0.7% |
| O88884 | A-kinase anchor protein 1, mitochondrial | 91.746 | 18.048 | 4.6 | 3 | 3 | 3 | 1.01 | 15.9% |
| P08644 | GTPase KRas | 21.656 | 11.892 | 32.8 | 5 | 9 | 2 | 1.01 | 16.7% |
| F1LRS8 | CD2-associated protein | 70.49 | 12.964 | 6.6 | 4 | 5 | 3 | 1.01 | 5.0% |
| A0A0G2K0P0 | Protein Akap8l | 71.15 | 14.374 | 3.9 | 2 | 2 | 2 | 1.01 | 2.6% |
| Q6P6S9 | Ectonucleoside triphosphate diphosphohydrolase 5 | 47.372 | 79.471 | 21.8 | 6 | 11 | 6 | 1.01 | 3.4% |
| Q62825 | Exocyst complex component 3 | 86.496 | 38.4 | 8.3 | 5 | 6 | 5 | 1.01 | 9.5% |
| Q6P9T8 | Tubulin beta-4B chain | 49.8 | 79.878 | 61.8 | 24 | 148 | 1 | 1.01 | 7.2% |
| D3ZAF7 | Protein Tbc1d2b | 96.558 | 41.432 | 8.8 | 6 | 6 | 6 | 1.01 | 5.9% |
| P84092 | AP-2 complex subunit mu | 49.654 | 139.83 | 42.5 | 19 | 37 | 19 | 1.01 | 2.6% |
| P00786 | Pro-cathepsin H | 37.104 | 25.313 | 16.2 | 4 | 7 | 4 | 1.01 | 0.7% |
| A0A0G2K386 | Protein Parn | 73.383 | 13.586 | 3.9 | 2 | 2 | 2 | 1.01 | 1.4% |
| P0C5W1 | Microtubule-associated protein 1S | 102.8 | 63.309 | 13.6 | 11 | 14 | 11 | 1.01 | 2.9% |
| A0A0G2K618 | Protein Large | 87.979 | 3.278 | 1.7 | 1 | 1 | 1 | 1.01 | 7.6% |
| P35738 | 2-oxoisovalerate dehydrogenase subunit beta, mitochondrial | 42.823 | 59.792 | 16.7 | 5 | 8 | 5 | 1.01 | 7.8% |
| A0JPJ0 | Nicotinamide-nucleotide adenylyltransferase | 32.534 | 4.3817 | 6.3 | 1 | 1 | 1 | 1.01 | 7.5% |
| P12368 | cAMP-dependent protein kinase type II-alpha regulatory subunit | 45.54 | 190.91 | 49.1 | 16 | 41 | 14 | 1.01 | 2.0% |
| D4A1J6 | Protein Ankfy1 | 128.66 | 47.668 | 7.2 | 9 | 10 | 9 | 1.01 | 0.9% |
| M0RC99 | Ras-related protein Rab-5A | 23.624 | 79.173 | 47.4 | 7 | 14 | 4 | 1.01 | 1.6% |
| F1LZ81 | Protein Dock4 | 224.99 | 10.914 | 2.6 | 5 | 5 | 3 | 1.01 | 4.1% |
| D3ZCP9 | Transcription initiation factor IIE subunit beta | 32.959 | 18.865 | 14.1 | 4 | 4 | 4 | 1.01 | 0.1% |
| Q5PQX1 | Torsin-1A-interacting protein 1 | 65.648 | 186.66 | 31.6 | 13 | 23 | 13 | 1.01 | 2.3% |
| E9PT85 | Protein Xrcc6 | 69.588 | 33.711 | 11.2 | 6 | 7 | 6 | 1.01 | 0.7% |
| G3V965 | Agpat5 protein | 41.531 | 18.697 | 16.7 | 6 | 6 | 6 | 1.01 | 0.4% |
| Q499V0 | COMM domain containing 7 | 22.595 | 9.5146 | 9.5 | 2 | 2 | 2 | 1.01 | 0.0% |
| Q8K3P6 | Calcium-binding mitochondrial carrier protein SCaMC-2 | 52.694 | 2.8641 | 4.1 | 2 | 2 | 2 | 1.01 | 0.1% |
| Q9JIM0 | Double-strand break repair protein MRE11A | 80.121 | 36.855 | 10.6 | 7 | 7 | 7 | 1.01 | 1.4% |
| Q499N3 | WD repeat-containing protein 18 | 47.225 | 47.402 | 13.9 | 4 | 4 | 4 | 1.01 | 1.8% |
| Q5XI01 | La-related protein 7 | 64.949 | 7.329 | 3.9 | 2 | 2 | 2 | 1.01 | 1.9% |
| F1MAJ2 |  | 138.09 | 152.1 | 19.4 | 18 | 30 | 18 | 1.01 | 2.0% |
| D3ZRC4 | Protein Pnpla8 | 87.96 | 23.154 | 7.6 | 5 | 5 | 5 | 1.01 | 6.9% |
| G3V7N6 | Protein LOC100909580 | 53.379 | 17.858 | 5.1 | 2 | 2 | 2 | 1.01 | 4.3% |
| A0A0G2K402 | Protein Myef2 | 63.375 | 36.234 | 12.7 | 7 | 8 | 7 | 1.01 | 3.3% |
| B0BNE3 | Protein Trappc5 | 20.795 | 6.6434 | 12.2 | 2 | 2 | 2 | 1.01 | 1.2% |
| B2RYJ3 | Protein Cul4a | 87.773 | 58.949 | 15.5 | 11 | 13 | 7 | 1.01 | 1.1% |
| Q01714 | Transcription factor Sp1 | 80.771 | 2.6189 | 1.1 | 1 | 1 | 1 | 1.01 | 0.1% |
| B1H230 | Map2k3 protein | 39.31 | 24.938 | 10.7 | 3 | 5 | 3 | 1.01 | 0.2% |
| Q9QYG8 | Uridine-cytidine kinase 2 | 29.404 | 29.67 | 16.9 | 3 | 3 | 3 | 1.01 | 0.4% |
| D4AAT7 | ATP-dependent (S)-NAD(P)H-hydrate dehydratase | 36.518 | 17.826 | 11.1 | 3 | 4 | 3 | 1.01 | 0.5% |
| P04550 | Parathymosin | 11.559 | 16.935 | 23.5 | 3 | 4 | 3 | 1.01 | 2.4% |
| G3V8C0 | Protein Dctn5 | 20.14 | 11.907 | 13.7 | 3 | 3 | 3 | 1.01 | 2.5% |
| Q63270 | Cytoplasmic aconitate hydratase | 98.126 | 70.068 | 17.1 | 12 | 15 | 12 | 1.01 | 3.5% |
| Q56R17 | Importin subunit alpha | 57.922 | 115 | 32.2 | 13 | 16 | 7 | 1.01 | 10.9% |
| Q3KR97 | Brain-specific angiogenesis inhibitor 1-associated protein 2-like protein 1 | 57.468 | 22.829 | 9.7 | 5 | 5 | 5 | 1.00 | 2.7% |
| D3ZCI5 | Protein Stxbp6 | 23.671 | 10.387 | 22.9 | 5 | 5 | 5 | 1.00 | 0.1% |
| Q9WTV0 | Prolactin regulatory element-binding protein | 45.357 | 49.091 | 20.6 | 6 | 7 | 6 | 1.00 | 1.6% |
| P40241 | CD9 antigen | 25.215 | 22.916 | 25.2 | 6 | 7 | 6 | 1.00 | 2.3% |
| P12749 | 60S ribosomal protein L26 | 17.277 | 35.126 | 39.3 | 10 | 26 | 10 | 1.00 | 3.9% |
| Q5XIM7 | Lysine--tRNA ligase | 71.622 | 130.54 | 32.9 | 20 | 32 | 20 | 1.00 | 1.2% |
| Q920A6 | Retinoid-inducible serine carboxypeptidase | 51.174 | 122.82 | 31 | 13 | 42 | 13 | 1.00 | 0.9% |
| Q62908 | Cysteine and glycine-rich protein 2 | 20.94 | 150.27 | 51.8 | 11 | 26 | 11 | 1.00 | 0.3% |
| D3ZA84 | Protein Tln2 | 271.68 | 323.31 | 29.5 | 62 | 92 | 43 | 1.00 | 0.0% |
| Q4KLK8 | PX domain-containing protein 1 | 26.359 | 5.4715 | 4.8 | 1 | 2 | 1 | 1.00 | 5.0% |
| Q63488 | Sodium-dependent phosphate transporter 2 | 70.747 | 2.6793 | 1.7 | 1 | 1 | 1 | 1.00 | 0.8% |
| Q6IRK9 | Carboxypeptidase Q | 52.041 | 53.665 | 21.2 | 8 | 15 | 8 | 1.00 | 4.0% |
| P05696 | Protein kinase C alpha type | 76.791 | 53.095 | 19.5 | 11 | 15 | 11 | 1.00 | 3.9% |
| P53534 | Glycogen phosphorylase, brain form | 96.173 | 99.227 | 19.6 | 15 | 23 | 13 | 1.00 | 3.1% |
| Q3T1L0 | Aldehyde dehydrogenase family 16 member A1 | 85.413 | 84.118 | 17.1 | 11 | 14 | 11 | 1.00 | 2.5% |
| Q68A21 | Transcriptional activator protein Pur-beta | 33.418 | 54.358 | 33.7 | 8 | 12 | 8 | 1.00 | 1.3% |
| D3ZUV0 | Protein LOC100910429 | 131.05 | 1.945 | 1.7 | 2 | 2 | 2 | 1.00 | 0.5% |
| Q0D2L6 | Protein Rragc | 44.106 | 62.139 | 24.9 | 8 | 13 | 8 | 1.00 | 2.1% |
| D3ZUY8 | Adaptor protein complex AP-2, alpha 1 subunit (Predicted) | 107.67 | 170.91 | 33.8 | 30 | 67 | 23 | 1.00 | 2.3% |
| Q91ZW6 | Trimethyllysine dioxygenase, mitochondrial | 49.567 | 17.678 | 12.1 | 5 | 5 | 5 | 1.00 | 2.9% |
| A0A0G2JYU3 | Protein Astn2 | 149.57 | 2.2817 | 0.5 | 1 | 1 | 1 | 1.00 | 4.9% |
| A0A0G2JX94 | Protein Amot | 127.73 | 8.5373 | 3.5 | 3 | 4 | 1 | 1.00 | 2.4% |
| D4A644 | Protein Map7d1 | 90.418 | 61.31 | 16.9 | 11 | 19 | 11 | 1.00 | 0.2% |
| D4A7U1 | Protein Zyx | 60.303 | 307.91 | 53.4 | 22 | 62 | 20 | 1.00 | 0.2% |
| P13471 | 40S ribosomal protein S14 | 16.259 | 81.195 | 41.7 | 9 | 22 | 9 | 1.00 | 0.6% |
| Q5M949 | Nipsnap homolog 3A (C. elegans) | 28.34 | 34.47 | 17.4 | 3 | 5 | 3 | 1.00 | 3.2% |
| F7FF45 | Protein Numa1 | 234.94 | 111.23 | 9.1 | 17 | 19 | 17 | 1.00 | 2.6% |
| A0A0G2KAI2 | Protein Phf2 | 118.2 | 9.182 | 3.5 | 4 | 4 | 4 | 1.00 | 3.7% |
| Q63159 | Hexaprenyldihydroxybenzoate methyltransferase, mitochondrial | 38.708 | 15.48 | 12.5 | 3 | 3 | 3 | 1.00 | 6.3% |
| P15651 | Short-chain specific acyl-CoA dehydrogenase, mitochondrial | 44.765 | 47.91 | 18 | 6 | 9 | 6 | 1.00 | 7.2% |
| Q9JKL7 | Splicing regulatory glutamine/lysine-rich protein 1 | 56.849 | -2 | 2 | 1 | 1 | 1 | 1.00 | 5.2% |
| Q4AE70 | Histone-arginine methyltransferase CARM1 | 70.34 | 23.707 | 6.9 | 4 | 4 | 4 | 1.00 | 4.7% |
| B5DES0 | Protein Snrpd2 | 13.527 | 80.571 | 61.9 | 9 | 17 | 9 | 1.00 | 3.4% |
| B0BN82 | Brix domain containing 1 | 35.256 | 4.8611 | 5.6 | 1 | 1 | 1 | 1.00 | 2.8% |
| P84903 | Stromal interaction molecule 1 | 77.448 | 33.796 | 10.2 | 6 | 8 | 6 | 1.00 | 0.9% |
| Q75WE7 | von Willebrand factor A domain-containing protein 5A | 91.481 | 90.371 | 19.6 | 15 | 19 | 15 | 1.00 | 0.9% |
| Q63312 | Pleckstrin homology-like domain family B member 1 | 93.54 | 55.077 | 16.5 | 10 | 11 | 10 | 1.00 | 2.3% |
| Q9ES71 | Dihydroxyacetone phosphate acyltransferase | 77.075 | 7.5876 | 2.4 | 1 | 1 | 1 | 1.00 | 2.4% |
| D3ZVT2 | Protein Thada | 217.24 | 18.891 | 2.3 | 4 | 4 | 4 | 1.00 | 4.8% |
| A0A0G2JUI5 | Protein Clasp1 | 170.81 | 11.179 | 3.7 | 5 | 5 | 4 | 1.00 | 11.0% |
| Q505J9 | ATPase family AAA domain-containing protein 1 | 40.717 | 23.878 | 15.2 | 5 | 6 | 5 | 1.00 | 17.1% |
| D3ZKI9 | Protein Nop9 | 69.701 | 16.133 | 2.4 | 1 | 2 | 1 | 1.00 | 25.5% |
| Q5XIF4 | Small ubiquitin-related modifier 3 | 12.456 | 3.2917 | 19.1 | 2 | 4 | 1 | 1.00 | 6.6% |
| A0A0G2JTX2 | Protein Praf2 | 19.078 | 23.635 | 20.8 | 5 | 10 | 5 | 1.00 | 2.5% |
| D3Z863 | CWF19-like 1, cell cycle control (S. pombe) (Predicted) | 60.377 | 3.1129 | 4.7 | 2 | 2 | 2 | 1.00 | 1.8% |
| Q6AYB2 | Protein Sphk2 | 65.522 | 16.156 | 5.5 | 3 | 3 | 3 | 1.00 | 1.7% |
| P24050 | 40S ribosomal protein S5 | 22.878 | 26.513 | 31.9 | 7 | 14 | 7 | 1.00 | 1.5% |
| P27008 | Poly [ADP-ribose] polymerase 1 | 112.66 | 35.621 | 6.1 | 5 | 6 | 5 | 1.00 | 1.2% |
| Q02253 | Methylmalonate-semialdehyde dehydrogenase [acylating], mitochondrial | 57.807 | 180.23 | 42.4 | 17 | 29 | 17 | 1.00 | 2.9% |
| B1WBS4 | Protein Vps26b | 39.124 | 60.612 | 33 | 10 | 18 | 10 | 1.00 | 3.0% |
| P50393 | Cytosolic phospholipase A2 | 85.706 | 41.486 | 12.6 | 9 | 12 | 9 | 1.00 | 3.3% |
| Q6AY90 | NEFA-interacting nuclear protein NIP30 | 28.575 | 4.1965 | 12.6 | 3 | 3 | 3 | 1.00 | 4.2% |
| P60517 | Gamma-aminobutyric acid receptor-associated protein | 13.918 | 5.5739 | 9.4 | 1 | 1 | 1 | 1.00 | 6.7% |
| Q4V8B2 | DCN1-like protein 3 | 34.35 | 24.981 | 16.4 | 4 | 4 | 4 | 1.00 | 11.0% |
| Q6XLI7 | Protein Rbm12 | 106.26 | 13.561 | 4.2 | 5 | 6 | 5 | 1.00 | 3.4% |
| A0A0G2JYP0 |  | 7.8971 | 8.3891 | 19.7 | 1 | 2 | 1 | 1.00 | 2.7% |
| P23928 | Alpha-crystallin B chain | 20.089 | 105.5 | 57.1 | 11 | 41 | 11 | 1.00 | 0.8% |
| Q9EQG6 | Kinase D-interacting substrate of 220 kDa | 195.71 | 9.7389 | 2 | 4 | 4 | 4 | 1.00 | 0.9% |
| O08697 | ADP-ribosylation factor-like protein 2 | 20.836 | 39.082 | 33.2 | 5 | 10 | 5 | 1.00 | 1.0% |
| O35274 | Neurabin-2 | 89.645 | 48.132 | 14.3 | 8 | 8 | 8 | 1.00 | 1.3% |
| D3ZW58 | Protein Casc4 | 52.325 | 10.37 | 7.8 | 3 | 3 | 3 | 1.00 | 4.8% |
| B2GUV5 | ATPase, H transporting, lysosomal V1 subunit G1 | 13.71 | 14.998 | 11.9 | 2 | 3 | 2 | 1.00 | 7.1% |
| P97700 | Mitochondrial 2-oxoglutarate/malate carrier protein | 34.133 | 44.712 | 29.9 | 9 | 13 | 9 | 1.00 | 4.8% |
| Q5XIP1 | Protein pelota homolog | 43.407 | 72.889 | 19.7 | 8 | 9 | 8 | 1.00 | 4.0% |
| Q4KLN7 | ADP-ribosylation factor GTPase-activating protein 3 | 57.67 | 81.43 | 22.1 | 12 | 13 | 12 | 1.00 | 1.8% |
| Q2TL32 | E3 ubiquitin-protein ligase UBR4 | 573.79 | 174.81 | 4.8 | 20 | 22 | 20 | 1.00 | 0.0% |
| P54275 | DNA mismatch repair protein Msh2 | 104.03 | 7.2224 | 2.8 | 2 | 3 | 2 | 1.00 | 0.1% |
| Q07647 | Solute carrier family 2, facilitated glucose transporter member 3 | 53.58 | 11.087 | 6.5 | 3 | 3 | 3 | 1.00 | 0.8% |
| Q5I0C3 | Methylcrotonoyl-CoA carboxylase subunit alpha, mitochondrial | 79.329 | 44.49 | 8 | 4 | 6 | 4 | 1.00 | 4.5% |
| D3ZGR7 | Protein LOC100910447 | 18.472 | 1.8645 | 5.8 | 1 | 1 | 1 | 1.00 | 9.7% |
| D3ZEI0 | Protein LOC690096 | 16.552 | 48.401 | 45.1 | 9 | 22 | 2 | 1.00 | 5.8% |
| F1M7B8 | Protein Ube3a | 99.752 | 71.914 | 17.5 | 12 | 13 | 12 | 1.00 | 4.4% |
| D4A3U2 | Tetraspanin | 27.787 | 10.061 | 7.3 | 2 | 3 | 2 | 1.00 | 1.1% |
| Q99PF5 | Far upstream element-binding protein 2 | 74.226 | 148.9 | 33.4 | 20 | 30 | 17 | 1.00 | 0.8% |
| P25286 | V-type proton ATPase 116 kDa subunit a isoform 1 | 96.327 | 74.642 | 14.2 | 9 | 15 | 9 | 1.00 | 0.0% |
| Q6AY02 | Protein Rbm17 | 45.347 | 17.877 | 4.2 | 1 | 1 | 1 | 1.00 | 3.1% |
| Q4QQT6 | Ribosome biogenesis protein BRX1 homolog | 41.114 | 23.397 | 10.2 | 4 | 5 | 4 | 1.00 | 3.7% |
| P84586 | RNA-binding motif protein, X chromosome retrogene-like | 42.203 | 5.9347 | 29.4 | 13 | 24 | 3 | 1.00 | 4.1% |
| Q498D9 | PDZ domain-containing protein GIPC2 | 34.202 | 29.916 | 5.4 | 1 | 1 | 1 | 1.00 | 5.7% |
| M0RDI1 | Glutathione S-transferase | 21.148 | 10.433 | 26.6 | 4 | 6 | 2 | 1.00 | 11.0% |
| P62944 | AP-2 complex subunit beta | 104.55 | 296.18 | 37.9 | 35 | 71 | 17 | 1.00 | 0.5% |
| Q5U1Y1 | Ras-related protein Rab-34 | 29.102 | 20.75 | 20.8 | 5 | 5 | 5 | 1.00 | 0.1% |
| D3ZD29 | Protein Ado | 22.226 | 15.344 | 18.2 | 3 | 3 | 3 | 1.00 | 1.1% |
| Q9QUR2 | Dynactin subunit 4 | 53.088 | 86.669 | 27.6 | 8 | 12 | 8 | 1.00 | 1.1% |
| P40615 | H/ACA ribonucleoprotein complex subunit 4 | 56.614 | 37.128 | 13 | 8 | 11 | 8 | 1.00 | 2.6% |
| P35280 | Ras-related protein Rab-8A | 23.668 | 29.556 | 33.8 | 7 | 13 | 2 | 1.00 | 1.6% |
| A0A0G2JX45 | Protein Ate1 | 58.7 | 84.331 | 27.7 | 12 | 19 | 12 | 1.00 | 2.2% |
| Q8R500 | Mitofusin-2 | 86.122 | 43.88 | 10.7 | 8 | 8 | 8 | 1.00 | 7.7% |
| A0A0G2K860 | Protein Arhgef12 | 171.52 | 16.924 | 3.8 | 5 | 5 | 5 | 1.00 | 6.5% |
| A0A0G2JWZ2 |  | 151.65 | 47.165 | 6.6 | 8 | 10 | 8 | 1.00 | 3.1% |
| Q498R7 | UPF0587 protein C1orf123 homolog | 18.094 | 27.405 | 33.1 | 4 | 5 | 4 | 1.00 | 1.7% |
| Q9Z340 | Partitioning defective 3 homolog | 149.45 | 12.458 | 3.8 | 5 | 7 | 5 | 1.00 | 0.5% |
| D3ZZN4 | 60S ribosomal protein L35a | 12.623 | 27.547 | 48.2 | 8 | 15 | 8 | 1.00 | 2.3% |
| Q9JHW0 | Proteasome subunit beta type-7 | 29.927 | 15.577 | 15.9 | 4 | 6 | 4 | 1.00 | 3.6% |
| Q6AZ61 | Probable lysosomal cobalamin transporter | 61.018 | 8.143 | 5.2 | 2 | 2 | 2 | 1.00 | 1.2% |
| Q4V8E2 | Proteasome (Prosome, macropain) 26S subunit, non-ATPase, 14 | 34.577 | 58.719 | 34.5 | 8 | 15 | 8 | 1.00 | 1.0% |
| P97532 | 3-mercaptopyruvate sulfurtransferase | 32.94 | 56.33 | 37.7 | 8 | 12 | 8 | 1.00 | 5.4% |
| Q5RJZ6 | Short coiled-coil protein | 13.689 | 21.464 | 23 | 2 | 4 | 2 | 1.00 | 5.4% |
| D3ZFF4 | Protein Lclat1 | 44.566 | 9.092 | 7.4 | 3 | 3 | 3 | 1.00 | 6.6% |
| D3ZKC6 | Protein Vps13d | 488.95 | 7.5578 | 1 | 5 | 5 | 5 | 1.00 | 5.1% |
| D4A5W8 | Protein Pgs1 | 57.755 | 16.832 | 8.4 | 4 | 4 | 4 | 1.00 | 3.8% |
| D3ZNF4 | Protein Tbl1xr1 | 55.67 | 16.308 | 7.4 | 3 | 3 | 3 | 1.00 | 2.4% |
| A0A0G2JTX7 | Protein Col6a5 | 288.98 | 4.1547 | 0.8 | 2 | 2 | 2 | 1.00 | 1.8% |
| Q6AXT5 | Ras-related protein Rab-21 | 24.163 | 63.014 | 39.9 | 8 | 13 | 8 | 1.00 | 1.1% |
| Q5XIC1 | Mannose-1-phosphate guanyltransferase alpha | 46.191 | 52.503 | 16.9 | 5 | 7 | 5 | 1.00 | 0.9% |
| B2RYJ7 | ARP1 actin-related protein 1 homolog B (Yeast) | 42.281 | 40.801 | 37.5 | 12 | 31 | 4 | 1.00 | 1.4% |
| D4AEG3 | Protein Ppil4 | 57.26 | 4.6359 | 5.3 | 2 | 2 | 2 | 1.00 | 2.3% |
| Q6XVN8 | Microtubule-associated proteins 1A/1B light chain 3A | 14.272 | 6.4033 | 23.1 | 3 | 3 | 3 | 1.00 | 9.1% |
| A0A0G2K988 | Protein Daam2 | 128.1 | 5.5881 | 2.8 | 3 | 4 | 3 | 1.00 | 6.7% |
| D3ZI07 | Kinesin-like protein | 85.256 | 33.603 | 11 | 7 | 8 | 7 | 1.00 | 3.6% |
| Q5M9G3 | Caprin-1 | 78.12 | 94.603 | 17.7 | 12 | 22 | 12 | 1.00 | 1.4% |
| Q6P7A2 | Ubiquitin conjugation factor E4 A | 122.38 | 59.546 | 10.3 | 9 | 11 | 9 | 1.00 | 0.7% |
| B5DEG7 | Protein Champ1 | 87.39 | 3.3381 | 1.1 | 1 | 1 | 1 | 1.00 | 0.2% |
| Q4KLI7 | Protein Sf3a3 | 58.841 | 42.044 | 14.2 | 6 | 7 | 6 | 1.00 | 0.4% |
| Q64122 | Myosin regulatory light polypeptide 9 | 19.61 | 97.655 | 52.6 | 11 | 63 | 5 | 1.00 | 0.5% |
| P69060 | N-acylneuraminate cytidylyltransferase | 48.129 | 39.235 | 21.3 | 9 | 10 | 9 | 1.00 | 1.1% |
| A0A0G2K8R3 | Protein Lmo7 | 195.68 | 219.44 | 25.9 | 38 | 64 | 38 | 1.00 | 2.0% |
| Q6AY61 | Serine protease 23 | 43.159 | 26.357 | 13.1 | 4 | 5 | 4 | 1.00 | 4.8% |
| Q498T2 | Chromatin target of PRMT1 protein | 26.516 | 2.7916 | 6.5 | 1 | 1 | 1 | 1.00 | 7.3% |
| D4A9W3 | Protein RGD1311756 | 66.055 | 24.775 | 10 | 6 | 11 | 6 | 1.00 | 2.5% |
| D3ZF26 | Protein Tnks1bp1 | 180.01 | 289.05 | 26.9 | 31 | 48 | 31 | 1.00 | 0.2% |
| D3ZIN7 | Mitochondrial ribosomal protein S23 (Predicted), isoform CRA_b | 20.321 | 5.1641 | 11.3 | 2 | 2 | 2 | 1.00 | 0.9% |
| A0A0G2K6E8 | Protein Spag9 | 149.58 | 161.09 | 22.3 | 27 | 38 | 27 | 1.00 | 1.9% |
| E9PSY8 | Protein Eps15 | 98.793 | 109.02 | 23.2 | 18 | 22 | 18 | 1.00 | 1.9% |
| D3ZVN7 | Orexin | 50.784 | 18.893 | 14 | 5 | 5 | 5 | 1.00 | 3.3% |
| A0A0G2K7W6 | 60S ribosomal protein L27a | 16.587 | 30.573 | 31.8 | 6 | 11 | 6 | 1.00 | 3.0% |
| F1LN59 | Protein Eif4g2 | 102.11 | 173.08 | 28.6 | 23 | 33 | 23 | 1.00 | 0.6% |
| A0A0G2KAN5 | Enhancer of rudimentary homolog | 14.206 | 20.873 | 29.5 | 4 | 7 | 4 | 1.00 | 6.1% |
| Q04931 | FACT complex subunit SSRP1 | 80.914 | 37.707 | 11.4 | 7 | 9 | 7 | 1.00 | 6.7% |
| Q99PW3 | Sialidase-1 | 44.695 | 51.372 | 19.6 | 6 | 7 | 6 | 1.00 | 6.7% |
| Q498U4 | SAP domain-containing ribonucleoprotein | 23.605 | 52.425 | 29.5 | 5 | 6 | 5 | 1.00 | 3.7% |
| M0R3Z8 | Protein Rbm15 | 105.76 | 57.075 | 10.3 | 8 | 10 | 8 | 1.00 | 3.5% |
| O35092 | Mitochondrial import inner membrane translocase subunit Tim17-A | 18.037 | 22.669 | 13.5 | 1 | 1 | 1 | 1.00 | 1.5% |
| D3ZIE9 | Protein Aldh18a1 | 87.328 | 138.53 | 28.4 | 19 | 28 | 19 | 1.00 | 0.0% |
| P81155 | Voltage-dependent anion-selective channel protein 2 | 31.745 | 191.23 | 49.2 | 14 | 31 | 13 | 1.00 | 2.1% |
| D3ZXH7 | Protein Alyref | 19.915 | 45.587 | 29.1 | 5 | 7 | 5 | 1.00 | 4.3% |
| F1LQZ3 | Kinesin-like protein | 80.285 | 28.485 | 10.5 | 7 | 8 | 7 | 1.00 | 7.2% |
| Q920F5 | Malonyl-CoA decarboxylase, mitochondrial | 54.761 | 33.234 | 6.9 | 2 | 2 | 2 | 1.00 | 7.2% |
| A0A0G2JYE0 | Protein Atxn2l | 113.58 | 89.905 | 15.5 | 14 | 19 | 14 | 1.00 | 3.4% |
| D4A5F1 | Protein Pkd2 | 109.09 | 35.284 | 10 | 8 | 11 | 8 | 1.00 | 2.2% |
| D3ZPW7 | Glutathione peroxidase | 24.143 | 26.62 | 38.8 | 7 | 9 | 7 | 1.00 | 1.0% |
| G3V8G2 | Proteasome (Prosome, macropain) 26S subunit, non-ATPase, 5 (Predicted), isoform CRA_a | 55.852 | 129.61 | 31.3 | 12 | 17 | 12 | 1.00 | 1.7% |
| P22734 | Catechol O-methyltransferase | 29.597 | 16.096 | 21.2 | 5 | 11 | 5 | 1.00 | 0.9% |
| Q5M7W5 | Microtubule-associated protein 4 | 110.3 | 323.31 | 47.3 | 38 | 87 | 38 | 1.00 | 0.6% |
| Q3MIE4 | Synaptic vesicle membrane protein VAT-1 homolog | 43.118 | 211.8 | 59.4 | 17 | 55 | 17 | 1.00 | 0.4% |
| A0A0G2K1U9 | Protein Irgq | 59.103 | 37.886 | 11.2 | 4 | 4 | 4 | 1.00 | 1.0% |
| D4AD37 | Inositol monophosphatase 3 | 38.569 | 28.093 | 13.8 | 5 | 10 | 5 | 1.00 | 1.3% |
| Q62969 | Prostacyclin synthase | 57.127 | 14.484 | 2.8 | 1 | 1 | 1 | 1.00 | 4.3% |
| P08430 | UDP-glucuronosyltransferase 1-6 | 60.131 | 6.811 | 3.8 | 2 | 2 | 2 | 1.00 | 8.1% |
| E9PTF3 | Protein Noc2l | 73.767 | 26.943 | 4.2 | 2 | 3 | 2 | 1.00 | 3.4% |
| P84817 | Mitochondrial fission 1 protein | 16.994 | 48.111 | 30.9 | 6 | 9 | 6 | 1.00 | 3.3% |
| A1A5S1 | Pre-mRNA-processing factor 6 | 106.76 | 13.169 | 4.3 | 4 | 5 | 4 | 1.00 | 1.7% |
| Q63358 | Unconventional myosin-IXb | 225.03 | 33.996 | 5.4 | 11 | 12 | 11 | 1.00 | 0.1% |
| D3ZRJ0 | AarF domain containing kinase 1 (Predicted) | 51.71 | 3.1778 | 2.9 | 1 | 1 | 1 | 1.00 | 1.6% |
| Q5BK32 | FAS-associated factor 2 | 41.079 | 105.73 | 42.2 | 10 | 15 | 10 | 1.00 | 7.0% |
| Q7TP40 | PEST proteolytic signal-containing nuclear protein | 20.195 | 9.1594 | 12.7 | 2 | 2 | 2 | 1.00 | 6.5% |
| F1LQ27 | Protein Fam98c | 37.168 | 12.121 | 9.9 | 3 | 3 | 3 | 1.00 | 8.2% |
| B1WC40 | Nuclear cap-binding protein subunit 2 | 18.017 | 8.9054 | 13.5 | 2 | 3 | 2 | 1.00 | 4.0% |
| Q9EPJ0 | Nuclear ubiquitous casein and cyclin-dependent kinase substrate 1 | 27.14 | 39.707 | 15.2 | 4 | 6 | 4 | 1.00 | 1.6% |
| Q9WV25 | Poly(U)-binding-splicing factor PUF60 | 60.248 | 89.191 | 21.6 | 9 | 10 | 9 | 1.00 | 3.6% |
| Q498M8 | Nuclear RNA export factor 7 | 71.232 | 2.9757 | 2 | 1 | 2 | 1 | 1.00 | 4.4% |
| G3V7P6 | Nudix (Nucleoside diphosphate linked moiety X)-type motif 16 (Predicted), isoform CRA_a | 21.695 | 30.062 | 26.2 | 4 | 4 | 4 | 1.00 | 8.2% |
| P37199 | Nuclear pore complex protein Nup155 | 155 | 46.652 | 9.3 | 11 | 13 | 11 | 1.00 | 5.3% |
| F1M3P6 | Protein Scai | 70.288 | 5.613 | 1.7 | 1 | 1 | 1 | 1.00 | 5.1% |
| D4A650 | Protein Msrb3 | 29.033 | 55.362 | 24.4 | 6 | 9 | 6 | 1.00 | 4.9% |
| D4AC70 | Protein Col8a1 | 73.506 | 35.477 | 5.8 | 4 | 7 | 4 | 1.00 | 4.7% |
| D3ZSV7 | Protein Thumpd3 | 56.381 | 6.4682 | 4.6 | 2 | 2 | 2 | 1.00 | 2.1% |
| Q6P685 | Eukaryotic translation initiation factor 2, subunit 2 (Beta) | 38.243 | 91.611 | 41.1 | 15 | 28 | 15 | 1.00 | 0.2% |
| B1WC34 | Protein Prkcsh | 59.218 | 88.542 | 25.3 | 16 | 27 | 16 | 1.00 | 0.7% |
| Q5FWT1 | Protein FAM98A | 55.07 | 95.044 | 20.8 | 7 | 9 | 7 | 1.00 | 0.6% |
| D3ZQI0 | Polymerase (RNA) II (DNA directed) polypeptide J (Predicted) | 13.293 | 3.0058 | 6.8 | 1 | 2 | 1 | 1.00 | 0.8% |
| Q923S8 | Pantothenate kinase 4 | 86.242 | 18.329 | 4.9 | 3 | 3 | 3 | 0.99 | 7.6% |
| Q499P8 | UPF0420 protein C16orf58 homolog | 50.779 | 50.473 | 14.8 | 5 | 6 | 5 | 0.99 | 6.4% |
| Q68FW9 | COP9 signalosome complex subunit 3 | 47.859 | 115.46 | 23.4 | 8 | 13 | 8 | 0.99 | 1.8% |
| Q62696 | Disks large homolog 1 | 100.57 | 81.649 | 17.6 | 13 | 15 | 12 | 0.99 | 0.7% |
| A0A0G2JXT8 | Protein Flnb | 277.84 | 323.31 | 57.6 | 118 | 278 | 103 | 0.99 | 0.4% |
| A0A0G2JXD0 | 60S ribosomal protein L9 | 21.424 | 72.212 | 39.9 | 8 | 23 | 8 | 0.99 | 0.2% |
| Q5HZE2 | Transmembrane protein 120A | 40.657 | 23.461 | 13.1 | 4 | 4 | 4 | 0.99 | 0.3% |
| Q62848 | ADP-ribosylation factor GTPase-activating protein 1 | 45.442 | 56.227 | 22.4 | 7 | 10 | 7 | 0.99 | 0.9% |
| Q9JIL3 | Interleukin enhancer-binding factor 3 | 95.934 | 39.977 | 15.2 | 12 | 17 | 12 | 0.99 | 2.2% |
| P57093 | Phytanoyl-CoA dioxygenase, peroxisomal | 38.588 | 3.2482 | 4.7 | 2 | 2 | 2 | 0.99 | 2.8% |
| B5DFF4 | Protein LOC100910929 | 38.44 | 20.666 | 9.1 | 2 | 2 | 2 | 0.99 | 4.0% |
| Q6AY25 | Transmembrane emp24 domain-containing protein 3 | 25.51 | 49.907 | 18.1 | 4 | 8 | 4 | 0.99 | 4.3% |
| O35760 | Isopentenyl-diphosphate Delta-isomerase 1 | 26.396 | 39.784 | 22.5 | 6 | 7 | 6 | 0.99 | 7.0% |
| D3ZAQ2 | Mothers against decapentaplegic homolog | 49.234 | 2.3313 | 3.5 | 2 | 4 | 2 | 0.99 | 4.2% |
| B2RYW9 | Fumarylacetoacetate hydrolase domain-containing protein 2 | 34.581 | 97.451 | 37.1 | 8 | 11 | 8 | 0.99 | 3.4% |
| D3ZH40 | Protein Otud7b | 91.954 | 4.3824 | 1.8 | 1 | 1 | 1 | 0.99 | 2.9% |
| Q08849 | Syntaxin-3 | 33.257 | 8.0815 | 7.3 | 2 | 2 | 2 | 0.99 | 1.8% |
| Q5XI44 | Protein Xrcc4 | 36.477 | 13.337 | 9.9 | 3 | 4 | 3 | 0.99 | 1.5% |
| P62161 | Calmodulin | 16.837 | 99.671 | 52.3 | 11 | 60 | 11 | 0.99 | 0.5% |
| P24062 | Insulin-like growth factor 1 receptor | 155.39 | 19.234 | 6.5 | 9 | 11 | 9 | 0.99 | 0.9% |
| Q7TQ77 | Ac1288 | 80.493 | 67.3 | 15.1 | 11 | 18 | 11 | 0.99 | 1.2% |
| Q7M733 | Hermansky-Pudlak syndrome 6 protein homolog | 87.461 | 4.6354 | 2 | 1 | 2 | 1 | 0.99 | 10.1% |
| B5DEZ6 | Glucosamine-6-phosphate isomerase | 31.006 | 19.632 | 21.4 | 5 | 7 | 3 | 0.99 | 8.3% |
| D4A629 | Protein Pcmtd1 | 40.653 | 3.1335 | 2.8 | 1 | 1 | 1 | 0.99 | 7.5% |
| F1MAQ8 | Protein Pnisr | 93.438 | 2.5397 | 0.9 | 1 | 1 | 1 | 0.99 | 2.1% |
| P38650 | Cytoplasmic dynein 1 heavy chain 1 | 532.25 | 323.31 | 44.3 | 197 | 368 | 197 | 0.99 | 0.5% |
| M0RBX8 | Oligosaccharyltransferase complex subunit OSTC | 9.377 | 12.038 | 14.6 | 1 | 6 | 1 | 0.99 | 1.5% |
| D3ZHA1 | Protein B3gnt1 | 47.47 | 10.006 | 3.9 | 1 | 1 | 1 | 0.99 | 3.3% |
| P62744 | AP-2 complex subunit sigma | 17.018 | 32.854 | 35.9 | 5 | 8 | 5 | 0.99 | 4.1% |
| Q66H47 | 39S ribosomal protein L24, mitochondrial | 25 | 8.4763 | 11.1 | 1 | 1 | 1 | 0.99 | 5.7% |
| O35831 | Cyclin-dependent kinase 17 | 59.431 | 14.193 | 7.5 | 4 | 5 | 2 | 0.99 | 14.3% |
| A0A0G2K889 | Protein Ehmt1 | 141.46 | 8.4073 | 1.1 | 1 | 1 | 1 | 0.99 | 3.8% |
| D3ZQC1 | Protein B3galt6 | 37.148 | 13.499 | 12.6 | 3 | 3 | 3 | 0.99 | 1.8% |
| D3ZHG3 | Protein Ptk7 | 86.263 | 323.01 | 38 | 22 | 37 | 22 | 0.99 | 1.6% |
| D4AC73 | Protein Chchd7 | 10.101 | 15.889 | 21.2 | 1 | 1 | 1 | 0.99 | 10.2% |
| D4A2U2 | Protein Zbtb32 | 52.542 | -2 | 1.4 | 1 | 1 | 1 | 0.99 | 10.3% |
| D4A8G0 | Protein Lsm12 | 21.701 | 12.109 | 18.5 | 3 | 4 | 3 | 0.99 | 3.7% |
| A0A0G2JXP3 | Protein Nedd4l | 110.63 | 8.0396 | 4.5 | 5 | 8 | 2 | 0.99 | 2.5% |
| D4A9D8 | Oxysterol-binding protein | 77.806 | 102.48 | 28.3 | 18 | 24 | 18 | 0.99 | 1.1% |
| B2RYK2 | Diphthamide biosynthesis protein 1 | 48.104 | 14.116 | 3.9 | 1 | 1 | 1 | 0.99 | 0.4% |
| O54857 | Phosphatase and tensin homolog, isoform CRA_a | 47.118 | 8.3491 | 7.2 | 2 | 2 | 2 | 0.99 | 1.1% |
| Q8K4Y7 | Soluble calcium-activated nucleotidase 1 | 45.658 | 23.99 | 14.1 | 5 | 6 | 5 | 0.99 | 5.6% |
| Q6P6T9 | Importin subunit alpha | 57.836 | 30.551 | 5.7 | 2 | 4 | 2 | 0.99 | 15.8% |
| M0R5D2 | Protein RGD1564148 | 51.859 | 9.3557 | 7.8 | 3 | 3 | 3 | 0.99 | 4.2% |
| Q9JHX4 | Caspase-8 | 55.338 | 72.791 | 17 | 5 | 6 | 5 | 0.99 | 11.2% |
| M0R7E6 | Protein Srrt | 54.984 | 45.453 | 16.6 | 8 | 10 | 8 | 0.99 | 4.3% |
| B2RZ74 | Protein Snrnp70 | 52.133 | 27.446 | 18 | 9 | 10 | 9 | 0.99 | 3.6% |
| A0A096MKH2 | Protein Vta1 | 33.975 | 19.157 | 15.9 | 4 | 5 | 4 | 0.99 | 1.9% |
| D4A2Z8 | DEAH (Asp-Glu-Ala-His) box polypeptide 36 (Predicted), isoform CRA_a | 113.84 | 21.48 | 6.7 | 6 | 6 | 6 | 0.99 | 0.1% |
| E9PU78 | Bcl-2-like protein 1 | 19.059 | 7.2924 | 11.2 | 2 | 3 | 2 | 0.99 | 0.3% |
| Q5XI13 | Glutamate-rich WD repeat-containing protein 1 | 49.168 | 25.562 | 4.7 | 2 | 2 | 2 | 0.99 | 3.9% |
| D4A4J0 | Protein Supt16h | 119.86 | 20.752 | 5.8 | 5 | 5 | 5 | 0.99 | 5.0% |
| A0A0G2K264 | Disks large-associated protein 3 | 46.665 | 24.783 | 7.5 | 2 | 2 | 2 | 0.99 | 16.7% |
| P12075 | Cytochrome c oxidase subunit 5B, mitochondrial | 13.915 | 21.141 | 36.4 | 6 | 18 | 6 | 0.99 | 1.1% |
| Q09167 | Serine/arginine-rich splicing factor 5 | 30.891 | 17.185 | 17.5 | 5 | 7 | 5 | 0.99 | 2.3% |
| A1A5Q1 | Parp9 protein | 92.214 | 3.463 | 2.4 | 2 | 2 | 2 | 0.99 | 2.7% |
| D4A0J7 | Olfactomedin-like 2B (Predicted), isoform CRA_a | 83.447 | 39.739 | 3.9 | 2 | 2 | 2 | 0.99 | 6.5% |
| D4A3V4 | Protein Rnf214 | 77.723 | 21.002 | 4.3 | 2 | 2 | 2 | 0.99 | 16.1% |
| D3ZCW5 | Asparagine-linked glycosylation 9 homolog (Yeast, alpha 1,2 mannosyltransferase) (Predicted) | 47.731 | 3.5105 | 3 | 1 | 1 | 1 | 0.99 | 5.9% |
| O35353 | Guanine nucleotide-binding protein subunit beta-4 | 37.363 | 28.797 | 24.4 | 7 | 17 | 4 | 0.99 | 4.3% |
| Q6P9U3 | COMM domain-containing protein 3 | 21.932 | 157.91 | 47.2 | 7 | 10 | 7 | 0.99 | 0.8% |
| Q9JJ22 | Endoplasmic reticulum aminopeptidase 1 | 106.42 | 128.35 | 21.2 | 17 | 26 | 17 | 0.99 | 1.3% |
| D3ZFY0 | Protein Sephs1 | 42.892 | 74.531 | 13.8 | 3 | 3 | 3 | 0.99 | 2.5% |
| F1LPJ9 | Uncharacterized protein | 43.351 | 10.838 | 6.3 | 3 | 3 | 2 | 0.99 | 4.9% |
| Q5U3Y2 | SWI/SNF-related matrix-associated actin-dependent regulator of chromatin subfamily D member 2 | 53.615 | 29.56 | 8.5 | 2 | 2 | 2 | 0.99 | 9.2% |
| P63014 | Paired mesoderm homeobox protein 1 | 27.269 | 13.913 | 10.6 | 2 | 2 | 2 | 0.99 | 1.2% |
| D3ZPJ9 | Protein Ascc2 | 85.695 | 30.276 | 8.5 | 5 | 5 | 5 | 0.99 | 1.6% |
| B2GV57 | Protein Cars2 | 60.917 | 12.977 | 2.2 | 1 | 1 | 1 | 0.99 | 10.6% |
| P80385 | 5-AMP-activated protein kinase subunit gamma-1 | 37.386 | 38.895 | 24.2 | 7 | 9 | 7 | 0.99 | 2.1% |
| Q5I0E7 | Transmembrane emp24 domain-containing protein 9 | 27.028 | 79.839 | 30.6 | 7 | 21 | 6 | 0.99 | 0.2% |
| F1LN46 | Carnitine O-palmitoyltransferase 1, brain isoform | 90.169 | 7.6148 | 3 | 3 | 4 | 3 | 0.99 | 1.1% |
| D3ZX87 | 60S ribosomal protein L31 | 16.321 | 80.989 | 41.3 | 7 | 13 | 7 | 0.99 | 4.7% |
| G3V6G7 | Polyribonucleotide nucleotidyltransferase 1 | 85.897 | 18.2 | 6.6 | 5 | 5 | 5 | 0.99 | 3.2% |
| Q4V898 | RNA-binding motif protein, X chromosome | 42.257 | 59.475 | 31 | 14 | 25 | 4 | 0.99 | 3.6% |
| Q7TSE9 | HCLS1-associated protein X-1 | 31.448 | 10.395 | 10.4 | 3 | 3 | 3 | 0.99 | 10.4% |
| G3V6H2 | Pre-mRNA processing factor 8, isoform CRA_a | 273.61 | 111.8 | 11.4 | 24 | 28 | 24 | 0.99 | 0.9% |
| A0A0G2K6R8 | Protein Ehbp1l1 | 182.51 | 238.37 | 21.4 | 28 | 38 | 28 | 0.99 | 0.3% |
| O88941 | Mannosyl-oligosaccharide glucosidase | 91.87 | 69.489 | 16.8 | 11 | 19 | 11 | 0.99 | 0.4% |
| P11442 | Clathrin heavy chain 1 | 191.6 | 323.31 | 54.9 | 86 | 217 | 86 | 0.99 | 0.9% |
| P05508 | NADH-ubiquinone oxidoreductase chain 4 | 51.782 | 4.521 | 3.7 | 2 | 4 | 2 | 0.99 | 10.0% |
| D3ZD05 | Protein Kank2 | 90.883 | 304.45 | 43.8 | 27 | 44 | 26 | 0.99 | 0.9% |
| A0A0G2K9P5 | Protein RGD1563296 | 83.618 | 62.791 | 14.2 | 9 | 10 | 9 | 0.99 | 0.1% |
| B1H2A6 | Fxr2 protein | 74.374 | 123.86 | 28 | 17 | 24 | 13 | 0.99 | 0.4% |
| Q5PPJ6 | Leucyl-tRNA synthetase | 134.28 | 235.14 | 32.7 | 33 | 57 | 33 | 0.99 | 0.6% |
| Q331S7 | Protein Dbndd2 | 17.186 | 4.6459 | 7.6 | 1 | 1 | 1 | 0.99 | 0.8% |
| Q32Q88 | F-box protein 22 | 44.008 | 9.7241 | 5.5 | 2 | 2 | 2 | 0.99 | 1.4% |
| Q6AY94 | Complex I assembly factor TIMMDC1, mitochondrial | 32.05 | 16.894 | 6.3 | 1 | 1 | 1 | 0.99 | 2.2% |
| Q8R2E7 | FADD/MORT1 protein with death effector domain | 23.124 | 25.182 | 13 | 2 | 2 | 2 | 0.99 | 2.8% |
| O35186 | Cathepsin K | 36.79 | 4.4548 | 7.3 | 3 | 3 | 3 | 0.99 | 3.6% |
| Q499T7 | Cilia- and flagella-associated protein 20 | 19.132 | 2.4328 | 5.5 | 1 | 2 | 1 | 0.99 | 4.6% |
| P55266 | Double-stranded RNA-specific adenosine deaminase | 129.91 | 2.7458 | 1.3 | 1 | 1 | 1 | 0.99 | 4.9% |
| Q64244 | ADP-ribosyl cyclase/cyclic ADP-ribose hydrolase 1 | 34.436 | 14.59 | 11.6 | 3 | 5 | 3 | 0.99 | 10.9% |
| F1MA59 | Protein Col4a1 | 160.61 | 51.385 | 4.1 | 5 | 6 | 5 | 0.99 | 5.7% |
| Q5PPJ9 | Endophilin-B2 | 44.852 | 2.4239 | 6.4 | 3 | 4 | 2 | 0.99 | 5.5% |
| P35284 | Ras-related protein Rab-12 | 27.271 | 15.047 | 18.5 | 5 | 6 | 5 | 0.99 | 1.9% |
| Q4G079 | Protein Aimp1 | 34.574 | 166.52 | 52.7 | 13 | 28 | 13 | 0.99 | 1.0% |
| D4AE02 | Protein Fam98b | 46.414 | 17.171 | 9.7 | 3 | 3 | 3 | 0.99 | 0.1% |
| Q9QYW3 | MOB-like protein phocein | 26.032 | 28.791 | 32.9 | 5 | 5 | 5 | 0.99 | 0.8% |
| A0A0G2K9J2 | Protein Atp6v1h | 55.868 | 52.275 | 18.4 | 6 | 7 | 6 | 0.99 | 2.5% |
| D3ZHK4 | Protein Rb1cc1 | 182.23 | 113.29 | 9.8 | 11 | 11 | 11 | 0.99 | 1.4% |
| A0JN30 | Canopy 2 homolog (Zebrafish) | 20.709 | 75.271 | 56.6 | 10 | 25 | 10 | 0.99 | 2.2% |
| D3ZC89 | Protein Fam114a2 | 54.454 | 76.829 | 13.6 | 5 | 6 | 5 | 0.99 | 6.2% |
| D4AB23 | Protein Noc3l | 92.424 | 12.766 | 3.4 | 2 | 2 | 2 | 0.99 | 4.3% |
| A0A0G2K502 | Protein Me2 | 66.309 | 153.06 | 29.4 | 13 | 17 | 13 | 0.99 | 3.7% |
| Q9Z2X5 | Homer protein homolog 3 | 39.891 | 51.618 | 24.9 | 7 | 9 | 7 | 0.99 | 1.0% |
| P13852 | Major prion protein | 27.804 | 11.06 | 8.7 | 3 | 5 | 3 | 0.99 | 0.2% |
| Q6AYH5 | Dynactin subunit 2 | 44.147 | 238.31 | 62.2 | 22 | 48 | 22 | 0.99 | 0.1% |
| Q920L2 | Succinate dehydrogenase [ubiquinone] flavoprotein subunit, mitochondrial | 71.614 | 182.48 | 32.8 | 17 | 28 | 17 | 0.99 | 0.1% |
| A0A0G2JZI2 | Protein Eprs | 169.86 | 323.31 | 42.3 | 54 | 105 | 54 | 0.99 | 2.1% |
| B5DEH0 | LIM domain-containing protein 1 | 71.392 | 67.8 | 12.7 | 5 | 6 | 5 | 0.99 | 6.1% |
| F1LPV8 | Succinyl-CoA ligase subunit beta | 46.638 | 121.6 | 42 | 15 | 18 | 15 | 0.99 | 1.4% |
| Q499T5 | Calpain 7 | 92.515 | 24.076 | 5.2 | 3 | 3 | 3 | 0.99 | 3.0% |
| Q6AYY8 | Acetyl-coenzyme A transporter 1 | 61.101 | 22.405 | 6 | 3 | 5 | 3 | 0.99 | 8.2% |
| Q9JJP9 | Ubiquilin-1 | 62.071 | 15.872 | 6 | 3 | 6 | 2 | 0.99 | 8.9% |
| M0R715 | Protein Pnpla6 | 149.44 | 34.345 | 4.8 | 5 | 6 | 4 | 0.99 | 20.3% |
| F1M0Z4 | Protein Arhgef10 | 143.38 | 8.3156 | 1.9 | 2 | 2 | 2 | 0.99 | 3.6% |
| Q5XIU9 | Membrane-associated progesterone receptor component 2 | 23.403 | 57.134 | 45.6 | 9 | 12 | 8 | 0.99 | 2.7% |
| Q9JJ54 | Heterogeneous nuclear ribonucleoprotein D0 | 38.218 | 80.717 | 31.7 | 11 | 23 | 10 | 0.99 | 2.2% |
| P51799 | H(+)/Cl(-) exchange transporter 7 | 88.73 | 30.293 | 7.7 | 6 | 6 | 6 | 0.99 | 1.9% |
| Q6AYH6 | ER membrane protein complex subunit 10 | 26.968 | 69.441 | 22.5 | 3 | 6 | 3 | 0.99 | 1.2% |
| Q68FP9 | Conserved oligomeric Golgi complex subunit 6 | 72.956 | 26.637 | 9.4 | 6 | 6 | 6 | 0.99 | 0.2% |
| D4ABK7 | Heterogeneous nuclear ribonucleoprotein H3 (2H9) (Predicted), isoform CRA_c | 36.869 | 46.285 | 19.7 | 6 | 8 | 5 | 0.99 | 2.0% |
| P54311 | Guanine nucleotide-binding protein G(I)/G(S)/G(T) subunit beta-1 | 37.377 | 253.86 | 46.8 | 12 | 40 | 6 | 0.99 | 2.0% |
| D3ZZP2 | Protein Rab39a | 24.92 | 24.339 | 26.3 | 6 | 12 | 5 | 0.99 | 2.6% |
| D4ADR5 | Protein Chpf2 | 85.921 | 43.429 | 14 | 10 | 11 | 10 | 0.99 | 3.1% |
| A0A0G2K5L2 | Protein LOC100911440 | 34.662 | 13.048 | 13 | 4 | 5 | 4 | 0.99 | 5.5% |
| B2RYI2 | Signal recognition particle subunit SRP68 | 70.491 | 170.8 | 41.1 | 24 | 32 | 24 | 0.99 | 4.8% |
| Q52KJ9 | Protein Tmx1 | 31.434 | 27.741 | 17.6 | 7 | 11 | 7 | 0.99 | 2.9% |
| P14882 | Propionyl-CoA carboxylase alpha chain, mitochondrial | 81.622 | 45.237 | 11.5 | 7 | 10 | 7 | 0.99 | 1.5% |
| P36202 | PDZ and LIM domain protein 4 | 35.521 | 125.34 | 52.4 | 14 | 20 | 14 | 0.99 | 0.6% |
| M0R535 | Cation-dependent mannose-6-phosphate receptor | 31.077 | 34.438 | 21.6 | 5 | 10 | 5 | 0.99 | 0.0% |
| Q5BJT6 | Large subunit GTPase 1 homolog | 74.402 | 8.8327 | 4.9 | 3 | 3 | 3 | 0.99 | 0.0% |
| B5DFM8 | Breast carcinoma amplified sequence 2 | 26.101 | 25.234 | 15.6 | 3 | 4 | 3 | 0.99 | 0.2% |
| A0A0G2K5D7 | Protein Specc1 | 127.6 | 141.28 | 22.2 | 24 | 27 | 24 | 0.99 | 1.3% |
| D4A1F2 | Protein-methionine sulfoxide oxidase MICAL2 | 110.09 | 62.072 | 12.9 | 11 | 16 | 11 | 0.99 | 1.5% |
| Q6P691 | Protein C2cd2 | 76.538 | 62.79 | 13.5 | 11 | 14 | 11 | 0.99 | 2.0% |
| Q562A2 | Zinc finger RNA-binding protein | 116.82 | 58.397 | 6.7 | 6 | 6 | 6 | 0.99 | 2.1% |
| Q5U2Z4 | Nuclear factor of kappa light polypeptide gene enhancer in B-cells 2, p49/p100 | 96.738 | 52.523 | 8.2 | 6 | 6 | 6 | 0.99 | 2.7% |
| Q6MG55 | Abhydrolase domain-containing protein 16A | 63.037 | 67.036 | 17.4 | 8 | 8 | 8 | 0.99 | 4.7% |
| D3ZQM3 | Integrin alpha 3 variant B | 119.12 | 10.919 | 4.3 | 4 | 4 | 4 | 0.99 | 8.4% |
| D3ZAY8 | Protein Pnn | 82.609 | 34.203 | 6.7 | 4 | 4 | 4 | 0.99 | 6.4% |
| P63164 | Small nuclear ribonucleoprotein-associated protein N | 24.614 | 36.251 | 27.1 | 6 | 13 | 6 | 0.99 | 3.3% |
| Q66H61 | Glutaminyl-tRNA synthetase | 87.699 | 198.01 | 33.4 | 23 | 29 | 23 | 0.99 | 3.3% |
| G3V9N7 | Protein Pacsin3 | 48.598 | 27.906 | 13.4 | 5 | 5 | 5 | 0.99 | 1.5% |
| B2RYP6 | LUC7-like 2 (S. cerevisiae) | 46.582 | 45.201 | 21.4 | 7 | 10 | 5 | 0.99 | 0.7% |
| P06685 | Sodium/potassium-transporting ATPase subunit alpha-1 | 113.05 | 323.31 | 44.1 | 40 | 98 | 40 | 0.99 | 0.4% |
| Q32PX7 | Far upstream element-binding protein 1 | 67.196 | 79.009 | 32.7 | 21 | 26 | 18 | 0.99 | 0.2% |
| A0A0G2K3C6 | Glutathione S-transferase theta-2 | 20.318 | 17.471 | 19.9 | 3 | 3 | 3 | 0.99 | 0.1% |
| Q99376 | Transferrin receptor protein 1 | 70.152 | 26.165 | 13.5 | 9 | 12 | 9 | 0.99 | 0.1% |
| Q5XIF6 | Tubulin alpha-4A chain | 49.924 | 55.66 | 53.6 | 21 | 130 | 4 | 0.99 | 0.4% |
| Q63713 | Ras GTPase-activating protein 2 | 96.373 | 9.3295 | 3.8 | 3 | 3 | 3 | 0.99 | 1.5% |
| B0BNK1 | Protein Rab5c | 23.425 | 111.52 | 68.5 | 11 | 41 | 8 | 0.99 | 2.1% |
| F1LY19 | Protein Upf1 | 122.64 | 249.8 | 31.4 | 29 | 35 | 29 | 0.99 | 2.5% |
| B5DEK0 | Protein Rprd1b | 36.883 | 47.6 | 16.9 | 4 | 4 | 4 | 0.99 | 5.1% |
| G3V6S8 | Serine/arginine-rich splicing factor 6 | 39.025 | 26.478 | 26 | 10 | 13 | 10 | 0.99 | 2.8% |
| B2GV55 | Protein Ube2q1 | 46.172 | 19.906 | 14 | 3 | 3 | 3 | 0.99 | 2.2% |
| Q09325 | Alpha-1,3-mannosyl-glycoprotein 2-beta-N-acetylglucosaminyltransferase | 51.585 | 37.424 | 18.6 | 7 | 11 | 7 | 0.99 | 1.8% |
| D3ZYX8 | Cytochrome c oxidase subunit VIIa polypeptide 2 like (Predicted), isoform CRA_e | 13.273 | 13.235 | 18.3 | 3 | 4 | 3 | 0.99 | 4.2% |
| Q68FQ2 | Junctional adhesion molecule C | 34.782 | 2.152 | 5.5 | 2 | 2 | 2 | 0.99 | 4.8% |
| A0A0G2K0I1 | Protein Spata5 | 91.4 | 48.961 | 7 | 4 | 5 | 4 | 0.99 | 0.1% |
| Q792I0 | Protein lin-7 homolog C | 21.834 | 19.486 | 16.2 | 3 | 4 | 3 | 0.99 | 0.0% |
| G3V9K0 | Cysteinyl-tRNA synthetase (Predicted), isoform CRA_b | 85.593 | 109.02 | 23.3 | 15 | 20 | 15 | 0.99 | 0.8% |
| D4A4Q3 | Protein yippee-like | 13.841 | 35.136 | 41.3 | 4 | 6 | 4 | 0.99 | 1.1% |
| P38656 | Lupus La protein homolog | 47.777 | 95.125 | 36.9 | 16 | 23 | 16 | 0.99 | 1.5% |
| P61107 | Ras-related protein Rab-14 | 23.927 | 103.01 | 41.9 | 9 | 20 | 9 | 0.99 | 2.9% |
| D4A7N1 | MICOS complex subunit Mic25 | 29.211 | 36.239 | 30.3 | 7 | 11 | 7 | 0.99 | 3.9% |
| Q63921 | Prostaglandin G/H synthase 1 | 69.032 | 12.056 | 7.5 | 4 | 4 | 4 | 0.99 | 2.4% |
| Q8VHV7 | Heterogeneous nuclear ribonucleoprotein H | 49.188 | 78.035 | 27.2 | 9 | 28 | 3 | 0.99 | 2.4% |
| O35264 | Platelet-activating factor acetylhydrolase IB subunit beta | 25.581 | 18.538 | 21.8 | 3 | 3 | 3 | 0.99 | 5.4% |
| D3ZGP7 | Protein Lyst | 427.18 | 17.151 | 0.8 | 3 | 3 | 3 | 0.99 | 11.3% |
| O54922 | Exocyst complex component 7 | 75.045 | 42.731 | 14.2 | 8 | 10 | 8 | 0.99 | 4.5% |
| D3ZWZ6 | Protein Igf2bp2 | 65.404 | 34.176 | 13.3 | 6 | 9 | 5 | 0.99 | 3.4% |
| D3ZM69 | Protein Epb41l2 | 110.71 | 323.31 | 42.5 | 35 | 63 | 34 | 0.99 | 2.9% |
| P62815 | V-type proton ATPase subunit B, brain isoform | 56.55 | 121.21 | 31.3 | 13 | 26 | 13 | 0.99 | 1.8% |
| D3ZUC2 | Protein Mov10 | 113.76 | 9.0687 | 4.2 | 5 | 5 | 5 | 0.99 | 0.9% |
| O55043 | Rho guanine nucleotide exchange factor 7 | 73.139 | 41.337 | 13.2 | 8 | 9 | 6 | 0.99 | 0.9% |
| Q5VLR5 | BWK4 | 46.878 | 147.36 | 46.8 | 15 | 33 | 15 | 0.99 | 0.8% |
| M0RA26 | 40S ribosomal protein S27 | 9.5161 | 15.719 | 38.1 | 3 | 5 | 3 | 0.99 | 1.6% |
| F1M1R4 | Protein Rbm27 | 118.68 | 14.478 | 3.7 | 3 | 3 | 3 | 0.99 | 3.1% |
| Q4KM24 | Peroxisomal biogenesis factor 11 beta | 28.642 | 3.319 | 6.6 | 2 | 2 | 2 | 0.99 | 26.2% |
| Q5XIF0 | Protein Tex264 | 33.771 | 10.292 | 10.7 | 3 | 3 | 3 | 0.99 | 7.5% |
| F1LYZ8 | Protein Ppp1r21 | 88.36 | 114.02 | 14.9 | 8 | 10 | 8 | 0.99 | 5.7% |
| D3ZTR4 | Protein Sumf2 | 34.651 | 30.53 | 16 | 4 | 6 | 4 | 0.99 | 5.4% |
| Q9Z1E1 | Flotillin-1 | 47.499 | 127.48 | 47.7 | 16 | 24 | 16 | 0.99 | 3.9% |
| P16409 | Myosin light chain 3 | 22.156 | 25.627 | 12 | 3 | 9 | 2 | 0.99 | 2.5% |
| F1M558 | Protein Ddhd2 | 81.545 | 10.986 | 5.3 | 4 | 4 | 4 | 0.99 | 1.5% |
| Q68FW4 | Syntaxin-18 | 38.512 | 21.611 | 18 | 7 | 7 | 7 | 0.99 | 0.8% |
| P12007 | Isovaleryl-CoA dehydrogenase, mitochondrial | 46.435 | 65.732 | 32.1 | 12 | 22 | 12 | 0.99 | 0.6% |
| Q9ES21 | Phosphatidylinositide phosphatase SAC1 | 67.038 | 112.8 | 37.3 | 22 | 36 | 22 | 0.99 | 0.5% |
| Q642A9 | Protein Vps16 | 94.906 | 47.149 | 10.6 | 7 | 7 | 7 | 0.99 | 2.5% |
| A0A0G2K8F6 | Protein Man2b2 | 114.87 | 16.565 | 4.6 | 4 | 4 | 4 | 0.99 | 3.0% |
| Q9WUJ3 | Myomegalin | 262.04 | 16.769 | 1.8 | 3 | 3 | 3 | 0.99 | 9.7% |
| Q3KRE3 | Guanine nucleotide-binding protein subunit gamma | 7.2293 | 26.783 | 52.9 | 3 | 5 | 3 | 0.99 | 5.8% |
| F7ENH8 | Histone deacetylase | 55.345 | 32.036 | 7.8 | 3 | 3 | 3 | 0.99 | 2.1% |
| F7F469 | Protein Igtp | 157.13 | 8.8017 | 3.5 | 3 | 3 | 3 | 0.99 | 1.1% |
| O35550 | Rab GTPase-binding effector protein 1 | 99.427 | 109.55 | 16.4 | 12 | 14 | 12 | 0.99 | 1.1% |
| A0A0G2JUA5 | Protein Ahnak | 581.12 | 323.31 | 71.8 | 213 | 548 | 213 | 0.99 | 0.6% |
| A0A0G2JTB2 | Protein Gbe1 | 75.706 | 22.636 | 6.1 | 3 | 4 | 3 | 0.99 | 0.4% |
| Q9ESN0 | Protein Niban | 103.46 | 188.21 | 32.6 | 27 | 53 | 27 | 0.99 | 0.6% |
| I6L9G6 | Protein Tardbp | 32.147 | 39.263 | 22.8 | 7 | 13 | 7 | 0.99 | 1.0% |
| Q6AXV4 | Sorting and assembly machinery component 50 homolog | 51.96 | 103.49 | 35.2 | 11 | 18 | 11 | 0.99 | 1.2% |
| B0BNA7 | Eukaryotic translation initiation factor 3 subunit I | 36.46 | 38.772 | 19.7 | 6 | 9 | 6 | 0.99 | 1.5% |
| D4ACJ1 | 40S ribosomal protein S24 | 15.395 | 61.034 | 40.6 | 7 | 23 | 7 | 0.99 | 4.1% |
| P40190 | Interleukin-6 receptor subunit beta | 102.45 | 22.669 | 5.9 | 4 | 4 | 4 | 0.99 | 8.3% |
| Q64259 | Von Hippel-Lindau disease tumor suppressor | 21.215 | 4.6949 | 9.2 | 2 | 2 | 2 | 0.99 | 2.0% |
| D4A7J8 | PRP4 pre-mRNA processing factor 4 homolog (Yeast) | 58.342 | 15.923 | 7.3 | 3 | 4 | 3 | 0.99 | 6.2% |
| D4A8M5 | Protein Snrpep2 | 10.833 | 9.0501 | 25 | 2 | 3 | 2 | 0.99 | 5.8% |
| P36201 | Cysteine-rich protein 2 | 22.696 | 28.874 | 11.1 | 2 | 4 | 2 | 0.99 | 4.0% |
| D4AA63 | Protein Ubqln2 | 67.259 | 111.45 | 17.6 | 7 | 13 | 6 | 0.99 | 2.3% |
| G3V6B0 | Protein Pdxdc1 | 86.97 | 84.23 | 19.4 | 13 | 18 | 13 | 0.99 | 1.2% |
| Q07014 | Tyrosine-protein kinase Lyn | 58.659 | 17.942 | 6.4 | 3 | 5 | 2 | 0.99 | 0.8% |
| A0A0G2K6S0 | Protein Cln5 | 39.002 | 12.019 | 11.8 | 4 | 6 | 4 | 0.99 | 0.7% |
| Q641X8 | Eukaryotic translation initiation factor 3 subunit E | 52.22 | 82.566 | 41.8 | 16 | 21 | 16 | 0.99 | 0.8% |
| Q3MIE9 | Protein Sms | 41.31 | 13.703 | 7.9 | 2 | 2 | 2 | 0.99 | 1.4% |
| D3ZIK0 | Protein Glce | 70.118 | 4.5617 | 2.3 | 1 | 1 | 1 | 0.99 | 4.0% |
| Q3T1G7 | Conserved oligomeric Golgi complex subunit 7 | 86.211 | 31.19 | 9.9 | 7 | 7 | 7 | 0.99 | 1.6% |
| P42893 | Endothelin-converting enzyme 1 | 86.125 | 11.785 | 7.9 | 6 | 7 | 6 | 0.98 | 1.4% |
| Q5U4E4 | Protein Trmt1 | 47.643 | 32.198 | 17.7 | 5 | 5 | 5 | 0.98 | 1.1% |
| A0A0G2K9L2 | Protein Tom1l2 | 57.946 | 110.62 | 30.7 | 11 | 13 | 11 | 0.98 | 0.6% |
| D3ZKN0 | Protein RGD1565536 | 58.311 | 13.533 | 11.9 | 5 | 5 | 5 | 0.98 | 0.6% |
| O70351 | 3-hydroxyacyl-CoA dehydrogenase type-2 | 27.245 | 166.9 | 52.9 | 10 | 18 | 10 | 0.98 | 0.3% |
| P13086 | Succinyl-CoA ligase [ADP/GDP-forming] subunit alpha, mitochondrial | 36.148 | 63.657 | 21.1 | 6 | 11 | 6 | 0.98 | 3.2% |
| Q5M9I6 | Multiple myeloma tumor-associated protein 2 homolog | 29.299 | 3.6206 | 5.4 | 1 | 1 | 1 | 0.98 | 15.0% |
| B5DEY5 | Protein Thoc3 | 38.795 | 2.3012 | 2.3 | 1 | 2 | 1 | 0.98 | 7.5% |
| P29266 | 3-hydroxyisobutyrate dehydrogenase, mitochondrial | 35.302 | 102.99 | 42.1 | 11 | 19 | 11 | 0.98 | 5.5% |
| D4AA14 | Protein Aifm2 | 40.69 | 32.752 | 19 | 6 | 6 | 6 | 0.98 | 4.7% |
| D4A7F0 | Protein Ubr3 | 212.92 | 4.2703 | 0.9 | 2 | 2 | 2 | 0.98 | 4.1% |
| A0A0G2K777 | Protein Rhot1 | 86.063 | 83.022 | 14.1 | 9 | 12 | 9 | 0.98 | 3.8% |
| D4ABD7 | Protein Trip11 | 225.97 | 83.649 | 14.3 | 25 | 25 | 25 | 0.98 | 3.6% |
| P20070 | NADH-cytochrome b5 reductase 3 | 34.174 | 81.77 | 34.6 | 9 | 26 | 9 | 0.98 | 2.6% |
| D3ZJF9 | Protein Gla | 47.573 | 72.168 | 26.2 | 9 | 20 | 9 | 0.98 | 1.9% |
| A0A0G2K3H2 | Protein Dock7 | 241.29 | 209.83 | 16.2 | 27 | 40 | 26 | 0.98 | 0.9% |
| D3ZMS0 | Protein Grcc10 | 13.194 | 42.494 | 36.5 | 3 | 6 | 3 | 0.98 | 0.4% |
| O35889 | Afadin | 207.68 | 79.635 | 7.4 | 11 | 11 | 11 | 0.98 | 1.8% |
| D3ZZZ9 | Catenin (Cadherin associated protein), delta 1 (Predicted), isoform CRA_a | 104.07 | 160.38 | 25.8 | 22 | 33 | 22 | 0.98 | 2.9% |
| Q5PQJ7 | Tubulin-specific chaperone cofactor E-like protein | 48.045 | 16.085 | 6.6 | 2 | 3 | 2 | 0.98 | 4.5% |
| B4F795 | Choline transporter-like protein 2 | 79.896 | 24.232 | 8.1 | 4 | 6 | 4 | 0.98 | 4.7% |
| Q5EAP4 | Guanine nucleotide binding protein, alpha 14 | 41.441 | 23.423 | 7.3 | 2 | 3 | 1 | 0.98 | 5.5% |
| M0R655 | Protein Fmnl2 | 118.66 | 49.939 | 8.5 | 8 | 12 | 2 | 0.98 | 12.4% |
| A0A0G2JZB7 | Protein Nav3 | 241.53 | 6.7752 | 2.2 | 4 | 4 | 4 | 0.98 | 2.9% |
| D4A022 | Protein Gapvd1 | 165.16 | 9.9223 | 4.1 | 5 | 5 | 5 | 0.98 | 1.5% |
| F1LUD3 | Protein Ahnak2 | 584.8 | 176.55 | 17.1 | 24 | 28 | 24 | 0.98 | 1.0% |
| Q64591 | 2,4-dienoyl-CoA reductase, mitochondrial | 36.132 | 62.636 | 22.7 | 7 | 8 | 7 | 0.98 | 0.1% |
| D3ZBM3 | Ferrochelatase | 47.551 | 11.37 | 5.7 | 2 | 3 | 2 | 0.98 | 0.9% |
| Q6PCT3 | Tumor protein D54 | 23.992 | 68.991 | 45.5 | 10 | 18 | 10 | 0.98 | 3.6% |
| B5DFB7 | Nat13 protein | 19.388 | 49.955 | 56.8 | 8 | 11 | 8 | 0.98 | 3.2% |
| Q5XIQ8 | Chondroitin polymerizing factor | 85.343 | 3.7679 | 1.4 | 1 | 1 | 1 | 0.98 | 8.9% |
| P14408 | Fumarate hydratase, mitochondrial | 54.463 | 188.91 | 42.6 | 16 | 34 | 16 | 0.98 | 0.9% |
| Q6Q0N3 | 5-nucleotidase domain-containing protein 2 | 63.652 | 27.954 | 12.7 | 7 | 8 | 7 | 0.98 | 0.3% |
| Q80WL2 | Bystin | 50.011 | 3.1456 | 2.1 | 1 | 1 | 1 | 0.98 | 0.1% |
| Q6AXY7 | Pre-mRNA-splicing factor 38B | 63.931 | 5.2466 | 3 | 2 | 2 | 2 | 0.98 | 1.3% |
| P62083 | 40S ribosomal protein S7 | 22.127 | 64.173 | 29.4 | 9 | 15 | 9 | 0.98 | 2.1% |
| A0A0G2KAW5 | Protein Wdr48 | 77.586 | 7.9442 | 3.6 | 2 | 2 | 2 | 0.98 | 4.1% |
| A0A0G2K7M2 | Protein Rad23a | 39.538 | 37.133 | 18.2 | 6 | 8 | 4 | 0.98 | 9.6% |
| Q5EB59 | Mediator of RNA polymerase II transcription subunit 23 | 156.23 | 4.3908 | 1.3 | 1 | 1 | 1 | 0.98 | 6.0% |
| Q6AXX4 | Something about silencing protein 10 | 53.923 | 3.0524 | 2.8 | 1 | 1 | 1 | 0.98 | 1.8% |
| P18645 | UDP-glucose 4-epimerase | 38.224 | 64.19 | 32.6 | 8 | 9 | 8 | 0.98 | 2.4% |
| P61227 | Ras-related protein Rap-2b | 20.504 | 32.376 | 30.6 | 5 | 9 | 4 | 0.98 | 4.8% |
| Q9WTT7 | Basic leucine zipper and W2 domain-containing protein 2 | 48.049 | 43.121 | 16.5 | 7 | 7 | 6 | 0.98 | 4.7% |
| D4A7D7 | Hexose-6-phosphate dehydrogenase (Glucose 1-dehydrogenase) (Predicted), isoform CRA_b | 89.839 | 23.652 | 7 | 5 | 6 | 5 | 0.98 | 1.0% |
| D3ZDE4 | Deoxyguanosine kinase (Predicted), isoform CRA_a | 32.227 | 62.055 | 14.4 | 3 | 3 | 3 | 0.98 | 0.9% |
| Q02356 | AMP deaminase 2 | 94.786 | 13.651 | 6.9 | 5 | 6 | 5 | 0.98 | 0.8% |
| Q5EAJ6 | Inhibitor of nuclear factor kappa-B kinase-interacting protein | 42.36 | 97.643 | 37 | 11 | 26 | 11 | 0.98 | 0.5% |
| A1L1J8 | Protein Rab5b | 23.674 | 56.304 | 53.5 | 8 | 21 | 5 | 0.98 | 0.1% |
| F1M0X7 | Protein Ckap5 | 218.56 | 130.67 | 13.5 | 26 | 32 | 26 | 0.98 | 0.9% |
| A0A0G2JXT6 | Protein Mtmr6 | 75.576 | 24.446 | 11.8 | 7 | 8 | 7 | 0.98 | 4.6% |
| Q5XI21 | Protein Tom1 | 54.088 | 70.349 | 17.1 | 7 | 10 | 7 | 0.98 | 5.8% |
| A0A0G2JZB6 |  | 15.797 | 5.3574 | 16.6 | 1 | 1 | 1 | 0.98 | 8.7% |
| Q4KM08 | ATP-binding cassette, sub-family G (WHITE), member 3-like 1 | 73.072 | 22.628 | 10.4 | 5 | 7 | 1 | 0.98 | 6.1% |
| A0A0G2K8V3 | Protein Thoc2 | 185.06 | 10.655 | 2.2 | 3 | 3 | 3 | 0.98 | 3.7% |
| D4ABC4 | Protein Smek1 | 82.373 | 10.31 | 3.7 | 2 | 3 | 2 | 0.98 | 1.5% |
| D4AE49 | Protein Skiv2l2 | 120.7 | 28.165 | 6.2 | 7 | 7 | 7 | 0.98 | 1.3% |
| A0A0G2K1Z9 | Histocompatibility 13 (Predicted), isoform CRA_d | 41.775 | 13.615 | 7.7 | 3 | 6 | 3 | 0.98 | 0.1% |
| M0R3M8 | Protein Rrp12 | 143.13 | 41.107 | 6.6 | 7 | 8 | 7 | 0.98 | 0.9% |
| D3ZLC1 | Protein Lmnb2 | 68.85 | 120.1 | 40.4 | 21 | 31 | 19 | 0.98 | 1.0% |
| Q8VID1 | Dehydrogenase/reductase SDR family member 4 | 29.821 | 28.097 | 18.3 | 5 | 7 | 5 | 0.98 | 1.3% |
| Q4KLH7 | Protein Rad21 | 71.991 | 27.25 | 5.7 | 3 | 3 | 3 | 0.98 | 3.7% |
| F1LZD9 | Protein LOC102554700 | 37.667 | 93.928 | 23.7 | 5 | 8 | 5 | 0.98 | 4.7% |
| Q8K3P7 | Histidine triad nucleotide-binding protein 3 | 19.693 | 6.163 | 13.7 | 2 | 2 | 2 | 0.98 | 4.7% |
| Q9Z1Z6 | Integrin-linked kinase-associated serine/threonine phosphatase 2C | 42.744 | 13.858 | 9.2 | 4 | 4 | 4 | 0.98 | 3.4% |
| D4A3E8 | Mitochondrial ribosomal protein S27 (Predicted), isoform CRA_b | 47.648 | 39.425 | 11.6 | 4 | 5 | 4 | 0.98 | 3.2% |
| D3ZH75 | AKT1 substrate 1 (Proline-rich) (Predicted), isoform CRA_d | 27.586 | 20.476 | 16.7 | 4 | 6 | 4 | 0.98 | 6.4% |
| Q6MG49 | Large proline-rich protein BAG6 | 120.01 | 70.893 | 11.1 | 11 | 13 | 11 | 0.98 | 4.6% |
| D3ZAF9 | Protein Sh3pxd2b | 88.929 | 48.045 | 15.4 | 12 | 13 | 11 | 0.98 | 4.4% |
| P08081 | Clathrin light chain A | 26.98 | 20.228 | 23.8 | 9 | 13 | 9 | 0.98 | 3.0% |
| P17955 | Nuclear pore glycoprotein p62 | 53.396 | 30.699 | 9.5 | 4 | 6 | 4 | 0.98 | 1.2% |
| Q5XIN4 | Myotubularin related protein 9 | 63.404 | 30.545 | 13.7 | 7 | 8 | 7 | 0.98 | 0.0% |
| D3ZUM4 | Beta-galactosidase | 73.227 | 136.31 | 24.9 | 12 | 34 | 12 | 0.98 | 1.4% |
| P14604 | Enoyl-CoA hydratase, mitochondrial | 31.516 | 122.27 | 52.4 | 14 | 24 | 14 | 0.98 | 2.1% |
| Q99ML5 | Prenylcysteine oxidase | 56.287 | 168.1 | 32.3 | 12 | 23 | 12 | 0.98 | 2.0% |
| Q9Z2Q7 | Syntaxin-8 | 26.91 | 49.388 | 21.2 | 3 | 4 | 3 | 0.98 | 6.1% |
| D3ZU63 | Protein Stard13 | 124.76 | 2.741 | 1.3 | 1 | 2 | 1 | 0.98 | 5.9% |
| Q32KK2 | Arylsulfatase A | 53.731 | 56.627 | 17.2 | 5 | 5 | 5 | 0.98 | 19.2% |
| Q5PPK1 | Brain protein I3 | 13.613 | 2.6989 | 5.6 | 1 | 1 | 1 | 0.98 | 2.2% |
| D4AA64 | Protein Zc3h7b | 111.19 | 13.972 | 1.4 | 1 | 2 | 1 | 0.98 | 0.7% |
| D4A8G7 | Protein Snw1 | 61.463 | 41.705 | 11.6 | 5 | 5 | 5 | 0.98 | 0.5% |
| Q6P6Q9 | Calcium uptake protein 1, mitochondrial | 54.194 | 28.599 | 17 | 8 | 9 | 8 | 0.98 | 0.5% |
| F1MAQ3 | Protein Hgsnat | 72.151 | 10.216 | 2 | 1 | 1 | 1 | 0.98 | 3.1% |
| M0R5G2 | Protein Gemin7 | 15.883 | 2.1157 | 7.7 | 1 | 1 | 1 | 0.98 | 10.1% |
| Q63530 | Phosphotriesterase-related protein | 39.145 | 19.051 | 15.5 | 5 | 8 | 5 | 0.98 | 5.7% |
| Q642G4 | Peroxisomal membrane protein PEX14 | 40.936 | 81.914 | 20.2 | 6 | 8 | 6 | 0.98 | 4.9% |
| P62853 | 40S ribosomal protein S25 | 13.742 | 9.9458 | 28 | 4 | 9 | 4 | 0.98 | 1.9% |
| Q9EQR2 | Alkyldihydroxyacetonephosphate synthase, peroxisomal | 71.587 | 73.309 | 22.4 | 10 | 15 | 10 | 0.98 | 1.7% |
| P61354 | 60S ribosomal protein L27 | 15.798 | 87.593 | 51.5 | 8 | 21 | 8 | 0.98 | 1.6% |
| D4A768 | Protein Cmtm7 | 17.891 | 15.88 | 15.2 | 1 | 2 | 1 | 0.98 | 1.1% |
| A0A0G2JWG6 | Protein Golgb1 | 369.07 | 323.31 | 20.9 | 55 | 66 | 55 | 0.98 | 0.6% |
| Q9Z2S9 | Flotillin-2 | 47.037 | 153.06 | 43.2 | 16 | 22 | 16 | 0.98 | 1.6% |
| A9UMV9 | Ndufa7 protein | 12.5 | 25.018 | 40.2 | 5 | 7 | 5 | 0.98 | 3.9% |
| Q63965 | Sideroflexin-1 | 35.546 | 102.72 | 38.2 | 9 | 16 | 8 | 0.98 | 5.0% |
| D4A401 | Protein Tex10 | 105.28 | 12.229 | 5.1 | 4 | 4 | 4 | 0.98 | 5.3% |
| Q2KJ09 | Ubiquitin carboxyl-terminal hydrolase 16 | 93.761 | 2.5258 | 1.3 | 1 | 1 | 1 | 0.98 | 7.6% |
| F1LSC3 | Protein Sf1 | 68.346 | 27.002 | 9.2 | 6 | 8 | 6 | 0.98 | 7.1% |
| Q3B8Q2 | Eukaryotic initiation factor 4A-III | 46.84 | 44.223 | 29 | 12 | 21 | 9 | 0.98 | 6.6% |
| O09027 | Atypical chemokine receptor 2 | 43.293 | -2 | 4.2 | 1 | 1 | 1 | 0.98 | 6.5% |
| P61959 | Small ubiquitin-related modifier 2 | 10.871 | 14.124 | 27.4 | 3 | 5 | 2 | 0.98 | 5.8% |
| F1LYQ8 | FERM, RhoGEF and pleckstrin domain-containing protein 1 | 118.84 | 92.348 | 14.2 | 12 | 13 | 12 | 0.98 | 1.5% |
| P62628 | Dynein light chain roadblock-type 1 | 10.99 | 40.92 | 34.4 | 2 | 6 | 2 | 0.98 | 1.2% |
| D3ZU83 | ERGIC and golgi 3 (Predicted) | 43.223 | 26.613 | 13.6 | 6 | 7 | 6 | 0.98 | 1.0% |
| F1LNJ2 | U5 small nuclear ribonucleoprotein 200 kDa helicase | 244.87 | 129.39 | 11.2 | 19 | 23 | 19 | 0.98 | 2.0% |
| F1LVV3 | Protein Ranbp9 | 74.201 | 28.567 | 4.3 | 2 | 3 | 2 | 0.98 | 5.4% |
| Q6AYS3 | Protective protein for beta-galactosidase | 51.215 | 78.986 | 19.5 | 8 | 21 | 8 | 0.98 | 3.2% |
| Q6UK00 | Promethin | 17.578 | 20.725 | 8.1 | 1 | 2 | 1 | 0.98 | 2.4% |
| Q1RP77 | Nucleolar protein 16 | 21.117 | 4.0471 | 7.9 | 2 | 3 | 2 | 0.98 | 5.4% |
| D3ZL50 | Protein Ttc37 | 174.29 | 65.921 | 9.3 | 12 | 15 | 12 | 0.98 | 4.9% |
| B0BN68 | Mitochondrial ribosomal protein S9 | 45.01 | 17.905 | 12.3 | 4 | 4 | 4 | 0.98 | 2.6% |
| Q63610 | Tropomyosin alpha-3 chain | 29.006 | 174.23 | 82.3 | 39 | 168 | 20 | 0.98 | 1.8% |
| Q9R0T3 | DnaJ homolog subfamily C member 3 | 57.56 | 160.3 | 33.7 | 18 | 25 | 18 | 0.98 | 1.6% |
| Q9ES67 | Rho guanine nucleotide exchange factor 11 | 168.53 | 31.202 | 2.2 | 3 | 3 | 3 | 0.98 | 1.1% |
| P70615 | Lamin-B1 | 66.606 | 136.17 | 41.1 | 27 | 42 | 25 | 0.98 | 0.7% |
| Q4FZV0 | Beta-mannosidase | 101.22 | 45.785 | 9.8 | 7 | 8 | 7 | 0.98 | 1.4% |
| Q5U2V2 | Protein Serinc3 | 52.321 | 14.585 | 2.8 | 2 | 3 | 2 | 0.98 | 2.0% |
| P17702 | 60S ribosomal protein L28 | 15.848 | 4.2195 | 49.6 | 9 | 24 | 2 | 0.98 | 4.2% |
| A0A0G2JVC2 | Protein Scyl2 | 103.77 | 12.088 | 2.7 | 2 | 2 | 2 | 0.98 | 5.7% |
| A0A096MJN4 | Protein Sept4 | 53.083 | 21.63 | 13.3 | 6 | 11 | 5 | 0.98 | 2.9% |
| P15087 | Carboxypeptidase E | 53.308 | 15.837 | 9.5 | 4 | 5 | 4 | 0.98 | 7.7% |
| Q4KM98 | Mitochondrial fission factor | 24.97 | 17.295 | 31.7 | 3 | 3 | 3 | 0.98 | 9.6% |
| Q63100 | Cytoplasmic dynein 1 intermediate chain 1 | 72.753 | 21.691 | 9.3 | 4 | 4 | 4 | 0.98 | 5.8% |
| M0R402 | Protein Tmx3 | 52.1 | 53.348 | 21.4 | 10 | 22 | 10 | 0.98 | 0.5% |
| F1LXE1 | Protein Sptbn5 | 400.76 | 3.2888 | 0.9 | 3 | 3 | 3 | 0.98 | 0.9% |
| B0BNB1 | Commd1 protein | 21.037 | 25.482 | 20.2 | 4 | 4 | 4 | 0.98 | 1.8% |
| P54290 | Voltage-dependent calcium channel subunit alpha-2/delta-1 | 123.82 | 83.261 | 16.6 | 16 | 17 | 16 | 0.98 | 2.1% |
| D3ZGY1 | Protein Wibg | 22.726 | 1.8897 | 3.9 | 1 | 1 | 1 | 0.98 | 4.5% |
| F7F6T5 | Protein Serpinb6b | 42.688 | 10.58 | 13.8 | 6 | 7 | 4 | 0.98 | 4.0% |
| D3ZKC8 | Protein Vangl1 | 60.104 | 8.1859 | 2.1 | 1 | 1 | 1 | 0.98 | 3.9% |
| Q6AY30 | Saccharopine dehydrogenase-like oxidoreductase | 47.088 | 73.796 | 37.1 | 11 | 19 | 11 | 0.98 | 3.1% |
| Q9ESV1 | Leucine zipper protein 1 | 117.17 | 173.49 | 28.2 | 25 | 30 | 25 | 0.98 | 0.6% |
| Q5U312 | Ankycorbin | 109.13 | 323.31 | 49 | 52 | 81 | 52 | 0.98 | 0.3% |
| B2RZ68 | Protein Dcaf7 | 38.926 | 37.393 | 17.8 | 5 | 6 | 5 | 0.98 | 0.6% |
| B0K008 | Eukaryotic translation initiation factor 1 | 12.746 | 80.585 | 61.1 | 5 | 8 | 5 | 0.98 | 1.5% |
| D3ZWW5 | Protein Slc30a9 | 62.684 | 43.526 | 11.1 | 6 | 6 | 6 | 0.98 | 5.3% |
| B0BNB5 | LOC683983 protein | 41.806 | 15.94 | 3.7 | 1 | 1 | 1 | 0.98 | 13.1% |
| P62603 | Tripartite motif-containing protein 26 | 62.63 | 19.25 | 7.4 | 3 | 3 | 3 | 0.98 | 1.2% |
| Q5U3Z7 | Serine hydroxymethyltransferase | 55.764 | 136.22 | 40.9 | 19 | 35 | 17 | 0.98 | 0.1% |
| Q3KRD5 | Mitochondrial import receptor subunit TOM34 | 34.461 | 60.879 | 25.9 | 7 | 10 | 7 | 0.98 | 0.7% |
| Q6AXU6 | Hematological and neurological expressed 1 protein | 15.575 | 38.244 | 26.8 | 3 | 3 | 3 | 0.98 | 1.4% |
| P70541 | Translation initiation factor eIF-2B subunit gamma | 50.435 | 30.88 | 19.5 | 9 | 10 | 9 | 0.98 | 2.3% |
| Q8VHI8 | Vesicle transport protein SEC20 | 26.17 | 79.182 | 26.8 | 5 | 10 | 5 | 0.98 | 5.7% |
| Q9QXU8 | Cytoplasmic dynein 1 light intermediate chain 1 | 56.792 | 153.91 | 38.4 | 19 | 28 | 19 | 0.98 | 2.4% |
| M0RDC9 | Protein Exosc1 | 21.407 | 3.4553 | 16.9 | 2 | 2 | 2 | 0.98 | 4.2% |
| Q6AYJ1 | ATP-dependent DNA helicase Q1 | 69.641 | 17.433 | 10.5 | 7 | 8 | 7 | 0.98 | 4.4% |
| Q03346 | Mitochondrial-processing peptidase subunit beta | 54.265 | 141.09 | 25.4 | 11 | 12 | 10 | 0.98 | 2.3% |
| Q5EB77 | Ras-related protein Rab-18 | 22.976 | 103.15 | 57.3 | 10 | 26 | 10 | 0.98 | 2.3% |
| P18163 | Long-chain-fatty-acid--CoA ligase 1 | 78.178 | 91.686 | 27.3 | 17 | 21 | 15 | 0.98 | 1.0% |
| Q63616 | Vacuolar protein sorting-associated protein 33B | 70.693 | 18.79 | 8.6 | 5 | 5 | 5 | 0.98 | 0.2% |
| D4AB03 | Protein Fam120a | 122.14 | 147.35 | 27.3 | 24 | 35 | 24 | 0.98 | 0.5% |
| Q3LUD4 | Leucine zipper putative tumor suppressor 2 | 72.492 | 13.108 | 5.5 | 3 | 3 | 3 | 0.98 | 3.4% |
| E9PT51 | DNA-directed polymerase delta interacting protein 2 | 41.797 | 17.675 | 10.9 | 4 | 8 | 4 | 0.98 | 3.8% |
| F1LRK4 | Protein Grsf1 | 52.907 | 24.604 | 11.1 | 4 | 4 | 4 | 0.98 | 5.9% |
| O08781 | Zinc finger matrin-type protein 3 | 31.918 | 15.925 | 6.2 | 1 | 1 | 1 | 0.98 | 10.8% |
| A0A0G2K0C1 | Protein Ces2 | 62.167 | 18.806 | 6.4 | 3 | 4 | 3 | 0.98 | 5.3% |
| M0R665 | 60S ribosomal protein L29 | 17.345 | 7.1047 | 21.8 | 4 | 6 | 4 | 0.98 | 4.5% |
| O08815 | STE20-like serine/threonine-protein kinase | 137.89 | 64.754 | 13.9 | 15 | 18 | 14 | 0.98 | 3.7% |
| D3ZWL9 | Protein Dido1 | 243.29 | 5.6057 | 1.4 | 3 | 3 | 3 | 0.98 | 2.4% |
| D3ZEG8 | Protein RGD1309188 | 29.873 | 14.417 | 17.6 | 4 | 4 | 4 | 0.98 | 1.3% |
| A0A0G2K681 | Protein Clip1 | 159.46 | 185.52 | 21.9 | 28 | 40 | 24 | 0.98 | 0.2% |
| G3V9T7 | ATPase Asna1 | 38.822 | 59.757 | 23 | 6 | 8 | 6 | 0.98 | 0.2% |
| P54313 | Guanine nucleotide-binding protein G(I)/G(S)/G(T) subunit beta-2 | 37.331 | 161.66 | 43.8 | 12 | 40 | 6 | 0.98 | 0.3% |
| Q62915 | Peripheral plasma membrane protein CASK | 103.26 | 65.734 | 15.1 | 13 | 16 | 13 | 0.98 | 1.1% |
| Q66H39 | ATP-binding cassette sub-family F member 3 | 79.855 | 4.1112 | 2.8 | 2 | 2 | 2 | 0.98 | 2.2% |
| O88813 | Long-chain-fatty-acid--CoA ligase 5 | 76.404 | 104.62 | 23.9 | 14 | 18 | 12 | 0.98 | 2.7% |
| D3ZHU8 | Protein Rbm19 | 106.36 | 26.108 | 2.8 | 2 | 2 | 2 | 0.98 | 11.5% |
| Q5I0L7 | Protein KTI12 homolog | 38.357 | 8.0478 | 5.7 | 1 | 1 | 1 | 0.98 | 3.1% |
| Q9QZM5 | Abl interactor 1 | 51.704 | 62.65 | 23.5 | 10 | 11 | 10 | 0.98 | 0.7% |
| G3V9T1 | Protein Crispld2 | 55.804 | 5.2769 | 3.8 | 2 | 5 | 2 | 0.98 | 0.1% |
| D3ZQ57 | Plexin B2 | 205.95 | 173.13 | 15.9 | 26 | 37 | 26 | 0.98 | 1.3% |
| Q4VBH2 | Protein LOC100911093 | 49.821 | 30.057 | 10.4 | 4 | 6 | 4 | 0.98 | 2.8% |
| D4A4K6 | Protein Nrap | 196.3 | 3.471 | 0.8 | 1 | 1 | 1 | 0.98 | 3.5% |
| Q4V8C2 | Centromere/kinetochore protein zw10 homolog | 87.966 | 110.43 | 22.5 | 13 | 14 | 13 | 0.98 | 4.0% |
| Q5EIC4 | Interferon regulatory factor 2-binding protein-like | 81.495 | 30.53 | 6.4 | 4 | 4 | 4 | 0.98 | 6.0% |
| P32198 | Carnitine O-palmitoyltransferase 1, liver isoform | 88.124 | 53.414 | 16.2 | 12 | 15 | 12 | 0.98 | 6.6% |
| F1M6T6 | Protein Ppp6r2 | 97.641 | 2.5813 | 1.9 | 1 | 2 | 1 | 0.98 | 16.5% |
| A0A0G2JUS5 | Dolichyl pyrophosphate phosphatase 1 (Predicted), isoform CRA_b | 27.069 | 12.795 | 5.5 | 1 | 2 | 1 | 0.98 | 6.2% |
| P97690 | Structural maintenance of chromosomes protein 3 | 138.45 | 55.334 | 10.4 | 10 | 11 | 10 | 0.98 | 2.2% |
| D3ZZC1 | Protein Txndc5 | 46.353 | 211.13 | 35 | 15 | 29 | 15 | 0.98 | 2.1% |
| O70257 | Syntaxin-7 | 29.85 | 107.77 | 52.9 | 10 | 17 | 10 | 0.98 | 2.1% |
| Q99J82 | Integrin-linked protein kinase | 51.373 | 62.747 | 33.4 | 16 | 41 | 16 | 0.98 | 1.6% |
| Q566E4 | Heterogeneous nuclear ribonucleoprotein R | 70.873 | 115.77 | 31.6 | 20 | 32 | 15 | 0.98 | 0.7% |
| Q9JLT0 | Myosin-10 | 228.96 | 323.31 | 55.1 | 121 | 305 | 94 | 0.98 | 0.3% |
| Q63072 | ADP-ribosyl cyclase/cyclic ADP-ribose hydrolase 2 | 35.131 | 23.549 | 12.9 | 4 | 5 | 4 | 0.98 | 0.5% |
| Q63028 | Alpha-adducin | 80.354 | 8.2783 | 4.6 | 3 | 3 | 3 | 0.98 | 5.4% |
| Q99PV3 | Muskelin | 84.833 | 7.1111 | 3.7 | 3 | 3 | 3 | 0.98 | 11.5% |
| Q2LC84 | Protein numb homolog | 70.595 | 38.704 | 12.6 | 8 | 9 | 7 | 0.98 | 3.4% |
| O35777 | Magnesium transporter protein 1 | 37.992 | 9.6475 | 5.7 | 3 | 6 | 3 | 0.98 | 5.1% |
| M0RBW5 | Protein Ccdc40 | 143.44 | 2.3589 | 1 | 1 | 3 | 1 | 0.98 | 2.6% |
| D3ZU13 | Protein Eif4g1 | 175.7 | 245.96 | 28.1 | 40 | 62 | 35 | 0.98 | 2.1% |
| M0RCA3 | Protein Zfpl1 | 34.253 | 25.923 | 16.5 | 5 | 7 | 5 | 0.98 | 2.1% |
| Q6P742 | Proteolipid protein 2 | 16.556 | 8.3938 | 17.9 | 2 | 3 | 2 | 0.98 | 1.9% |
| Q62991 | Sec1 family domain-containing protein 1 | 72.262 | 177.84 | 26.1 | 12 | 23 | 12 | 0.98 | 1.3% |
| B0BN20 | Tetraspanin | 27.535 | 11.979 | 10.2 | 2 | 2 | 2 | 0.98 | 0.3% |
| P26284 | Pyruvate dehydrogenase E1 component subunit alpha, somatic form, mitochondrial | 43.226 | 158.43 | 49.2 | 21 | 35 | 21 | 0.98 | 0.3% |
| P24473 | Glutathione S-transferase kappa 1 | 25.493 | 45.107 | 37.2 | 7 | 12 | 7 | 0.98 | 0.8% |
| P35434 | ATP synthase subunit delta, mitochondrial | 17.595 | 16.911 | 13.7 | 2 | 4 | 2 | 0.98 | 0.8% |
| D4A830 | Protein Ppa2 | 35.046 | 65.263 | 23.7 | 6 | 8 | 6 | 0.98 | 1.3% |
| F1M8X9 | Protein Gbf1 | 206.52 | 146.36 | 12.7 | 20 | 25 | 20 | 0.98 | 3.0% |
| Q569B7 | RWD domain-containing protein 4 | 21.114 | 12.162 | 20.2 | 4 | 5 | 4 | 0.98 | 4.1% |
| D3ZC46 | Protein Tcf25 | 75.32 | 11.948 | 4.8 | 3 | 4 | 3 | 0.98 | 4.9% |
| D4A7H9 | CHMP family, member 7 (Predicted), isoform CRA_a | 50.543 | 18.753 | 12.7 | 4 | 4 | 4 | 0.97 | 5.0% |
| D4A5A6 | DNA-directed RNA polymerase | 217.2 | 11.796 | 2.7 | 3 | 4 | 3 | 0.97 | 4.8% |
| M0R762 | Protein Smg1 | 410.43 | 7.3487 | 0.9 | 3 | 3 | 3 | 0.97 | 3.0% |
| Q66HC5 | Nuclear pore complex protein Nup93 | 93.301 | 58.376 | 20 | 15 | 15 | 15 | 0.97 | 2.2% |
| P07153 | Dolichyl-diphosphooligosaccharide--protein glycosyltransferase subunit 1 | 68.303 | 323.31 | 51.1 | 32 | 82 | 32 | 0.97 | 0.9% |
| P31977 | Ezrin | 69.39 | 55.535 | 37 | 22 | 56 | 10 | 0.97 | 0.8% |
| A0A096MJR6 | Protein Parp4 (Fragment) | 89.891 | 19.752 | 5.5 | 4 | 6 | 4 | 0.97 | 0.3% |
| B1H227 | LOC682908 protein | 45.111 | 4.8207 | 4.3 | 2 | 2 | 2 | 0.97 | 0.5% |
| E9PTI6 | Protein Raly | 33.054 | 14.562 | 11.9 | 4 | 5 | 4 | 0.97 | 0.7% |
| D3ZY50 | ATP synthase mitochondrial F1 complex assembly factor 1 (Predicted), isoform CRA_a | 38.649 | 3.5395 | 2.6 | 2 | 2 | 2 | 0.97 | 0.8% |
| B2GV98 | Lrch1 protein | 75.211 | 38.728 | 9.2 | 5 | 6 | 5 | 0.97 | 2.1% |
| A0A0G2K1E2 | Integrin alpha 5 (Mapped) | 119.46 | 21.593 | 6.3 | 6 | 7 | 6 | 0.97 | 2.3% |
| G3V629 | Protein Ppp1r18 | 65.568 | 124.32 | 41.2 | 19 | 24 | 19 | 0.97 | 3.7% |
| D3ZDP2 | Protein Ict1 | 23.47 | 13.879 | 21.8 | 4 | 4 | 4 | 0.97 | 4.8% |
| Q6PDU7 | ATP synthase subunit g, mitochondrial | 11.433 | 15.356 | 34 | 3 | 5 | 3 | 0.97 | 4.8% |
| O70188 | Nuclear factor 1 | 48.742 | 6.8256 | 4.6 | 2 | 2 | 2 | 0.97 | 5.0% |
| A0A0G2K677 | Protein Sptbn4 | 288.81 | -2 | 1.9 | 4 | 10 | 1 | 0.97 | 17.8% |
| M0R6J0 | Protein Mrpl39 | 38.374 | 27.786 | 12.8 | 4 | 5 | 4 | 0.97 | 6.2% |
| Q6AY87 | THO complex subunit 6 homolog | 37.422 | 12.526 | 10.3 | 3 | 3 | 3 | 0.97 | 2.8% |
| B0BNG0 | ER membrane protein complex subunit 2 | 34.869 | 48.247 | 23.6 | 6 | 11 | 6 | 0.97 | 2.7% |
| Q2M1K6 | Zinc transporter ZIP13 | 38.397 | 2.1121 | 2.5 | 1 | 1 | 1 | 0.97 | 2.4% |
| D4AEJ5 | Protein Wwc2 | 133.05 | 29.089 | 5 | 5 | 5 | 5 | 0.97 | 2.0% |
| Q5XII0 | Mammalian ependymin-related protein 1 | 25.639 | 39.603 | 28.6 | 6 | 14 | 6 | 0.97 | 1.4% |
| A0A0G2JX93 | Protein Stat1 | 125.24 | 134.13 | 21.2 | 21 | 35 | 21 | 0.97 | 0.1% |
| Q9WTN5 | Two pore calcium channel protein 1 | 94.404 | 15.372 | 5.1 | 3 | 3 | 3 | 0.97 | 1.5% |
| A0JPQ9 | Chitinase domain-containing protein 1 | 44.874 | 130.26 | 31 | 10 | 13 | 10 | 0.97 | 1.8% |
| B2GV72 | Carbonyl reductase 3 | 30.841 | 55.137 | 41.9 | 8 | 11 | 7 | 0.97 | 2.1% |
| Q9ET50 | Protein Stau1 | 54.801 | 50.702 | 17.2 | 7 | 11 | 7 | 0.97 | 3.4% |
| B5DF91 | ELAV-like protein | 36.169 | 36.483 | 25.8 | 8 | 12 | 8 | 0.97 | 4.2% |
| Q5XIW8 | U4/U6.U5 tri-snRNP-associated protein 1 | 91.01 | 79.507 | 11.2 | 7 | 8 | 7 | 0.97 | 5.1% |
| A0A0G2K5K7 | Protein Inpp5k | 52.118 | 28.582 | 13.7 | 6 | 10 | 6 | 0.97 | 2.1% |
| P69682 | Adaptin ear-binding coat-associated protein 1 | 29.792 | 16.134 | 23.1 | 4 | 5 | 4 | 0.97 | 0.9% |
| F1MAQ4 | Protein Trappc10 | 141.66 | 13.936 | 1.9 | 2 | 2 | 2 | 0.97 | 0.4% |
| B4F7A9 | Casein kinase 2, alpha prime polypeptide | 41.201 | 41.934 | 22.6 | 7 | 9 | 7 | 0.97 | 0.4% |
| E9PT66 | Protein Sf3b3 | 135.55 | 81.808 | 15.4 | 16 | 21 | 16 | 0.97 | 1.7% |
| P61131 | CMP-N-acetylneuraminate-beta-galactosamide-alpha-2,3-sialyltransferase 4 | 38.043 | 7.2786 | 7.2 | 2 | 2 | 2 | 0.97 | 5.2% |
| D3ZFE9 | Protein LOC100359687 | 36.895 | 6.3576 | 6.1 | 2 | 3 | 2 | 0.97 | 7.8% |
| Q5PPM8 | Type 1 phosphatidylinositol 4,5-bisphosphate 4-phosphatase | 29.993 | 10.401 | 13.7 | 4 | 5 | 3 | 0.97 | 6.0% |
| P21775 | 3-ketoacyl-CoA thiolase A, peroxisomal | 43.833 | 151.73 | 43.6 | 12 | 18 | 12 | 0.97 | 3.0% |
| B2RYT7 | Haloacid dehalogenase-like hydrolase domain containing 3 | 27.794 | 10.18 | 12.4 | 4 | 4 | 4 | 0.97 | 2.0% |
| D4ABM3 | Dishevelled associated activator of morphogenesis 1 (Predicted) | 112.64 | 20.426 | 8.7 | 8 | 9 | 8 | 0.97 | 0.1% |
| P31399 | ATP synthase subunit d, mitochondrial | 18.763 | 113.38 | 72 | 10 | 24 | 10 | 0.97 | 0.0% |
| Q5U367 | Procollagen-lysine,2-oxoglutarate 5-dioxygenase 3 | 85.059 | 178.17 | 29.1 | 19 | 35 | 18 | 0.97 | 0.3% |
| G3V9D0 | Protein O-glucosyltransferase 1 | 46.509 | 45.68 | 17.3 | 6 | 10 | 6 | 0.97 | 0.4% |
| P83565 | 39S ribosomal protein L40, mitochondrial | 24.397 | 21.711 | 25.7 | 4 | 6 | 4 | 0.97 | 0.9% |
| Q6AXS5 | Plasminogen activator inhibitor 1 RNA-binding protein | 44.754 | 94.959 | 41 | 16 | 24 | 16 | 0.97 | 2.7% |
| A0A0G2JZ40 | Protein Reck | 105.97 | 16.469 | 5.6 | 5 | 5 | 5 | 0.97 | 0.2% |
| Q66HD0 | Endoplasmin | 92.77 | 323.31 | 54.4 | 50 | 151 | 48 | 0.97 | 0.1% |
| D4AAH9 | Protein Tbc1d23 | 78.386 | 14.187 | 4.4 | 3 | 3 | 3 | 0.97 | 1.9% |
| Q3ZAV8 | Enhancer of mRNA-decapping protein 4 | 152.59 | 147.67 | 14.1 | 14 | 18 | 14 | 0.97 | 1.9% |
| O08984 | Lamin-B receptor | 70.723 | 2.5555 | 3.4 | 2 | 2 | 2 | 0.97 | 2.9% |
| P07872 | Peroxisomal acyl-coenzyme A oxidase 1 | 74.678 | 21.479 | 7.9 | 3 | 4 | 3 | 0.97 | 4.9% |
| O88201 | C-type lectin domain family 11 member A | 36.387 | 2.2776 | 5.5 | 2 | 4 | 2 | 0.97 | 2.8% |
| P97588 | Mothers against decapentaplegic homolog 1 | 52.713 | 8.6307 | 10.7 | 4 | 4 | 2 | 0.97 | 2.3% |
| Q5XI72 | Eukaryotic translation initiation factor 4H | 27.324 | 35.969 | 33.1 | 9 | 14 | 9 | 0.97 | 1.5% |
| Q63686 | Cyclin-dependent kinase 16 | 55.764 | 3.8026 | 5.4 | 3 | 3 | 2 | 0.97 | 6.9% |
| Q5XIE0 | Acidic leucine-rich nuclear phosphoprotein 32 family member E | 29.418 | 11.989 | 4.7 | 1 | 1 | 1 | 0.97 | 9.3% |
| F1LP64 | E3 ubiquitin-protein ligase TRIP12 | 223.93 | 87.085 | 10.3 | 17 | 23 | 17 | 0.97 | 7.5% |
| Q5U1Y0 | Transmembrane protein 192 | 30.252 | 9.0494 | 11.3 | 3 | 4 | 3 | 0.97 | 5.2% |
| Q4V8K2 | Beta-catenin-like protein 1 | 64.947 | 20.424 | 11.9 | 7 | 8 | 7 | 0.97 | 2.6% |
| B2RYS8 | NADH dehydrogenase [ubiquinone] 1 beta subcomplex subunit 8, mitochondrial | 21.959 | 15.748 | 25.8 | 4 | 5 | 4 | 0.97 | 0.8% |
| P70483 | Striatin | 86.225 | 57.5 | 13.1 | 8 | 11 | 7 | 0.97 | 0.6% |
| P61589 | Transforming protein RhoA | 21.782 | 48.251 | 43.5 | 9 | 22 | 4 | 0.97 | 0.3% |
| F1LT35 | 60S ribosomal protein L23a | 17.734 | 39.134 | 49.4 | 10 | 27 | 10 | 0.97 | 1.3% |
| D3ZSA9 | Protein Nomo1 | 133.44 | 250.43 | 26 | 22 | 35 | 22 | 0.97 | 1.6% |
| Q6AXM8 | Serum paraoxonase/arylesterase 2 | 39.617 | 31.071 | 16.1 | 5 | 6 | 5 | 0.97 | 1.8% |
| Q6DGG9 | Calcium modulating ligand | 32.79 | 17.429 | 12.5 | 4 | 6 | 4 | 0.97 | 1.9% |
| A0A0G2JZ53 | Protein Birc6 | 434.3 | 51.987 | 1.9 | 7 | 8 | 7 | 0.97 | 3.3% |
| O35162 | Heat shock 70 kDa protein 13 | 51.795 | 28.414 | 13.4 | 6 | 7 | 6 | 0.97 | 4.9% |
| D3ZZ38 | Sorting nexin | 67.866 | 90.581 | 19.5 | 9 | 13 | 9 | 0.97 | 6.2% |
| A0A0G2JV05 | Protein Mroh1 | 181.91 | 3.9241 | 0.7 | 1 | 1 | 1 | 0.97 | 5.5% |
| Q5RK00 | 39S ribosomal protein L46, mitochondrial | 31.673 | 20.653 | 9.4 | 2 | 2 | 2 | 0.97 | 5.8% |
| D3ZSL1 | RNA binding protein fox-1 homolog | 44.555 | 8.4433 | 5.3 | 2 | 4 | 2 | 0.97 | 2.4% |
| Q5U1Z0 | Rab3 GTPase-activating protein non-catalytic subunit | 154.43 | 95.327 | 17.8 | 21 | 28 | 21 | 0.97 | 1.6% |
| Q80Z29 | Nicotinamide phosphoribosyltransferase | 55.437 | 39.521 | 20.8 | 9 | 11 | 9 | 0.97 | 0.0% |
| D3ZIH4 | F-box only protein 4 (Predicted) | 48.572 | 5.0427 | 4.6 | 2 | 2 | 2 | 0.97 | 0.4% |
| D3ZYR1 | FCH domain only protein 2 | 88.711 | 2.2181 | 2.2 | 2 | 2 | 2 | 0.97 | 1.3% |
| D4A8Z3 | Protein Frrs1 | 65.614 | 3.7756 | 2.4 | 2 | 2 | 2 | 0.97 | 1.6% |
| F1M5V2 | Protein Glipr2 | 18.985 | 113.62 | 48.2 | 6 | 9 | 6 | 0.97 | 2.2% |
| D3ZBN3 | Receptor protein-tyrosine kinase | 108.74 | 82.137 | 11.9 | 10 | 17 | 10 | 0.97 | 2.4% |
| M0RDI5 | Protein Mcu | 39.757 | 109.25 | 32.9 | 11 | 14 | 11 | 0.97 | 3.7% |
| Q5RJK8 | Acyl-CoA-binding domain-containing protein 6 | 30.81 | 10.965 | 14.5 | 3 | 3 | 3 | 0.97 | 3.9% |
| P97576 | GrpE protein homolog 1, mitochondrial | 24.297 | 62.321 | 41 | 8 | 10 | 8 | 0.97 | 3.3% |
| P06761 | 78 kDa glucose-regulated protein | 72.346 | 323.31 | 61.3 | 43 | 157 | 40 | 0.97 | 1.3% |
| Q641Z5 | Cation transport regulator-like protein 2 | 20.167 | 8.2308 | 7.3 | 1 | 2 | 1 | 0.97 | 1.3% |
| Q9JHU5 | Arfaptin-1 | 40.779 | 67.354 | 28.4 | 8 | 11 | 8 | 0.97 | 1.0% |
| Q4QR75 | Exosome complex component RRP45 | 48.881 | 10.567 | 4.8 | 2 | 3 | 2 | 0.97 | 0.1% |
| D3ZQ09 | Protein Zcchc24 | 26.99 | 17.888 | 11.2 | 2 | 2 | 2 | 0.97 | 0.6% |
| Q06647 | ATP synthase subunit O, mitochondrial | 23.397 | 101.06 | 57.7 | 11 | 27 | 11 | 0.97 | 0.8% |
| A0A0G2K2B5 | Protein Asph | 89.524 | 133.68 | 24.2 | 18 | 25 | 18 | 0.97 | 1.2% |
| F1M0U5 | Protein Nbas | 265.8 | 170.88 | 12.7 | 24 | 28 | 24 | 0.97 | 1.2% |
| P29995 | Inositol 1,4,5-trisphosphate receptor type 2 | 307.05 | 2.6035 | 3.4 | 11 | 14 | 1 | 0.97 | 7.9% |
| B2RYM2 | Protein Wipi1 | 48.727 | 7.8867 | 7 | 2 | 2 | 2 | 0.97 | 9.5% |
| P70580 | Membrane-associated progesterone receptor component 1 | 21.598 | 55.875 | 44.6 | 8 | 14 | 7 | 0.97 | 3.4% |
| Q5U1Z9 | Metaxin 2 | 29.718 | 16.06 | 17.9 | 5 | 7 | 5 | 0.97 | 3.3% |
| P42676 | Neurolysin, mitochondrial | 80.253 | 71.799 | 21.2 | 13 | 16 | 13 | 0.97 | 1.1% |
| D3ZZT9 | Protein Col14a1 | 192.56 | 29.984 | 3.5 | 5 | 6 | 5 | 0.97 | 0.9% |
| F1M5H6 | Protein Tp53bp2 | 125.31 | 38.721 | 2.6 | 2 | 2 | 2 | 0.97 | 0.7% |
| P05712 | Ras-related protein Rab-2A | 23.535 | 101.47 | 54.7 | 11 | 29 | 5 | 0.97 | 1.1% |
| D3ZAZ0 | Eukaryotic translation initiation factor 3 subunit M | 42.516 | 67.097 | 19 | 6 | 9 | 6 | 0.97 | 1.3% |
| B2GUZ5 | F-actin-capping protein subunit alpha-1 | 32.909 | 111.72 | 37.4 | 8 | 18 | 7 | 0.97 | 1.4% |
| Q91ZW1 | Transcription factor A, mitochondrial | 28.186 | 24.186 | 25.4 | 6 | 6 | 6 | 0.97 | 3.0% |
| D3ZNI6 | Protein Dnajc13 | 255.13 | 214.91 | 14.6 | 26 | 30 | 26 | 0.97 | 3.2% |
| Q4V8F9 | Hydroxysteroid dehydrogenase-like protein 2 | 58.343 | 86.525 | 15.1 | 6 | 9 | 6 | 0.97 | 3.3% |
| P97586 | Cell growth regulator with EF hand domain protein 1 | 30.834 | 60.707 | 17.4 | 4 | 7 | 4 | 0.97 | 3.9% |
| Q9Z327 | Synaptopodin | 99.985 | 16.189 | 2.7 | 2 | 2 | 2 | 0.97 | 4.8% |
| A0A0G2JZE7 | Protein Arhgef40 | 165.34 | 31.088 | 4.9 | 7 | 9 | 7 | 0.97 | 6.9% |
| G3V6D1 | Protein Nagpa | 55.688 | 18.48 | 7.2 | 3 | 3 | 3 | 0.97 | 5.7% |
| D4A5Z0 | Protein Csgalnact2 | 62.654 | 39.162 | 13.1 | 5 | 5 | 5 | 0.97 | 4.0% |
| Q63016 | Large neutral amino acids transporter small subunit 1 | 55.903 | 21.309 | 9 | 4 | 4 | 4 | 0.97 | 1.6% |
| Q9JHB5 | Translin-associated protein X | 33.005 | 96.15 | 38.6 | 10 | 13 | 10 | 0.97 | 1.0% |
| D3ZH41 | Cytoskeleton-associated protein 4 (Predicted) | 36.315 | 180.94 | 60.4 | 22 | 67 | 22 | 0.97 | 0.8% |
| Q811A3 | Procollagen-lysine,2-oxoglutarate 5-dioxygenase 2 | 84.541 | 255.4 | 44.1 | 31 | 69 | 30 | 0.97 | 1.5% |
| B4F7D6 | Protein Stk11ip | 118.13 | 33.118 | 5 | 4 | 5 | 4 | 0.97 | 2.8% |
| P34900 | Syndecan-2 | 22.149 | 5.6772 | 8.5 | 2 | 2 | 2 | 0.97 | 4.0% |
| A0A0G2JTA7 | Protein Rasal2 | 143.67 | 22.857 | 5.5 | 7 | 7 | 6 | 0.97 | 4.2% |
| Q5FWT5 | Glutamyl-tRNA(Gln) amidotransferase subunit A, mitochondrial | 56.837 | 10.324 | 3 | 1 | 1 | 1 | 0.97 | 5.7% |
| Q7TQ85 | Ac1164 | 58.796 | 35.187 | 11.5 | 6 | 7 | 6 | 0.97 | 4.1% |
| P32089 | Tricarboxylate transport protein, mitochondrial | 33.835 | 91.081 | 35.4 | 10 | 18 | 10 | 0.97 | 1.7% |
| Q6NYB7 | Ras-related protein Rab-1A | 22.677 | 225.11 | 76.1 | 16 | 60 | 9 | 0.97 | 1.2% |
| O55171 | Acyl-coenzyme A thioesterase 2, mitochondrial | 49.701 | 88.81 | 32.9 | 12 | 17 | 12 | 0.97 | 0.9% |
| Q498R3 | DnaJ homolog subfamily C member 10 | 90.746 | 69.708 | 20.7 | 16 | 24 | 16 | 0.97 | 0.7% |
| P26376 | Interferon-induced transmembrane protein 3 | 14.971 | 23.405 | 21.9 | 2 | 6 | 2 | 0.97 | 1.1% |
| D4A133 | Protein Atp6v1a | 68.264 | 121.79 | 34.7 | 17 | 26 | 17 | 0.97 | 2.0% |
| D3ZRM3 | Carbohydrate (Chondroitin) synthase 1 (Predicted) | 89.95 | 24.147 | 9.5 | 4 | 4 | 4 | 0.97 | 2.4% |
| Q05962 | ADP/ATP translocase 1 | 32.989 | 77.8 | 61.1 | 20 | 61 | 9 | 0.97 | 3.3% |
| P19491 | Glutamate receptor 2 | 98.687 | 13.205 | 6.1 | 4 | 4 | 4 | 0.97 | 11.9% |
| D3ZTB4 | Protein Vps11 | 101.17 | 29.919 | 9.9 | 7 | 7 | 7 | 0.97 | 8.2% |
| M0R5U3 | Protein Tatdn3 | 21.248 | 1.9301 | 4.2 | 1 | 1 | 1 | 0.97 | 5.9% |
| D4A031 | DEAD (Asp-Glu-Ala-Asp) box polypeptide 42 (Predicted) | 102.07 | 57.322 | 10.5 | 7 | 9 | 7 | 0.97 | 3.9% |
| Q07984 | Translocon-associated protein subunit delta | 18.979 | 33.665 | 19.7 | 3 | 13 | 3 | 0.97 | 4.2% |
| Q496Z1 | Bicaudal D homolog 2 (Drosophila) | 96.708 | 95.306 | 21 | 16 | 20 | 16 | 0.97 | 2.5% |
| Q923V8 | 15 kDa selenoprotein | 17.822 | 48.75 | 29.6 | 4 | 5 | 4 | 0.97 | 2.4% |
| P28023 | Dynactin subunit 1 | 141.93 | 311.34 | 37.3 | 40 | 57 | 40 | 0.97 | 0.7% |
| Q5RJN0 | NADH dehydrogenase (Ubiquinone) Fe-S protein 7 | 23.945 | 19.224 | 19.3 | 4 | 7 | 4 | 0.97 | 0.3% |
| A0A0G2K3Y6 | Protein Cpeb4 | 80.161 | 4.4321 | 1.5 | 1 | 1 | 1 | 0.97 | 0.1% |
| D3ZLX2 | Protein RGD1311783 | 11.663 | 27.897 | 28.3 | 2 | 2 | 2 | 0.97 | 3.8% |
| A0A0G2K459 | Protein Mtch2 | 33.757 | 71.312 | 29.7 | 7 | 8 | 7 | 0.97 | 4.4% |
| Q569C1 | Charged multivesicular body protein 4c | 26.323 | 19.336 | 17.7 | 3 | 4 | 3 | 0.97 | 11.9% |
| D3ZAI8 | Protein Pkd1l3 | 239.96 | 3.0568 | 0.7 | 2 | 2 | 2 | 0.97 | 9.6% |
| Q4QQU5 | Protein YIPF6 | 26.106 | 6.8058 | 9.3 | 2 | 2 | 2 | 0.97 | 8.1% |
| Q0KL00 | Piezo-type mechanosensitive ion channel component 1 | 290.31 | 87.428 | 4.2 | 8 | 12 | 8 | 0.97 | 6.8% |
| D3ZP13 | Sulfhydryl oxidase | 79.197 | 30.345 | 10 | 5 | 7 | 5 | 0.97 | 2.1% |
| Q63081 | Protein disulfide-isomerase A6 | 48.173 | 229.7 | 43.4 | 17 | 39 | 17 | 0.97 | 1.3% |
| D4AEC0 | Histone H2A | 13.509 | 37.162 | 31.2 | 5 | 12 | 3 | 0.97 | 1.2% |
| P07861 | Neprilysin | 85.794 | 115.62 | 24.4 | 14 | 17 | 14 | 0.97 | 0.7% |
| D4A7L4 | NADH dehydrogenase (Ubiquinone) 1 beta subcomplex, 11 (Predicted) | 17.634 | 7.8172 | 17.9 | 2 | 2 | 2 | 0.97 | 1.4% |
| B2RZB5 | Chromatin modifying protein 2A | 25.134 | 35.374 | 19.8 | 5 | 6 | 5 | 0.97 | 2.9% |
| D4ADG9 | Protein Col8a2 | 66.93 | 8.465 | 2 | 1 | 1 | 1 | 0.97 | 3.2% |
| F1M4I4 | Protein Vps51 | 86.041 | 35.65 | 7.9 | 5 | 5 | 5 | 0.97 | 3.7% |
| M0RB44 | Protein Shroom1 | 92.773 | 12.265 | 2.6 | 2 | 2 | 2 | 0.97 | 6.1% |
| P62268 | 40S ribosomal protein S23 | 15.807 | 29.326 | 29.4 | 5 | 15 | 5 | 0.97 | 2.2% |
| Q9JHZ4 | GRIP1-associated protein 1 | 96.073 | 29.479 | 12.2 | 8 | 8 | 8 | 0.97 | 1.4% |
| Q6TUG0 | DnaJ homolog subfamily B member 11 | 40.495 | 168.76 | 40.8 | 16 | 33 | 16 | 0.97 | 0.7% |
| D3ZR64 | Protein Zfp598 | 98.853 | 12.586 | 5.9 | 4 | 4 | 4 | 0.97 | 1.4% |
| G3V8N0 | Protein Sfxn2 | 16.551 | 14.268 | 9 | 1 | 1 | 1 | 0.97 | 2.2% |
| Q641Y2 | NADH dehydrogenase [ubiquinone] iron-sulfur protein 2, mitochondrial | 52.561 | 56.906 | 29.8 | 11 | 14 | 11 | 0.97 | 2.9% |
| F1LPG5 | Protein LOC688963 | 15.064 | 6.7434 | 17.8 | 2 | 2 | 2 | 0.97 | 3.5% |
| B2GUZ9 | Fam49b protein | 36.776 | 50.498 | 32.7 | 9 | 12 | 7 | 0.97 | 7.5% |
| F1LX07 | Protein Slc25a12 | 71.916 | 69.918 | 20.9 | 11 | 14 | 9 | 0.97 | 6.5% |
| P70550 | Ras-related protein Rab-8B | 23.603 | 20.446 | 33.3 | 7 | 12 | 2 | 0.97 | 12.8% |
| Q6AYD9 | Nucleoside diphosphate-linked moiety X motif 19, mitochondrial | 39.938 | 40.802 | 19.3 | 5 | 7 | 5 | 0.97 | 3.7% |
| F1LWG4 | NADH dehydrogenase (Ubiquinone) 1 alpha subcomplex, assembly factor 1 (Predicted), isoform CRA_a | 37.781 | 14.101 | 12.2 | 4 | 4 | 4 | 0.97 | 2.1% |
| B2GV54 | Neutral cholesterol ester hydrolase 1 | 45.821 | 37.305 | 25 | 9 | 10 | 9 | 0.97 | 1.9% |
| Q9JM53 | Apoptosis-inducing factor 1, mitochondrial | 66.722 | 104.48 | 27.9 | 14 | 26 | 14 | 0.97 | 1.8% |
| D4ADZ1 | Protein Arhgef17 | 222 | 62.412 | 6 | 10 | 10 | 10 | 0.97 | 1.4% |
| P29994 | Inositol 1,4,5-trisphosphate receptor type 1 | 313.26 | 314.77 | 19.1 | 45 | 64 | 35 | 0.97 | 1.3% |
| A0A096MJZ2 | Protein Tbl2 | 49.613 | 37.833 | 18.8 | 8 | 13 | 8 | 0.97 | 1.0% |
| Q9Z0V5 | Peroxiredoxin-4 | 31.007 | 80.731 | 46.9 | 10 | 23 | 9 | 0.97 | 0.4% |
| Q9WVB1 | Ras-related protein Rab-6A | 23.59 | 90.844 | 51.9 | 11 | 30 | 7 | 0.97 | 2.4% |
| Q68FQ7 | RNA polymerase II-associated protein 3 | 74.644 | 9.2963 | 5.5 | 3 | 3 | 3 | 0.97 | 9.7% |
| A0A0G2JWB6 | Protein Pxdn | 165.21 | 14.984 | 4.1 | 5 | 5 | 5 | 0.97 | 4.1% |
| D3ZHF8 | Translation factor Guf1, mitochondrial | 73.206 | 6.4497 | 2.3 | 1 | 1 | 1 | 0.97 | 3.7% |
| A0A0G2JT90 | Protein Ash2l | 68.684 | 2.969 | 1.3 | 1 | 1 | 1 | 0.97 | 19.4% |
| P06762 | Heme oxygenase 1 | 33.005 | 118.66 | 37.7 | 8 | 18 | 8 | 0.97 | 2.1% |
| Q5XIP9 | Transmembrane protein 43 | 44.774 | 124.8 | 36.2 | 12 | 22 | 12 | 0.97 | 1.3% |
| O35547 | Long-chain-fatty-acid--CoA ligase 4 | 74.326 | 66.59 | 15.1 | 7 | 11 | 7 | 0.97 | 0.8% |
| Q9QUL6 | Vesicle-fusing ATPase | 82.652 | 165.76 | 31.6 | 24 | 39 | 24 | 0.97 | 0.8% |
| P48450 | Lanosterol synthase | 83.3 | 27.059 | 10.1 | 7 | 10 | 7 | 0.97 | 0.2% |
| D3ZLC3 | Protein Ssfa2 | 137.19 | 27.391 | 3.8 | 4 | 4 | 4 | 0.97 | 2.1% |
| P21571 | ATP synthase-coupling factor 6, mitochondrial | 12.494 | 30.853 | 29.6 | 2 | 3 | 2 | 0.97 | 2.1% |
| D3ZTL0 | Protein Tcerg1 | 121.85 | 72.02 | 12.5 | 14 | 15 | 14 | 0.97 | 5.4% |
| P52555 | Endoplasmic reticulum resident protein 29 | 28.574 | 60.787 | 47.3 | 10 | 20 | 10 | 0.97 | 3.4% |
| Q6AXT8 | Splicing factor 3A subunit 2 | 49.89 | 32.51 | 9.1 | 4 | 4 | 4 | 0.97 | 0.8% |
| B2GV41 | Protein Usp39 | 65.244 | 11.954 | 4.3 | 2 | 2 | 2 | 0.97 | 3.5% |
| M0RAP5 | Protein Sbf1 | 211.4 | 43.298 | 5.7 | 9 | 9 | 8 | 0.97 | 4.2% |
| A1L1J9 | Lipase maturation factor 2 | 80.253 | 2.1873 | 1.1 | 1 | 1 | 1 | 0.97 | 13.4% |
| Q4QQS6 | Asparagine-linked glycosylation 5 homolog (Yeast, dolichyl-phosphate beta-glucosyltransferase), isoform CRA_a | 36.856 | 86.965 | 31.5 | 7 | 10 | 7 | 0.97 | 4.8% |
| A0A0G2K6Z8 | Protein Plekha7 | 135.65 | 25.703 | 8.7 | 11 | 11 | 11 | 0.97 | 3.5% |
| Q09073 | ADP/ATP translocase 2 | 32.901 | 197.14 | 58.1 | 20 | 55 | 9 | 0.97 | 2.5% |
| Q91V33 | KH domain-containing, RNA-binding, signal transduction-associated protein 1 | 48.315 | 7.7213 | 7.2 | 3 | 3 | 3 | 0.97 | 1.6% |
| P09527 | Ras-related protein Rab-7a | 23.504 | 141.06 | 81.6 | 16 | 41 | 16 | 0.97 | 1.0% |
| B5DFB0 | Leprecan-like 2 (Predicted), isoform CRA_b | 62.206 | 86.552 | 21.5 | 10 | 13 | 10 | 0.97 | 0.9% |
| Q64678 | Cytochrome P450 1B1 | 60.556 | 56.654 | 15.3 | 8 | 11 | 8 | 0.97 | 0.4% |
| D3ZF54 | Anoctamin | 79.039 | 82.103 | 17.6 | 10 | 15 | 10 | 0.97 | 0.9% |
| P28075 | Proteasome subunit beta type-5 | 28.585 | 57.649 | 41.1 | 9 | 9 | 9 | 0.97 | 1.2% |
| Q4V7D9 | Protein Smpdl3b | 51.645 | 7.0065 | 4.8 | 2 | 3 | 2 | 0.97 | 2.7% |
| G3V991 | Integrin, alpha 6, isoform CRA_b | 121.81 | 1.8733 | 1.4 | 2 | 2 | 1 | 0.97 | 5.3% |
| P18886 | Carnitine O-palmitoyltransferase 2, mitochondrial | 74.109 | 85.242 | 20.5 | 11 | 14 | 11 | 0.97 | 5.7% |
| P97519 | Hydroxymethylglutaryl-CoA lyase, mitochondrial | 34.191 | 42.104 | 18.2 | 4 | 5 | 4 | 0.97 | 4.0% |
| F1LN42 | Protein Tns1 | 203.91 | 256.69 | 22.5 | 35 | 57 | 32 | 0.97 | 3.5% |
| D3ZQL1 | Protein Emc7 | 26.44 | 24.248 | 16.2 | 3 | 4 | 3 | 0.97 | 4.5% |
| Q62929 | Interleukin-1 receptor-like 2 | 64.141 | 3.9101 | 2.9 | 2 | 2 | 2 | 0.97 | 4.7% |
| Q5I0H9 | Protein disulfide-isomerase A5 | 59.399 | 20.354 | 11 | 6 | 7 | 6 | 0.97 | 2.3% |
| O88764 | Death-associated protein kinase 3 | 51.449 | 20.636 | 13.6 | 6 | 8 | 6 | 0.97 | 1.7% |
| D3ZTT7 | Protein Sun2 | 81.526 | 143.48 | 26.8 | 16 | 21 | 16 | 0.97 | 1.1% |
| D3ZGF2 | Protein LOC501033 | 101.63 | 2.2533 | 2.1 | 2 | 2 | 2 | 0.97 | 1.0% |
| P15684 | Aminopeptidase N | 109.45 | 64.486 | 9.6 | 8 | 16 | 8 | 0.97 | 0.6% |
| F1M3H8 | Protein Hnrnpa0 | 30.076 | 121.31 | 45.2 | 12 | 24 | 12 | 0.96 | 3.6% |
| Q5PQP1 | RNA-binding motif, single-stranded-interacting protein 1 | 44.067 | 25.406 | 11.7 | 4 | 11 | 1 | 0.96 | 2.9% |
| P80201 | Activin receptor type-1 | 57.194 | 5.1952 | 2.2 | 1 | 1 | 1 | 0.96 | 2.5% |
| D3ZME7 | Protein Hscb | 27.065 | 9.6476 | 17.5 | 4 | 4 | 4 | 0.96 | 2.2% |
| P62997 | Transformer-2 protein homolog beta | 33.665 | 51.864 | 27.8 | 6 | 12 | 5 | 0.96 | 0.8% |
| Q5U2V1 | FK506 binding protein 10 | 64.786 | 138.59 | 32.9 | 17 | 41 | 16 | 0.96 | 0.2% |
| D4A5K7 | Protein Rbm28 | 84.372 | 7.236 | 2.8 | 2 | 2 | 2 | 0.96 | 0.9% |
| G3V829 | Protein Fubp3 | 61.436 | 73.124 | 28.3 | 15 | 18 | 15 | 0.96 | 1.1% |
| P22509 | rRNA 2-O-methyltransferase fibrillarin | 34.221 | 79.794 | 39.8 | 11 | 16 | 11 | 0.96 | 1.2% |
| P82471 | Guanine nucleotide-binding protein G(q) subunit alpha | 42.144 | 63.185 | 35.7 | 11 | 14 | 9 | 0.96 | 1.8% |
| Q5BJP4 | Protein LOC100910882 | 58.683 | 64.492 | 15.6 | 7 | 9 | 7 | 0.96 | 2.1% |
| P62718 | 60S ribosomal protein L18a | 20.732 | 47.305 | 49.4 | 10 | 25 | 10 | 0.96 | 2.2% |
| B1WC12 | Protein Mfsd5 | 49.683 | 15.564 | 3.3 | 1 | 2 | 1 | 0.96 | 2.4% |
| B2RYF6 | Cleft lip and palate associated transmembrane protein 1 | 75.276 | 27.443 | 9.5 | 5 | 5 | 5 | 0.96 | 5.4% |
| O35165 | Golgi SNAP receptor complex member 2 | 24.607 | 38.283 | 25 | 5 | 15 | 5 | 0.96 | 5.4% |
| F1M9W9 | Protein Trappc8 | 160.35 | 24.458 | 3.7 | 5 | 6 | 5 | 0.96 | 6.3% |
| P12001 | 60S ribosomal protein L18 | 21.658 | 40.216 | 24.5 | 4 | 10 | 4 | 0.96 | 3.5% |
| Q6MG08 | ATP-binding cassette sub-family F member 1 | 95.251 | 123.74 | 16.8 | 10 | 14 | 10 | 0.96 | 3.1% |
| G3V6H0 | Ras-related protein Rab-1B | 22.177 | 46.49 | 58.7 | 11 | 28 | 4 | 0.96 | 2.7% |
| P63326 | 40S ribosomal protein S10 | 18.916 | 41.456 | 53.9 | 12 | 31 | 11 | 0.96 | 2.1% |
| P10888 | Cytochrome c oxidase subunit 4 isoform 1, mitochondrial | 19.514 | 37.019 | 41.4 | 10 | 21 | 10 | 0.96 | 1.5% |
| Q63584 | Transmembrane emp24 domain-containing protein 10 | 24.857 | 131.12 | 43.8 | 12 | 33 | 12 | 0.96 | 1.6% |
| D4A1Q9 | Protein Ttll12 | 73.897 | 55.131 | 16.1 | 8 | 8 | 8 | 0.96 | 6.4% |
| M0R907 | Protein Snrpd3 | 13.916 | 73.486 | 31.7 | 3 | 10 | 3 | 0.96 | 3.6% |
| A0FKI7 | Acyl-CoA-binding domain-containing protein 5 | 56.781 | 24.089 | 7.7 | 5 | 7 | 5 | 0.96 | 2.9% |
| P04762 | Catalase | 59.756 | 156.11 | 38.1 | 18 | 29 | 18 | 0.96 | 1.9% |
| D3ZRM5 | Protein Rab23 | 26.633 | 121.92 | 35.9 | 6 | 16 | 6 | 0.96 | 1.6% |
| Q4KLX9 | Protein Ccdc163 | 39.505 | -2 | 3.5 | 1 | 3 | 1 | 0.96 | 1.6% |
| D4A0T0 | Protein Ndufb10 | 20.858 | 71.231 | 64.2 | 10 | 15 | 10 | 0.96 | 2.3% |
| Q5M7V8 | Thyroid hormone receptor-associated protein 3 | 108.25 | 51.871 | 13.9 | 10 | 11 | 10 | 0.96 | 2.4% |
| D4A030 | Protein Ubp1 | 60.162 | 9.5098 | 6.7 | 3 | 3 | 2 | 0.96 | 2.5% |
| Q10473 | Polypeptide N-acetylgalactosaminyltransferase 1 | 64.228 | 26.579 | 13.4 | 7 | 10 | 7 | 0.96 | 2.9% |
| Q6QMY6 | Tsukushin | 38.09 | 6.8966 | 4.2 | 1 | 1 | 1 | 0.96 | 5.7% |
| D4A9Y0 | Protein Sdf2l1 | 23.403 | 51.9 | 23.6 | 4 | 9 | 4 | 0.96 | 8.8% |
| F1LXV3 | Protein Stk26 | 46.573 | 31.623 | 15.9 | 5 | 7 | 3 | 0.96 | 6.3% |
| D3ZAF6 | ATP synthase subunit f, mitochondrial | 10.452 | 14.478 | 23.9 | 2 | 4 | 2 | 0.96 | 2.7% |
| D3ZJ32 | Protein Esyt2 | 93.95 | 269.4 | 31.5 | 20 | 40 | 20 | 0.96 | 4.3% |
| P04905 | Glutathione S-transferase Mu 1 | 25.914 | 27.926 | 41.3 | 9 | 14 | 7 | 0.96 | 5.5% |
| D4A994 | Protein Emc1 | 111.18 | 90.723 | 9.9 | 9 | 14 | 9 | 0.96 | 1.7% |
| Q5XHZ8 | Component of oligomeric golgi complex 3 | 94.246 | 48.749 | 10.6 | 7 | 12 | 7 | 0.96 | 1.3% |
| M0R3V4 | Uncharacterized protein | 17.952 | 19.196 | 21.1 | 3 | 8 | 3 | 0.96 | 0.7% |
| P18420 | Proteasome subunit alpha type-1 | 29.517 | 91.865 | 46 | 14 | 27 | 14 | 0.96 | 0.4% |
| Q4KLK7 | Nucleolar protein 5A | 65.399 | 87.293 | 24.8 | 12 | 18 | 12 | 0.96 | 0.5% |
| P07687 | Epoxide hydrolase 1 | 52.581 | 44.553 | 20.4 | 10 | 17 | 10 | 0.96 | 1.8% |
| B0BNM4 | Protein RGD1308134 | 17.954 | 13.016 | 17.5 | 2 | 3 | 2 | 0.96 | 2.3% |
| O55035 | Peptidyl-prolyl cis-trans isomerase G | 88.378 | 19.72 | 4.8 | 3 | 4 | 3 | 0.96 | 5.4% |
| P61621 | Protein transport protein Sec61 subunit alpha isoform 1 | 52.264 | 39.768 | 15.3 | 7 | 8 | 7 | 0.96 | 4.3% |
| Q4FZX7 | Signal recognition particle receptor subunit beta | 29.569 | 111.33 | 31.2 | 9 | 13 | 9 | 0.96 | 3.5% |
| Q6EV70 | GDP-fucose protein O-fucosyltransferase 1 | 44.982 | 31.048 | 17.5 | 5 | 8 | 5 | 0.96 | 4.1% |
| P32551 | Cytochrome b-c1 complex subunit 2, mitochondrial | 48.396 | 114.16 | 37.8 | 14 | 25 | 14 | 0.96 | 4.6% |
| P62193 | 26S protease regulatory subunit 4 | 49.184 | 205.13 | 50.5 | 21 | 42 | 21 | 0.96 | 2.3% |
| Q4KLP0 | Probable 2-oxoglutarate dehydrogenase E1 component DHKTD1, mitochondrial | 102.64 | 35.632 | 5.3 | 3 | 4 | 3 | 0.96 | 2.0% |
| B0BN89 | Dihydrouridine synthase 2-like, SMM1 homolog (S. cerevisiae) | 55.41 | 2.0639 | 1.4 | 1 | 1 | 1 | 0.96 | 1.8% |
| D4A2G6 | Protein Thbs2 | 129.73 | 150.95 | 19 | 19 | 28 | 18 | 0.96 | 1.8% |
| Q9WVJ4 | Synaptojanin-2-binding protein | 15.825 | 31.093 | 24.1 | 3 | 5 | 3 | 0.96 | 1.8% |
| B1WC61 | Acad9 protein | 68.842 | 74.409 | 19.4 | 11 | 16 | 11 | 0.96 | 1.4% |
| D4A0H1 | Receptor expression-enhancing protein | 29.157 | 5.2997 | 3.9 | 1 | 1 | 1 | 0.96 | 0.6% |
| O08722 | Netrin receptor UNC5B | 103.52 | 106.92 | 9.2 | 8 | 10 | 8 | 0.96 | 4.8% |
| Q62611 | Interleukin-1 receptor-like 1 | 64.405 | 6.5012 | 5.7 | 3 | 3 | 3 | 0.96 | 7.4% |
| D3ZD04 | Dystrobrevin | 77.583 | 21.032 | 6.7 | 4 | 5 | 4 | 0.96 | 5.4% |
| D3ZG78 | Protein Zzef1 | 332.37 | 35.739 | 2.1 | 5 | 6 | 5 | 0.96 | 4.7% |
| Q01205 | Dihydrolipoyllysine-residue succinyltransferase component of 2-oxoglutarate dehydrogenase complex, mitochondrial | 48.925 | 106.05 | 36.8 | 13 | 23 | 13 | 0.96 | 2.8% |
| Q5XIA1 | Nicalin | 62.992 | 80.515 | 16.9 | 10 | 14 | 10 | 0.96 | 2.8% |
| P67874 | Casein kinase II subunit beta | 24.942 | 31.075 | 30.7 | 5 | 7 | 5 | 0.96 | 7.7% |
| P61765 | Syntaxin-binding protein 1 | 67.568 | 87.573 | 25.4 | 12 | 15 | 11 | 0.96 | 3.9% |
| G3V7T6 | Protein Sf3b1 | 145.83 | 126.46 | 19.8 | 19 | 23 | 19 | 0.96 | 1.9% |
| P97852 | Peroxisomal multifunctional enzyme type 2 | 79.427 | 136.13 | 31.2 | 19 | 27 | 19 | 0.96 | 1.0% |
| Q5U1X1 | Oligoribonuclease, mitochondrial | 26.751 | 73.486 | 42.6 | 8 | 19 | 8 | 0.96 | 0.4% |
| P0C5H9 | Mesencephalic astrocyte-derived neurotrophic factor | 20.388 | 100.89 | 45.3 | 13 | 23 | 13 | 0.96 | 1.1% |
| D3ZFB2 | Protein Luc7l3 | 58.423 | 24.246 | 10.2 | 5 | 6 | 5 | 0.96 | 1.3% |
| B5DF27 | Lysyl oxidase homolog 2 | 87.184 | 64.945 | 9.9 | 6 | 6 | 6 | 0.96 | 2.7% |
| Q5XI81 | Fragile X mental retardation syndrome-related protein 1 | 63.947 | 68.589 | 21.5 | 11 | 15 | 8 | 0.96 | 3.1% |
| O70595 | ATP-binding cassette sub-family B member 6, mitochondrial | 93.304 | 49.108 | 7.8 | 4 | 4 | 4 | 0.96 | 0.6% |
| A0A0G2K7B6 | Protein Dysf | 242.46 | 323.31 | 34.6 | 57 | 89 | 56 | 0.96 | 0.4% |
| D4ACN6 | Procollagen, type IV, alpha 3 (Goodpasture antigen) binding protein (Predicted), isoform CRA_a | 71.068 | 15.111 | 5.6 | 3 | 3 | 3 | 0.96 | 0.4% |
| M0RDK9 | Protein Acad8 | 45.155 | 1.9456 | 1.9 | 1 | 1 | 1 | 0.96 | 0.6% |
| Q6PEC3 | Protein YIF1B | 28.418 | 24.356 | 15.1 | 2 | 2 | 2 | 0.96 | 1.6% |
| P13233 | 2,3-cyclic-nucleotide 3-phosphodiesterase | 47.268 | 36.151 | 21.7 | 8 | 9 | 8 | 0.96 | 1.9% |
| A0A0G2K5E4 | Protein Dnaja3 | 52.403 | 52.153 | 9.4 | 4 | 5 | 4 | 0.96 | 7.1% |
| O35821 | Myb-binding protein 1A | 152.28 | 267.52 | 24.4 | 31 | 44 | 31 | 0.96 | 3.6% |
| Q9QX81 | Protein Hnrnpab | 36.232 | 110.23 | 31 | 12 | 32 | 11 | 0.96 | 2.6% |
| E9PTR4 | Protein Ubap2l | 116.86 | 158.52 | 14.7 | 11 | 21 | 11 | 0.96 | 1.0% |
| P11884 | Aldehyde dehydrogenase, mitochondrial | 56.488 | 217.32 | 46.8 | 18 | 46 | 18 | 0.96 | 0.4% |
| D3ZUL1 | Protein Ccdc124 | 25.317 | 26.72 | 31.8 | 6 | 6 | 6 | 0.96 | 1.4% |
| Q68FV6 | Lysosomal protein NCU-G1 | 43.871 | 54.197 | 10.6 | 2 | 3 | 2 | 0.96 | 10.7% |
| Q5RJS6 | Motile sperm domain-containing protein 1 | 24.088 | 11.389 | 11.7 | 2 | 2 | 2 | 0.96 | 5.3% |
| Q6MG12 | Uncharacterized protein C6orf136 homolog | 20.445 | 7.6215 | 7 | 1 | 1 | 1 | 0.96 | 2.8% |
| P41740 | Atrial natriuretic peptide receptor 3 | 59.727 | 50.706 | 19.1 | 9 | 14 | 9 | 0.96 | 1.6% |
| Q5RJT2 | pre-rRNA processing protein FTSJ3 | 94.766 | 11.011 | 6.3 | 4 | 4 | 4 | 0.96 | 1.4% |
| P07151 | Beta-2-microglobulin | 13.72 | 23.518 | 26.1 | 4 | 8 | 4 | 0.96 | 1.3% |
| P50430 | Arylsulfatase B | 58.958 | 30.342 | 14 | 7 | 10 | 7 | 0.96 | 1.3% |
| Q6P4Z8 | Dolichyl-phosphate (UDP-N-acetylglucosamine) N-acetylglucosaminephosphotransferase 1 (GlcNAc-1-P transferase) | 46.461 | 2.3888 | 2 | 1 | 1 | 1 | 0.96 | 1.1% |
| A0A0G2JVM2 | Protein Mia3 | 153.47 | 131.2 | 13.9 | 18 | 27 | 18 | 0.96 | 0.3% |
| P45479 | Palmitoyl-protein thioesterase 1 | 34.455 | 50.88 | 25.2 | 7 | 12 | 7 | 0.96 | 0.1% |
| D3ZDI7 | Protein Ppp2r5a | 56.336 | 14.463 | 6.4 | 2 | 2 | 2 | 0.96 | 0.3% |
| D4A962 | Heterogeneous nuclear ribonucleoprotein U-like 1 (Predicted) | 96.001 | 65.7 | 13.2 | 10 | 12 | 10 | 0.96 | 0.4% |
| F1LN92 | Protein Afg3l2 | 89.35 | 98.211 | 22.9 | 17 | 19 | 16 | 0.96 | 0.8% |
| Q9JMJ4 | Pre-mRNA-processing factor 19 | 55.238 | 77.2 | 25.8 | 11 | 15 | 11 | 0.96 | 1.3% |
| Q68FY0 | Cytochrome b-c1 complex subunit 1, mitochondrial | 52.848 | 72.268 | 25.4 | 10 | 18 | 9 | 0.96 | 2.2% |
| D4ADL7 | Protein Xxylt1 | 43.912 | 7.114 | 5.9 | 2 | 2 | 2 | 0.96 | 5.0% |
| B0BNL2 | Peptidylprolyl cis/trans isomerase, NIMA-interacting 1 | 18.332 | 61.582 | 22.4 | 3 | 7 | 3 | 0.96 | 3.2% |
| D3ZL83 | Protein Fhdc1 | 125.57 | 2.5161 | 1.2 | 2 | 3 | 2 | 0.96 | 3.1% |
| F1M8B8 | Protein RGD1306556 | 34.649 | 32.33 | 19.1 | 5 | 12 | 5 | 0.96 | 1.8% |
| Q9WUL0 | DNA topoisomerase 1 | 90.759 | 93.314 | 22.9 | 16 | 23 | 16 | 0.96 | 0.8% |
| Q0ZFS7 | 1-acylglycerol-3-phosphate O-acyltransferase 6 (Lysophosphatidic acid acyltransferase, zeta) | 52.123 | 12.863 | 5 | 2 | 2 | 2 | 0.96 | 0.2% |
| P17164 | Tissue alpha-L-fucosidase | 53.486 | 130.23 | 28.8 | 11 | 26 | 11 | 0.96 | 0.1% |
| Q5XIA6 | Sphingomyelin phosphodiesterase | 69.76 | 15.378 | 7 | 4 | 5 | 4 | 0.96 | 0.3% |
| Q9WUD2 | Transient receptor potential cation channel subfamily V member 2 | 86.705 | 98.794 | 19.7 | 12 | 14 | 12 | 0.96 | 1.4% |
| B2RYW3 | NADH dehydrogenase (Ubiquinone) 1 beta subcomplex, 9 | 21.892 | 27.323 | 23.5 | 5 | 6 | 5 | 0.96 | 1.6% |
| Q1H5H1 | Selenoprotein T | 22.292 | 9.3207 | 11.8 | 2 | 2 | 2 | 0.96 | 1.6% |
| A0A0G2K8K0 | Protein Sfpq | 75.085 | 123.5 | 29 | 22 | 34 | 21 | 0.96 | 2.7% |
| Q5PQQ7 | LIX1-like protein | 36.662 | 3.9721 | 5.3 | 2 | 2 | 2 | 0.96 | 3.0% |
| D3ZVH6 | Protein Vps41 | 89.581 | 16.564 | 5.3 | 3 | 3 | 3 | 0.96 | 4.2% |
| A0A0G2K261 | Protein Iars2 | 112.68 | 160.67 | 22.5 | 18 | 29 | 18 | 0.96 | 5.5% |
| Q6AYD2 | CST complex subunit STN1 | 46.782 | 21.182 | 12 | 4 | 4 | 3 | 0.96 | 0.1% |
| A0A0G2K8W9 | Protein Sptbn1 | 273.47 | 323.31 | 44 | 95 | 193 | 88 | 0.96 | 0.3% |
| D4A720 | Protein Srsf7 | 27.377 | 34.116 | 33.6 | 9 | 17 | 8 | 0.96 | 1.4% |
| P21263 | Nestin | 208.79 | 88.531 | 10.6 | 15 | 18 | 15 | 0.96 | 1.7% |
| P70566 | Tropomodulin-2 | 39.491 | 5.0851 | 6.3 | 2 | 3 | 1 | 0.96 | 6.9% |
| D4A6C5 | Protein Arhgap1 | 50.622 | 58.891 | 30.5 | 13 | 18 | 13 | 0.96 | 3.7% |
| P58775 | Tropomyosin beta chain | 32.836 | 323.31 | 73.6 | 46 | 255 | 5 | 0.96 | 3.5% |
| Q62636 | Ras-related protein Rap-1b | 20.798 | 164.79 | 69 | 14 | 44 | 7 | 0.96 | 2.9% |
| Q60587 | Trifunctional enzyme subunit beta, mitochondrial | 51.414 | 110.44 | 41.5 | 16 | 33 | 16 | 0.96 | 1.1% |
| Q32KJ5 | Glucosamine (N-acetyl)-6-sulfatase | 60.913 | 56.228 | 17.6 | 10 | 14 | 10 | 0.96 | 0.7% |
| F1LR10 | Epithelial protein lost in neoplasm | 83.796 | 320.09 | 47.2 | 30 | 69 | 30 | 0.96 | 0.4% |
| D4AEC2 | Protein Camsap2 | 165.61 | 2.5197 | 0.7 | 1 | 1 | 1 | 0.96 | 1.5% |
| Q9Z1K9 | Disintegrin and metalloproteinase domain-containing protein 17 | 93.016 | 32.702 | 4 | 3 | 3 | 3 | 0.96 | 1.5% |
| M0R6L8 | Protein Dnajc19 | 19.364 | 14.635 | 21.3 | 4 | 5 | 4 | 0.96 | 1.7% |
| Q63772 | Growth arrest-specific protein 6 | 74.637 | 23.338 | 13.8 | 8 | 9 | 8 | 0.96 | 0.8% |
| Q9Z2L0 | Voltage-dependent anion-selective channel protein 1 | 30.755 | 265.07 | 67.5 | 16 | 36 | 16 | 0.96 | 0.6% |
| P15999 | ATP synthase subunit alpha, mitochondrial | 59.753 | 315.22 | 54.2 | 29 | 92 | 10 | 0.96 | 0.5% |
| P48679 | Prelamin-A/C | 74.323 | 323.31 | 72.5 | 58 | 159 | 57 | 0.96 | 0.4% |
| D3ZC96 | Tetratricopeptide repeat protein 39B | 70.167 | 41.404 | 14.3 | 5 | 6 | 5 | 0.96 | 0.5% |
| P29410 | Adenylate kinase 2, mitochondrial | 26.379 | 56.282 | 38.5 | 9 | 25 | 9 | 0.96 | 0.9% |
| A0JN29 | Limb and neural patterns | 42.169 | 47.467 | 16.5 | 7 | 10 | 7 | 0.96 | 1.3% |
| F1LQJ7 | Protein Pck2 | 71.206 | 206.11 | 40.9 | 21 | 45 | 21 | 0.96 | 3.0% |
| Q6UPE1 | Electron transfer flavoprotein-ubiquinone oxidoreductase, mitochondrial | 68.197 | 83.429 | 29.4 | 17 | 24 | 17 | 0.96 | 4.6% |
| B2GV63 | Carbohydrate (N-acetylgalactosamine 4-0) sulfotransferase 14 | 43.074 | 16.367 | 12.8 | 4 | 4 | 4 | 0.96 | 5.3% |
| P0C2X9 | Delta-1-pyrroline-5-carboxylate dehydrogenase, mitochondrial | 61.868 | 24.309 | 9.4 | 5 | 6 | 5 | 0.96 | 7.8% |
| B0BN56 | 28S ribosomal protein S31, mitochondrial | 43.961 | 31.046 | 20.2 | 7 | 8 | 7 | 0.96 | 4.6% |
| Q6QGW5 | Steroid receptor RNA activator 1 | 25.263 | 32.897 | 25.1 | 4 | 6 | 4 | 0.96 | 3.6% |
| A0A096MIX2 | DEAD (Asp-Glu-Ala-Asp) box polypeptide 17, isoform CRA_a | 72.644 | 64.291 | 32.2 | 22 | 34 | 14 | 0.96 | 2.9% |
| Q66HL2 | Src substrate cortactin | 56.941 | 93.269 | 34.2 | 19 | 38 | 19 | 0.96 | 1.9% |
| D3Z9U7 | Protein Zc3h4 | 141.09 | 2.5669 | 2.1 | 2 | 2 | 2 | 0.96 | 0.3% |
| D3ZZW1 | Protein Dock1 | 214.96 | 26.604 | 4.2 | 6 | 7 | 5 | 0.96 | 2.0% |
| P11598 | Protein disulfide-isomerase A3 | 56.623 | 323.31 | 66.9 | 42 | 124 | 42 | 0.96 | 2.3% |
| D3ZNP2 | Protein Marveld1 | 19.059 | 4.065 | 8.1 | 2 | 5 | 2 | 0.96 | 3.0% |
| A0A0G2K9T1 | Protein Itch | 100.83 | 25.689 | 2.8 | 2 | 2 | 1 | 0.96 | 12.5% |
| Q2TA68 | Dynamin-like 120 kDa protein, mitochondrial | 111.31 | 172.63 | 29.1 | 23 | 30 | 23 | 0.96 | 5.5% |
| Q62745 | CD81 antigen | 25.888 | 41.992 | 15.3 | 2 | 15 | 2 | 0.96 | 3.4% |
| Q5XIL4 | Protein Sorbs3 | 82.095 | 36.589 | 9.4 | 5 | 6 | 5 | 0.96 | 2.8% |
| F1MA89 | Protein Ccny | 39.367 | 24.182 | 7.6 | 2 | 2 | 2 | 0.96 | 2.3% |
| P28042 | Single-stranded DNA-binding protein, mitochondrial | 17.455 | 62.164 | 45.7 | 6 | 14 | 6 | 0.96 | 3.1% |
| D3ZWE0 | Histone H2A | 13.999 | 2.7082 | 17.7 | 3 | 4 | 1 | 0.96 | 8.3% |
| Q4QQU9 | Osteopetrosis associated transmembrane protein 1 | 37.909 | 26.938 | 14.5 | 4 | 5 | 4 | 0.96 | 0.5% |
| Q6AYS8 | Estradiol 17-beta-dehydrogenase 11 | 32.937 | 109.47 | 44.6 | 12 | 21 | 12 | 0.96 | 0.4% |
| Q2KMM2 | Trafficking protein particle complex subunit 1 | 16.881 | 3.5772 | 6.9 | 1 | 1 | 1 | 0.96 | 1.1% |
| D4A779 | Protein Phldb2 | 147.52 | 91.335 | 13.5 | 19 | 24 | 19 | 0.96 | 2.0% |
| O54924 | Exocyst complex component 8 | 81.042 | 77.535 | 18.6 | 11 | 15 | 11 | 0.96 | 3.0% |
| Q9JJK4 | Peroxisomal biogenesis factor 3 | 42.209 | 29.696 | 6.5 | 2 | 2 | 2 | 0.96 | 3.1% |
| Q08602 | Geranylgeranyl transferase type-2 subunit alpha | 64.904 | 22.235 | 12.9 | 7 | 8 | 7 | 0.96 | 5.4% |
| Q6UPR8 | Endoplasmic reticulum metallopeptidase 1 | 99.896 | 7.0018 | 3.6 | 3 | 4 | 3 | 0.96 | 5.5% |
| F1LM55 | Protein Ccar2 | 102.79 | 53.743 | 13.6 | 13 | 17 | 13 | 0.96 | 1.1% |
| F1LQH9 | Protein Bag2 | 23.805 | 92.631 | 42.9 | 10 | 19 | 10 | 0.96 | 0.7% |
| Q498C8 | Protein RER1 | 22.988 | 12.735 | 18.9 | 4 | 6 | 4 | 0.96 | 0.5% |
| B2GUZ3 | Mthfd1l protein | 105.84 | 126.72 | 21.8 | 20 | 27 | 20 | 0.96 | 0.2% |
| Q9ESR9 | ATP-binding cassette sub-family A member 2 | 270.92 | 2.2111 | 0.7 | 2 | 2 | 2 | 0.96 | 0.7% |
| A2RUW1 | Toll-interacting protein | 30.314 | 61.855 | 41.2 | 10 | 14 | 10 | 0.96 | 1.1% |
| D3ZFK8 | FERM, RhoGEF and pleckstrin domain protein 2 (Predicted) | 120.8 | 42.732 | 7.7 | 7 | 8 | 7 | 0.96 | 3.5% |
| P04785 | Protein disulfide-isomerase | 56.951 | 250.15 | 55.8 | 34 | 102 | 34 | 0.96 | 0.5% |
| F1LQ22 | Protein Use1 | 30.513 | 39.997 | 30.4 | 7 | 7 | 7 | 0.96 | 0.0% |
| Q5I0D5 | Phospholysine phosphohistidine inorganic pyrophosphate phosphatase | 29.19 | 57.505 | 27 | 4 | 8 | 4 | 0.96 | 0.3% |
| B1WBY5 | DnaJ (Hsp40) homolog, subfamily C, member 11 | 63.204 | 51.139 | 16.8 | 8 | 11 | 8 | 0.96 | 1.2% |
| Q4KLI4 | Peptidyl-prolyl cis-trans isomerase | 18.251 | 12.471 | 21.7 | 3 | 4 | 3 | 0.96 | 1.4% |
| Q7TPB4 | CD276 antigen | 34.074 | 4.9789 | 3.2 | 1 | 3 | 1 | 0.96 | 1.7% |
| Q5EB81 | NADH-cytochrome b5 reductase 1 | 34.222 | 84.744 | 45.2 | 10 | 19 | 10 | 0.96 | 1.9% |
| P49793 | Nuclear pore complex protein Nup98-Nup96 | 197.28 | 41.959 | 4.2 | 7 | 10 | 7 | 0.96 | 2.9% |
| D4A2C6 | LSM4 homolog, U6 small nuclear RNA associated (S. cerevisiae) (Predicted), isoform CRA_a | 15.204 | 9.0618 | 15.9 | 2 | 4 | 2 | 0.96 | 3.2% |
| A0A0G2JZ48 | Protein Tmf1 | 121.9 | 33.789 | 9.1 | 9 | 10 | 9 | 0.96 | 2.8% |
| P04692 | Tropomyosin alpha-1 chain | 32.68 | 233.04 | 79.9 | 43 | 186 | 25 | 0.96 | 2.0% |
| Q4V8I7 | Volume-regulated anion channel subunit LRRC8A | 94.161 | 27.555 | 5.8 | 4 | 6 | 3 | 0.96 | 3.0% |
| Q8K3E7 | Protein dpy-30 homolog | 11.2 | 5.3776 | 22.2 | 3 | 3 | 3 | 0.96 | 3.0% |
| F1M387 | Protein B3galtl | 53.831 | 16.62 | 9.7 | 5 | 7 | 5 | 0.96 | 4.8% |
| D3ZIJ5 | Protein Dpm3 | 10.091 | 5.0974 | 13 | 1 | 1 | 1 | 0.96 | 5.5% |
| P60192 | SNARE-associated protein Snapin | 14.904 | 23.072 | 26.5 | 3 | 6 | 3 | 0.96 | 6.4% |
| P70583 | Deoxyuridine 5-triphosphate nucleotidohydrolase | 22.003 | 12.455 | 22 | 3 | 4 | 3 | 0.96 | 6.5% |
| F1M062 | Protein Larp1 | 106.94 | 52.79 | 10.4 | 10 | 10 | 10 | 0.96 | 1.9% |
| A0A0G2JXN8 | Protein Osbpl8 | 101.16 | 64.848 | 9.3 | 6 | 6 | 6 | 0.96 | 2.1% |
| P08753 | Guanine nucleotide-binding protein G(k) subunit alpha | 40.522 | 74.699 | 41 | 13 | 26 | 6 | 0.96 | 2.1% |
| P58405 | Striatin-3 | 87.11 | 37.452 | 10.3 | 7 | 8 | 5 | 0.96 | 2.8% |
| Q6AXW2 | Protein Tmod3 | 39.471 | 111.87 | 42 | 13 | 27 | 12 | 0.96 | 5.4% |
| A0A0G2JYR1 | Protein Exoc6b | 94.189 | 79.256 | 13.2 | 11 | 14 | 11 | 0.96 | 4.8% |
| D3ZE15 | Protein LOC100911483 | 16.776 | 15.81 | 30.6 | 4 | 13 | 4 | 0.96 | 1.5% |
| P70496 | Phospholipase D1 | 123.81 | 63.586 | 8.8 | 7 | 7 | 7 | 0.96 | 1.3% |
| D3ZKG1 | Protein Mut | 92.035 | 97.484 | 14.4 | 10 | 17 | 10 | 0.96 | 0.9% |
| A0A0G2JW60 | Protein Utrn | 392.07 | 323.31 | 24.4 | 69 | 88 | 69 | 0.96 | 0.7% |
| D3ZY44 | Protein Mrps2 | 32.175 | 52.705 | 26.1 | 5 | 7 | 5 | 0.96 | 0.7% |
| Q64428 | Trifunctional enzyme subunit alpha, mitochondrial | 82.664 | 323.31 | 49 | 30 | 81 | 30 | 0.96 | 1.0% |
| P16036 | Phosphate carrier protein, mitochondrial | 39.445 | 85.628 | 36.5 | 14 | 34 | 14 | 0.96 | 1.3% |
| Q5XFV6 | Ribosomal protein L34 | 10.446 | 2.4842 | 11 | 1 | 2 | 1 | 0.95 | 2.8% |
| P04256 | Heterogeneous nuclear ribonucleoprotein A1 | 34.212 | 263.76 | 39.1 | 13 | 44 | 13 | 0.95 | 2.1% |
| A0A096MK61 | Protein Crtap | 46.49 | 86.717 | 33.3 | 13 | 23 | 13 | 0.95 | 5.0% |
| P70500 | CDP-diacylglycerol--inositol 3-phosphatidyltransferase | 23.613 | 7.8104 | 15 | 3 | 3 | 3 | 0.95 | 5.6% |
| D3ZWB4 | Protein Spata13 | 137.76 | 17.917 | 3.9 | 4 | 6 | 4 | 0.95 | 6.0% |
| D4A4R1 | Protein Eml3 | 95.639 | 32.107 | 3.6 | 2 | 2 | 2 | 0.95 | 6.2% |
| Q4KLH4 | Paraspeckle component 1 | 58.759 | 86.867 | 14.4 | 7 | 7 | 6 | 0.95 | 1.2% |
| Q9ERE6 | Myosin phosphatase Rho-interacting protein | 117.11 | 323.31 | 57.5 | 48 | 91 | 47 | 0.95 | 0.5% |
| B5DEL8 | NADH dehydrogenase (Ubiquinone) Fe-S protein 5 | 12.7 | 41.827 | 64.2 | 7 | 9 | 7 | 0.95 | 8.9% |
| Q4KM57 | LEM domain containing 2 | 36.243 | 12.538 | 14.1 | 4 | 4 | 4 | 0.95 | 9.9% |
| D3ZJZ0 | Protein Tmem205 | 21.185 | 3.9994 | 9 | 1 | 1 | 1 | 0.95 | 4.9% |
| Q5NDL0 | EGF domain-specific O-linked N-acetylglucosamine transferase | 61.523 | 10.717 | 8.3 | 4 | 5 | 4 | 0.95 | 4.8% |
| B2GUY0 | Endoplasmic reticulum mannosyl-oligosaccharide 1,2-alpha-mannosidase | 74.894 | 13.775 | 6.5 | 4 | 4 | 4 | 0.95 | 3.2% |
| D3ZHV2 | Microtubule-actin cross-linking factor 1 | 619.59 | 323.31 | 22.3 | 100 | 138 | 99 | 0.95 | 1.9% |
| Q05175 | Brain acid soluble protein 1 | 21.79 | 213.51 | 69.5 | 19 | 36 | 19 | 0.95 | 1.9% |
| A0A0G2K1P3 | Protein Bnip2 | 38.323 | 11.844 | 11.2 | 4 | 4 | 4 | 0.95 | 1.0% |
| F7F3Z1 | Lectin, mannose-binding 2-like (Predicted), isoform CRA_c | 40.087 | 5.4719 | 7.2 | 3 | 3 | 3 | 0.95 | 1.0% |
| F1LQZ0 | Protein Tmem65 | 27.733 | 19.829 | 17.4 | 4 | 4 | 4 | 0.95 | 1.2% |
| D4ADX8 | Protein Raph1 | 136.63 | 45.628 | 8.6 | 9 | 11 | 8 | 0.95 | 2.6% |
| P36407 | E3 ubiquitin-protein ligase TRIM23 | 63.892 | 4.9789 | 1.7 | 1 | 3 | 1 | 0.95 | 6.0% |
| Q9EPJ3 | 28S ribosomal protein S26, mitochondrial | 23.349 | 15.871 | 22 | 4 | 4 | 4 | 0.95 | 14.0% |
| P62856 | 40S ribosomal protein S26 | 13.015 | 28.275 | 27 | 3 | 8 | 3 | 0.95 | 4.4% |
| Q9WV97 | Mitochondrial import inner membrane translocase subunit Tim9 | 10.376 | 12.367 | 27 | 3 | 5 | 3 | 0.95 | 1.6% |
| Q9JLA3 | UDP-glucose:glycoprotein glucosyltransferase 1 | 176.43 | 261.92 | 27.2 | 33 | 57 | 32 | 0.95 | 0.8% |
| D4A9H6 | Protein St5 | 122.98 | 42.33 | 6.2 | 5 | 8 | 4 | 0.95 | 0.3% |
| Q91XU8 | Phosphatidate cytidylyltransferase 2 | 51.322 | 31.344 | 12.2 | 4 | 4 | 4 | 0.95 | 2.3% |
| D3ZPM7 | Protein Adam19 | 101.12 | 21.255 | 5.2 | 4 | 5 | 4 | 0.95 | 3.1% |
| A0A0G2JYI7 | Protein Ubap2 | 118.58 | 97.738 | 11.7 | 8 | 9 | 8 | 0.95 | 3.7% |
| Q5FVL2 | ER membrane protein complex subunit 8 | 23.405 | 90.729 | 43 | 7 | 9 | 7 | 0.95 | 5.9% |
| M0RDA4 | Receptor protein-tyrosine kinase | 108.73 | 8.3406 | 2.2 | 2 | 2 | 2 | 0.95 | 10.2% |
| D3ZBN0 | Histone H1.5 | 22.649 | 23.247 | 25.7 | 7 | 11 | 5 | 0.95 | 2.2% |
| P62890 | 60S ribosomal protein L30 | 12.784 | 54.686 | 50.4 | 7 | 13 | 7 | 0.95 | 2.8% |
| D4A0Q3 | tRNA (guanine-N(7)-)-methyltransferase non-catalytic subunit Wdr4 | 50.442 | 3.7318 | 3.3 | 1 | 1 | 1 | 0.95 | 11.9% |
| P54777 | Peroxisome assembly factor 2 | 104.43 | 11.46 | 1.7 | 1 | 1 | 1 | 0.95 | 17.6% |
| P97887 | Presenilin-1 | 52.789 | 2.7456 | 3.6 | 2 | 2 | 2 | 0.95 | 0.6% |
| Q9ES40 | PRA1 family protein 3 | 21.548 | 51.319 | 27.7 | 6 | 12 | 6 | 0.95 | 0.6% |
| Q6P7R8 | Estradiol 17-beta-dehydrogenase 12 | 34.84 | 70.583 | 27.6 | 7 | 15 | 7 | 0.95 | 0.5% |
| D3ZVF4 | Trafficking protein particle complex subunit 2 | 16.453 | 8.3137 | 15.7 | 2 | 2 | 2 | 0.95 | 0.3% |
| D3Z8R4 | Protein Rbm25l1 | 96.523 | 50.43 | 7.5 | 5 | 6 | 5 | 0.95 | 0.1% |
| E9PTX9 | Protein Slc12a2 | 130.37 | 16.555 | 3.6 | 4 | 4 | 4 | 0.95 | 1.2% |
| Q5XIA8 | Growth hormone-inducible transmembrane protein | 37.178 | 9.4866 | 9.5 | 3 | 3 | 3 | 0.95 | 1.6% |
| D3ZCV0 | Protein Actn2 | 103.83 | 2.6677 | 20.8 | 21 | 53 | 1 | 0.95 | 7.6% |
| G3V784 | ADP-dependent glucokinase, isoform CRA_a | 53.918 | 78.138 | 24 | 9 | 16 | 9 | 0.95 | 0.9% |
| Q05096 | Unconventional myosin-Ib | 131.92 | 157.39 | 26.5 | 28 | 34 | 28 | 0.95 | 0.4% |
| Q56B11 | Proline-, glutamic acid- and leucine-rich protein 1 | 119.14 | 8.162 | 2.7 | 2 | 3 | 2 | 0.95 | 0.7% |
| Q8CGS5 | Zinc phosphodiesterase ELAC protein 2 | 92.339 | 14.442 | 1.6 | 1 | 1 | 1 | 0.95 | 8.9% |
| P18418 | Calreticulin | 47.995 | 217.9 | 58.2 | 26 | 81 | 26 | 0.95 | 0.7% |
| Q498M4 | WD repeat-containing protein 5 | 36.588 | 22.406 | 9.3 | 3 | 4 | 3 | 0.95 | 0.4% |
| B2GV15 | Dihydrolipoamide branched chain transacylase E2 | 53.273 | 110.87 | 30.7 | 14 | 25 | 14 | 0.95 | 0.2% |
| Q66H12 | Alpha-N-acetylgalactosaminidase | 46.871 | 59.641 | 33.3 | 13 | 20 | 13 | 0.95 | 0.2% |
| D3ZF13 | Acyl carrier protein | 17.514 | 23.089 | 15.4 | 3 | 4 | 3 | 0.95 | 3.7% |
| D3ZCI9 | Protein Myl10 | 16.911 | 4.5175 | 6.8 | 1 | 1 | 1 | 0.95 | 4.9% |
| G3V6N7 | Protein Slc12a6 | 127.59 | 7.3347 | 1.7 | 2 | 2 | 1 | 0.95 | 5.6% |
| O88496 | Vitamin K-dependent gamma-carboxylase | 87.478 | 40.731 | 11.1 | 8 | 11 | 8 | 0.95 | 3.7% |
| G3V828 | Protein Cnpy3 | 30.647 | 76.049 | 34.4 | 8 | 8 | 8 | 0.95 | 3.4% |
| B0BNE6 | NADH dehydrogenase (Ubiquinone) Fe-S protein 8 (Predicted), isoform CRA_a | 23.97 | 33.554 | 34.9 | 7 | 11 | 7 | 0.95 | 3.3% |
| Q7TP42 | Ab2-292 | 67.93 | 13.955 | 6.3 | 4 | 6 | 4 | 0.95 | 2.8% |
| Q5PQL5 | Phosphatidylserine synthase 1 | 55.663 | 16.834 | 12.3 | 5 | 6 | 5 | 0.95 | 0.9% |
| Q6AY58 | B-cell receptor-associated protein 31 | 27.911 | 72.639 | 35.9 | 11 | 23 | 11 | 0.95 | 0.3% |
| D4AB01 | Histidine triad nucleotide binding protein 2 (Predicted), isoform CRA_a | 17.38 | 108.63 | 49.7 | 7 | 13 | 7 | 0.95 | 1.0% |
| Q5FVC2 | Rho guanine nucleotide exchange factor 2 | 111.91 | 67.69 | 14 | 11 | 18 | 11 | 0.95 | 1.3% |
| P97633 | Casein kinase I isoform alpha | 37.495 | 15.864 | 19.4 | 5 | 6 | 5 | 0.95 | 2.8% |
| P49432 | Pyruvate dehydrogenase E1 component subunit beta, mitochondrial | 38.982 | 131.45 | 43.2 | 14 | 40 | 14 | 0.95 | 1.6% |
| Q8CHN6 | Sphingosine-1-phosphate lyase 1 | 63.758 | 71.425 | 25 | 12 | 15 | 12 | 0.95 | 2.5% |
| F1LU03 | Glyceraldehyde-3-phosphate dehydrogenase | 32.217 | 3.4353 | 8 | 3 | 4 | 1 | 0.95 | 8.0% |
| D3Z9I1 | Protein Coa3 | 11.928 | 4.7308 | 8.3 | 1 | 1 | 1 | 0.95 | 4.2% |
| F1LV96 | Protein Scube3 | 108.94 | 4.7799 | 2.5 | 2 | 2 | 2 | 0.95 | 3.9% |
| D3ZIY3 | Protein Ythdf3 | 63.934 | 37.707 | 13.8 | 7 | 8 | 4 | 0.95 | 1.8% |
| F1M365 | Protein Ints9 | 74.059 | 2.0703 | 1.8 | 1 | 1 | 1 | 0.95 | 3.0% |
| Q6P6V6 | Protein GPR108 | 64.72 | 17.044 | 5 | 3 | 3 | 3 | 0.95 | 5.4% |
| D3ZUU5 | DnaJ (Hsp40) homolog, subfamily B, member 1 (Predicted), isoform CRA_a | 41.532 | 28.993 | 21.3 | 7 | 12 | 6 | 0.95 | 8.6% |
| D3ZZM0 | Protein Slc39a14 | 54.213 | 10.811 | 3.5 | 1 | 2 | 1 | 0.95 | 5.7% |
| Q3B7D0 | Oxygen-dependent coproporphyrinogen-III oxidase, mitochondrial | 49.278 | 28.295 | 14 | 5 | 5 | 5 | 0.95 | 0.5% |
| Q7TPJ0 | Translocon-associated protein subunit alpha | 35.629 | 19.495 | 10.7 | 3 | 6 | 3 | 0.95 | 0.1% |
| P0CE43 | Growth factor receptor-bound protein 10 | 67.882 | 103.15 | 20.2 | 10 | 14 | 9 | 0.95 | 0.1% |
| B5DEH2 | Erlin-2 | 37.71 | 66.397 | 29.8 | 9 | 12 | 9 | 0.95 | 0.4% |
| D4A9D6 | DEAH (Asp-Glu-Ala-His) box polypeptide 9 (Predicted) | 131.73 | 159.86 | 22.4 | 23 | 30 | 23 | 0.95 | 1.9% |
| Q63486 | Ras-related GTP-binding protein A | 36.566 | 30.482 | 20.8 | 6 | 8 | 6 | 0.95 | 1.9% |
| G3V8Y5 | DNA-directed RNA polymerase | 133.9 | 42.706 | 6.8 | 6 | 6 | 6 | 0.95 | 2.1% |
| A3KNA0 | Aqr protein | 170.76 | 7.7411 | 1.3 | 2 | 2 | 2 | 0.95 | 2.2% |
| P81795 | Eukaryotic translation initiation factor 2 subunit 3 | 51.079 | 65.451 | 36.7 | 16 | 23 | 5 | 0.95 | 3.8% |
| D3ZGV8 | Protein RGD1307838 | 310.92 | 67.064 | 8.3 | 21 | 23 | 20 | 0.95 | 0.7% |
| P29419 | ATP synthase subunit e, mitochondrial | 8.2545 | 23.008 | 62 | 4 | 8 | 4 | 0.95 | 0.1% |
| G3V912 | Protein Tmx4 | 37.54 | 18.047 | 7.4 | 2 | 2 | 2 | 0.95 | 1.4% |
| Q9Z0V6 | Thioredoxin-dependent peroxide reductase, mitochondrial | 28.295 | 121.32 | 40.9 | 9 | 23 | 9 | 0.95 | 2.1% |
| B1WC67 | Protein Slc25a24 | 52.89 | 179.15 | 53.7 | 23 | 44 | 23 | 0.95 | 2.2% |
| F1MA31 | Protein Kdm1a | 94.6 | 4.7604 | 1.6 | 1 | 2 | 1 | 0.95 | 3.9% |
| Q9Z1M9 | Structural maintenance of chromosomes protein 1A | 143.2 | 46.88 | 9.6 | 10 | 10 | 10 | 0.95 | 2.2% |
| Q641X9 | 39S ribosomal protein L9, mitochondrial | 30.076 | 6.5053 | 8.4 | 2 | 3 | 2 | 0.95 | 2.2% |
| B2RYW4 | Mitochondrial ribosomal protein L53 | 12.735 | 11.021 | 32.8 | 3 | 3 | 3 | 0.95 | 4.1% |
| Q6URK4 | Heterogeneous nuclear ribonucleoprotein A3 | 39.652 | 316.48 | 39.3 | 16 | 36 | 16 | 0.95 | 4.4% |
| P35435 | ATP synthase subunit gamma, mitochondrial | 30.19 | 65.47 | 33.3 | 9 | 29 | 9 | 0.95 | 0.9% |
| Q5U1Z2 | Trafficking protein particle complex subunit 3 | 20.302 | 5.8246 | 12.8 | 2 | 3 | 2 | 0.95 | 0.8% |
| F6PUQ7 | Protein Nmd3 | 57.746 | 7.9333 | 5 | 2 | 2 | 2 | 0.95 | 0.0% |
| D3ZTP0 | 10-formyltetrahydrofolate dehydrogenase | 101.8 | 215.1 | 37.2 | 30 | 53 | 30 | 0.95 | 0.3% |
| Q5XIK7 | Katanin p60 ATPase-containing subunit A-like 1 | 55.2 | 6.8708 | 4.9 | 3 | 3 | 3 | 0.95 | 0.3% |
| M0R7V3 | Protein Apoo | 22.629 | 35.085 | 26.8 | 5 | 6 | 5 | 0.95 | 0.7% |
| B5DFB2 | Protein Rbbp4 | 47.655 | 54.984 | 16.9 | 6 | 9 | 1 | 0.95 | 2.8% |
| Q66HF9 | Leucine-rich repeat flightless-interacting protein 1 | 80.018 | 206.32 | 36.9 | 21 | 34 | 20 | 0.95 | 2.3% |
| D4AB50 | Protein Arhgap10 | 89.44 | 13.325 | 4.8 | 4 | 5 | 4 | 0.95 | 1.4% |
| P10719 | ATP synthase subunit beta, mitochondrial | 56.353 | 323.31 | 66.4 | 23 | 94 | 23 | 0.95 | 1.8% |
| F1MA98 | Nucleoprotein TPR | 267.3 | 293.49 | 23.3 | 48 | 58 | 48 | 0.95 | 2.4% |
| P19139 | Casein kinase II subunit alpha | 45.073 | 72.789 | 28.1 | 9 | 11 | 9 | 0.95 | 2.8% |
| O08700 | Vacuolar protein sorting-associated protein 45 | 64.893 | 26.355 | 6.3 | 4 | 4 | 4 | 0.95 | 20.5% |
| Q80Z70 | Protein sel-1 homolog 1 | 88.682 | 68.035 | 9.1 | 6 | 6 | 6 | 0.95 | 0.5% |
| D3ZUN5 | Protein O-fucosyltransferase 2 (Predicted), isoform CRA_a | 49.543 | 80.764 | 36.8 | 18 | 23 | 18 | 0.95 | 0.2% |
| D3ZVB7 | Osteoglycin (Predicted) | 34.069 | 18.583 | 6.4 | 1 | 1 | 1 | 0.95 | 9.9% |
| B2RYN7 | Spastin | 63.021 | 9.6388 | 7.2 | 3 | 4 | 3 | 0.95 | 2.6% |
| F1LSG8 | Syndetin | 111.19 | 6.6103 | 2.5 | 2 | 3 | 2 | 0.95 | 0.4% |
| D3ZUI5 | Protein cordon-bleu | 122.27 | 20.972 | 3.4 | 3 | 4 | 3 | 0.95 | 0.1% |
| P41516 | DNA topoisomerase 2-alpha | 173.22 | 20.453 | 3.4 | 5 | 8 | 5 | 0.95 | 3.7% |
| A0A0G2K4C0 | Protein B4galt7 | 43.587 | 20.81 | 16 | 5 | 5 | 5 | 0.95 | 4.6% |
| F1LT09 | Protein Wdr33 | 145.41 | 2.2444 | 0.8 | 1 | 1 | 1 | 0.95 | 0.2% |
| D3ZZC5 | Protein Tradd | 34.53 | 33.582 | 17.1 | 4 | 6 | 4 | 0.95 | 2.4% |
| M0RAD5 | ATP-dependent Clp protease proteolytic subunit | 29.722 | 53.974 | 25 | 5 | 7 | 5 | 0.95 | 3.3% |
| Q5FVQ8 | NLR family member X1 | 107.59 | 72.4 | 12.4 | 8 | 10 | 8 | 0.95 | 3.7% |
| A0A0G2K022 | Protein Rcn3 | 38.942 | 157.52 | 34.7 | 10 | 24 | 10 | 0.95 | 3.4% |
| Q5U204 | Ragulator complex protein LAMTOR3 | 13.58 | 6.5188 | 8.9 | 1 | 6 | 1 | 0.95 | 3.3% |
| P14562 | Lysosome-associated membrane glycoprotein 1 | 43.969 | 65.12 | 17.4 | 7 | 19 | 7 | 0.95 | 1.7% |
| P17764 | Acetyl-CoA acetyltransferase, mitochondrial | 44.695 | 245.71 | 48.3 | 15 | 30 | 15 | 0.95 | 1.1% |
| D3ZV26 | Protein Sft2d3 | 22.351 | 10.107 | 10.1 | 1 | 1 | 1 | 0.95 | 3.0% |
| P85515 | Alpha-centractin | 42.613 | 151.74 | 40.4 | 13 | 23 | 5 | 0.95 | 3.6% |
| O88923 | Latrophilin-2 | 166.73 | 4.5191 | 0.7 | 1 | 1 | 1 | 0.95 | 1.4% |
| Q5XIN6 | LETM1 and EF-hand domain-containing protein 1, mitochondrial | 83.059 | 265.92 | 30.3 | 16 | 34 | 16 | 0.95 | 1.3% |
| Q63524 | Transmembrane emp24 domain-containing protein 2 | 22.733 | 42.432 | 33.8 | 7 | 25 | 7 | 0.95 | 2.2% |
| P07633 | Propionyl-CoA carboxylase beta chain, mitochondrial | 58.626 | 107.19 | 36 | 14 | 21 | 14 | 0.95 | 2.6% |
| Q9EQV6 | Tripeptidyl-peptidase 1 | 61.332 | 39.211 | 8.2 | 3 | 4 | 3 | 0.95 | 4.8% |
| B0BMZ1 | Protein RGD1305587 | 13.185 | 41.063 | 26.7 | 2 | 3 | 2 | 0.95 | 6.9% |
| D3ZPA1 | Protein Gcc1 | 87.747 | 40.642 | 6.9 | 5 | 5 | 5 | 0.95 | 4.1% |
| Q2YDU8 | Protein spinster homolog 1 | 56.795 | 10.013 | 6.6 | 3 | 4 | 3 | 0.95 | 2.5% |
| Q4V8H8 | EH domain-containing protein 2 | 61.237 | 83.559 | 30.6 | 14 | 24 | 11 | 0.95 | 1.8% |
| Q63357 | Unconventional myosin-Id | 116.09 | 323.31 | 43.5 | 40 | 70 | 40 | 0.95 | 0.4% |
| Q3KR86 | MICOS complex subunit Mic60 | 67.176 | 314.84 | 53.5 | 30 | 54 | 30 | 0.95 | 1.4% |
| P70705 | Copper-transporting ATPase 1 | 162.09 | 8.9822 | 2.8 | 4 | 4 | 4 | 0.95 | 1.5% |
| D4A944 | Protein Snrnp40 | 43.793 | 7.8037 | 7.2 | 3 | 3 | 3 | 0.95 | 2.2% |
| B2RYL3 | Protein Tmem119 | 29.864 | 42.529 | 22.6 | 5 | 6 | 5 | 0.95 | 8.2% |
| Q6AXQ5 | 2,5-phosphodiesterase 12 | 67.176 | 27.925 | 11.2 | 5 | 6 | 5 | 0.95 | 6.7% |
| Q8VH46 | Actin filament-associated protein 1 | 80.751 | 89.541 | 20.4 | 16 | 24 | 16 | 0.95 | 3.3% |
| Q5XI86 | Peptidyl-tRNA hydrolase 2 | 19.529 | 28.454 | 25.4 | 4 | 5 | 4 | 0.95 | 3.1% |
| B2RYC9 | Glucosylceramidase | 57.491 | 139.88 | 29.9 | 12 | 23 | 12 | 0.95 | 1.4% |
| Q00238 | Intercellular adhesion molecule 1 | 60.141 | 35.871 | 9.2 | 4 | 6 | 4 | 0.95 | 0.1% |
| B1WC56 | Nola2 protein | 17.292 | 43.447 | 39.2 | 4 | 5 | 4 | 0.95 | 0.3% |
| Q63617 | Hypoxia up-regulated protein 1 | 111.29 | 323.31 | 32.1 | 29 | 57 | 29 | 0.95 | 1.0% |
| Q6TMG5 | NF-kappa-B essential modulator | 48.066 | 51.906 | 14.3 | 5 | 7 | 5 | 0.95 | 1.1% |
| G3V6U3 | Asparagine-linked glycosylation 2 homolog (Yeast, alpha-1,3-mannosyltransferase), isoform CRA_a | 47.318 | 93.68 | 32.3 | 11 | 16 | 11 | 0.95 | 2.2% |
| B5DF48 | Protein Tyw1 | 82.086 | 17.32 | 6.1 | 4 | 5 | 4 | 0.95 | 2.5% |
| Q4KM38 | FUS interacting protein (Serine-arginine rich) 1 | 19.971 | 45.506 | 20.7 | 4 | 4 | 4 | 0.95 | 4.8% |
| Q3L7M0 | N-acetyllactosaminide alpha-1,3-galactosyltransferase | 43.541 | 3.5488 | 2.7 | 1 | 1 | 1 | 0.95 | 4.9% |
| Q9WVS2 | Probable tRNA N6-adenosine threonylcarbamoyltransferase | 36.356 | 16.403 | 17.3 | 5 | 5 | 5 | 0.95 | 5.4% |
| Q6MGB4 | H2-K region expressed gene 4, rat orthologue | 49.499 | 20.672 | 10.5 | 5 | 6 | 5 | 0.95 | 6.2% |
| Q63151 | Long-chain-fatty-acid--CoA ligase 3 | 80.457 | 53.903 | 12.2 | 7 | 9 | 7 | 0.95 | 3.6% |
| Q5PPI1 | Serine/arginine-rich splicing factor 9 | 25.498 | 10.443 | 5.4 | 1 | 1 | 1 | 0.95 | 2.3% |
| Q675A5 | Group XV phospholipase A2 | 47.391 | 22.466 | 13.6 | 4 | 4 | 4 | 0.95 | 2.0% |
| A0A0G2QC27 | Protein Nol6 | 128.07 | 9.5477 | 2.7 | 3 | 3 | 3 | 0.95 | 1.3% |
| P15800 | Laminin subunit beta-2 | 196.47 | 13.381 | 3.1 | 5 | 5 | 5 | 0.95 | 1.7% |
| P43244 | Matrin-3 | 94.446 | 66.922 | 17.2 | 14 | 20 | 14 | 0.95 | 1.7% |
| F6QBA3 | Protein Plin2 | 46.184 | 66.164 | 27.3 | 10 | 14 | 10 | 0.95 | 3.6% |
| P82450 | Sialate O-acetylesterase | 60.466 | 2.7776 | 2.2 | 1 | 1 | 1 | 0.95 | 3.6% |
| F1LZX5 | Protein Hectd4 | 452.5 | 10.876 | 1.1 | 4 | 4 | 4 | 0.95 | 3.8% |
| D3ZF34 |  | 59.692 | 67.448 | 20.8 | 9 | 18 | 2 | 0.95 | 4.2% |
| P20417 | Tyrosine-protein phosphatase non-receptor type 1 | 49.674 | 37.7 | 20.6 | 9 | 11 | 9 | 0.95 | 8.8% |
| D4A4Y0 | Protein Exosc4 | 26.391 | 7.2739 | 8.2 | 2 | 2 | 2 | 0.95 | 15.4% |
| D4ABY4 | Protein Ube2j1 | 27.78 | 24.565 | 20.2 | 4 | 4 | 4 | 0.95 | 6.1% |
| Q5U301 | A-kinase anchor protein 2 | 95.94 | 269.36 | 31.3 | 23 | 44 | 23 | 0.95 | 0.1% |
| D4AAG8 | Protein Smchd1 | 225.99 | 13.784 | 2.6 | 5 | 5 | 5 | 0.95 | 13.8% |
| P11240 | Cytochrome c oxidase subunit 5A, mitochondrial | 16.129 | 30.969 | 33.6 | 5 | 13 | 5 | 0.94 | 4.2% |
| D3ZF45 | Protein Larp4b | 81.084 | 7.8447 | 6 | 5 | 5 | 5 | 0.94 | 3.9% |
| P0C219 | Sarcolemmal membrane-associated protein | 98.223 | 149.77 | 24.1 | 21 | 37 | 21 | 0.94 | 2.3% |
| D3ZD97 | DEAH (Asp-Glu-Ala-His) box polypeptide 15 (Predicted), isoform CRA_b | 90.976 | 60.771 | 14 | 11 | 20 | 11 | 0.94 | 1.1% |
| G3V7V3 | Protein Slc27a4 | 72.188 | 88.719 | 28.3 | 15 | 17 | 13 | 0.94 | 1.4% |
| Q9Z270 | Vesicle-associated membrane protein-associated protein A | 27.841 | 79.182 | 46.2 | 13 | 21 | 12 | 0.94 | 2.6% |
| P21588 | 5-nucleotidase | 63.968 | 46.915 | 15.1 | 7 | 9 | 7 | 0.94 | 1.6% |
| Q6W3E9 | Prolyl 4-hydroxylase subunit alpha-3 | 61.165 | 127.67 | 22.4 | 12 | 17 | 12 | 0.94 | 1.5% |
| Q5SGE0 | Leucine-rich PPR motif-containing protein, mitochondrial | 156.65 | 219.72 | 31.9 | 43 | 64 | 43 | 0.94 | 0.5% |
| G3V661 | Bromodomain adjacent to zinc finger domain protein 1B | 170.29 | 7.3818 | 1.4 | 2 | 3 | 2 | 0.94 | 0.8% |
| Q00438 | Polypyrimidine tract-binding protein 1 | 59.353 | 232.22 | 41.6 | 14 | 23 | 11 | 0.94 | 5.5% |
| Q9R1J8 | Prolyl 3-hydroxylase 1 | 82.389 | 120.52 | 24.9 | 17 | 28 | 17 | 0.94 | 2.6% |
| M0R3M4 | Protein Ranbp2 | 344.39 | 134.52 | 9.2 | 26 | 28 | 25 | 0.94 | 1.7% |
| F1LR97 | Protein LOC304239 | 88.3 | -2 | 1.2 | 1 | 1 | 1 | 0.94 | 1.2% |
| D4ABA5 | Protein Smtn | 99.878 | 290.66 | 45.7 | 40 | 69 | 40 | 0.94 | 0.9% |
| Q924S5 | Lon protease homolog, mitochondrial | 105.79 | 238.28 | 31.4 | 26 | 52 | 26 | 0.94 | 0.9% |
| P00388 | NADPH--cytochrome P450 reductase | 76.962 | 88.341 | 25.7 | 14 | 18 | 14 | 0.94 | 0.8% |
| P11507 | Sarcoplasmic/endoplasmic reticulum calcium ATPase 2 | 114.77 | 323.31 | 40 | 35 | 81 | 35 | 0.94 | 0.5% |
| P30427 | Plectin | 533.53 | 323.31 | 51 | 226 | 392 | 225 | 0.94 | 0.2% |
| Q63065 | [Pyruvate dehydrogenase (acetyl-transferring)] kinase isozyme 1, mitochondrial | 49.08 | 3.986 | 2.5 | 1 | 1 | 1 | 0.94 | 5.2% |
| O08628 | Procollagen C-endopeptidase enhancer 1 | 50.184 | 94.469 | 29.3 | 12 | 14 | 12 | 0.94 | 3.7% |
| Q0ZFS4 | Mitochondrial ribosomal protein S33 (Predicted), isoform CRA_a | 12.372 | 36.01 | 39 | 3 | 4 | 3 | 0.94 | 3.1% |
| F2Z3T9 | Protein U2af2 | 53.516 | 70.671 | 20.6 | 9 | 16 | 9 | 0.94 | 2.2% |
| A0A0G2K1Q8 | Protein Abca3 | 191.77 | 34.234 | 3.1 | 5 | 5 | 5 | 0.94 | 1.4% |
| B1H257 | Loss of heterozygosity, 12, chromosomal region 1 homolog (Human) | 22.176 | 20.826 | 24.1 | 4 | 6 | 4 | 0.94 | 3.8% |
| D4A264 | Protein Zadh2 | 40.535 | 27.812 | 14.3 | 4 | 4 | 4 | 0.94 | 4.6% |
| A0A0G2K0L0 | Protein Papss1 | 70.562 | 52.338 | 10.4 | 4 | 4 | 4 | 0.94 | 5.0% |
| D3ZD11 | Protein Spcs2 | 24.973 | 76.008 | 47.3 | 10 | 15 | 10 | 0.94 | 5.0% |
| O09014 | Solute carrier family 15 member 4 | 61.948 | 7.0152 | 4.2 | 2 | 2 | 2 | 0.94 | 5.0% |
| A0A0G2JV51 | Protein Nat10 | 115.36 | 25.259 | 10.6 | 10 | 11 | 10 | 0.94 | 7.0% |
| A0A0G2K6D5 | Protein Pitrm1 | 117.42 | 107.18 | 20.2 | 18 | 23 | 18 | 0.94 | 0.5% |
| Q9QYF3 | Unconventional myosin-Va | 211.76 | 158.04 | 15.1 | 23 | 26 | 23 | 0.94 | 0.2% |
| D3ZAI0 | Protein U2surp | 118.23 | 28.415 | 6.3 | 6 | 6 | 6 | 0.94 | 4.0% |
| Q5FVM6 | Myotubularin-related protein 12 | 85.685 | 3.5575 | 2.7 | 2 | 2 | 2 | 0.94 | 3.2% |
| Q810U0 | Coiled-coil domain-containing protein 50 | 35.158 | 40.186 | 23.3 | 6 | 8 | 6 | 0.94 | 3.1% |
| Q0ZHH6 | Atlastin-3 | 60.585 | 182.47 | 34.4 | 14 | 39 | 14 | 0.94 | 1.8% |
| O88791 | High mobility group AT-hook 2 | 11.676 | 37.686 | 33.6 | 2 | 3 | 2 | 0.94 | 1.6% |
| F1LZW6 | Protein Slc25a13 | 54.099 | 81.465 | 21.7 | 8 | 12 | 6 | 0.94 | 1.5% |
| Q63321 | Procollagen-lysine,2-oxoglutarate 5-dioxygenase 1 | 83.611 | 158.8 | 32.4 | 21 | 38 | 21 | 0.94 | 1.5% |
| M0R635 | N-alpha-acetyltransferase 11 | 24.767 | 12.307 | 16.8 | 4 | 4 | 2 | 0.94 | 1.2% |
| B2RZ79 | Iron-sulfur cluster scaffold homolog (E. coli) | 17.967 | 46.341 | 41.3 | 6 | 10 | 6 | 0.94 | 0.9% |
| Q5XIU4 | B-cell receptor-associated protein 29 | 28.234 | 27.932 | 34.4 | 11 | 16 | 11 | 0.94 | 0.9% |
| P21533 | 60S ribosomal protein L6 | 33.561 | 80.841 | 38.3 | 16 | 30 | 16 | 0.94 | 0.6% |
| G3V6R4 | Protein Lrrc1 | 59.387 | 4.5889 | 4.6 | 2 | 2 | 1 | 0.94 | 1.3% |
| D3ZFQ8 | Cytochrome c-1 (Predicted), isoform CRA_c | 35.434 | 56.067 | 30.4 | 7 | 10 | 7 | 0.94 | 2.2% |
| Q6P7A9 | Lysosomal alpha-glucosidase | 106.21 | 266.42 | 23.3 | 20 | 41 | 20 | 0.94 | 5.7% |
| Q62764 | Y-box-binding protein 3 | 38.851 | 16.834 | 15.5 | 4 | 8 | 3 | 0.94 | 0.5% |
| P45953 | Very long-chain specific acyl-CoA dehydrogenase, mitochondrial | 70.748 | 212.22 | 44.4 | 24 | 37 | 24 | 0.94 | 0.5% |
| G3V9R8 | Heterogeneous nuclear ribonucleoprotein C | 32.857 | 116.15 | 41.3 | 12 | 30 | 12 | 0.94 | 2.5% |
| Q62902 | Protein ERGIC-53 | 57.956 | 160.15 | 30.8 | 14 | 20 | 14 | 0.94 | 0.6% |
| P04897 | Guanine nucleotide-binding protein G(i) subunit alpha-2 | 40.499 | 219.72 | 65.6 | 20 | 47 | 12 | 0.94 | 3.3% |
| F1M9Z9 | Protein Adam12 | 96.517 | 8.6889 | 3.5 | 3 | 3 | 3 | 0.94 | 3.9% |
| Q925R7 | Polypeptide N-acetylgalactosaminyltransferase 10 | 69.116 | 20.988 | 13.3 | 6 | 8 | 6 | 0.94 | 2.0% |
| Q66HF1 | NADH-ubiquinone oxidoreductase 75 kDa subunit, mitochondrial | 79.411 | 244.41 | 43.6 | 25 | 38 | 25 | 0.94 | 1.1% |
| D4ABI7 | Protein Hacd3 | 43.104 | 9.2203 | 7.2 | 3 | 5 | 3 | 0.94 | 0.7% |
| Q6AYS6 | Sorting nexin-17 | 52.882 | 58.171 | 19.8 | 6 | 7 | 6 | 0.94 | 0.1% |
| D4A1D3 | Protein Sacs | 521.48 | 63.003 | 4 | 17 | 19 | 17 | 0.94 | 0.5% |
| Q63060 | Glycerol kinase | 57.476 | 16.725 | 6.7 | 3 | 3 | 3 | 0.94 | 0.6% |
| A2RUV9 | Adipocyte enhancer-binding protein 1 | 128.06 | 15.242 | 5.1 | 5 | 5 | 5 | 0.94 | 2.8% |
| Q75Q41 | Mitochondrial import receptor subunit TOM22 homolog | 15.49 | 16.618 | 16.2 | 2 | 7 | 2 | 0.94 | 4.5% |
| B2RYT0 | Mitochondrial ribosomal protein S21 (Predicted), isoform CRA_a | 10.6 | 14.281 | 29.9 | 3 | 4 | 3 | 0.94 | 3.5% |
| Q9Z158 | Syntaxin-17 | 33.182 | 2.6718 | 3 | 1 | 1 | 1 | 0.94 | 2.8% |
| Q4QQV8 | Charged multivesicular body protein 5 | 24.575 | 45.163 | 31.5 | 5 | 9 | 5 | 0.94 | 0.7% |
| D4A512 | Protein Nemf | 93.444 | 6.2423 | 3.3 | 3 | 3 | 3 | 0.94 | 3.6% |
| Q2KN99 | Cytospin-A | 124.34 | 115.57 | 19.3 | 20 | 25 | 20 | 0.94 | 5.7% |
| D3ZGY2 | Protein Otud6b | 36.274 | 49.863 | 30.7 | 8 | 11 | 8 | 0.94 | 5.8% |
| B1WC84 | Canopy 4 homolog (Zebrafish) | 28.384 | 118.25 | 30.4 | 7 | 18 | 7 | 0.94 | 0.2% |
| Q5XIS1 | Protein phosphatase Slingshot homolog 3 | 72.07 | 21.565 | 5.5 | 3 | 3 | 3 | 0.94 | 6.2% |
| P62634 | Cellular nucleic acid-binding protein | 19.463 | 61.031 | 36.7 | 5 | 10 | 5 | 0.94 | 1.5% |
| Q561S0 | NADH dehydrogenase [ubiquinone] 1 alpha subcomplex subunit 10, mitochondrial | 40.493 | 83.82 | 34.1 | 12 | 16 | 12 | 0.94 | 1.2% |
| P38659 | Protein disulfide-isomerase A4 | 72.719 | 316.6 | 48.5 | 38 | 84 | 38 | 0.94 | 1.9% |
| P52873 | Pyruvate carboxylase, mitochondrial | 129.78 | 96.267 | 18.3 | 17 | 21 | 17 | 0.94 | 2.7% |
| F6Q5K7 | Mitochondrial ribosomal protein S18B, isoform CRA_a | 29.159 | 21.315 | 21.8 | 4 | 5 | 4 | 0.94 | 4.0% |
| D3ZEA8 | Protein Syne1 | 315.79 | 61.49 | 3.8 | 9 | 9 | 9 | 0.94 | 6.8% |
| Q4G067 | Mitochondrial ribosomal protein L44 | 37.44 | 13.791 | 5.7 | 1 | 1 | 1 | 0.94 | 12.2% |
| F1M6E5 | Protein Nacad | 147.6 | 3.9992 | 2.5 | 3 | 3 | 3 | 0.94 | 11.7% |
| Q63083 | Nucleobindin-1 | 53.506 | 208.13 | 51.6 | 24 | 41 | 24 | 0.94 | 0.1% |
| Q498C7 | Small integral membrane protein 14 | 10.684 | 2.0923 | 13.1 | 1 | 1 | 1 | 0.94 | 11.3% |
| G3V728 | 4-nitrophenylphosphatase domain and non-neuronal SNAP25-like protein homolog 1 (C. elegans), isoform CRA_b | 33.346 | 20.757 | 10.9 | 3 | 3 | 3 | 0.94 | 13.5% |
| Q6TEK4 | Vitamin K epoxide reductase complex subunit 1 | 17.783 | 10.179 | 8.1 | 1 | 1 | 1 | 0.94 | 10.2% |
| P58366 | Progressive ankylosis protein homolog | 54.264 | 4.6954 | 2.4 | 1 | 1 | 1 | 0.94 | 7.7% |
| P11951 | Cytochrome c oxidase subunit 6C-2 | 8.4548 | 12.673 | 52.6 | 4 | 10 | 4 | 0.94 | 4.9% |
| A0A0G2JXJ6 | Protein Ttc7b | 64.913 | 14.04 | 7.4 | 4 | 4 | 4 | 0.94 | 3.4% |
| B0BNJ4 | Ethylmalonic encephalopathy 1 | 27.676 | 34.971 | 24 | 5 | 7 | 5 | 0.94 | 0.7% |
| P55062 | Bax inhibitor 1 | 26.463 | 4.8538 | 3.8 | 2 | 2 | 2 | 0.94 | 0.5% |
| Q5XIC0 | Enoyl-CoA delta isomerase 2, mitochondrial | 43.021 | 47.648 | 16.6 | 6 | 12 | 6 | 0.94 | 0.3% |
| F1LUU1 | Protein Itga11 | 121.76 | 269.53 | 30.7 | 27 | 57 | 27 | 0.94 | 0.1% |
| Q3B7V5 | Protein Rab2b | 24.085 | 41.696 | 53 | 9 | 23 | 3 | 0.94 | 0.5% |
| D3ZTF6 | Phosphatidylinositol 3-kinase, C2 domain containing, alpha polypeptide (Predicted), isoform CRA_a | 190.91 | 39.157 | 7.6 | 12 | 15 | 12 | 0.94 | 1.7% |
| P08289 | Alkaline phosphatase, tissue-nonspecific isozyme | 57.659 | 187.88 | 42.2 | 18 | 52 | 18 | 0.94 | 2.0% |
| Q6GQP4 | Ras-related protein Rab-31 | 21.368 | 74.327 | 42.8 | 7 | 14 | 5 | 0.94 | 2.3% |
| M0R7B4 | Protein LOC684828 | 22.233 | 3.6991 | 26.8 | 9 | 20 | 2 | 0.94 | 4.0% |
| O08564 | Lipid phosphate phosphohydrolase 1 | 31.996 | 14.223 | 8.9 | 2 | 2 | 2 | 0.94 | 3.5% |
| Q5RJR8 | Leucine-rich repeat-containing protein 59 | 34.869 | 185.2 | 46.6 | 15 | 32 | 15 | 0.94 | 3.0% |
| Q5M7T6 | ATPase, H+ transporting, lysosomal 38kDa, V0 subunit d1 | 40.301 | 97.31 | 39 | 13 | 21 | 13 | 0.94 | 1.6% |
| F1LM66 | Protein Eftud2 | 109.48 | 178.62 | 21.6 | 16 | 27 | 15 | 0.94 | 1.2% |
| E9PU29 | Protein Rnf31 | 119.24 | 21.653 | 2.6 | 2 | 2 | 2 | 0.94 | 4.0% |
| P51146 | Ras-related protein Rab-4B | 23.629 | 23.344 | 35.7 | 6 | 7 | 6 | 0.94 | 4.3% |
| Q9JJL4 | Rho-related GTP-binding protein RhoQ | 22.645 | 19.586 | 17.6 | 3 | 7 | 2 | 0.94 | 1.7% |
| Q66HF2 | Transmembrane 9 superfamily member 1 | 66.918 | 16.753 | 7.1 | 4 | 4 | 4 | 0.94 | 1.6% |
| D4A6L1 | Protein Tapbpl | 48.27 | 36.516 | 9.9 | 3 | 5 | 3 | 0.94 | 0.5% |
| Q9ER34 | Aconitate hydratase, mitochondrial | 85.432 | 323.31 | 47.8 | 32 | 76 | 32 | 0.94 | 0.2% |
| D4A3E2 | Aminopeptidase-like 1 (Predicted) | 43.682 | 49.329 | 14.8 | 4 | 5 | 4 | 0.94 | 2.4% |
| Q4FZS1 | Chromobox homolog 8 (Drosophila, Pc class), isoform CRA_a | 40.412 | 23.677 | 7.4 | 2 | 2 | 2 | 0.94 | 2.6% |
| Q63362 | NADH dehydrogenase [ubiquinone] 1 alpha subcomplex subunit 5 | 13.412 | 12.362 | 38.8 | 3 | 5 | 3 | 0.94 | 4.8% |
| Q5U3Z3 | Isochorismatase domain-containing protein 2, mitochondrial | 23.157 | 19.034 | 7.6 | 1 | 1 | 1 | 0.94 | 0.1% |
| Q6AYA6 | Uncharacterized protein C17orf62 homolog | 20.894 | 21.893 | 20.9 | 4 | 5 | 4 | 0.94 | 10.4% |
| D3ZKU7 | Biogenesis of lysosome-related organelles complex 1 subunit 1 | 14.311 | 56.047 | 54.4 | 4 | 6 | 4 | 0.94 | 4.6% |
| P58195 | Phospholipid scramblase 1 | 36.71 | 6.3469 | 3 | 1 | 1 | 1 | 0.94 | 7.5% |
| Q9WUQ1 | A disintegrin and metalloproteinase with thrombospondin motifs 1 | 105.7 | 15.636 | 2 | 1 | 1 | 1 | 0.94 | 5.3% |
| Q6AYN2 | Store-operated calcium entry-associated regulatory factor | 35.885 | 22.385 | 13.8 | 4 | 4 | 4 | 0.94 | 4.7% |
| P04166 | Cytochrome b5 type B | 16.265 | 26.692 | 32.9 | 4 | 7 | 4 | 0.94 | 3.5% |
| Q68FW7 | Threonine--tRNA ligase, mitochondrial | 81.671 | 51.839 | 19.4 | 12 | 15 | 11 | 0.94 | 2.8% |
| Q5QD51 | A-kinase anchor protein 12 | 181.11 | 323.31 | 32.1 | 42 | 90 | 42 | 0.94 | 1.2% |
| D3ZGT6 | Procollagen-proline, 2-oxoglutarate 4-dioxygenase (Proline 4-hydroxylase), alpha II polypeptide (Predicted), isoform CRA_a | 60.866 | 193.69 | 50.1 | 22 | 48 | 22 | 0.94 | 0.9% |
| P20909 | Collagen alpha-1(XI) chain | 181.02 | 115.85 | 13.2 | 20 | 29 | 15 | 0.94 | 0.4% |
| F1M842 | Protein Tp53bp1 | 212.86 | 12.73 | 2.1 | 3 | 3 | 3 | 0.94 | 0.2% |
| Q8VHF5 | Citrate synthase, mitochondrial | 51.866 | 88.133 | 33.7 | 16 | 38 | 16 | 0.94 | 0.5% |
| D3ZZ21 | NADH dehydrogenase (Ubiquinone) 1 beta subcomplex, 6 (Predicted) | 15.638 | 15.8 | 14.1 | 1 | 3 | 1 | 0.94 | 1.7% |
| F1M2M4 |  | 32.179 | 28.112 | 8.1 | 1 | 6 | 1 | 0.94 | 5.0% |
| G3V619 | Activating signal cointegrator 1 complex subunit 1, isoform CRA_b | 41.179 | 12.063 | 7.9 | 4 | 6 | 4 | 0.94 | 5.7% |
| D4A104 | Mitochondrial ribosomal protein L45 (Predicted) | 35.439 | 10.855 | 11.4 | 2 | 3 | 2 | 0.94 | 4.2% |
| F1M8K0 | Protein Dag1 | 96.705 | 55.003 | 8 | 5 | 6 | 5 | 0.94 | 2.2% |
| Q5U316 | Ras-related protein Rab-35 | 23.025 | 41.841 | 36.3 | 7 | 13 | 7 | 0.94 | 0.3% |
| Q9ESZ0 | DNA repair protein XRCC1 | 68.835 | 3.0679 | 1.4 | 1 | 1 | 1 | 0.94 | 2.3% |
| P11915 | Non-specific lipid-transfer protein | 58.813 | 102.56 | 22.5 | 13 | 29 | 13 | 0.94 | 3.0% |
| D4A0Y4 | Oxidoreductase NAD-binding domain containing 1 (Predicted), isoform CRA_b | 37.427 | 11.631 | 9 | 3 | 3 | 3 | 0.94 | 3.4% |
| Q6AYA1 | H/ACA ribonucleoprotein complex subunit 1 | 23.01 | 23.555 | 17.3 | 4 | 4 | 4 | 0.94 | 3.6% |
| P08082 | Clathrin light chain B | 25.117 | 22.622 | 25.3 | 9 | 14 | 9 | 0.94 | 1.8% |
| P62074 | Mitochondrial import inner membrane translocase subunit Tim10 | 10.333 | 6.0954 | 24.4 | 2 | 3 | 2 | 0.94 | 1.7% |
| P63039 | 60 kDa heat shock protein, mitochondrial | 60.955 | 323.31 | 62 | 34 | 108 | 34 | 0.94 | 0.9% |
| Q9ES72 | Protein CYR61 | 41.687 | 35.198 | 19.3 | 7 | 8 | 7 | 0.94 | 3.0% |
| B1WBW4 | Armadillo repeat-containing protein 10 | 33.421 | 52.33 | 22.9 | 5 | 9 | 5 | 0.94 | 3.9% |
| Q8R4A1 | ERO1-like protein alpha | 54.018 | 56.371 | 27.2 | 11 | 15 | 10 | 0.94 | 3.2% |
| Q5BK63 | NADH dehydrogenase [ubiquinone] 1 alpha subcomplex subunit 9, mitochondrial | 42.559 | 59.526 | 28.9 | 10 | 16 | 10 | 0.94 | 7.0% |
| Q5FVH0 | Complement C1q tumor necrosis factor-related protein 5 | 25.334 | 40.985 | 19.8 | 3 | 7 | 3 | 0.94 | 7.5% |
| D3ZT52 | Protein Pbrm1 | 197.18 | 6.7147 | 1.6 | 3 | 3 | 3 | 0.94 | 9.1% |
| Q9WVA1 | Mitochondrial import inner membrane translocase subunit Tim8 A | 11.042 | 35.634 | 49.5 | 4 | 7 | 4 | 0.94 | 4.2% |
| F1M589 | Protein Pml | 98.11 | 9.8948 | 4.7 | 4 | 4 | 4 | 0.94 | 1.6% |
| D4A914 | 5-3 exoribonuclease 2 (Predicted), isoform CRA_a | 108.65 | 21.773 | 6.7 | 5 | 9 | 5 | 0.94 | 1.1% |
| P29975 | Aquaporin-1 | 28.856 | 45.024 | 17.8 | 5 | 17 | 5 | 0.94 | 0.7% |
| Q66H94 | Peptidyl-prolyl cis-trans isomerase FKBP9 | 63.126 | 84.815 | 27.4 | 15 | 36 | 14 | 0.94 | 1.3% |
| P85834 | Elongation factor Tu, mitochondrial | 49.522 | 190.53 | 56.6 | 20 | 44 | 20 | 0.94 | 1.6% |
| D4A3T3 | Protein Cbx1 | 21.403 | 39.43 | 18.9 | 3 | 10 | 3 | 0.94 | 2.4% |
| D4ACN8 | Plasminogen receptor (KT) | 17.312 | 14.358 | 23.1 | 3 | 8 | 3 | 0.94 | 1.5% |
| Q9JHY2 | Sideroflexin-3 | 35.433 | 106.03 | 37.1 | 10 | 23 | 9 | 0.94 | 1.5% |
| Q3KRE0 | ATPase family AAA domain-containing protein 3 | 66.758 | 103.42 | 27.9 | 13 | 16 | 13 | 0.94 | 0.8% |
| F1M9C9 | Protein Hars2 | 57.327 | 17.909 | 10.1 | 5 | 5 | 3 | 0.94 | 1.1% |
| Q68FX0 | Isocitrate dehydrogenase [NAD] subunit beta, mitochondrial | 42.353 | 92.011 | 31.7 | 9 | 20 | 9 | 0.94 | 5.6% |
| P51577 | P2X purinoceptor 4 | 43.501 | 5.506 | 4.1 | 1 | 3 | 1 | 0.94 | 10.6% |
| Q6MG85 | 1-acyl-sn-glycerol-3-phosphate acyltransferase | 31.698 | 50.756 | 25.7 | 5 | 6 | 5 | 0.94 | 8.9% |
| Q6TXI6 | LRRGT00013 | 31.357 | 20.624 | 17.5 | 4 | 4 | 4 | 0.94 | 4.3% |
| P62762 | Visinin-like protein 1 | 22.142 | 55.518 | 49.2 | 8 | 14 | 8 | 0.94 | 1.5% |
| A0A0G2JZZ4 | Protein Selm | 16.21 | 50.442 | 45.8 | 5 | 7 | 5 | 0.94 | 0.6% |
| Q63632 | Solute carrier family 12 member 4 | 120.63 | 62.503 | 13.2 | 11 | 12 | 10 | 0.94 | 2.1% |
| P19234 | NADH dehydrogenase [ubiquinone] flavoprotein 2, mitochondrial | 27.378 | 57.086 | 35.9 | 7 | 12 | 7 | 0.94 | 4.0% |
| M0RBJ0 | Guanine nucleotide-binding protein subunit gamma | 4.8827 | 8.2943 | 36.4 | 1 | 2 | 1 | 0.94 | 5.8% |
| P54001 | Prolyl 4-hydroxylase subunit alpha-1 | 60.897 | 253.84 | 43.6 | 26 | 49 | 26 | 0.93 | 0.3% |
| Q4V8E1 | GATA zinc finger domain containing 2B | 65.26 | 18.449 | 6.6 | 3 | 3 | 3 | 0.93 | 7.2% |
| Q6AYT7 | Monoacylglycerol lipase ABHD12 | 45.295 | 71.157 | 25.1 | 9 | 13 | 9 | 0.93 | 6.0% |
| D3ZNG3 | Protein Sppl2a | 59.836 | 16.39 | 2.6 | 1 | 2 | 1 | 0.93 | 3.7% |
| Q6AYK5 | Cell growth-regulating nucleolar protein | 43.68 | 6.422 | 3.6 | 1 | 1 | 1 | 0.93 | 3.6% |
| B0BMT9 | Protein Sqrdl | 50.201 | 211.37 | 50.4 | 22 | 38 | 22 | 0.93 | 1.0% |
| Q5BJT0 | Arginine and glutamate-rich protein 1 | 32.887 | 8.5715 | 14 | 5 | 6 | 5 | 0.93 | 0.6% |
| P48721 | Stress-70 protein, mitochondrial | 73.857 | 323.31 | 58.3 | 40 | 111 | 39 | 0.93 | 0.5% |
| P24368 | Peptidyl-prolyl cis-trans isomerase B | 23.802 | 105.84 | 61.6 | 14 | 49 | 14 | 0.93 | 1.2% |
| P13264 | Glutaminase kidney isoform, mitochondrial | 74.023 | 323.31 | 53.1 | 30 | 74 | 30 | 0.93 | 1.4% |
| Q5PQL7 | Integral membrane protein 2C | 30.48 | 12.314 | 11.2 | 3 | 4 | 3 | 0.93 | 2.3% |
| D3ZPL1 | Cleavage and polyadenylation specific factor 6, 68kDa (Predicted), isoform CRA_b | 59.179 | 63.148 | 18.1 | 7 | 9 | 7 | 0.93 | 5.0% |
| P08050 | Gap junction alpha-1 protein | 43.031 | 7.2697 | 5.8 | 2 | 2 | 2 | 0.93 | 7.1% |
| Q63199 | Tumor necrosis factor receptor superfamily member 6 | 36.834 | 24.535 | 13.3 | 3 | 3 | 3 | 0.93 | 8.6% |
| P41565 | Isocitrate dehydrogenase [NAD] subunit gamma 1, mitochondrial | 42.851 | 63.884 | 18.1 | 7 | 10 | 7 | 0.93 | 5.2% |
| D3ZPP9 | Protein RGD1306058 | 23.328 | 8.2937 | 15.8 | 3 | 3 | 3 | 0.93 | 1.3% |
| D3ZAN3 | Alpha glucosidase 2 alpha neutral subunit (Predicted) | 90.571 | 225.63 | 33 | 25 | 48 | 25 | 0.93 | 3.1% |
| O35412 | Signal-induced proliferation-associated 1-like protein 1 | 201.92 | 33.811 | 6.2 | 9 | 11 | 9 | 0.93 | 4.3% |
| B2GV58 | Lsm14a protein | 51.226 | 30.917 | 8.8 | 4 | 5 | 4 | 0.93 | 8.9% |
| D4A208 | SLIT-ROBO Rho GTPase-activating protein 2 | 120.88 | 48.594 | 9.7 | 8 | 8 | 6 | 0.93 | 2.6% |
| Q794F9 | 4F2 cell-surface antigen heavy chain | 58.071 | 179.53 | 28.1 | 12 | 25 | 12 | 0.93 | 1.2% |
| D3ZFJ6 | Lactamase, beta (Predicted) | 60.42 | 2.6196 | 3.8 | 2 | 2 | 2 | 0.93 | 0.9% |
| Q07266 | Drebrin | 77.471 | 320.64 | 38.5 | 26 | 50 | 26 | 0.93 | 1.0% |
| A0A0G2JTK6 | Protein Mta3 | 65.707 | 6.5923 | 6.7 | 4 | 4 | 1 | 0.93 | 1.3% |
| P27615 | Lysosome membrane protein 2 | 54.09 | 47.382 | 13.8 | 6 | 6 | 6 | 0.93 | 1.6% |
| Q4V884 | CDC16 cell division cycle 16 homolog (S. cerevisiae) | 71.36 | 85.791 | 14.8 | 6 | 6 | 6 | 0.93 | 7.3% |
| A0A0G2K016 | Protein Sun1 | 102.4 | 8.3827 | 2.2 | 2 | 2 | 2 | 0.93 | 8.9% |
| Q5U2X8 | Acyl-CoA thioesterase 9 | 50.443 | 93.249 | 36.7 | 17 | 27 | 17 | 0.93 | 0.8% |
| D3ZYW7 | Frataxin, mitochondrial | 23.066 | 10.68 | 11.1 | 2 | 3 | 2 | 0.93 | 0.5% |
| Q63803 | Guanine nucleotide-binding protein G(s) subunit alpha isoforms XLas | 122.89 | 49.07 | 11.7 | 12 | 20 | 11 | 0.93 | 0.1% |
| Q9WU82 | Catenin beta-1 | 85.454 | 151.85 | 27.9 | 19 | 31 | 16 | 0.93 | 0.1% |
| F1LM47 | Succinyl-CoA ligase subunit beta | 50.306 | 111.66 | 49.2 | 21 | 37 | 21 | 0.93 | 0.2% |
| P18614 | Integrin alpha-1 | 130.81 | 182.83 | 16.9 | 21 | 35 | 21 | 0.93 | 1.2% |
| Q80W89 | NADH dehydrogenase [ubiquinone] 1 alpha subcomplex subunit 11 | 14.854 | 16.248 | 19.1 | 2 | 3 | 2 | 0.93 | 2.6% |
| P08461 | Dihydrolipoyllysine-residue acetyltransferase component of pyruvate dehydrogenase complex, mitochondrial | 67.165 | 206.08 | 39.4 | 19 | 36 | 19 | 0.93 | 3.6% |
| Q64654 | Lanosterol 14-alpha demethylase | 56.706 | 53.649 | 18.5 | 8 | 9 | 8 | 0.93 | 0.2% |
| Q6VEU8 | DEAD (Asp-Glu-Ala-Asp) box polypeptide 24 | 95.292 | 5.0405 | 1.4 | 1 | 1 | 1 | 0.93 | 6.8% |
| Q5U2Q3 | Ester hydrolase C11orf54 homolog | 34.993 | 10.021 | 12.1 | 4 | 5 | 4 | 0.93 | 4.8% |
| Q9R0L4 | Cullin-associated NEDD8-dissociated protein 2 | 139.67 | 31.358 | 5.1 | 5 | 6 | 4 | 0.93 | 2.8% |
| P97829 | Leukocyte surface antigen CD47 | 32.995 | 18.985 | 9.9 | 3 | 9 | 3 | 0.93 | 1.4% |
| Q6DKG0 | N-alpha-acetyltransferase 35, NatC auxiliary subunit | 83.209 | 2.1851 | 2.1 | 2 | 2 | 2 | 0.93 | 1.2% |
| Q9WVH8 | Fibulin-5 | 50.16 | 44.89 | 20.1 | 8 | 9 | 8 | 0.93 | 2.6% |
| M0R850 |  | 23.655 | 2.9891 | 3.8 | 1 | 1 | 1 | 0.93 | 2.1% |
| F7FJQ3 | Niemann Pick type C2 | 16.748 | 36.597 | 36.8 | 7 | 14 | 7 | 0.93 | 2.4% |
| F1LQC5 | Receptor protein serine/threonine kinase | 114.88 | 10.495 | 2.8 | 3 | 3 | 3 | 0.93 | 5.8% |
| D3ZMR1 | Protein Tomm7 | 6.1773 | 4.2545 | 30.9 | 1 | 2 | 1 | 0.93 | 4.7% |
| Q63269 | Inositol 1,4,5-trisphosphate receptor type 3 | 304.28 | 3.456 | 2.2 | 8 | 11 | 2 | 0.93 | 2.7% |
| F1LS72 | Protein Uba2 | 66.338 | 69.807 | 29.7 | 13 | 15 | 13 | 0.93 | 2.0% |
| Q9Z2J4 | Nexilin | 78.392 | 150.92 | 39.6 | 27 | 48 | 27 | 0.93 | 0.8% |
| D3ZG43 | NADH dehydrogenase (Ubiquinone) Fe-S protein 3 (Predicted), isoform CRA_c | 30.226 | 82.562 | 33 | 9 | 16 | 9 | 0.93 | 0.4% |
| D3ZAQ0 | Protein Fundc2 | 16.344 | 14.164 | 20.5 | 3 | 5 | 3 | 0.93 | 0.1% |
| O35854 | Branched-chain-amino-acid aminotransferase, mitochondrial | 44.275 | 107.88 | 22.4 | 6 | 7 | 6 | 0.93 | 1.4% |
| Q6QD51 | Coiled-coil domain-containing protein 80 | 107.69 | 43.034 | 8.6 | 8 | 11 | 8 | 0.93 | 5.2% |
| F1LT78 | Protein Antxr2 | 53.272 | 10.026 | 2.7 | 1 | 1 | 1 | 0.93 | 5.8% |
| F1MAK9 | Protein Smpd4 | 93.313 | 26.562 | 9.1 | 5 | 5 | 5 | 0.93 | 3.7% |
| Q6AY21 | GTPase activating protein (SH3 domain) binding protein 2 | 50.77 | 55.972 | 23.8 | 10 | 24 | 9 | 0.93 | 2.6% |
| M0R7G2 |  | 189.29 | 104.67 | 7.7 | 11 | 15 | 2 | 0.93 | 1.2% |
| P13084 | Nucleophosmin | 32.56 | 143.81 | 39.4 | 12 | 34 | 12 | 0.93 | 1.4% |
| P00787 | Cathepsin B | 37.47 | 136.95 | 42.2 | 12 | 51 | 12 | 0.93 | 1.8% |
| D4A0L1 | MON1 homolog b (Yeast) (Predicted), isoform CRA_b | 48.021 | 18.832 | 5.9 | 2 | 2 | 2 | 0.93 | 2.5% |
| A0A0G2JY73 | Protein Eif4g3 | 179.11 | 14.597 | 6.5 | 9 | 12 | 4 | 0.93 | 12.6% |
| D4AC93 | Protein Tmem223 | 21.673 | 2.1499 | 3.5 | 1 | 1 | 1 | 0.93 | 14.3% |
| P25235 | Dolichyl-diphosphooligosaccharide--protein glycosyltransferase subunit 2 | 69.077 | 294.75 | 33.1 | 18 | 42 | 18 | 0.93 | 0.4% |
| P97680 | Ras and Rab interactor 1 | 84.726 | 22.3 | 5.9 | 4 | 5 | 4 | 0.93 | 0.2% |
| Q5HZA6 | Prolyl endopeptidase-like | 83.481 | 29.741 | 6.9 | 4 | 4 | 4 | 0.93 | 2.1% |
| P61805 | Dolichyl-diphosphooligosaccharide--protein glycosyltransferase subunit DAD1 | 12.497 | 9.6378 | 19.5 | 2 | 4 | 2 | 0.93 | 1.2% |
| Q5U2X6 | Coiled-coil domain-containing protein 47 | 55.732 | 50.678 | 23.2 | 9 | 10 | 9 | 0.93 | 1.2% |
| B3DMA0 | Tumor protein p53-inducible protein 11 | 20.939 | 13.905 | 18 | 3 | 3 | 3 | 0.93 | 2.6% |
| Q5RKH1 | Serine/threonine-protein kinase PRP4 homolog | 117.01 | 4.5964 | 2.1 | 2 | 2 | 2 | 0.93 | 4.2% |
| Q7TNY6 | Golgi resident protein GCP60 | 60.478 | 63.921 | 22.2 | 10 | 15 | 10 | 0.93 | 6.1% |
| G3V6N2 | Protein Tmed4 | 26.117 | 10.007 | 16.3 | 3 | 9 | 2 | 0.93 | 0.8% |
| F1M155 | Protein Svil | 242.05 | 233.36 | 21.8 | 41 | 53 | 41 | 0.93 | 0.4% |
| Q6P6R2 | Dihydrolipoyl dehydrogenase, mitochondrial | 54.037 | 273.74 | 44.8 | 19 | 41 | 19 | 0.93 | 0.2% |
| P35952 | Low-density lipoprotein receptor | 96.621 | 24.122 | 7.8 | 6 | 7 | 6 | 0.93 | 0.3% |
| D3ZXP1 | Protein Ttc28 | 255.31 | 35.917 | 3.2 | 5 | 5 | 5 | 0.93 | 0.6% |
| Q64194 | Lysosomal acid lipase/cholesteryl ester hydrolase | 45.185 | 7.8881 | 5.8 | 2 | 4 | 2 | 0.93 | 13.1% |
| Q5FWU3 | Autophagy-related protein 9A | 94.487 | 5.9072 | 2.3 | 2 | 2 | 2 | 0.93 | 5.2% |
| Q08463 | Frizzled-1 | 71.027 | 17.287 | 6.4 | 4 | 4 | 4 | 0.93 | 7.0% |
| Q5M9G9 | Protein TBRG4 | 71.18 | 24.441 | 7 | 4 | 5 | 4 | 0.93 | 6.5% |
| P21396 | Amine oxidase [flavin-containing] A | 59.507 | 144.72 | 26.8 | 12 | 13 | 12 | 0.93 | 2.7% |
| Q63787 | Phosphatidylinositol 3-kinase regulatory subunit alpha | 83.53 | 5.0052 | 1.5 | 1 | 1 | 1 | 0.93 | 1.9% |
| Q562B5 | Serine/threonine-protein phosphatase PGAM5, mitochondrial | 32.06 | 24.016 | 24 | 6 | 10 | 6 | 0.93 | 1.8% |
| P70582 | Nuclear pore complex protein Nup54 | 55.744 | 39.401 | 10.6 | 4 | 6 | 4 | 0.93 | 0.6% |
| P04636 | Malate dehydrogenase, mitochondrial | 35.683 | 229.65 | 62.7 | 20 | 72 | 20 | 0.93 | 0.8% |
| Q5XIH3 | NADH dehydrogenase (Ubiquinone) flavoprotein 1 | 50.73 | 152.02 | 42 | 17 | 23 | 17 | 0.93 | 4.1% |
| Q9QW07 | 1-phosphatidylinositol 4,5-bisphosphate phosphodiesterase beta-4 | 134.5 | 113.29 | 17.5 | 19 | 25 | 19 | 0.93 | 4.7% |
| B2RYN3 | Eukaryotic translation elongation factor 1 epsilon 1 | 19.842 | 28.191 | 39.1 | 7 | 9 | 7 | 0.93 | 9.3% |
| Q8CG09 | Multidrug resistance-associated protein 1 | 171.49 | 46.353 | 10.2 | 14 | 15 | 13 | 0.93 | 3.0% |
| D3ZWA1 | Protein Fam63b | 65.696 | 2.6639 | 2 | 1 | 1 | 1 | 0.93 | 1.1% |
| A0A0G2K1M7 | Protein Ano6 | 105.84 | 47.928 | 12.5 | 10 | 13 | 10 | 0.93 | 0.4% |
| Q62826 | Heterogeneous nuclear ribonucleoprotein M | 73.782 | 202.07 | 41.3 | 27 | 43 | 27 | 0.93 | 0.9% |
| Q9WUD9 | Proto-oncogene tyrosine-protein kinase Src | 59.972 | 25.241 | 15.3 | 7 | 9 | 4 | 0.93 | 14.1% |
| G3V9C7 | Histone H2B | 13.89 | 56.313 | 58.7 | 9 | 60 | 9 | 0.93 | 0.9% |
| Q8VHE9 | All-trans-retinol 13,14-reductase | 67.53 | 29.771 | 12.5 | 7 | 11 | 7 | 0.93 | 0.4% |
| A0A0G2JX72 | Muscleblind-like protein 2 | 47.203 | 22.503 | 14.4 | 7 | 16 | 7 | 0.93 | 0.4% |
| Q63135 | Complement component receptor 1-like protein | 61.68 | 67.099 | 14.8 | 6 | 9 | 6 | 0.93 | 3.1% |
| Q99P63 | Calcium uptake protein 2, mitochondrial | 49.441 | 78.162 | 7.9 | 3 | 6 | 3 | 0.93 | 1.5% |
| P68136 | Actin, alpha skeletal muscle | 42.051 | 29.754 | 73.2 | 27 | 588 | 2 | 0.93 | 2.1% |
| P70567 | Tropomodulin-1 | 40.48 | 35.929 | 23.7 | 6 | 7 | 5 | 0.93 | 5.2% |
| M0R6K2 | Protein Erbb2ip | 158.02 | 29.298 | 5.4 | 6 | 7 | 6 | 0.93 | 0.3% |
| P51400 | Double-stranded RNA-specific editase 1 | 77.924 | 15.585 | 6 | 3 | 4 | 3 | 0.93 | 5.7% |
| Q5PPN7 | Coiled-coil domain-containing protein 51 | 45.813 | 9.4727 | 8.5 | 4 | 4 | 4 | 0.93 | 4.6% |
| Q6TUD4 | Protein YIPF3 | 37.973 | 4.3693 | 4.6 | 2 | 3 | 2 | 0.93 | 1.6% |
| Q62780 | Probable ATP-dependent RNA helicase DDX46 | 117.38 | 21.594 | 7.7 | 7 | 7 | 7 | 0.93 | 1.1% |
| D4A4P3 | Protein LOC100361144 | 11.267 | 6.8508 | 19.2 | 2 | 4 | 2 | 0.93 | 5.7% |
| Q704S8 | Carnitine O-acetyltransferase | 70.8 | 24.373 | 10.2 | 6 | 7 | 6 | 0.93 | 12.9% |
| D3ZM21 | Catechol-O-methyltransferase domain containing 1 (Predicted), isoform CRA_a | 28.987 | 51.015 | 20.6 | 3 | 4 | 3 | 0.93 | 5.4% |
| Q6AYE5 | Out at first protein homolog | 31.775 | 13.173 | 9.9 | 3 | 4 | 3 | 0.93 | 5.2% |
| E9PTC0 | Protein PVR | 45.537 | 13.965 | 10.9 | 3 | 3 | 3 | 0.93 | 3.7% |
| Q3ZU82 | Golgin subfamily A member 5 | 82.333 | 111.84 | 22.7 | 14 | 18 | 14 | 0.93 | 2.1% |
| F1LU48 | Protein Ergic1 | 35.011 | 50.482 | 30.4 | 8 | 19 | 8 | 0.93 | 0.7% |
| P62076 | Mitochondrial import inner membrane translocase subunit Tim13 | 10.458 | 34.532 | 51.6 | 5 | 8 | 5 | 0.93 | 0.6% |
| Q5BJZ3 | Nicotinamide nucleotide transhydrogenase | 113.87 | 305.61 | 32.3 | 35 | 60 | 35 | 0.93 | 0.3% |
| Q99068 | Alpha-2-macroglobulin receptor-associated protein | 42.031 | 80.281 | 46.7 | 18 | 24 | 18 | 0.93 | 0.3% |
| Q4V8C7 | Interferon-inducible double-stranded RNA-dependent protein kinase activator A | 34.355 | 43.889 | 22.4 | 6 | 9 | 6 | 0.93 | 0.7% |
| Q6AXR4 | Beta-hexosaminidase subunit beta | 61.527 | 69.466 | 19.2 | 12 | 19 | 12 | 0.93 | 1.2% |
| P25977 | Nucleolar transcription factor 1 | 89.436 | 34.616 | 7.7 | 5 | 5 | 5 | 0.93 | 1.5% |
| Q9Z0U5 | Aldehyde oxidase 1 | 146.92 | 37.549 | 2.9 | 3 | 3 | 2 | 0.93 | 2.9% |
| Q5BJP2 | Spliceosome-associated protein CWC15 homolog | 26.638 | 11.379 | 22.3 | 4 | 4 | 4 | 0.93 | 3.3% |
| D3ZNS8 | Achalasia, adrenocortical insufficiency, alacrimia (Allgrove, triple-A) (Predicted), isoform CRA_a | 59.36 | 5.3178 | 2.2 | 1 | 1 | 1 | 0.93 | 5.3% |
| D3ZQI1 | Glutathione peroxidase | 21.089 | 52.956 | 52.2 | 8 | 17 | 8 | 0.93 | 6.8% |
| Q8CHM7 | 2-hydroxyacyl-CoA lyase 1 | 63.615 | 11.842 | 5.3 | 3 | 3 | 3 | 0.93 | 3.2% |
| Q6P7D4 | Cytochrome P450 20A1 | 51.997 | 55.588 | 19.9 | 8 | 16 | 8 | 0.93 | 0.8% |
| P67779 | Prohibitin | 29.82 | 105.48 | 49.6 | 12 | 22 | 12 | 0.93 | 0.6% |
| Q5FVM4 | Non-POU domain-containing octamer-binding protein | 54.925 | 59.249 | 27.7 | 16 | 37 | 14 | 0.93 | 0.4% |
| Q9QZ86 | Nucleolar protein 58 | 60.07 | 75.088 | 23.6 | 9 | 14 | 9 | 0.93 | 0.2% |
| Q5XI78 | 2-oxoglutarate dehydrogenase, mitochondrial | 116.29 | 200.47 | 27.8 | 26 | 42 | 23 | 0.93 | 0.8% |
| Q5XIT9 | Methylcrotonoyl-CoA carboxylase beta chain, mitochondrial | 61.516 | 102.96 | 33.6 | 14 | 17 | 14 | 0.93 | 2.4% |
| F1LQH5 | Phospholipid-transporting ATPase | 128.22 | 11.511 | 3.5 | 3 | 3 | 3 | 0.93 | 3.0% |
| D3ZC56 | Protein Dst | 847.2 | 152.56 | 3.7 | 21 | 27 | 20 | 0.93 | 0.2% |
| Q6AY41 | Cell cycle control protein 50A | 37.172 | 6.2707 | 5.2 | 3 | 3 | 3 | 0.93 | 4.4% |
| E9PT59 | Protein Arhgef5 | 174.84 | 16.705 | 3.3 | 4 | 4 | 4 | 0.93 | 3.8% |
| D3ZBQ5 | Protein Scarf2 | 87.951 | 51.923 | 14 | 9 | 11 | 9 | 0.93 | 2.9% |
| D3ZXT4 | Protein Surf6 | 39.834 | 15.568 | 11 | 3 | 3 | 3 | 0.93 | 2.9% |
| Q6AYS4 | Plasma alpha-L-fucosidase | 53.257 | 6.2513 | 2.4 | 1 | 2 | 1 | 0.93 | 1.9% |
| A0A0G2K089 | Protein Dennd4c | 211.08 | 29.824 | 3.3 | 6 | 6 | 6 | 0.93 | 1.5% |
| Q99NA5 | Isocitrate dehydrogenase [NAD] subunit alpha, mitochondrial | 39.613 | 126.89 | 45.6 | 16 | 33 | 16 | 0.93 | 2.0% |
| Q6AXS9 | Protein FAM101B | 23.453 | 11.317 | 14.4 | 2 | 3 | 2 | 0.93 | 6.0% |
| Q5HZY2 | GTP-binding protein SAR1b | 22.41 | 46.585 | 35.4 | 7 | 12 | 7 | 0.93 | 0.2% |
| Q4V797 | Interferon-gamma-inducible GTPase Ifgga1 protein | 48.271 | 17.862 | 8.4 | 3 | 4 | 3 | 0.93 | 6.8% |
| P43278 | Histone H1.0 | 20.885 | 24.596 | 22.7 | 4 | 11 | 4 | 0.93 | 3.9% |
| P23965 | Enoyl-CoA delta isomerase 1, mitochondrial | 32.254 | 48.503 | 25.3 | 7 | 18 | 7 | 0.93 | 1.5% |
| A0A0G2K5Q8 | Protein Clk2 | 92.554 | 62.602 | 7.6 | 6 | 12 | 6 | 0.93 | 2.3% |
| D3ZZR9 | Peptidyl-prolyl cis-trans isomerase | 15.388 | 38.747 | 25.7 | 6 | 11 | 6 | 0.93 | 2.8% |
| D3ZVS2 | L-2-hydroxyglutarate dehydrogenase (Predicted) | 50.732 | 4.1107 | 3.2 | 1 | 1 | 1 | 0.93 | 6.6% |
| Q3KRC3 | Protein Srpr | 69.656 | 86.919 | 21.2 | 13 | 18 | 13 | 0.93 | 3.3% |
| Q562C2 | Ribosome biogenesis protein BOP1 | 82.639 | 8.5137 | 2.7 | 2 | 2 | 2 | 0.93 | 3.1% |
| P13596 | Neural cell adhesion molecule 1 | 94.657 | 213.43 | 37.1 | 27 | 54 | 27 | 0.93 | 0.3% |
| P35571 | Glycerol-3-phosphate dehydrogenase, mitochondrial | 80.972 | 59.882 | 17.5 | 12 | 17 | 12 | 0.93 | 0.3% |
| D3ZNI3 | Protein Pdcd11 | 209.48 | 68.844 | 6.7 | 10 | 10 | 10 | 0.93 | 0.2% |
| D4A4Z9 | Protein Ktn1 | 151.79 | 323.31 | 46.6 | 59 | 96 | 59 | 0.93 | 0.2% |
| A0A0G2K1E5 | Protein Sympk | 141.91 | 20.075 | 4.1 | 5 | 5 | 5 | 0.93 | 3.2% |
| Q5EBA1 | ATP-dependent RNA helicase SUPV3L1, mitochondrial | 86.705 | 11.791 | 6.7 | 4 | 4 | 4 | 0.93 | 4.7% |
| D4A1G8 | Protein Cep170b | 171.69 | 29.596 | 5 | 6 | 7 | 6 | 0.93 | 5.8% |
| D3ZAS8 | Protein Sart3 | 109.42 | 50.533 | 4.4 | 4 | 4 | 4 | 0.93 | 4.4% |
| Q62638 | Golgi apparatus protein 1 | 133.56 | 293.11 | 33.7 | 33 | 41 | 33 | 0.93 | 1.8% |
| B5DEQ4 | Protein Snrpb2 | 25.352 | 24.763 | 20 | 4 | 5 | 3 | 0.93 | 1.3% |
| D3Z9L0 | Protein Agk | 54.258 | 44.136 | 16.3 | 6 | 9 | 6 | 0.93 | 3.2% |
| A1A5P4 | Kinesin-like protein | 85.062 | 8.6994 | 3.6 | 2 | 2 | 2 | 0.93 | 5.4% |
| D3ZZL9 | GRIP and coiled-coil domain-containing protein 2 | 195.08 | 60.701 | 6.8 | 10 | 10 | 10 | 0.93 | 6.7% |
| D3ZEH2 | Protein Foxred1 | 54.229 | 16.406 | 7.4 | 3 | 4 | 3 | 0.92 | 0.6% |
| P24268 | Cathepsin D | 44.68 | 108.59 | 33.4 | 12 | 47 | 12 | 0.92 | 0.4% |
| Q6VBQ5 | Myeloid-associated differentiation marker | 35.148 | 53.166 | 15.4 | 5 | 22 | 5 | 0.92 | 0.9% |
| Q5NDF0 | Protein O-linked-mannose beta-1,4-N-acetylglucosaminyltransferase 2 | 66.716 | 3.2426 | 3.6 | 2 | 2 | 2 | 0.92 | 5.9% |
| G3V9M1 | DEAD (Asp-Glu-Ala-Asp) box polypeptide 23 (Predicted), isoform CRA_b | 95.495 | 38.087 | 10.3 | 8 | 10 | 8 | 0.92 | 4.9% |
| M0R548 | Pumilio domain-containing protein KIAA0020 homolog | 72.735 | 12.644 | 6.2 | 4 | 4 | 4 | 0.92 | 4.7% |
| Q99P75 | Ras-related protein Rab-9A | 22.895 | 25.906 | 32.8 | 6 | 8 | 6 | 0.92 | 4.5% |
| Q3B7U1 | Melanoma antigen, family D, 2 | 65.753 | 9.7767 | 6.1 | 4 | 4 | 4 | 0.92 | 4.4% |
| D3ZHW0 | Protein Dhx29 | 154.12 | 28.824 | 8.1 | 9 | 9 | 9 | 0.92 | 3.5% |
| P85125 | Polymerase I and transcript release factor | 43.908 | 173.4 | 38.5 | 14 | 35 | 14 | 0.92 | 2.7% |
| F1LZB0 | Protein R3hcc1 | 65.507 | 24.773 | 5.9 | 3 | 5 | 3 | 0.92 | 2.0% |
| A0A096MJW8 | Phosphoinositide phospholipase C | 183.71 | 2.961 | 0.5 | 1 | 6 | 1 | 0.92 | 1.6% |
| Q68FV3 | Alpha-(1,3)-fucosyltransferase 11 | 56 | 14.609 | 7.9 | 3 | 3 | 3 | 0.92 | 6.9% |
| F1LNF3 | Protein Nfxl1 | 101.92 | 20.372 | 9.5 | 7 | 7 | 7 | 0.92 | 0.5% |
| B1WC33 | CDC42 effector protein (Rho GTPase binding) 4 (Predicted), isoform CRA_a | 37.927 | 3.7221 | 4 | 1 | 1 | 1 | 0.92 | 1.2% |
| Q8VII6 | Choline transporter-like protein 1 | 73.091 | 30.643 | 9.5 | 5 | 7 | 5 | 0.92 | 3.2% |
| P17046 | Lysosome-associated membrane glycoprotein 2 | 45.163 | 11.845 | 8.5 | 4 | 8 | 4 | 0.92 | 2.5% |
| Q4KLL4 | Transmembrane 9 superfamily member 4 | 74.674 | 50.742 | 11.8 | 8 | 10 | 8 | 0.92 | 6.7% |
| Q4G063 | Cysteine-rich with EGF-like domain protein 2 | 38.25 | 41.958 | 32.1 | 8 | 10 | 8 | 0.92 | 4.0% |
| P01830 | Thy-1 membrane glycoprotein | 18.172 | 50.041 | 28.6 | 4 | 11 | 4 | 0.92 | 0.7% |
| Q5BJK8 | Golgi integral membrane protein 4 | 76.596 | 187.16 | 28.5 | 21 | 31 | 21 | 0.92 | 0.8% |
| Q66HG5 | Transmembrane 9 superfamily member 2 | 75.585 | 59.071 | 18.6 | 10 | 17 | 10 | 0.92 | 1.5% |
| F1LND1 | Protein Cog1 | 108.7 | 48.415 | 7.7 | 6 | 6 | 6 | 0.92 | 2.2% |
| Q5XID6 | Protein Sgcg | 32.18 | 47.154 | 14.4 | 3 | 3 | 3 | 0.92 | 9.4% |
| D3ZXF9 | Protein Mrpl12 | 29.441 | 29.837 | 20.7 | 3 | 5 | 3 | 0.92 | 0.1% |
| A0A0G2K9K2 | Protein Tacc1 | 85.692 | 20.653 | 9.3 | 8 | 8 | 5 | 0.92 | 1.2% |
| Q63042 | FAD-linked sulfhydryl oxidase ALR | 22.836 | 4.236 | 9.6 | 2 | 2 | 2 | 0.92 | 6.9% |
| Q5BJS2 | Lipoma HMGIC fusion partner | 21.666 | 2.6177 | 6.5 | 1 | 1 | 1 | 0.92 | 8.2% |
| M0R6B9 | Protein Cstf2t | 65.515 | 12.022 | 5.7 | 4 | 4 | 2 | 0.92 | 9.3% |
| Q68G38 | Torsin-1A | 37.949 | 2.4813 | 3.3 | 1 | 1 | 1 | 0.92 | 1.4% |
| Q5FVJ3 | Probable lipid phosphate phosphatase PPAPDC3 | 29.768 | 2.3825 | 6.3 | 1 | 1 | 1 | 0.92 | 1.2% |
| D3ZJX5 | Protein Timm50 | 39.854 | 24.97 | 18.7 | 7 | 9 | 7 | 0.92 | 0.2% |
| D3ZIN5 | Protein Vwa8 | 150.28 | 32.827 | 5.8 | 7 | 8 | 7 | 0.92 | 0.5% |
| O08587 | Nuclear pore complex protein Nup50 | 49.817 | 46.841 | 21.4 | 7 | 8 | 7 | 0.92 | 1.1% |
| A1A5P2 | Ribosome biogenesis regulatory protein homolog | 41.536 | 13.724 | 10.4 | 3 | 3 | 3 | 0.92 | 1.7% |
| F1LTZ2 | Protein Sepn1 | 53.435 | 17.194 | 6.4 | 3 | 4 | 3 | 0.92 | 1.9% |
| Q6IMK2 | Protein Sp7 | 44.649 | 13.609 | 4 | 1 | 2 | 1 | 0.92 | 2.4% |
| P11960 | 2-oxoisovalerate dehydrogenase subunit alpha, mitochondrial | 50.164 | 33.015 | 20 | 7 | 8 | 7 | 0.92 | 6.6% |
| Q4FZT0 | Stomatin-like protein 2, mitochondrial | 38.413 | 192.75 | 42.8 | 12 | 19 | 12 | 0.92 | 1.4% |
| P13832 | Myosin regulatory light chain RLC-A | 19.895 | 79.545 | 66.3 | 13 | 78 | 3 | 0.92 | 0.7% |
| A0A0G2K3L9 | Protein Ppfia1 | 137.98 | 73.823 | 10.7 | 10 | 14 | 10 | 0.92 | 2.4% |
| D3ZMQ3 | Protein Itga4 | 115.59 | 10.856 | 2.1 | 2 | 2 | 2 | 0.92 | 4.2% |
| P19511 | ATP synthase F(0) complex subunit B1, mitochondrial | 28.868 | 53.087 | 31.2 | 10 | 19 | 10 | 0.92 | 6.5% |
| D3ZTX0 | Transmembrane emp24 domain-containing protein 7 | 25.479 | 42.542 | 28.8 | 6 | 9 | 6 | 0.92 | 2.4% |
| Q6AYI1 | DEAD (Asp-Glu-Ala-Asp) box polypeptide 5 | 69.238 | 142.26 | 47 | 29 | 47 | 21 | 0.92 | 1.7% |
| Q9QZA6 | CD151 antigen | 28.355 | 9.9219 | 10.3 | 3 | 4 | 3 | 0.92 | 1.4% |
| B0K020 | CDGSH iron-sulfur domain-containing protein 1 | 12.097 | 39.388 | 44.4 | 5 | 12 | 5 | 0.92 | 0.1% |
| Q6Q7Y5 | Guanine nucleotide-binding protein subunit alpha-13 | 44.011 | 56.415 | 22.5 | 8 | 10 | 8 | 0.92 | 0.1% |
| D3ZJ92 | Pre-mRNA processing factor 40 homolog A (Yeast) (Predicted) | 108.47 | 49.26 | 7.2 | 4 | 5 | 4 | 0.92 | 1.8% |
| B2RZD6 | Ndufa4 protein | 9.3267 | 29.257 | 43.9 | 6 | 12 | 6 | 0.92 | 3.1% |
| Q80ZG1 | Synembryn-A | 59.832 | 14.974 | 11.7 | 5 | 5 | 5 | 0.92 | 4.3% |
| D3ZQM0 | Protein Sf3a1 | 88.587 | 62.898 | 16.2 | 13 | 15 | 13 | 0.92 | 7.2% |
| A0A0G2K2A5 | Protein Uchl5 | 38.845 | 12.89 | 16.2 | 4 | 4 | 4 | 0.92 | 8.8% |
| P51145 | Fos-related antigen 2 | 35.255 | 2.1506 | 3.7 | 1 | 1 | 1 | 0.92 | 9.9% |
| A0A0G2JY48 | Protein Ephb2 | 109.89 | 6.7093 | 1.9 | 2 | 2 | 2 | 0.92 | 3.1% |
| Q62698 | Cytoplasmic dynein 1 light intermediate chain 2 | 54.744 | 108.26 | 25.4 | 9 | 16 | 9 | 0.92 | 2.9% |
| Q7TP98 | Interleukin enhancer-binding factor 2 | 51.38 | 32.878 | 8.2 | 3 | 3 | 3 | 0.92 | 2.3% |
| D3ZWU9 | Protein Rmnd5a | 43.992 | 23.068 | 6.4 | 2 | 3 | 2 | 0.92 | 1.7% |
| P02466 | Collagen alpha-2(I) chain | 129.56 | 323.31 | 52.3 | 52 | 92 | 52 | 0.92 | 0.8% |
| Q9Z1X1 | Extended synaptotagmin-1 | 121.16 | 323.31 | 39.5 | 31 | 58 | 31 | 0.92 | 0.5% |
| Q5XHZ0 | Heat shock protein 75 kDa, mitochondrial | 80.46 | 144.6 | 34.4 | 20 | 25 | 20 | 0.92 | 0.5% |
| Q924C3 | Ectonucleotide pyrophosphatase/phosphodiesterase family member 1 | 102.94 | 306.95 | 35.5 | 25 | 47 | 25 | 0.92 | 3.7% |
| Q642A4 | UPF0598 protein C8orf82 homolog | 24.397 | 16.057 | 17.4 | 3 | 6 | 3 | 0.92 | 9.3% |
| D3ZTM7 | Protein Efcab14 | 53.477 | 29.679 | 9.5 | 4 | 5 | 4 | 0.92 | 0.9% |
| O35094 | Mitochondrial import inner membrane translocase subunit TIM44 | 51.06 | 87.775 | 26.5 | 11 | 17 | 11 | 0.92 | 0.9% |
| D3ZYS7 | Protein G3bp1 | 51.786 | 72.914 | 25.2 | 9 | 20 | 8 | 0.92 | 0.2% |
| P97615 | Thioredoxin, mitochondrial | 18.232 | 55.222 | 21.7 | 2 | 4 | 2 | 0.92 | 3.2% |
| P20695 | Interferon-related developmental regulator 1 | 49.782 | 18.153 | 3.8 | 1 | 3 | 1 | 0.92 | 3.0% |
| P35565 | Calnexin | 67.254 | 173.11 | 37.2 | 26 | 52 | 26 | 0.92 | 2.0% |
| Q9WTT2 | Caseinolytic peptidase B protein homolog | 75.701 | 14.414 | 9.3 | 5 | 5 | 5 | 0.92 | 2.1% |
| Q08851 | Syntaxin-5 | 39.75 | 14.612 | 7.9 | 3 | 6 | 3 | 0.92 | 2.8% |
| A0A0G2K948 | Protein Dpf2 | 45.84 | 10.791 | 6.9 | 2 | 3 | 2 | 0.92 | 7.0% |
| Q4KM65 | Cleavage and polyadenylation specificity factor subunit 5 | 26.24 | 26.726 | 30.8 | 6 | 9 | 6 | 0.92 | 0.8% |
| B1WC25 | Protein Tra2a | 32.578 | 23.654 | 21.3 | 5 | 5 | 4 | 0.92 | 0.3% |
| A0A0G2K528 | Protein Stx16 | 35.441 | 45.111 | 20.7 | 5 | 7 | 5 | 0.92 | 2.9% |
| P98166 | Very low-density lipoprotein receptor | 96.541 | 6.4492 | 1.9 | 2 | 2 | 2 | 0.92 | 2.3% |
| P18437 | Non-histone chromosomal protein HMG-17 | 9.3654 | 15.245 | 44.4 | 4 | 4 | 4 | 0.92 | 8.2% |
| A0A0G2K782 | Protein Zc2hc1a | 35.219 | 19.347 | 18.5 | 8 | 12 | 8 | 0.92 | 3.1% |
| Q32PX6 | Protein Rhog | 21.308 | 73.233 | 39.8 | 6 | 12 | 5 | 0.92 | 0.1% |
| Q62599 | Metastasis-associated protein MTA1 | 79.411 | 36.5 | 14.1 | 9 | 11 | 7 | 0.92 | 3.0% |
| F1M3J4 | Protein Abcc4 | 148.9 | 15.618 | 3.3 | 4 | 4 | 3 | 0.92 | 3.2% |
| Q6P6S4 | Nucleotide exchange factor SIL1 | 52.349 | 58.396 | 16.8 | 6 | 9 | 6 | 0.92 | 3.3% |
| P16636 | Protein-lysine 6-oxidase | 46.558 | 5.4363 | 7.5 | 2 | 3 | 2 | 0.92 | 4.6% |
| D4A111 | Protein Col6a3 | 306.15 | 79.654 | 5.4 | 12 | 14 | 12 | 0.92 | 4.8% |
| Q64017 | Probable G-protein coupled receptor 176 | 56.784 | 2.014 | 1.4 | 1 | 1 | 1 | 0.92 | 6.4% |
| Q6TUD3 | LRRGT00111 | 44.779 | 13.729 | 7.9 | 3 | 4 | 3 | 0.92 | 5.7% |
| Q27W01 | RNA-binding protein 8A | 19.889 | 13.12 | 21.3 | 4 | 5 | 4 | 0.92 | 5.4% |
| O54861 | Sortilin | 91.168 | 15.33 | 4.6 | 3 | 3 | 3 | 0.92 | 2.7% |
| D3ZZG3 | Protein LOC691921 | 7.6048 | 2.2597 | 10.4 | 1 | 1 | 1 | 0.92 | 1.5% |
| A0A0G2JUJ9 | Protein Sec63 | 87.716 | 96.187 | 16.8 | 10 | 12 | 10 | 0.92 | 0.1% |
| Q04970 | GTPase NRas | 21.243 | 21.646 | 34.4 | 5 | 13 | 1 | 0.92 | 0.2% |
| Q62753 | Syntaxin-binding protein 2 | 66.695 | 34.96 | 12 | 6 | 6 | 5 | 0.92 | 3.4% |
| F1LN63 | Protein Tlr3 | 103.01 | 8.5317 | 4 | 3 | 3 | 3 | 0.92 | 9.8% |
| Q6P7S1 | Acid ceramidase | 44.443 | 131.93 | 46.2 | 19 | 35 | 19 | 0.92 | 1.4% |
| G3V6N5 | PQ loop repeat containing 3 | 22.767 | 7.3496 | 9.9 | 2 | 2 | 2 | 0.92 | 9.7% |
| D3ZL57 | Protein Usp6nl | 93.275 | 2.029 | 1.6 | 1 | 1 | 1 | 0.92 | 4.4% |
| B2GV96 | Coiled-coil domain containing 115 | 19.814 | 33.934 | 37.8 | 6 | 8 | 6 | 0.92 | 2.6% |
| Q8CGU6 | Nicastrin | 78.399 | 26.294 | 7.9 | 5 | 7 | 5 | 0.92 | 3.5% |
| Q6P7B4 | Protein FAM198B | 58.205 | 138.88 | 31.6 | 13 | 19 | 13 | 0.92 | 1.4% |
| O70377 | Synaptosomal-associated protein 23 | 23.235 | 88.249 | 37.1 | 6 | 9 | 6 | 0.92 | 0.2% |
| Q4QQV2 | Docking protein 1 | 52.169 | 36.386 | 16.5 | 4 | 4 | 4 | 0.92 | 5.7% |
| Q62896 | BET1 homolog | 13.23 | 34.92 | 24.6 | 2 | 2 | 2 | 0.92 | 4.6% |
| F1LQ00 | Protein Col5a2 | 145.2 | 196.29 | 22.6 | 24 | 38 | 23 | 0.92 | 4.1% |
| P42533 | Tissue factor | 33.443 | 14.186 | 9.8 | 3 | 5 | 3 | 0.92 | 5.0% |
| Q1HAQ0 | Lysophosphatidylcholine acyltransferase 1 | 59.761 | 17.129 | 6.6 | 3 | 3 | 3 | 0.92 | 5.2% |
| B1H282 | Glycosyltransferase 25 domain containing 1 | 71.114 | 41.12 | 19.1 | 11 | 13 | 11 | 0.92 | 4.3% |
| P13437 | 3-ketoacyl-CoA thiolase, mitochondrial | 41.87 | 226.51 | 52.1 | 14 | 32 | 14 | 0.92 | 1.7% |
| Q63750 | 39S ribosomal protein L23, mitochondrial | 17.05 | 9.1698 | 10.3 | 1 | 2 | 1 | 0.92 | 4.6% |
| Q5FVH2 | Phospholipase D3 | 54.399 | 18.535 | 10.2 | 5 | 6 | 5 | 0.92 | 8.6% |
| G3V7N0 | Heparan sulfate 2-O-sulfotransferase 1, isoform CRA_a | 41.851 | 31.615 | 19.9 | 6 | 7 | 6 | 0.92 | 2.1% |
| Q3B8Q1 | Nucleolar RNA helicase 2 | 85.965 | 53.676 | 16 | 9 | 11 | 9 | 0.92 | 0.4% |
| P20788 | Cytochrome b-c1 complex subunit Rieske, mitochondrial | 29.445 | 104.82 | 29.6 | 8 | 14 | 8 | 0.92 | 0.7% |
| Q5XI04 | Protein Stom | 31.378 | 37.659 | 29.2 | 6 | 7 | 6 | 0.92 | 2.4% |
| D3ZAS1 | Protein RGD1562399 | 30.871 | 17.91 | 18.9 | 5 | 13 | 1 | 0.92 | 3.5% |
| M0R835 | Protein Sf3b6 | 14.328 | 10.485 | 9.8 | 1 | 2 | 1 | 0.92 | 2.0% |
| P00406 | Cytochrome c oxidase subunit 2 | 25.928 | 39.971 | 23.8 | 5 | 13 | 5 | 0.92 | 5.5% |
| Q9WUC4 | Copper transport protein ATOX1 | 7.2924 | 27.904 | 57.4 | 3 | 5 | 3 | 0.92 | 2.0% |
| B2GV06 | Succinyl-CoA:3-ketoacid coenzyme A transferase 1, mitochondrial | 56.203 | 196.03 | 41.9 | 16 | 36 | 16 | 0.92 | 1.1% |
| P27867 | Sorbitol dehydrogenase | 38.234 | 32.069 | 12.6 | 3 | 3 | 3 | 0.92 | 12.7% |
| O08623 | Sequestosome-1 | 47.681 | 44.421 | 12.1 | 3 | 4 | 3 | 0.92 | 10.0% |
| P20069 | Mitochondrial-processing peptidase subunit alpha | 58.607 | 72.449 | 26.5 | 13 | 15 | 13 | 0.92 | 5.6% |
| Q64380 | Sarcosine dehydrogenase, mitochondrial | 101.44 | 35.864 | 11.2 | 10 | 12 | 10 | 0.92 | 3.5% |
| P86252 | Transcriptional activator protein Pur-alpha | 15.322 | 67.471 | 47.1 | 4 | 10 | 4 | 0.92 | 3.6% |
| P53565 | Homeobox protein cut-like 1 | 164.54 | 65.621 | 9.8 | 12 | 12 | 12 | 0.92 | 7.0% |
| Q7TMD5 | Zinc finger CCCH domain-containing protein 14 | 82.631 | 4.597 | 1.4 | 1 | 1 | 1 | 0.92 | 8.0% |
| Q08850 | Syntaxin-4 | 34.209 | 39.239 | 13.1 | 3 | 4 | 3 | 0.92 | 3.5% |
| Q5PQP2 | Receptor-binding cancer antigen expressed on SiSo cells | 24.187 | 16.339 | 14.6 | 2 | 3 | 2 | 0.92 | 0.7% |
| Q63355 | Unconventional myosin-Ic | 119.81 | 323.31 | 48.3 | 47 | 97 | 47 | 0.92 | 0.2% |
| P28494 | Alpha-mannosidase 2 | 131.24 | 151.7 | 23.8 | 24 | 31 | 24 | 0.92 | 0.4% |
| Q75Q39 | Mitochondrial import receptor subunit TOM70 | 67.444 | 91.127 | 37.2 | 20 | 27 | 20 | 0.92 | 1.9% |
| P52481 | Adenylyl cyclase-associated protein 2 | 52.912 | 28.091 | 16.6 | 7 | 9 | 6 | 0.92 | 2.2% |
| D3ZRG7 | Protein Rbm26 | 113.54 | 13.245 | 2.2 | 2 | 2 | 2 | 0.92 | 2.3% |
| Q920Q0 | Paralemmin-1 | 41.926 | 22.914 | 13.6 | 6 | 8 | 4 | 0.92 | 5.7% |
| O88563 | Canalicular multispecific organic anion transporter 2 | 168.98 | 9.1517 | 2.4 | 4 | 4 | 3 | 0.92 | 6.1% |
| B0BNB9 | HtrA serine peptidase 2 | 49.094 | 39.878 | 14.6 | 5 | 7 | 5 | 0.92 | 0.2% |
| D3ZUB0 | Protein Rcn1 | 38.09 | 245.17 | 55.4 | 21 | 56 | 21 | 0.92 | 0.5% |
| Q9JI03 | Collagen alpha-1(V) chain | 183.98 | 241.72 | 21.4 | 31 | 54 | 25 | 0.92 | 1.5% |
| P39948 | G1/S-specific cyclin-D1 | 33.483 | 2.5077 | 6.8 | 2 | 2 | 2 | 0.92 | 2.1% |
| Q5U2U0 | ATP-dependent Clp protease ATP-binding subunit clpX-like, mitochondrial | 69.207 | 63.982 | 15 | 8 | 11 | 8 | 0.92 | 2.5% |
| G3V7P1 | Syntaxin-12 | 31.187 | 62.508 | 27.7 | 5 | 10 | 5 | 0.92 | 3.7% |
| Q498E0 | Thioredoxin domain-containing protein 12 | 19.018 | 53.484 | 49.4 | 8 | 21 | 8 | 0.92 | 4.3% |
| D3ZT98 | Protein Bola3 | 12.297 | 18.414 | 30.9 | 3 | 4 | 3 | 0.92 | 4.6% |
| A0A0G2K1W1 | Protein Rab11fip5 | 123.81 | 120.51 | 15.1 | 11 | 12 | 11 | 0.92 | 6.7% |
| D4A563 | Protein Peak1 | 191.11 | 143.97 | 13.2 | 17 | 22 | 17 | 0.91 | 0.7% |
| F1MAA7 | Protein Lamc1 | 177.38 | 25.977 | 4.5 | 6 | 7 | 6 | 0.91 | 0.2% |
| P10860 | Glutamate dehydrogenase 1, mitochondrial | 61.415 | 311.08 | 46.8 | 26 | 70 | 26 | 0.91 | 1.5% |
| D3Z9M5 | Protein Fkbp7 | 24.818 | 48.119 | 46.3 | 11 | 16 | 11 | 0.91 | 1.8% |
| A0A0G2K781 | Protein Ppfibp1 | 116.1 | 221.9 | 26 | 19 | 24 | 19 | 0.91 | 1.4% |
| P10824 | Guanine nucleotide-binding protein G(i) subunit alpha-1 | 40.345 | 7.1564 | 30.5 | 10 | 20 | 2 | 0.91 | 4.9% |
| D4A4H5 | Protein Sdf2 | 24.056 | 68.584 | 37.9 | 7 | 14 | 7 | 0.91 | 6.0% |
| D3ZF11 | Hepatitis B virus x interacting protein (Predicted), isoform CRA_a | 16.39 | 90.594 | 47.4 | 4 | 9 | 4 | 0.91 | 6.8% |
| G3V928 | Protein Lrp1 | 504.88 | 323.31 | 17.8 | 69 | 92 | 69 | 0.91 | 1.4% |
| Q4V899 | Transmembrane protein 165 | 34.809 | 42.919 | 21.4 | 4 | 5 | 4 | 0.91 | 2.7% |
| Q9Z142 | Transmembrane protein 33 | 27.983 | 14.012 | 13 | 3 | 4 | 3 | 0.91 | 5.4% |
| G3V7I3 | ATPase type 13A1 (Predicted), isoform CRA_a | 131.91 | 149.75 | 21.6 | 21 | 28 | 21 | 0.91 | 4.6% |
| P25809 | Creatine kinase U-type, mitochondrial | 47.028 | 73.479 | 21.8 | 7 | 13 | 7 | 0.91 | 1.8% |
| Q8VIG2 | Meiosis arrest female protein 1 | 192.64 | 9.9942 | 0.7 | 1 | 1 | 1 | 0.91 | 0.2% |
| P0C5I0 | Cerebral dopamine neurotrophic factor | 21.361 | 4.7271 | 5.9 | 1 | 1 | 1 | 0.91 | 0.2% |
| P31016 | Disks large homolog 4 | 80.464 | 7.1454 | 2.6 | 2 | 2 | 1 | 0.91 | 1.6% |
| Q5U2W5 | Transducin beta-like protein 3 | 88.371 | 5.4523 | 2.5 | 2 | 2 | 2 | 0.91 | 3.4% |
| D4A900 | Protein Filip1l | 135.84 | 69.225 | 14.9 | 17 | 19 | 17 | 0.91 | 0.4% |
| A0A0G2JU15 | Protein Ptcd3 | 84.556 | 26.854 | 7.5 | 5 | 6 | 5 | 0.91 | 0.4% |
| P26453 | Basigin | 42.435 | 37.017 | 11.9 | 5 | 8 | 5 | 0.91 | 1.4% |
| O35413 | Sorbin and SH3 domain-containing protein 2 | 134.06 | 153.47 | 20.2 | 26 | 46 | 25 | 0.91 | 2.5% |
| P56603 | Secretory carrier-associated membrane protein 1 | 37.998 | 33.611 | 18.6 | 7 | 11 | 7 | 0.91 | 1.8% |
| D3ZUL3 | Protein Col6a1 | 108.8 | 54.9 | 13.9 | 12 | 13 | 12 | 0.91 | 0.8% |
| Q63635 | Syntaxin-6 | 29.056 | 26.701 | 20 | 4 | 4 | 4 | 0.91 | 0.5% |
| D3ZZ45 |  | 12.705 | 3.7218 | 7.3 | 2 | 4 | 2 | 0.91 | 1.2% |
| F1LVY9 | Protein Pdf | 25.879 | 7.936 | 9.1 | 2 | 2 | 2 | 0.91 | 9.0% |
| D3Z937 | High mobility group 20A (Predicted) | 39.902 | 22.89 | 4.9 | 1 | 1 | 1 | 0.91 | 7.1% |
| D4A3I5 | LOC361774 (Predicted) | 86.211 | 18.784 | 4.8 | 3 | 4 | 3 | 0.91 | 4.5% |
| Q9JK11 | Reticulon-4 | 126.39 | 119.68 | 12 | 12 | 29 | 12 | 0.91 | 3.7% |
| Q499S9 | Inactive rhomboid protein 1 | 97.261 | 15.527 | 4.8 | 3 | 4 | 3 | 0.91 | 4.4% |
| A0A0G2K9Y9 | Zinc finger protein 326 | 57.056 | 41.795 | 5.6 | 2 | 2 | 2 | 0.91 | 5.2% |
| Q5I0P2 | Glycine cleavage system H protein, mitochondrial | 18.485 | 42.058 | 24.1 | 4 | 6 | 4 | 0.91 | 3.1% |
| P26772 | 10 kDa heat shock protein, mitochondrial | 10.902 | 44.207 | 75.5 | 9 | 25 | 9 | 0.91 | 1.3% |
| Q6XFR6 | Glycophorin-C | 10.443 | 38.875 | 27.4 | 1 | 1 | 1 | 0.91 | 5.3% |
| Q5FVC4 | DnaJ (Hsp40) homolog, subfamily B, member 12 | 42.521 | 20.091 | 8.7 | 3 | 5 | 3 | 0.91 | 8.0% |
| Q5XIE6 | 3-hydroxyisobutyryl-CoA hydrolase, mitochondrial | 43.024 | 92.108 | 24.4 | 8 | 9 | 8 | 0.91 | 1.0% |
| F1MAH6 | Protein Cdh11 | 88.035 | 36.036 | 6.9 | 4 | 6 | 4 | 0.91 | 0.1% |
| P70584 | Short/branched chain specific acyl-CoA dehydrogenase, mitochondrial | 47.823 | 132.67 | 32.9 | 9 | 12 | 9 | 0.91 | 0.9% |
| Q75Q40 | Mitochondrial import receptor subunit TOM40 homolog | 37.919 | 47.946 | 25.2 | 7 | 10 | 7 | 0.91 | 1.4% |
| F1M9B2 | Insulin-like growth factor binding protein 7, isoform CRA_b | 28.949 | 12.626 | 14.2 | 3 | 5 | 3 | 0.91 | 2.4% |
| D3ZZU4 | Protein Tmem160 | 19.587 | 6.0032 | 6.9 | 1 | 1 | 1 | 0.91 | 1.4% |
| Q5HZE6 | Protein Rnd2 | 25.448 | 2.4263 | 3.1 | 1 | 1 | 1 | 0.91 | 1.3% |
| P13803 | Electron transfer flavoprotein subunit alpha, mitochondrial | 34.951 | 146.87 | 46.5 | 13 | 39 | 13 | 0.91 | 0.7% |
| Q62839 | Golgin subfamily A member 2 | 112.84 | 148.29 | 23.4 | 20 | 27 | 20 | 0.91 | 0.4% |
| Q63377 | Sodium/potassium-transporting ATPase subunit beta-3 | 31.829 | 58.998 | 27.6 | 6 | 12 | 6 | 0.91 | 1.7% |
| Q6MG48 | Protein PRRC2A | 229.04 | 61.075 | 6.8 | 11 | 11 | 10 | 0.91 | 4.7% |
| Q9Z1W6 | Protein LYRIC | 63.968 | 143.1 | 35.3 | 15 | 21 | 15 | 0.91 | 3.0% |
| F1M067 | Protein Slc26a11 | 64.382 | 14.753 | 2.2 | 1 | 2 | 1 | 0.91 | 5.5% |
| A0A0G2K302 | Protein Ap3s2 | 20.406 | 2.2245 | 6.1 | 1 | 1 | 1 | 0.91 | 1.0% |
| D4A0T8 | Protein Dhrs7 | 38.493 | 54.407 | 22.9 | 7 | 10 | 7 | 0.91 | 0.1% |
| D4A6D7 | Tetratricopeptide repeat protein 19, mitochondrial | 41.333 | 5.2484 | 3 | 1 | 1 | 1 | 0.91 | 1.1% |
| A0A0G2JV24 | Protein Thbs1 | 127.87 | 290.86 | 29.6 | 38 | 73 | 17 | 0.91 | 1.3% |
| F1LU71 | AU RNA binding protein/enoyl-coenzyme A hydratase (Predicted), isoform CRA_a | 33.341 | 71.942 | 16.8 | 5 | 7 | 5 | 0.91 | 1.8% |
| Q5XJW2 | Growth arrest and DNA damage-inducible proteins-interacting protein 1 | 26.467 | 15.078 | 19.7 | 4 | 5 | 4 | 0.91 | 2.6% |
| O08776 | NADH dehydrogenase [ubiquinone] 1 alpha subcomplex assembly factor 3 | 20.696 | 6.3503 | 5.9 | 1 | 1 | 1 | 0.91 | 5.8% |
| P81377 | cAMP-dependent protein kinase type I-beta regulatory subunit | 43.282 | 18.24 | 12.3 | 4 | 5 | 1 | 0.91 | 4.0% |
| Q5RK09 | Eukaryotic translation initiation factor 3 subunit G | 35.651 | 59.227 | 38.1 | 10 | 12 | 10 | 0.91 | 4.0% |
| D4A576 | Protein Abcd4 | 68.685 | 24.861 | 6.9 | 3 | 5 | 3 | 0.91 | 15.4% |
| A0A0G2JYD3 | Protein Uggt2 | 165.38 | 103.31 | 14.5 | 18 | 22 | 17 | 0.91 | 2.3% |
| Q62786 | Prostaglandin F2 receptor negative regulator | 98.73 | 68.832 | 16.7 | 14 | 16 | 14 | 0.91 | 1.6% |
| Q4V882 | Epsin-3 | 65.047 | 6.4722 | 3.6 | 2 | 2 | 1 | 0.91 | 7.1% |
| Q07258 | Transforming growth factor beta-3 | 47.115 | 103.56 | 30.1 | 12 | 27 | 12 | 0.91 | 3.1% |
| Q8VI60 | Extracellular sulfatase Sulf-1 | 100.86 | 5.0286 | 2.3 | 2 | 2 | 2 | 0.91 | 2.9% |
| A0A0G2K0D3 | Protein Lmod1 | 66.215 | 109.38 | 31.3 | 23 | 38 | 23 | 0.91 | 1.7% |
| D3Z9E6 | Cleavage and polyadenylation specific factor 2 (Predicted) | 88.35 | 11.586 | 4.7 | 3 | 4 | 3 | 0.91 | 0.2% |
| A0A0G2K1V7 | Protein Sgcb | 34.959 | 77.75 | 15 | 3 | 3 | 3 | 0.91 | 3.7% |
| P13383 | Nucleolin | 77.146 | 263.8 | 42.6 | 35 | 62 | 35 | 0.91 | 0.4% |
| F1LVX1 | Protein Dnajc1 | 65.36 | 57.362 | 21.6 | 12 | 17 | 12 | 0.91 | 0.2% |
| Q6AYK1 | RNA-binding protein with serine-rich domain 1 | 34.238 | 19.043 | 4.9 | 1 | 2 | 1 | 0.91 | 8.4% |
| D3ZNY8 | Tyrosine-protein kinase transmembrane receptor | 105.02 | 17.89 | 3.9 | 3 | 3 | 2 | 0.91 | 5.6% |
| F7EPZ4 | Protein Dis3 | 116.78 | 11.6 | 2.8 | 3 | 4 | 3 | 0.91 | 5.9% |
| A0A0G2QC38 | Protein Srsf11 | 56.66 | 19.276 | 9.4 | 4 | 4 | 4 | 0.91 | 9.4% |
| P49797 | Regulator of G-protein signaling 3 | 106.38 | 9.5033 | 2.3 | 2 | 3 | 2 | 0.91 | 7.2% |
| Q63942 | GTP-binding protein Rab-3D | 24.29 | 19.043 | 10.5 | 2 | 3 | 1 | 0.91 | 3.6% |
| Q5XIF3 | NADH dehydrogenase [ubiquinone] iron-sulfur protein 4, mitochondrial | 19.74 | 14.152 | 22.9 | 4 | 5 | 4 | 0.91 | 1.8% |
| Q4QQW8 | Putative phospholipase B-like 2 | 65.455 | 114.29 | 23.9 | 12 | 18 | 12 | 0.91 | 1.4% |
| Q9JI85 | Nucleobindin-2 | 50.09 | 240.97 | 58.6 | 20 | 42 | 20 | 0.91 | 1.2% |
| Q07803 | Elongation factor G, mitochondrial | 83.456 | 38.068 | 8.8 | 6 | 6 | 6 | 0.91 | 2.0% |
| Q62718 | Neurotrimin | 37.998 | 19.21 | 5.5 | 1 | 1 | 1 | 0.91 | 3.5% |
| B2RYU8 | LYR motif-containing protein 1 | 14.258 | 4.8002 | 14.8 | 2 | 2 | 2 | 0.91 | 1.3% |
| Q925S8 | ATP-dependent zinc metalloprotease YME1L1 | 79.866 | 19.459 | 8.4 | 5 | 6 | 5 | 0.91 | 1.3% |
| Q3KR55 | Splicing factor U2AF 26 kDa subunit | 27.815 | 17.368 | 18.8 | 4 | 5 | 4 | 0.91 | 4.5% |
| Q9ESH6 | Glutaredoxin-1 | 11.879 | 26.151 | 36.4 | 3 | 3 | 3 | 0.91 | 5.6% |
| A0A0G2K0Y2 | Insulin-like growth factor 2, binding protein 3 | 63.593 | 77.978 | 22.1 | 11 | 16 | 10 | 0.91 | 5.8% |
| Q9Z269 | Vesicle-associated membrane protein-associated protein B | 26.916 | 72.598 | 36.2 | 9 | 15 | 8 | 0.91 | 2.6% |
| Q64375 | Synaptonemal complex protein SC65 | 47.674 | 46.103 | 18.6 | 7 | 8 | 7 | 0.91 | 0.2% |
| P00507 | Aspartate aminotransferase, mitochondrial | 47.314 | 165.62 | 50 | 21 | 41 | 21 | 0.91 | 0.2% |
| Q80WE1 | Fragile X mental retardation protein 1 homolog | 66.78 | 54.878 | 16.9 | 9 | 13 | 6 | 0.91 | 6.4% |
| Q5XIF5 | Sphingolipid delta(4)-desaturase DES1 | 38.055 | 28.9 | 11.8 | 3 | 3 | 3 | 0.91 | 1.3% |
| P49134 | Integrin beta-1 | 88.494 | 271.08 | 29.8 | 23 | 55 | 23 | 0.91 | 0.2% |
| Q9QYW0 | Protein AATF | 59.496 | 24.024 | 5.2 | 2 | 3 | 2 | 0.91 | 7.9% |
| Q6AY57 | WD repeat domain phosphoinositide-interacting protein 2 | 48.519 | 8.9324 | 5.8 | 3 | 3 | 3 | 0.91 | 4.8% |
| E9PTA4 | Protein Adam9 | 92.367 | 39.334 | 10.1 | 6 | 6 | 6 | 0.91 | 1.4% |
| F1LRE5 | Oxysterol-binding protein | 81.773 | 11.066 | 5 | 3 | 3 | 3 | 0.91 | 2.1% |
| D3ZQ32 | Protein LOC304903 | 198.56 | 20.296 | 2.7 | 4 | 6 | 4 | 0.91 | 4.5% |
| Q66H15 | Regulator of microtubule dynamics protein 3 | 52.311 | 25.216 | 14 | 6 | 6 | 6 | 0.91 | 6.9% |
| B2GV01 | Metastasis-associated gene family, member 2 | 74.959 | 25.315 | 13.5 | 8 | 8 | 6 | 0.90 | 1.5% |
| A2VD12 | Pre-B-cell leukemia transcription factor-interacting protein 1 | 80.275 | 71.697 | 19.4 | 12 | 17 | 12 | 0.90 | 0.3% |
| Q63014 | A-kinase anchor protein 8 | 76.161 | 16.229 | 5.1 | 3 | 3 | 3 | 0.90 | 8.9% |
| Q4V7F2 | Cysteine-rich with EGF-like domain protein 1 | 45.696 | 90.866 | 23.8 | 9 | 17 | 9 | 0.90 | 0.8% |
| P08503 | Medium-chain specific acyl-CoA dehydrogenase, mitochondrial | 46.555 | 46.041 | 23.8 | 9 | 14 | 9 | 0.90 | 0.7% |
| P56522 | NADPH:adrenodoxin oxidoreductase, mitochondrial | 54.362 | 12.485 | 10.7 | 4 | 4 | 4 | 0.90 | 4.1% |
| B2RZD1 | Protein Sec61b | 9.9884 | 40.753 | 37.5 | 3 | 7 | 3 | 0.90 | 4.6% |
| Q6IN37 | GM2 ganglioside activator | 21.493 | 19.831 | 18.1 | 5 | 11 | 5 | 0.90 | 4.3% |
| Q5U2Y1 | General transcription factor II-I | 110.21 | 15.488 | 5.9 | 5 | 6 | 5 | 0.90 | 0.2% |
| D3ZAI6 | Protein Nt5dc3 | 63.126 | 44.939 | 15.9 | 8 | 11 | 8 | 0.90 | 2.5% |
| Q64566 | Calcium-transporting ATPase type 2C member 1 | 100.5 | 76.598 | 7.6 | 6 | 6 | 6 | 0.90 | 3.4% |
| F1LQ09 | Protein Atl2 | 66.194 | 11.198 | 6.2 | 4 | 6 | 4 | 0.90 | 8.4% |
| G3V7K5 | Protein Npc1 | 142.95 | 27.068 | 4.5 | 6 | 11 | 6 | 0.90 | 3.8% |
| P80432 | Cytochrome c oxidase subunit 7C, mitochondrial | 7.3746 | 7.2467 | 28.6 | 2 | 8 | 2 | 0.90 | 2.3% |
| Q99M64 | Phosphatidylinositol 4-kinase type 2-alpha | 54.304 | 18.662 | 14.4 | 7 | 7 | 7 | 0.90 | 3.5% |
| D4ADF6 | Protein Zfyve16 | 167.53 | 23.134 | 1.8 | 2 | 2 | 2 | 0.90 | 5.0% |
| P20611 | Lysosomal acid phosphatase | 48.319 | 54.033 | 22.5 | 9 | 13 | 9 | 0.90 | 3.0% |
| Q32Q54 | Protein Uqcc1 | 34.546 | 11.585 | 7 | 2 | 3 | 2 | 0.90 | 1.9% |
| Q5RJY4 | Dehydrogenase/reductase SDR family member 7B | 35.342 | 35.228 | 16.3 | 5 | 9 | 5 | 0.90 | 1.5% |
| Q8K1P9 | Fatty acid desaturase 3 | 51.467 | 14.122 | 6 | 2 | 3 | 2 | 0.90 | 12.4% |
| E9PT23 | Protein Slc38a10 | 118.8 | 44.863 | 9.6 | 8 | 9 | 8 | 0.90 | 2.1% |
| G3V7Z8 | Poly(A) binding protein, nuclear 1, isoform CRA_a | 32.31 | 23.557 | 13.9 | 5 | 7 | 5 | 0.90 | 1.7% |
| P62961 | Nuclease-sensitive element-binding protein 1 | 35.73 | 93.694 | 32.9 | 5 | 13 | 4 | 0.90 | 1.0% |
| D4A8M4 | Protein Lrch3 | 85.116 | 20.918 | 4.2 | 3 | 5 | 3 | 0.90 | 2.2% |
| D3ZXD8 | Transmembrane protein 245 | 97.281 | 9.7439 | 3 | 2 | 2 | 2 | 0.90 | 5.7% |
| D4AAE9 | Protein Cisd2 | 15.293 | 26.003 | 32.6 | 4 | 6 | 4 | 0.90 | 4.4% |
| G3V9M6 | Fibrillin 1, isoform CRA_a | 311.95 | 45.249 | 4 | 8 | 9 | 8 | 0.90 | 1.8% |
| O35796 | Complement component 1 Q subcomponent-binding protein, mitochondrial | 30.997 | 82.27 | 31.9 | 6 | 14 | 6 | 0.90 | 0.1% |
| Q9WVK7 | Hydroxyacyl-coenzyme A dehydrogenase, mitochondrial | 34.447 | 35.395 | 28.3 | 10 | 18 | 10 | 0.90 | 3.1% |
| O35095 | Neurochondrin | 78.923 | 15.591 | 9.5 | 5 | 5 | 5 | 0.90 | 10.9% |
| G3V6P8 | Guanine nucleotide-binding protein subunit gamma | 7.9851 | 29.099 | 59.7 | 4 | 11 | 4 | 0.90 | 4.8% |
| Q6MG51 | Uncharacterized protein C6orf47 homolog | 31.825 | 6.0736 | 6.8 | 2 | 2 | 2 | 0.90 | 1.3% |
| Q5U2R9 | Protein Scfd2 | 74.794 | 85.618 | 16.1 | 9 | 12 | 9 | 0.90 | 0.9% |
| Q6P747 | Heterochromatin protein 1-binding protein 3 | 60.806 | 50.645 | 21.9 | 12 | 15 | 12 | 0.90 | 3.0% |
| Q8R490 | Cadherin 13 | 78.085 | 26.358 | 8.7 | 5 | 7 | 5 | 0.90 | 6.1% |
| P56574 | Isocitrate dehydrogenase [NADP], mitochondrial | 50.967 | 140.93 | 43.4 | 20 | 39 | 19 | 0.90 | 1.9% |
| Q10739 | Matrix metalloproteinase-14 | 66.079 | 36.779 | 13.2 | 7 | 9 | 7 | 0.90 | 3.2% |
| F1LQ48 | Protein Fblim1 | 67.902 | 170.07 | 36.4 | 17 | 31 | 17 | 0.90 | 1.3% |
| Q91ZN7 | Serine/threonine-protein kinase Chk1 | 54.428 | 2.5412 | 1.5 | 1 | 1 | 1 | 0.90 | 4.2% |
| P10818 | Cytochrome c oxidase subunit 6A1, mitochondrial | 12.301 | 31.822 | 40.5 | 2 | 9 | 2 | 0.90 | 4.4% |
| D3ZAS9 | Protein Ddrgk1 | 38.605 | 27.918 | 11.5 | 4 | 10 | 4 | 0.90 | 3.2% |
| Q9QZP1 | Gem-associated protein 2 | 30.44 | 6.1751 | 6.3 | 1 | 1 | 1 | 0.90 | 1.6% |
| A0A0G2JZ52 | Protein Hnrnpu | 87.931 | 280.89 | 41.6 | 31 | 58 | 31 | 0.90 | 1.1% |
| Q2THW7 | Palmitoyltransferase ZDHHC5 | 77.429 | 12.439 | 4.5 | 2 | 2 | 2 | 0.90 | 1.9% |
| Q924S1 | 1-acyl-sn-glycerol-3-phosphate acyltransferase delta | 43.794 | 6.2221 | 5.3 | 2 | 2 | 2 | 0.90 | 2.0% |
| P60905 | DnaJ homolog subfamily C member 5 | 22.101 | 46.537 | 16.2 | 4 | 4 | 4 | 0.90 | 5.7% |
| D3ZSR7 | Protein Ccdc102a | 62.612 | 67.069 | 14.6 | 6 | 8 | 6 | 0.90 | 0.7% |
| B1WBS6 | Glb1l protein | 73.261 | 26.306 | 10.1 | 5 | 6 | 5 | 0.90 | 0.2% |
| Q5XII9 | Mitochondrial fission regulator 1-like | 31.73 | 26.871 | 19.7 | 5 | 5 | 5 | 0.90 | 8.8% |
| D4A305 | Coiled-coil domain containing 58 (Predicted), isoform CRA_c | 16.69 | 25.403 | 32.6 | 5 | 5 | 5 | 0.90 | 1.0% |
| P04182 | Ornithine aminotransferase, mitochondrial | 48.332 | 197.05 | 45.1 | 17 | 29 | 17 | 0.90 | 1.4% |
| D3ZT71 | Protein Bcl2l13 | 46.734 | 42.098 | 12.7 | 4 | 4 | 4 | 0.90 | 6.6% |
| M0R785 | Protein Chchd2 | 15.849 | 26.122 | 29.2 | 3 | 4 | 3 | 0.90 | 5.1% |
| A0A0G2JU25 | Protein Galnt2 | 61.854 | 80.392 | 20.7 | 9 | 13 | 9 | 0.90 | 4.4% |
| A0A0G2K4C8 | Protein Fnbp4 | 115.83 | 7.943 | 1.2 | 1 | 1 | 1 | 0.90 | 1.7% |
| Q4KLZ3 | DAZ associated protein 1 | 43.086 | 68.391 | 24.4 | 6 | 10 | 6 | 0.90 | 1.8% |
| Q3KR56 | GRAM domain-containing protein 1A | 80.683 | 3.911 | 1.4 | 1 | 1 | 1 | 0.90 | 2.8% |
| Q4FZZ1 | PX domain-containing protein kinase-like protein | 65.097 | 18.754 | 6.7 | 3 | 3 | 3 | 0.90 | 13.2% |
| Q6AY46 | tRNA (adenine(58)-N(1))-methyltransferase catalytic subunit TRMT61A | 31.618 | 1.9119 | 2.4 | 1 | 2 | 1 | 0.90 | 1.9% |
| Q9QZK5 | Serine protease HTRA1 | 51.33 | 17.48 | 13.5 | 6 | 6 | 6 | 0.90 | 5.7% |
| F1LR02 | Procollagen, type XVIII, alpha 1, isoform CRA_a | 134.64 | 10.92 | 4.6 | 5 | 5 | 5 | 0.90 | 4.6% |
| Q08013 | Translocon-associated protein subunit gamma | 21.064 | 19.715 | 11.9 | 2 | 3 | 2 | 0.90 | 0.6% |
| P07154 | Cathepsin L1 | 37.66 | 44.544 | 20.7 | 6 | 14 | 6 | 0.90 | 0.2% |
| Q9JLH7 | CDK5 regulatory subunit-associated protein 3 | 57.044 | 61.283 | 26.6 | 13 | 16 | 13 | 0.90 | 2.1% |
| Q68FU3 | Electron transfer flavoprotein subunit beta | 27.687 | 112.93 | 55.7 | 13 | 23 | 13 | 0.90 | 2.3% |
| P47198 | 60S ribosomal protein L22 | 14.789 | 59.057 | 36.7 | 3 | 5 | 1 | 0.90 | 2.9% |
| P02401 | 60S acidic ribosomal protein P2 | 11.692 | 174.73 | 92.2 | 8 | 27 | 7 | 0.90 | 6.0% |
| P14841 | Cystatin-C | 15.437 | 27.247 | 20 | 2 | 4 | 2 | 0.90 | 7.8% |
| O35254 | Golgi reassembly-stacking protein 1 | 47.672 | 18.445 | 7.8 | 3 | 5 | 3 | 0.89 | 0.5% |
| P55770 | NHP2-like protein 1 | 14.173 | 18.46 | 31.2 | 3 | 5 | 3 | 0.89 | 0.3% |
| E9PT53 | Protein Wfs1 | 100.56 | 100.13 | 17.6 | 13 | 17 | 13 | 0.89 | 0.8% |
| Q9Z1H9 | Protein kinase C delta-binding protein | 27.91 | 31.344 | 35.4 | 9 | 12 | 9 | 0.89 | 0.8% |
| Q6AYA5 | Transmembrane protein 106B | 31.152 | 19.45 | 11.3 | 3 | 3 | 3 | 0.89 | 2.7% |
| P07092 | Glia-derived nexin | 44.063 | 22.032 | 7.6 | 2 | 2 | 2 | 0.89 | 0.8% |
| B2GV62 | Mitochondrial ribosomal protein L20 | 17.525 | 5.1844 | 12.1 | 2 | 2 | 2 | 0.89 | 2.0% |
| Q6IUR5 | Neudesin | 18.991 | 73.262 | 67.3 | 9 | 16 | 9 | 0.89 | 2.5% |
| D3ZUX7 | Protein Acsf3 | 65.265 | 14.196 | 9.8 | 5 | 8 | 5 | 0.89 | 2.5% |
| P35171 | Cytochrome c oxidase subunit 7A2, mitochondrial | 9.3529 | 20.647 | 18.1 | 2 | 7 | 2 | 0.89 | 1.3% |
| M0R436 | Protein Rnf170 | 29.753 | 50.073 | 19.5 | 3 | 3 | 3 | 0.89 | 7.6% |
| D3ZRX9 | Calponin | 24.126 | 157.57 | 62.7 | 14 | 67 | 12 | 0.89 | 0.7% |
| A0A0G2JVZ6 | Protein Itgav | 115.81 | 99.634 | 18.3 | 16 | 20 | 16 | 0.89 | 1.0% |
| A0A0G2K5L1 | Protein Fat1 | 507.34 | 73.655 | 4.8 | 19 | 19 | 19 | 0.89 | 1.6% |
| D3ZUX5 | Coiled-coil-helix-coiled-coil-helix domain containing 3 (Predicted), isoform CRA_a | 26.434 | 58.629 | 32.2 | 7 | 12 | 7 | 0.89 | 3.0% |
| Q9JHW5 | Vesicle-associated membrane protein 7 | 24.775 | 47.024 | 26.8 | 6 | 8 | 6 | 0.89 | 4.8% |
| D4A617 | Ectonucleoside triphosphate diphosphohydrolase 1 | 52.016 | 12.446 | 5.8 | 3 | 4 | 3 | 0.89 | 3.4% |
| D3ZY47 | Protein RGD1559896 | 35.668 | 216.22 | 50 | 13 | 26 | 13 | 0.89 | 0.7% |
| D3ZK14 | Protein Tnn | 173.25 | 161.85 | 21.4 | 25 | 37 | 25 | 0.89 | 2.5% |
| G3V648 | 1-acylglycerol-3-phosphate O-acyltransferase 3 (Predicted), isoform CRA_b | 43.245 | 19.622 | 10.4 | 3 | 3 | 3 | 0.89 | 6.3% |
| Q76K24 | Ankyrin repeat domain-containing protein 46 | 25.18 | 15.904 | 9.6 | 2 | 3 | 2 | 0.89 | 1.4% |
| D4A705 | Protein Rint1 | 85.035 | 26.086 | 7.8 | 6 | 6 | 6 | 0.89 | 9.5% |
| F1M7H3 | Protein LOC100359912 | 90.798 | 35.569 | 12.7 | 10 | 12 | 10 | 0.89 | 4.8% |
| M0R808 | Up-regulated during skeletal muscle growth protein 5 | 6.4204 | 38.298 | 27.6 | 2 | 4 | 2 | 0.89 | 1.3% |
| D4A3X0 | Protein Seh1l | 260.99 | 17.608 | 1.8 | 5 | 12 | 2 | 0.89 | 3.8% |
| P0C089 | Phosphatidylglycerophosphatase and protein-tyrosine phosphatase 1 | 21.886 | 24.359 | 26.9 | 5 | 7 | 5 | 0.89 | 5.2% |
| D3ZXP3 | Histone H2A | 15.114 | 30.777 | 27.3 | 7 | 14 | 3 | 0.89 | 2.1% |
| Q3ZB99 | Protein Tjp2 | 131.36 | 40.679 | 12.5 | 13 | 14 | 13 | 0.89 | 0.7% |
| Q5BJP5 | Transmembrane protein 230 | 13.204 | 18.046 | 20.8 | 2 | 3 | 2 | 0.89 | 1.5% |
| D3ZQN7 | Protein Lamb1 | 197.39 | 138.46 | 12.5 | 20 | 23 | 20 | 0.89 | 1.7% |
| M0R849 |  | 44.019 | 5.3663 | 3.1 | 1 | 1 | 1 | 0.89 | 7.9% |
| P27274 | CD59 glycoprotein | 13.79 | 10.968 | 30.2 | 4 | 5 | 4 | 0.89 | 1.0% |
| P97546 | Neuroplastin | 43.931 | 8.2455 | 8.7 | 3 | 6 | 3 | 0.89 | 0.0% |
| Q64232 | Very-long-chain enoyl-CoA reductase | 36.122 | 24.538 | 20.1 | 8 | 11 | 8 | 0.89 | 1.2% |
| B2RYS9 | Protein Trmt112 | 14.127 | 13.238 | 21.6 | 2 | 3 | 2 | 0.89 | 1.7% |
| Q566E5 | KDEL motif-containing protein 2 | 58.701 | 88.869 | 31.1 | 12 | 18 | 12 | 0.89 | 3.4% |
| D4A742 | Protein LOC100910944 | 8.1828 | 2.2446 | 21.5 | 1 | 1 | 1 | 0.89 | 6.1% |
| Q09326 | Alpha-1,6-mannosyl-glycoprotein 2-beta-N-acetylglucosaminyltransferase | 51.109 | 25.448 | 13.8 | 5 | 6 | 5 | 0.89 | 1.6% |
| P63025 | Vesicle-associated membrane protein 3 | 11.48 | 124.41 | 39.8 | 5 | 14 | 2 | 0.89 | 8.7% |
| D4A206 | Protein Tcof1 | 144.66 | 58.003 | 8.3 | 10 | 11 | 10 | 0.89 | 3.8% |
| Q8JZQ0 | Macrophage colony-stimulating factor 1 | 62.186 | 71.508 | 15.5 | 8 | 9 | 8 | 0.89 | 2.2% |
| D3ZT90 | Glutaryl-Coenzyme A dehydrogenase (Predicted) | 49.713 | 32.576 | 13.4 | 4 | 6 | 4 | 0.89 | 1.7% |
| Q63190 | Emerin | 29.675 | 63.757 | 44.2 | 9 | 20 | 9 | 0.89 | 1.2% |
| D3ZZX1 | Inositol polyphosphate-5-phosphatase A (Predicted), isoform CRA_a | 47.622 | 15.376 | 17.2 | 7 | 8 | 7 | 0.89 | 6.3% |
| Q8K1Q0 | Glycylpeptide N-tetradecanoyltransferase 1 | 56.86 | 60.858 | 20.8 | 8 | 14 | 6 | 0.89 | 1.2% |
| P09606 | Glutamine synthetase | 42.267 | 11.697 | 10.7 | 4 | 4 | 4 | 0.89 | 0.8% |
| Q9JL55 | Glycerophosphodiester phosphodiesterase 1 | 37.634 | 12.553 | 14.5 | 4 | 4 | 4 | 0.89 | 4.1% |
| A7VJC2 | Heterogeneous nuclear ribonucleoproteins A2/B1 | 37.477 | 255.19 | 62 | 20 | 59 | 20 | 0.89 | 0.8% |
| A0A0G2K4F6 | Protein Srrm1 | 102.91 | 20.548 | 6.7 | 3 | 3 | 3 | 0.89 | 2.3% |
| P63322 | Ras-related protein Ral-A | 23.553 | 38.058 | 26.2 | 6 | 9 | 3 | 0.89 | 1.0% |
| P15650 | Long-chain specific acyl-CoA dehydrogenase, mitochondrial | 47.872 | 147.28 | 33.5 | 13 | 39 | 13 | 0.89 | 0.1% |
| Q5RK08 | Glioblastoma amplified sequence | 32.941 | 20.667 | 19.9 | 5 | 8 | 5 | 0.89 | 2.2% |
| P08721 | Osteopontin | 34.963 | 13.499 | 11.7 | 4 | 4 | 4 | 0.89 | 1.2% |
| B5DEP7 | Protein LOC100360635 | 8.496 | 16.476 | 35.5 | 3 | 6 | 3 | 0.89 | 11.4% |
| P56571 | ES1 protein homolog, mitochondrial | 28.172 | 186.98 | 47.4 | 9 | 15 | 9 | 0.89 | 0.7% |
| Q641Z9 | Protein Sdhc | 18.202 | 2.6933 | 4.7 | 1 | 2 | 1 | 0.89 | 1.2% |
| Q4V897 | Coiled-coil domain-containing protein 90B, mitochondrial | 29.8 | 79.454 | 19.1 | 3 | 3 | 3 | 0.89 | 1.8% |
| Q03348 | Receptor-type tyrosine-protein phosphatase alpha | 90.259 | 14.536 | 3.4 | 2 | 3 | 2 | 0.89 | 1.4% |
| P33436 | 72 kDa type IV collagenase | 74.149 | 41.033 | 13.6 | 7 | 9 | 7 | 0.89 | 3.7% |
| Q5HZY0 | UBX domain-containing protein 4 | 56.393 | 54.167 | 19.6 | 8 | 13 | 8 | 0.88 | 0.1% |
| Q2PS20 | Junctophilin-2 | 74.258 | 65.928 | 14.7 | 9 | 14 | 9 | 0.88 | 2.4% |
| D3ZEA0 | Fibronectin type III domain containing 3a (Predicted), isoform CRA_a | 128.99 | 2.9907 | 0.8 | 1 | 1 | 1 | 0.88 | 0.2% |
| Q5XIM4 | ATP synthase subunit s, mitochondrial | 23.324 | 9.6917 | 9.5 | 2 | 3 | 2 | 0.88 | 2.1% |
| P29411 | GTP:AMP phosphotransferase AK3, mitochondrial | 25.438 | 95.354 | 46.7 | 10 | 16 | 10 | 0.88 | 0.4% |
| O88453 | Scaffold attachment factor B1 | 104.57 | 36.337 | 9.2 | 7 | 9 | 2 | 0.88 | 4.0% |
| D4A4W7 | Protein Mgarp | 29.117 | 16.398 | 6.5 | 1 | 1 | 1 | 0.88 | 4.4% |
| D3ZMS1 | Protein Sf3b2 | 98.146 | 76.891 | 14.2 | 11 | 14 | 11 | 0.88 | 2.3% |
| Q5U2R7 | LDLR chaperone MESD | 25.215 | 67.019 | 39.7 | 7 | 13 | 7 | 0.88 | 4.2% |
| D3ZZR5 | Protein LOC100364748 | 28.317 | 24.587 | 18.4 | 6 | 7 | 6 | 0.88 | 3.8% |
| P13941 | Collagen alpha-1(III) chain | 138.93 | 210.94 | 23.5 | 28 | 57 | 21 | 0.88 | 1.9% |
| P30121 | Metalloproteinase inhibitor 2 | 24.356 | 7.9696 | 10.5 | 2 | 2 | 2 | 0.88 | 1.4% |
| Q5RJQ8 | Calcium homeostasis modulator protein 2 | 35.931 | 3.4164 | 8.4 | 2 | 2 | 2 | 0.88 | 9.4% |
| Q62632 | Follistatin-related protein 1 | 34.622 | 74.383 | 35.9 | 11 | 21 | 11 | 0.88 | 0.3% |
| P10960 | Sulfated glycoprotein 1 | 61.123 | 129.41 | 33.6 | 16 | 50 | 16 | 0.88 | 0.6% |
| Q9WUF4 | Vesicle-associated membrane protein 8 | 11.32 | 6.548 | 18 | 2 | 3 | 2 | 0.88 | 0.9% |
| P62738 | Actin, aortic smooth muscle | 42.009 | 323.31 | 78.2 | 28 | 636 | 2 | 0.88 | 9.5% |
| Q5I0K8 | 28S ribosomal protein S7, mitochondrial | 28.197 | 25.77 | 17.8 | 4 | 6 | 4 | 0.88 | 6.6% |
| D4A131 | Mitochondrial ribosomal protein L4 (Predicted), isoform CRA_b | 33.082 | 7.9254 | 10.5 | 3 | 3 | 3 | 0.88 | 3.1% |
| D3ZA85 | Histone cell cycle regulation defective interacting protein 5 (Predicted), isoform CRA_a | 28.367 | 18.012 | 15.8 | 3 | 5 | 3 | 0.88 | 2.8% |
| P04937 | Fibronectin | 272.51 | 323.31 | 30.5 | 58 | 98 | 58 | 0.88 | 1.1% |
| Q07257 | Transforming growth factor beta-2 | 50.533 | 90.165 | 24.9 | 9 | 11 | 9 | 0.88 | 3.6% |
| Q58NB7 | Protein Rarres1 | 31.952 | 3.35 | 3.2 | 1 | 1 | 1 | 0.88 | 10.1% |
| Q9JMA8 | Exostoses (Multiple)-like 3, isoform CRA_a | 104.44 | 33.953 | 9.4 | 7 | 8 | 7 | 0.88 | 5.2% |
| B5DFN3 | Ubiquinol-cytochrome-c reductase complex assembly factor 2 | 16.33 | 54.233 | 27.9 | 3 | 6 | 3 | 0.88 | 5.3% |
| A4L9P7 | Sister chromatid cohesion protein PDS5 homolog A | 150.28 | 52.469 | 4.1 | 5 | 6 | 4 | 0.88 | 2.4% |
| O88483 | [Pyruvate dehydrogenase [acetyl-transferring]]-phosphatase 1, mitochondrial | 61.207 | 43.647 | 11 | 5 | 8 | 5 | 0.88 | 2.6% |
| A0A0G2JXG5 |  | 17.705 | 6.0562 | 12.7 | 2 | 2 | 2 | 0.88 | 4.9% |
| Q4KM74 | Vesicle-trafficking protein SEC22b | 24.74 | 142.09 | 54.9 | 12 | 30 | 12 | 0.88 | 0.5% |
| P14925 | Peptidyl-glycine alpha-amidating monooxygenase | 108.67 | 35.459 | 6.9 | 5 | 5 | 5 | 0.88 | 2.9% |
| Q62703 | Reticulocalbin-2 | 37.432 | 191.86 | 47.5 | 15 | 41 | 15 | 0.88 | 0.1% |
| P36860 | Ras-related protein Ral-B | 23.317 | 10.061 | 22.8 | 5 | 8 | 2 | 0.88 | 1.6% |
| G3V7X8 | Cytochrome P450 26B1 | 57.352 | 13.209 | 2.9 | 1 | 1 | 1 | 0.88 | 6.4% |
| P50408 | V-type proton ATPase subunit F | 13.37 | 56.869 | 47.9 | 5 | 8 | 5 | 0.88 | 1.4% |
| D3ZDW3 | Protein Tsc22d2 | 78.417 | 7.971 | 4.6 | 4 | 6 | 2 | 0.88 | 8.9% |
| O08874 | Serine/threonine-protein kinase N2 | 112.07 | 27.41 | 8.4 | 7 | 7 | 6 | 0.88 | 1.9% |
| D3ZF97 | Protein Erlec1 | 54.665 | 25.923 | 12.2 | 5 | 6 | 5 | 0.88 | 1.5% |
| O70352 | CD82 antigen | 29.487 | 7.7763 | 7.5 | 2 | 2 | 2 | 0.88 | 2.1% |
| F1LTJ5 | Uncharacterized protein | 263.11 | 104.4 | 10.1 | 17 | 18 | 17 | 0.88 | 3.2% |
| D4A531 | DNA-directed RNA polymerase subunit | 14.523 | 11.653 | 18.4 | 1 | 1 | 1 | 0.88 | 3.3% |
| B2RYU6 | Trafficking protein particle complex subunit 2-like protein | 16.031 | 15.053 | 22.3 | 3 | 3 | 3 | 0.88 | 4.6% |
| M0RBV9 |  | 30.556 | 4.9675 | 3.6 | 1 | 2 | 1 | 0.88 | 3.2% |
| Q5M9I5 | Cytochrome b-c1 complex subunit 6, mitochondrial | 10.424 | 18.604 | 29.2 | 2 | 6 | 2 | 0.88 | 8.0% |
| O88984 | Nuclear RNA export factor 1 | 70.361 | 8.1392 | 3.4 | 2 | 2 | 2 | 0.88 | 1.6% |
| Q5XID8 | FK506 binding protein 14 | 24.254 | 5.8441 | 8.5 | 2 | 4 | 2 | 0.88 | 0.7% |
| G3V8S8 | Oxysterol-binding protein | 101.67 | 14.63 | 4.7 | 4 | 5 | 4 | 0.88 | 10.8% |
| P10252 | CD48 antigen | 27.679 | 10.753 | 16.2 | 5 | 6 | 5 | 0.88 | 2.1% |
| D3ZZV1 | Mitochondrial import inner membrane translocase subunit TIM16 | 13.771 | 8.9024 | 22.4 | 3 | 4 | 3 | 0.88 | 0.7% |
| O70280 | WAP four-disulfide core domain protein 1 | 23.23 | 14.004 | 10.8 | 2 | 3 | 2 | 0.88 | 1.7% |
| Q9ESQ5 | Transient receptor potential cation channel subfamily M member 4 | 135.34 | 3.6612 | 1.7 | 2 | 2 | 2 | 0.88 | 2.3% |
| P52590 | Nuclear pore complex protein Nup107 | 107.21 | 14.876 | 5.4 | 5 | 5 | 5 | 0.88 | 1.4% |
| P23764 | Glutathione peroxidase 3 | 25.424 | 3.7731 | 6.6 | 1 | 1 | 1 | 0.88 | 0.3% |
| B2RYS2 | Cytochrome b-c1 complex subunit 7 | 13.558 | 26.487 | 36.9 | 4 | 8 | 4 | 0.88 | 1.1% |
| O35783 | Calumenin | 36.996 | 210.01 | 39.7 | 11 | 34 | 11 | 0.88 | 3.9% |
| D4A845 | Protein Rpa3 | 13.639 | 8.4845 | 14 | 1 | 2 | 1 | 0.88 | 10.0% |
| Q6JE36 | Protein NDRG1 | 42.954 | 84.788 | 24.9 | 6 | 10 | 6 | 0.88 | 5.1% |
| P20961 | Plasminogen activator inhibitor 1 | 45.009 | 93.627 | 28.4 | 11 | 12 | 11 | 0.87 | 3.6% |
| B1WC88 | UPF0729 protein C18orf32 homolog | 8.1686 | 8.8487 | 37.5 | 3 | 4 | 3 | 0.87 | 2.9% |
| Q9R1E9 | Connective tissue growth factor | 37.756 | 128.31 | 66 | 21 | 43 | 21 | 0.87 | 1.1% |
| D4A465 | Protein Lamtor2 | 13.48 | 38.978 | 51.2 | 5 | 6 | 5 | 0.87 | 7.5% |
| A0A0G2K2D4 | Protein Agap3 | 88.357 | 10.956 | 3.4 | 2 | 2 | 2 | 0.87 | 14.6% |
| Q6AYQ1 | Golgin subfamily A member 7 | 15.778 | 14.263 | 16.8 | 2 | 2 | 2 | 0.87 | 2.7% |
| F1LXZ9 | Protein Asxl2 | 145.77 | 4.0935 | 0.7 | 1 | 1 | 1 | 0.87 | 0.2% |
| Q9Z311 | Trans-2-enoyl-CoA reductase, mitochondrial | 40.326 | 25.111 | 11.5 | 3 | 3 | 3 | 0.87 | 1.1% |
| A0A0G2K613 | MARCKS-related protein | 19.718 | 19.85 | 7.5 | 1 | 1 | 1 | 0.87 | 0.5% |
| Q921A2 | Proton myo-inositol cotransporter | 69.149 | 100.27 | 16.8 | 9 | 11 | 9 | 0.87 | 1.3% |
| B4F7A5 | Cd99 protein | 16.588 | 10.502 | 11.5 | 1 | 1 | 1 | 0.87 | 13.0% |
| B5DEQ3 | Probable Xaa-Pro aminopeptidase 3 | 56.454 | 9.8139 | 5.7 | 3 | 3 | 3 | 0.87 | 6.1% |
| Q99N37 | Rho GTPase-activating protein 17 | 93.753 | 33.345 | 8.3 | 7 | 8 | 7 | 0.87 | 0.9% |
| Q3SWU3 | Heterogeneous nuclear ribonucleoprotein D-like | 35.294 | 29.007 | 18.9 | 6 | 9 | 4 | 0.87 | 4.1% |
| Q5U1W6 | Apolipoprotein O-like | 28.237 | 63.732 | 35.8 | 8 | 15 | 8 | 0.87 | 0.3% |
| Q4QR80 | 28S ribosomal protein S25, mitochondrial | 19.775 | 17.012 | 17.5 | 2 | 3 | 2 | 0.87 | 6.1% |
| Q6AXT0 | 39S ribosomal protein L37, mitochondrial | 48.365 | 11.886 | 5.9 | 2 | 2 | 2 | 0.87 | 10.7% |
| B5DEZ8 | Plexin domain containing 2 | 59.416 | 51.486 | 17.2 | 9 | 13 | 9 | 0.87 | 1.7% |
| D3ZDQ5 | Protein Ccdc88a | 212.46 | 39.618 | 1.8 | 3 | 3 | 3 | 0.87 | 7.6% |
| P15129 | Cytochrome P450 4B1 | 58.936 | 22.424 | 14.1 | 6 | 6 | 6 | 0.87 | 11.1% |
| G3V7F6 | Protein RGD1561590 | 19.586 | 23.529 | 24.4 | 5 | 6 | 5 | 0.87 | 2.6% |
| P62804 | Histone H4 | 11.367 | 61.085 | 59.2 | 9 | 37 | 9 | 0.87 | 1.3% |
| D4A533 | Protein Tapt1 | 69.564 | 7.1276 | 3 | 3 | 4 | 3 | 0.87 | 6.6% |
| D4A4Q4 | Protein Ociad2 | 14.682 | 7.8891 | 14.4 | 2 | 3 | 2 | 0.87 | 3.8% |
| Q6P791 | Ragulator complex protein LAMTOR1 | 17.721 | 38.402 | 23.6 | 4 | 7 | 4 | 0.87 | 0.3% |
| Q9QWJ9 | Neuropilin-1 | 103.08 | 14.465 | 1.8 | 1 | 2 | 1 | 0.87 | 9.0% |
| Q3KRE2 | Methyltransferase like 7A | 28.142 | 13.805 | 11.1 | 2 | 2 | 2 | 0.87 | 6.7% |
| Q5FVQ7 | Membrane protein FAM174A | 20.228 | 5.7773 | 5.8 | 1 | 1 | 1 | 0.87 | 4.3% |
| Q00918 | Latent-transforming growth factor beta-binding protein 1 | 186.6 | 13.215 | 3.2 | 4 | 4 | 4 | 0.87 | 5.6% |
| Q99P55 | Sphingosine-1-phosphate phosphatase 1 | 47.649 | 5.6099 | 4 | 2 | 2 | 2 | 0.87 | 8.8% |
| B2GV24 | E3 UFM1-protein ligase 1 | 89.584 | 109.15 | 29.5 | 21 | 28 | 20 | 0.87 | 3.6% |
| Q5PQM2 | Kinesin light chain 4 | 68.963 | 19.557 | 17.9 | 9 | 12 | 5 | 0.87 | 0.1% |
| P50123 | Glutamyl aminopeptidase | 107.99 | 13.257 | 5.5 | 4 | 4 | 4 | 0.87 | 4.5% |
| F1LXA0 | NADH dehydrogenase (Ubiquinone) 1 alpha subcomplex, 12 (Predicted), isoform CRA_b | 17.177 | 20.639 | 26.2 | 3 | 4 | 3 | 0.87 | 0.6% |
| Q68FT3 | Pyridine nucleotide-disulfide oxidoreductase domain-containing protein 2 | 62.878 | 13.084 | 4.3 | 2 | 3 | 2 | 0.87 | 2.9% |
| F1LV37 | Protein Tnrc6b | 192.86 | 2.7412 | 1 | 2 | 2 | 2 | 0.87 | 2.3% |
| O35217 | Multiple inositol polyphosphate phosphatase 1 | 54.589 | 79.886 | 25.2 | 10 | 20 | 10 | 0.87 | 0.7% |
| B2GV94 | Fam134c protein | 51.528 | 7.0492 | 3.2 | 1 | 2 | 1 | 0.87 | 7.2% |
| D4ADD7 | Glutaredoxin 5 homolog (S. cerevisiae) (Predicted), isoform CRA_b | 16.432 | 47.685 | 34.9 | 4 | 8 | 4 | 0.87 | 2.6% |
| Q3MIE0 | Enoyl-CoA hydratase domain-containing protein 3, mitochondrial | 32.385 | 3.8495 | 6.7 | 2 | 2 | 2 | 0.87 | 4.2% |
| E9PU01 | Chromodomain-helicase-DNA-binding protein 5 | 218.18 | 93.899 | 7.4 | 12 | 14 | 12 | 0.86 | 2.4% |
| Q5FVN1 | Starch-binding domain-containing protein 1 | 35.494 | 7.3823 | 9 | 4 | 4 | 4 | 0.86 | 1.4% |
| Q63041 | Alpha-1-macroglobulin | 167.12 | 13.022 | 1.9 | 3 | 8 | 3 | 0.86 | 3.6% |
| P63045 | Vesicle-associated membrane protein 2 | 12.691 | 12.94 | 34.5 | 4 | 7 | 1 | 0.86 | 5.0% |
| D4A7I8 | Protein Sumf1 | 40.079 | 8.2738 | 7.3 | 2 | 3 | 2 | 0.86 | 2.9% |
| D3ZCL3 | U1 small nuclear ribonucleoprotein C | 17.364 | 3.0955 | 5.7 | 1 | 1 | 1 | 0.86 | 19.7% |
| P04218 | OX-2 membrane glycoprotein | 31.088 | 3.5273 | 4.3 | 1 | 1 | 1 | 0.86 | 0.6% |
| P29457 | Serpin H1 | 46.517 | 323.31 | 54.7 | 21 | 114 | 21 | 0.86 | 0.9% |
| Q64542 | Plasma membrane calcium-transporting ATPase 4 | 133.09 | 36.531 | 5.4 | 4 | 6 | 1 | 0.86 | 5.9% |
| D3ZS58 | NADH dehydrogenase [ubiquinone] 1 alpha subcomplex subunit 2 | 10.844 | 27.748 | 33 | 3 | 4 | 3 | 0.86 | 0.9% |
| D3ZV60 | Kinesin-like protein | 281.97 | 2.1713 | 1 | 2 | 2 | 2 | 0.86 | 0.3% |
| D3ZDF3 | MRV integration site 1 homolog (Mouse) (Predicted), isoform CRA_b | 93.566 | 19.913 | 7 | 5 | 6 | 5 | 0.86 | 2.1% |
| P07340 | Sodium/potassium-transporting ATPase subunit beta-1 | 35.201 | 39.679 | 15.5 | 5 | 6 | 5 | 0.86 | 1.8% |
| P83871 | PHD finger-like domain-containing protein 5A | 12.405 | 5.9701 | 20 | 2 | 3 | 2 | 0.86 | 0.1% |
| M0R8V0 | Protein LOC100910056 | 162.87 | 14.012 | 2.9 | 4 | 5 | 4 | 0.86 | 7.8% |
| Q8K3X0 | Protein CASC3 | 75.903 | 6.723 | 3 | 2 | 2 | 2 | 0.86 | 0.8% |
| Q66HG6 | Carbonic anhydrase 5B, mitochondrial | 36.597 | 10.873 | 11.4 | 3 | 3 | 3 | 0.86 | 3.3% |
| Q6AY23 | Pyrroline-5-carboxylate reductase 2 | 33.673 | 30.565 | 19.4 | 6 | 9 | 6 | 0.86 | 2.1% |
| Q5U2P1 | Metal transporter CNNM2 | 96.608 | 11.797 | 4.2 | 4 | 4 | 4 | 0.86 | 2.0% |
| Q08290 | Calponin-1 | 33.343 | 243.26 | 64.3 | 19 | 84 | 17 | 0.86 | 0.1% |
| P24942 | Excitatory amino acid transporter 1 | 59.697 | 2.2761 | 2 | 1 | 1 | 1 | 0.86 | 4.3% |
| B2RZ37 | Receptor expression-enhancing protein 5 | 21.431 | 22.683 | 21.7 | 7 | 10 | 7 | 0.86 | 5.6% |
| D4A5T1 | Protein Sf3b5 | 10.119 | 6.6153 | 30.2 | 2 | 2 | 2 | 0.86 | 3.4% |
| B2GV35 | B3gat3 protein | 37.072 | 6.78 | 7.2 | 2 | 2 | 2 | 0.86 | 1.1% |
| P23785 | Granulins | 63.369 | 85.531 | 19 | 8 | 24 | 8 | 0.86 | 3.7% |
| B5DEZ3 | Ccdc126 protein | 15.532 | 20.709 | 27.9 | 3 | 4 | 3 | 0.86 | 2.6% |
| F2Z3T8 | Guanine nucleotide-binding protein subunit gamma | 7.1872 | 4.4619 | 23.9 | 2 | 3 | 2 | 0.85 | 1.1% |
| P70490 | Lactadherin | 47.412 | 161.36 | 43.3 | 15 | 23 | 15 | 0.85 | 1.3% |
| P11661 | NADH-ubiquinone oxidoreductase chain 5 | 68.617 | 7.4228 | 1.8 | 1 | 2 | 1 | 0.85 | 2.8% |
| Q6AYQ4 | Transmembrane protein 109 | 26.242 | 18.586 | 8.6 | 2 | 4 | 2 | 0.85 | 2.9% |
| B1H219 | Dkk3 protein | 38.738 | 6.114 | 8.9 | 4 | 4 | 4 | 0.85 | 8.2% |
| Q6AY72 | UPF0449 protein C19orf25 homolog | 12.142 | 50.74 | 66.1 | 4 | 6 | 4 | 0.85 | 4.8% |
| P70560 | Collagen alpha-1(XII) chain | 32.001 | 13.83 | 20.2 | 6 | 8 | 6 | 0.85 | 2.8% |
| A0A0G2K904 | Protein Sltm | 117.17 | 12.973 | 4.6 | 5 | 5 | 5 | 0.85 | 2.6% |
| G3V824 | Insulin-like growth factor 2 receptor, isoform CRA_b | 273.59 | 15.727 | 2.1 | 5 | 7 | 5 | 0.85 | 1.6% |
| P97849 | Long-chain fatty acid transport protein 1 | 71.282 | 12.097 | 11.6 | 8 | 10 | 6 | 0.85 | 1.2% |
| M0R9L0 | Protein Naca | 220.19 | 77.522 | 3 | 5 | 16 | 5 | 0.85 | 2.1% |
| A0A0G2K0P5 | Protein Trip4 | 76.984 | 43.353 | 10.1 | 4 | 4 | 4 | 0.85 | 2.6% |
| O70454 | Protein BUD31 homolog | 17 | 6.6186 | 14.6 | 2 | 2 | 2 | 0.85 | 0.6% |
| Q5U4E6 | Golgin subfamily A member 4 | 260.19 | 76.597 | 5.8 | 11 | 12 | 11 | 0.85 | 2.1% |
| P30009 | Myristoylated alanine-rich C-kinase substrate | 29.794 | 80.933 | 16.2 | 5 | 11 | 5 | 0.85 | 5.7% |
| A0A0G2K1L0 | Protein Tnc | 221.74 | 242.54 | 17 | 25 | 33 | 25 | 0.85 | 2.6% |
| G3V9W0 | Centrin 2, isoform CRA_a | 19.841 | 11.429 | 7.6 | 1 | 1 | 1 | 0.85 | 9.4% |
| D4A416 | Protein Clptm1l | 62.29 | 16.046 | 6.3 | 3 | 4 | 3 | 0.85 | 2.6% |
| Q5XI41 | Translocating chain-associated membrane protein 1 | 43.03 | 8.7007 | 3.2 | 2 | 2 | 2 | 0.85 | 0.4% |
| Q5PQM0 | Transmembrane protein 168 | 79.771 | 4.6628 | 2.4 | 1 | 2 | 1 | 0.85 | 3.5% |
| D3ZV75 | Protein Mfsd1 | 51.2 | 19.372 | 3.9 | 1 | 2 | 1 | 0.85 | 5.5% |
| Q3B8N9 | Biphenyl hydrolase-like (Serine hydrolase) | 32.822 | 9.4122 | 6.2 | 2 | 3 | 2 | 0.85 | 0.5% |
| Q6AXU7 | MIF4G domain-containing protein | 25.471 | 13.382 | 18 | 3 | 4 | 3 | 0.85 | 12.3% |
| B5DEG1 | Protein Itga8 | 117.57 | 7.1958 | 2.4 | 3 | 3 | 3 | 0.85 | 1.6% |
| Q5BJU0 | Protein Rras2 | 23.399 | 63.574 | 49.5 | 10 | 21 | 8 | 0.85 | 2.9% |
| Q5PQQ2 | WW domain-binding protein 11 | 69.995 | 17.013 | 10.3 | 6 | 6 | 6 | 0.85 | 0.9% |
| Q63450 | Calcium/calmodulin-dependent protein kinase type 1 | 41.638 | 93.076 | 25.9 | 7 | 9 | 6 | 0.84 | 4.1% |
| Q5XIH7 | Prohibitin-2 | 33.312 | 83.271 | 50.2 | 15 | 26 | 15 | 0.84 | 6.0% |
| M0R5H9 |  | 34.477 | 15.861 | 21.9 | 5 | 5 | 5 | 0.84 | 1.1% |
| B1WC73 | ADP-ribosylation factor-like 6 (Predicted), isoform CRA_a | 20.968 | 5.0998 | 5.9 | 1 | 1 | 1 | 0.84 | 3.4% |
| D4A565 | NADH dehydrogenase (Ubiquinone) 1 beta subcomplex, 5 (Predicted), isoform CRA_b | 21.664 | 12.623 | 15.9 | 4 | 4 | 4 | 0.84 | 1.6% |
| A0A0G2K2M9 | Protein Srrm2 | 295.11 | 76.631 | 6.5 | 14 | 15 | 14 | 0.84 | 0.4% |
| D3ZJG3 | Protein P4htm | 56.881 | 22.908 | 10.9 | 4 | 5 | 4 | 0.84 | 2.9% |
| A0A0G2K0K6 | Protein Ap1ar | 33.921 | 1.9413 | 2.7 | 1 | 1 | 1 | 0.84 | 15.5% |
| P41350 | Caveolin-1 | 20.552 | 50.397 | 51.1 | 9 | 18 | 9 | 0.84 | 0.9% |
| F1LPD0 | Protein Col15a1 | 134.49 | 37.01 | 4.4 | 5 | 5 | 5 | 0.84 | 4.9% |
| Q6P767 | Pituitary tumor-transforming gene 1 protein-interacting protein | 19.9 | 3.5347 | 8.6 | 2 | 2 | 2 | 0.84 | 3.6% |
| Q497A2 | D-serine modulator-1 | 47.297 | 10.775 | 4.9 | 3 | 3 | 3 | 0.84 | 5.8% |
| D3ZFQ1 | Protein Atxn7l3b | 10.744 | 2.2499 | 10.3 | 1 | 1 | 1 | 0.84 | 1.8% |
| Q7M0E7 | 39S ribosomal protein L14, mitochondrial | 15.912 | 1.9884 | 6.2 | 1 | 1 | 1 | 0.84 | 0.8% |
| Q9R080 | G-protein-signaling modulator 1 | 74.439 | 35.358 | 5.9 | 2 | 2 | 2 | 0.84 | 1.0% |
| P37397 | Calponin-3 | 36.434 | 168.11 | 58.2 | 18 | 73 | 15 | 0.84 | 0.3% |
| A0A0G2KAH7 | Protein Ext2 | 82.018 | 32.573 | 5.7 | 4 | 4 | 4 | 0.84 | 2.8% |
| P36370 | Antigen peptide transporter 1 | 79.149 | 19.542 | 4 | 2 | 4 | 2 | 0.84 | 1.3% |
| P12843 | Insulin-like growth factor-binding protein 2 | 32.854 | 47.458 | 27 | 6 | 7 | 6 | 0.84 | 1.6% |
| P47853 | Biglycan | 41.706 | 121.71 | 33.3 | 10 | 15 | 10 | 0.84 | 1.3% |
| A0A0G2JUW8 | Leucyl-cystinyl aminopeptidase | 116.98 | 42.999 | 10.4 | 10 | 11 | 10 | 0.84 | 1.8% |
| Q3B7U9 | Peptidyl-prolyl cis-trans isomerase FKBP8 | 43.555 | 85.502 | 30 | 10 | 15 | 10 | 0.84 | 4.0% |
| D3ZLT1 | NADH dehydrogenase (Ubiquinone) 1 beta subcomplex, 7 (Predicted) | 16.568 | 21.312 | 21.2 | 2 | 4 | 2 | 0.83 | 10.2% |
| D4A3H9 | Protein Tmem87a | 64.316 | 14.828 | 2.5 | 2 | 2 | 2 | 0.83 | 2.5% |
| D3ZZY2 | Protein Utp14a | 91.043 | 10.759 | 3.7 | 3 | 3 | 3 | 0.83 | 2.8% |
| Q5M7T4 | Protein YIPF4 | 27.283 | 11.515 | 9.8 | 2 | 3 | 2 | 0.83 | 5.2% |
| D4ACM8 | Protein Fzd7 | 63.764 | 2.9276 | 1.7 | 1 | 2 | 1 | 0.83 | 2.5% |
| O54715 | V-type proton ATPase subunit S1 | 51.122 | 18.412 | 6.9 | 3 | 4 | 3 | 0.83 | 2.3% |
| A0A0G2KAD4 | Protein Lbh | 12.033 | 7.4987 | 18.3 | 1 | 2 | 1 | 0.83 | 2.9% |
| P31643 | Sodium- and chloride-dependent taurine transporter | 69.868 | 6.4411 | 4.2 | 2 | 3 | 2 | 0.83 | 2.1% |
| O08629 | Transcription intermediary factor 1-beta | 88.955 | 108.56 | 27.4 | 18 | 21 | 18 | 0.83 | 4.9% |
| Q499N5 | Acyl-CoA synthetase family member 2, mitochondrial | 67.886 | 25.926 | 13 | 7 | 8 | 7 | 0.83 | 1.7% |
| P18757 | Cystathionine gamma-lyase | 43.605 | 28.809 | 14.3 | 5 | 5 | 5 | 0.83 | 3.4% |
| G3V7I2 | Protein Rftn1 | 61.283 | 111.29 | 31.9 | 13 | 17 | 13 | 0.83 | 0.7% |
| O35806 | Latent-transforming growth factor beta-binding protein 2 | 189.87 | 16.88 | 2.8 | 6 | 6 | 6 | 0.83 | 2.7% |
| Q6P720 | Rho guanine nucleotide exchange factor 25 | 64.159 | 42.663 | 13.6 | 7 | 7 | 6 | 0.83 | 18.7% |
| G3V6X1 | Fibulin 2, isoform CRA_a | 131.3 | 12.639 | 2.9 | 3 | 3 | 3 | 0.83 | 1.8% |
| P19944 | 60S acidic ribosomal protein P1 | 11.498 | 61.615 | 89.5 | 4 | 17 | 4 | 0.83 | 1.6% |
| P80431 | Cytochrome c oxidase subunit 7B, mitochondrial | 8.9952 | 2.8766 | 10 | 2 | 2 | 2 | 0.83 | 9.8% |
| Q7TP77 | Aa2-277 | 19.406 | 7.2286 | 7.2 | 1 | 2 | 1 | 0.83 | 9.3% |
| P16975 | SPARC | 34.296 | 114.21 | 38.5 | 12 | 41 | 12 | 0.83 | 1.5% |
| Q5M848 | Calcium release-activated calcium channel protein 1 | 33.039 | 17.163 | 6.9 | 2 | 3 | 2 | 0.82 | 5.0% |
| Q5U2V8 | ER membrane protein complex subunit 3 | 29.98 | 14.552 | 11.9 | 4 | 5 | 4 | 0.82 | 9.1% |
| D3ZJN9 | Protein Evi5 | 92.862 | 10.918 | 4.2 | 3 | 3 | 3 | 0.82 | 1.6% |
| Q9Z118 | Polypyrimidine tract-binding protein 3 | 56.715 | 2.6614 | 9 | 5 | 7 | 2 | 0.82 | 1.0% |
| D3ZR49 | alpha-1,2-Mannosidase | 72.712 | 19.391 | 4.8 | 2 | 4 | 2 | 0.82 | 3.0% |
| Q6MG14 | Nurim | 29.381 | 9.5044 | 16.8 | 3 | 3 | 3 | 0.82 | 1.6% |
| F1M049 | Protein Atxn2 | 117.3 | 28.272 | 6.4 | 5 | 5 | 5 | 0.82 | 2.4% |
| O35260 | Nucleus accumbens-associated protein 1 | 56.449 | 1.8662 | 1.6 | 1 | 1 | 1 | 0.82 | 13.5% |
| Q5I0I8 | Nucleolar complex protein 4 homolog | 58.862 | 3.6995 | 3.3 | 2 | 2 | 2 | 0.82 | 7.0% |
| Q66HG8 | Protein Red | 65.587 | 20.739 | 8.3 | 4 | 5 | 4 | 0.82 | 6.0% |
| Q6QI25 | ORM1-like protein 3 | 17.462 | 2.0507 | 9.8 | 1 | 1 | 1 | 0.82 | 12.2% |
| A0A0G2K315 |  | 125.73 | 22.343 | 5.6 | 6 | 7 | 6 | 0.82 | 4.2% |
| Q6AYF4 | Integrin beta-6 | 85.958 | 2.9245 | 1.1 | 1 | 2 | 1 | 0.82 | 2.9% |
| D3ZWZ9 | G protein-coupled receptor 107 (Predicted) | 62.001 | 7.2395 | 4.2 | 2 | 2 | 2 | 0.81 | 10.4% |
| P23565 | Alpha-internexin | 56.115 | 8.7716 | 7.1 | 5 | 22 | 2 | 0.81 | 3.4% |
| Q76GL9 | Amino acid transporter | 55.9 | 84.42 | 19.7 | 8 | 11 | 8 | 0.81 | 1.9% |
| D3ZIX4 | Protein H1fx | 20.488 | 4.5585 | 9.4 | 2 | 3 | 2 | 0.81 | 2.5% |
| Q4V8C8 | Parafibromin | 60.602 | 9.8043 | 8.1 | 4 | 4 | 4 | 0.81 | 5.5% |
| B1WC06 | Mon1a protein | 62.154 | 2.8679 | 1.6 | 1 | 1 | 1 | 0.81 | 2.7% |
| P97603 | Neogenin | 150.64 | 11.387 | 1.9 | 2 | 2 | 2 | 0.81 | 9.9% |
| Q68FY1 | Nucleoporin NUP53 | 34.801 | 19.981 | 16 | 4 | 4 | 4 | 0.81 | 5.9% |
| P41777 | Nucleolar and coiled-body phosphoprotein 1 | 73.562 | 41.778 | 11.1 | 8 | 12 | 8 | 0.81 | 4.9% |
| Q62733 | Lamina-associated polypeptide 2, isoform beta | 50.277 | 85.062 | 29.9 | 9 | 13 | 9 | 0.80 | 0.6% |
| Q925G0 | Putative RNA-binding protein 3 | 16.855 | 11.333 | 22.6 | 3 | 6 | 3 | 0.80 | 7.2% |
| Q9JHW1 | Carboxypeptidase D | 152.61 | 16.691 | 6.4 | 8 | 9 | 8 | 0.80 | 12.8% |
| D4AA35 | Protein Asmtl | 24.25 | 7.7108 | 6.2 | 1 | 1 | 1 | 0.80 | 21.3% |
| Q6P7B7 | Protrudin | 44.947 | 5.5909 | 3 | 1 | 1 | 1 | 0.80 | 2.3% |
| Q5XI53 | CDK2-associated and cullin domain-containing protein 1 | 38.826 | 12.042 | 8.4 | 3 | 3 | 3 | 0.79 | 2.5% |
| D4A275 | Protein Gxylt2 | 51.352 | 2.7154 | 4.7 | 2 | 2 | 2 | 0.79 | 5.9% |
| A0A0G2KAX2 | Protein RGD1305455 | 62.82 | 2.0074 | 1.4 | 1 | 2 | 1 | 0.79 | 0.1% |
| O08837 | Cell division cycle 5-like protein | 92.216 | 52.856 | 14.7 | 8 | 10 | 8 | 0.79 | 0.1% |
| P51868 | Calsequestrin-2 | 47.838 | 24.925 | 15 | 7 | 10 | 7 | 0.79 | 1.2% |
| D3ZXC8 | Emopamil binding protein-like (Predicted), isoform CRA_a | 23.411 | 3.1933 | 4.4 | 1 | 1 | 1 | 0.79 | 9.0% |
| P24587 | A-kinase anchor protein 5 | 75.961 | 14.384 | 4.9 | 3 | 3 | 3 | 0.79 | 7.4% |
| P84109 | Sorbin and SH3 domain-containing protein 1 | 4.1073 | 2.332 | 54.3 | 2 | 2 | 1 | 0.79 | 0.5% |
| Q9QZ48 | Zinc finger and BTB domain-containing protein 7A | 60.543 | 11.319 | 2.6 | 1 | 2 | 1 | 0.79 | 2.9% |
| Q5PQT7 | Pleckstrin homology-like domain family A member 3 | 13.735 | 12.482 | 28.8 | 4 | 4 | 4 | 0.79 | 1.2% |
| A0A096MJY1 | Protein Gpc6 | 62.957 | 21.2 | 5.8 | 2 | 2 | 1 | 0.79 | 7.9% |
| D3ZSC1 | Protein Susd5 | 67.554 | 34.552 | 8.7 | 5 | 18 | 5 | 0.78 | 1.3% |
| Q8VHV8 | Selenoprotein S | 21.385 | 13.02 | 18.9 | 3 | 4 | 3 | 0.78 | 2.2% |
| O35910 | Monocarboxylate transporter 4 | 50.548 | 3.3458 | 3.2 | 2 | 2 | 2 | 0.78 | 19.0% |
| O35986 | Zinc finger Ran-binding domain-containing protein 2 | 37.35 | 4.7587 | 9.7 | 3 | 3 | 3 | 0.78 | 3.3% |
| A0A0G2JTD1 | Protein LOC100364568 | 154.31 | 12.813 | 1.4 | 2 | 2 | 2 | 0.78 | 2.5% |
| Q5PQK2 | Fusion, derived from t(1216) malignant liposarcoma (Human) | 52.673 | 83.992 | 15.4 | 7 | 20 | 4 | 0.78 | 7.7% |
| D4AE80 | Protein Dcp1a | 65.177 | 10.411 | 4.7 | 2 | 2 | 2 | 0.78 | 0.3% |
| B5DF51 | Membrane magnesium transporter 1 | 14.677 | 23.846 | 18.3 | 1 | 2 | 1 | 0.78 | 14.0% |
| M0R517 | Protein Lrrc74b | 40.628 | -2 | 2.9 | 1 | 1 | 1 | 0.78 | 3.1% |
| P23640 | Ras-related protein Rab-27A | 25.068 | 5.4715 | 5 | 1 | 1 | 1 | 0.77 | 2.2% |
| D3ZF56 | Protein Tmem134 | 21.55 | 3.1533 | 4.1 | 1 | 1 | 1 | 0.77 | 4.3% |
| Q63722 | Protein jagged-1 | 134.33 | 4.7825 | 2.1 | 3 | 3 | 3 | 0.76 | 1.8% |
| O35152 | BET1-like protein | 12.417 | 5.9374 | 13.5 | 1 | 2 | 1 | 0.76 | 22.2% |
| D3ZHW1 | LOC361614 (Predicted), isoform CRA_a | 26.157 | 3.8415 | 4.1 | 1 | 1 | 1 | 0.76 | 4.1% |
| P06760 | Beta-glucuronidase | 74.792 | 8.437 | 4.2 | 3 | 5 | 3 | 0.76 | 5.6% |
| Q5RJM0 | MKI67 FHA domain-interacting nucleolar phosphoprotein | 31.351 | 14.186 | 5.2 | 1 | 1 | 1 | 0.75 | 10.5% |
| P97544 | Lipid phosphate phosphohydrolase 3 | 35.318 | 8.5389 | 7.4 | 2 | 2 | 2 | 0.75 | 14.4% |
| Q62931 | Golgi SNAP receptor complex member 1 | 28.533 | 23.217 | 16.8 | 4 | 5 | 4 | 0.75 | 10.4% |
| Q5XID0 | Protein YIPF5 | 27.903 | 14.578 | 8.2 | 2 | 4 | 2 | 0.75 | 7.6% |
| P26051 | CD44 antigen | 55.945 | 48.984 | 13.1 | 5 | 14 | 5 | 0.75 | 3.2% |
| Q792S6 | Bcl-2-related ovarian killer protein | 23.456 | 20.101 | 17.8 | 3 | 3 | 3 | 0.74 | 3.9% |
| F1LXQ7 | Protein Arhgap21 | 216.37 | 20.085 | 3.4 | 5 | 5 | 5 | 0.74 | 3.0% |
| D3ZKK3 | Protein Cnst | 84.316 | 8.6783 | 1.5 | 1 | 1 | 1 | 0.74 | 10.9% |
| D4A7F2 | Protein Mycbp | 11.97 | 6.8335 | 10.7 | 1 | 1 | 1 | 0.73 | 3.7% |
| P86172 | NmrA-like family domain-containing protein 1 | 16.901 | 33.93 | 20.7 | 2 | 5 | 2 | 0.73 | 3.0% |
| Q9Z2P4 | Regulator of cell cycle RGCC | 14.766 | 80.086 | 51.8 | 5 | 7 | 5 | 0.73 | 9.1% |
| P70564 | Serpin B5 | 42.063 | 5.1841 | 2.7 | 1 | 1 | 1 | 0.73 | 0.6% |
| P08592 | Amyloid beta A4 protein | 86.703 | 41.165 | 6.8 | 4 | 5 | 4 | 0.72 | 6.3% |
| Q5U317 | Pre-mRNA 3-end-processing factor FIP1 | 60.187 | 10.163 | 2.4 | 1 | 1 | 1 | 0.72 | 0.6% |
| Q6AXN4 | Nucleoporin NDC1 | 75.712 | 2.4141 | 2.1 | 1 | 1 | 1 | 0.71 | 4.2% |
| Q76MV3 | Cytochrome C oxidase assembly protein COX17 | 6.784 | 17.092 | 41.3 | 2 | 3 | 2 | 0.71 | 2.2% |
| A0A0G2K7W4 | Protein Map4k4 | 153.19 | 28.506 | 8.9 | 11 | 12 | 8 | 0.71 | 4.7% |
| Q6GMN2 | Brain-specific angiogenesis inhibitor 1-associated protein 2 | 59.182 | 12.272 | 5 | 2 | 3 | 2 | 0.70 | 5.8% |
| D3ZWF4 | Protein Prr14 | 60.554 | 19.979 | 4.5 | 2 | 3 | 2 | 0.69 | 5.9% |
| D4A997 | HIV TAT specific factor 1 (Predicted) | 88.623 | 21.973 | 4.3 | 3 | 3 | 3 | 0.69 | 0.7% |
| D3ZZM1 | Protein LOC103694865 | 58.469 | 55.03 | 13 | 5 | 9 | 2 | 0.69 | 2.5% |
| Q6AY24 | Bone marrow stromal cell-derived ubiquitin-like protein | 40.562 | 4.5904 | 5.2 | 2 | 2 | 2 | 0.69 | 7.8% |
| O08875 | Serine/threonine-protein kinase DCLK1 | 47.68 | 6.7162 | 3.2 | 2 | 2 | 2 | 0.68 | 5.8% |
| F1M4U0 | Protein Ccnyl1 | 41.135 | 14.081 | 4.7 | 1 | 1 | 1 | 0.67 | 0.7% |
| D3ZC82 | Protein LOC687994 | 75.66 | 82.347 | 26.5 | 14 | 15 | 14 | 0.67 | 2.1% |
| B0BNB0 | Golt1b protein | 15.393 | 22.851 | 19.6 | 3 | 10 | 3 | 0.67 | 1.2% |
| Q6AYF8 | Protein Serpinb9 | 42.301 | 163.2 | 59.6 | 19 | 36 | 19 | 0.66 | 1.7% |
| F1LUV3 | Protein LOC291543 | 35.154 | 13.638 | 14.4 | 5 | 8 | 1 | 0.65 | 3.5% |
| Q4V8E4 | Coiled-coil domain-containing protein 104 | 39.59 | 36.168 | 9.9 | 2 | 2 | 2 | 0.63 | 3.3% |
| P19132 | Ferritin heavy chain | 21.126 | 25.493 | 17 | 2 | 3 | 2 | 0.62 | 0.5% |
| B2RYF7 | WAS protein family homolog 1 | 51.326 | 33.822 | 15.4 | 6 | 9 | 6 | 0.62 | 3.3% |
| A0A0G2JXC2 | Protein Fmnl3 | 106.96 | 10.526 | 6.2 | 5 | 10 | 1 | 0.61 | 5.5% |
| Q7TP54 | Ferritin light chain 1 | 144.71 | 168.29 | 8.2 | 8 | 27 | 8 | 0.59 | 1.8% |
| D4A110 | Protein Cmtm4 | 22.816 | 4.5589 | 4.8 | 1 | 2 | 1 | 0.59 | 6.0% |
| Q62910 | Synaptojanin-1 | 172.88 | 22.555 | 3.2 | 4 | 4 | 4 | 0.45 | 5.1% |
| P30823 | High affinity cationic amino acid transporter 1 | 67.266 | 6.8812 | 5.4 | 3 | 3 | 3 | 0.44 | 4.7% |
| D4A6W4 | Solute carrier family 52, riboflavin transporter, member 2 | 60.333 | 4.3906 | 2 | 1 | 1 | 1 | 0.43 | 1.7% |
